# Sequence analysis reveals a conserved extension in the capping enzyme of the alphavirus supergroup, and a homologous domain in nodaviruses

**DOI:** 10.1186/s13062-015-0050-0

**Published:** 2015-04-11

**Authors:** Tero Ahola, David G Karlin

**Affiliations:** Department of Food and Environmental Sciences, University of Helsinki, 00014 Helsinki, Finland; Department of Zoology, University of Oxford, Oxford, OX1 3PS UK; The Division of Structural Biology, Henry Wellcome Building, Roosevelt Drive, Oxford, OX3 7BN UK

**Keywords:** Methyltransferase, Guanylyltransferase, Capping, Alphavirus, Bromovirus, Nodavirus, Homology detection, Protein sequence analysis, Amphipathic alpha-helix, Viral replication factory, Chikungunya virus, Sindbis virus, Hepatitis E virus

## Abstract

**Background:**

Members of the alphavirus supergroup include human pathogens such as chikungunya virus, hepatitis E virus and rubella virus. They encode a capping enzyme with methyltransferase-guanylyltransferase (MTase-GTase) activity, which is an attractive drug target owing to its unique mechanism. However, its experimental study has proven very difficult.

**Results:**

We examined over 50 genera of viruses by sequence analyses*.* Earlier studies showed that the MTase-GTase contains a “Core” region conserved in sequence. We show that it is followed by a long extension, which we termed “Iceberg” region, whose secondary structure, but not sequence, is strikingly conserved throughout the alphavirus supergroup. Sequence analyses strongly suggest that the minimal capping domain corresponds to the Core and Iceberg regions combined, which is supported by earlier experimental data. The Iceberg region contains all known membrane association sites that contribute to the assembly of viral replication factories. We predict that it may also contain an overlooked, widely conserved membrane-binding amphipathic helix. Unexpectedly, we detected a sequence homolog of the alphavirus MTase-GTase in taxa related to nodaviruses and to chronic bee paralysis virus. The presence of a capping enzyme in nodaviruses is biologically consistent, since they have capped genomes but replicate in the cytoplasm, where no cellular capping enzyme is present. The putative MTase-GTase domain of nodaviruses also contains membrane-binding sites that may drive the assembly of viral replication factories, revealing an unsuspected parallel with the alphavirus supergroup.

**Conclusions:**

Our work will guide the functional analysis of the alphaviral MTase-GTase and the production of domains for structure determination. The identification of a homologous domain in a simple model system, nodaviruses, which replicate in numerous eukaryotic cell systems (yeast, flies, worms, mammals, and plants), can further help crack the function and structure of the enzyme.

**Reviewers:**

This article was reviewed by Valerian Dolja, Eugene Koonin and Sebastian Maurer-Stroh.

**Electronic supplementary material:**

The online version of this article (doi:10.1186/s13062-015-0050-0) contains supplementary material, which is available to authorized users.

## Background

The positive-strand (+ss) RNA viruses, i.e. viruses with a single-stranded RNA genome of the same polarity as mRNAs, constitute the large majority of known plant viruses, and also include major human and animal pathogens. They can be subdivided into large supergroups based on the presence of a shared set of domains in their replication proteins [1,2], such as the picornavirus, flavivirus, and alphavirus supergroups. +ssRNA viruses infecting eukaryotes replicate in the cytoplasm of infected cells in association with membranes [3] and utilize multiple strategies to express their proteins [4]. In particular, for many + ssRNA viruses, the viral mRNAs is capped, allowing efficient translation in eukaryotic cells [5]. Since cellular mRNA capping enzymes are located in the nucleus, many viruses that replicate in the cytoplasm encode their own capping enzymes [5].

**Figure 1:**
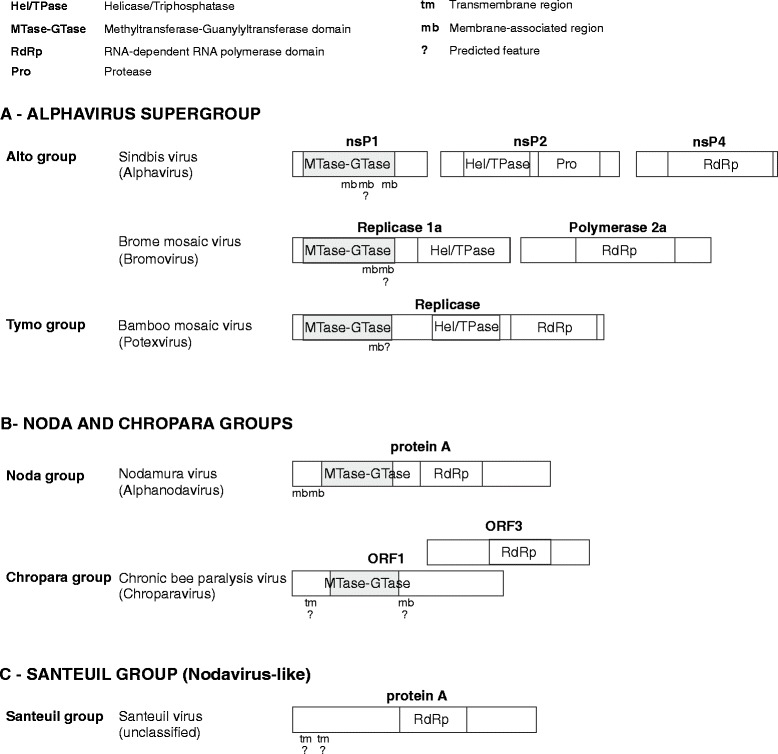
**Organization of the replicase domains in the viral groups studied.** The representation is approximately to scale, and incorporates the findings made in this study. See graphical legend (top) for abbreviations. Many members of the alphavirus supergroup encode a polyprotein, which is cleaved into several proteins or expressed as individual proteins, as exemplified by Sindbis virus and brome mosaic virus, respectively. In contrast, the BaMV replicase is not cleaved. For each virus, only proteins or domains containing a MTase-GTase, a protease, an RdRp, or a triphosphatase are shown. Membrane association segments (mb) that are predicted herein, rather than experimentally determined, are indicated by “?”.

The genomes of members of the alphavirus supergroup are 5′-capped, and the hallmark of this supergroup is the presence of a unique type of RNA capping enzyme [6,7], which has combined methyltransferase-guanylyltransferase (MTase-GTase) activity. The organization of the replicase proteins of three members of the alphavirus supergroup is shown in Figure 1A. The MTase-GTase is generally located upstream of a helicase domain; in *alphaviruses*, the viral polyprotein is cleaved, leading to the production of a shorter protein, nsp1, composed in good part of the MTase-GTase (Figure 1A). The capping enzyme was initially characterized as a guanine-7-MTase [8-10], and was thought to be encoded by a domain of ~200 amino acids (aas) containing 4 universally conserved residues [7], which we will refer to as the “Core” region. The secondary structure of the Core region and the location of its functionally important residues suggested that it could be structurally related to cellular methyltransferases with a Rossman fold [11]. The MTase-GTase of the alphavirus supergroup uses an atypical pathway for RNA capping (reviewed in [5]), which makes it an attractive drug target [12,13]. Cellular cap methyltransferase enzymes methylate GTP only after it has been transferred to the 5′ end of the mRNA [5]. In contrast, the alphaviral MTase-GTase first methylates GTP and only subsequently transfers it to the 5′ end of the viral mRNA [6,14,15]. The MTase-GTase forms a covalent complex with the product of the methyltransferase reaction, giving a covalent m^7^GMP-enzyme adduct with release of pyrophosphate [6]. Prior to MTase-GTase action, the first step of the alphavirus capping pathway is an RNA triphosphatase reaction, which is carried out by the viral helicase protein, using the same NTPase active site that powers the helicase [16-19].

Specific mutations within the Core region of the MTase-GTase can abolish both the MTase and/or the GTase activity [11,20,21]. However, the Core region alone is not sufficient for each enzymatic activity, and the minimal region required for activity probably also encompasses ~200 residues downstream of the Core region [11,22,21]. Interestingly, Koonin *et al.* discovered a region of that length downstream of the Core, the “Y domain”, conserved in sequence in three genera [23].

*Nodaviridae* is another family of + ssRNA viruses that also replicate in the cytoplasm [24,25] and have a capped genome [26,27], but the mechanism by which their genomes are capped is unknown. Members of the *Nodaviridae* are known to infect arthropods (genus *Alphanodavirus*) or fish (genus *Betanodavirus*) and have small bipartite genomes of altogether ~4.5 kb [28]. In the initial analysis of viral supergroups, they were classified as distantly related to picornaviruses [2,29]. However, in contrast to picornaviruses, which encode a polyprotein cleaved into several replication proteins, nodaviruses only encode a single replication protein of ~1000 aas, called protein A [28]. Protein A (Figure 1B) is organized into an N-terminal domain (or domains) of unknown function, which is a candidate for encoding the capping activity [30], and a C-terminal RNA-dependent RNA polymerase (RdRp), the activity of which has recently been demonstrated [31].

We re-analyzed the sequence properties of the MTase-GTase of the alphavirus supergroup. We show that it contains a region conserved in secondary structure, which we refer to as the “Iceberg region”, downstream of the Core region. Secondly, by using sensitive homology detection approaches [32-34], we discovered that the N-terminal moiety of the nodavirus protein A is homologous to the alphavirus MTase-GTase.

## Results

### The Core region of the alphavirus supergroup MTase-GTase contains 12 conserved predicted secondary elements and 4 conserved residues

**Figure 2:**
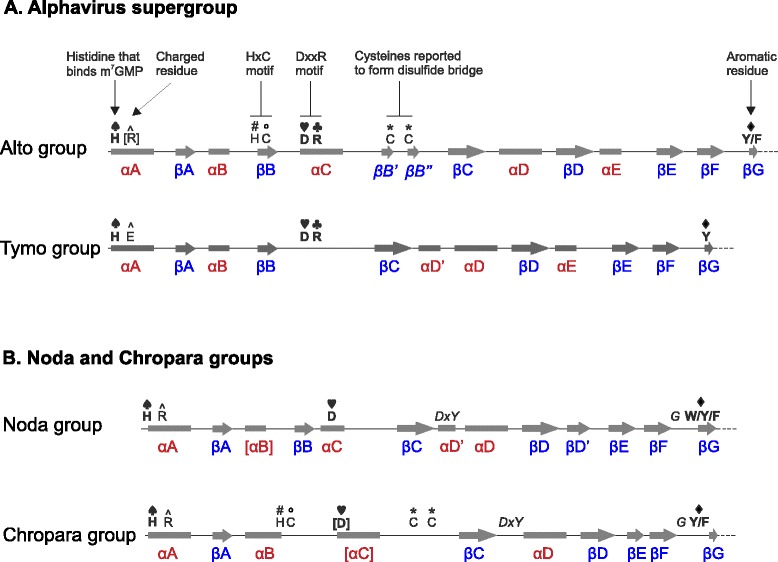
**Organization of the Core region of the MTase-GTase.** The representation is approximately to scale. α-helices and β-strands are indicated by rectangles and arrows, respectively. The charged residue (R^ or E^) in helix αA is located 7aas downstream of the histidine H♠, and the two cysteines (C*) upstream of βC are spaced by 6 aas. Secondary structure elements or positions that are not strictly conserved are indicated by brackets. Numbering of secondary elements in the noda and chropara groups is as for the alphavirus supergroup. The conserved positions unique to the noda and chropara groups are in italics.

We first re-analyzed the MTase-GTase of the alphavirus supergroup. This supergroup is divided into two groups on the basis of the sequence similarity of their RdRp and MTase-GTase [7]: the “alto” group and the “tymo” group (corresponding to the recently defined order *Tymovirales*). The N-terminus of the MTase-GTase (~140-250 aa) is called the Core region, and is conserved in sequence in both groups [7]. Figure 2A shows a summary of its predicted secondary structure and conserved residues. The full sequence alignments are in Additional file 1: Figures S2 and S3, for the alto and tymo groups, respectively. As reported previously [11], the Core region is composed of 9 main interspersed, predicted α-helices and β-strands, αA to αE and βA to βD, followed by three β-strands, βE to βG (Figure 2A). Accordingly, the recombinant, purified alphavirus MTase-GTase has a mixed α/β secondary structure [35]. In the Core region of the alphavirus supergroup, four residues are almost perfectly conserved, indicated by the four playing card symbols in Figure 2A [7]: a strictly conserved histidine (H♠) immediately upstream of αA, which most probably covalently binds the m^7^GMP [11,36]; a conserved aspartate (D♥) in αC, followed by an arginine (R♣) two aas downstream; and an almost strictly conserved Y residue (Y♦) at the beginning of βG, sometimes substituted by F. The D♥ and R♣ residues within αC form the DxxR motif (where x is any residue) [7], thought to be part of the binding site for the methyl donor substrate, S-adenosyl-methionine (also called SAM or AdoMet) [11].

In addition, we noticed a charged residue in helix αA (either R or E, marked by the symbol “^” in Figure 2A) in position +7 after the initial H♠, in most taxa with a few exceptions (*Alphavirus*, *Rubivirus*, *Benyvirus*, *Tetraviridae*, *Hepeviridae*) (see Additional file 1: Figure S2 and S3). In beet yellows closterovirus, the epitope 686–692 of the polyprotein, containing this residue, is exposed to the surface, suggesting that its conservation stems from functional, rather than structural, constraints [37]. The most striking difference between the alto and tymo groups is an insertion in the alto group of two predicted β-strands, βB’ and βB”, each containing a conserved cysteine (C*), between αC and βC. These cysteines, generally separated by six residues, are indicated by asterisks in Figure 2A and Additional file 1: Figure S2.

### The Core region of the alphavirus supergroup MTase-GTase is followed by a long C-terminal extension: the Iceberg region

**Figure 3:**
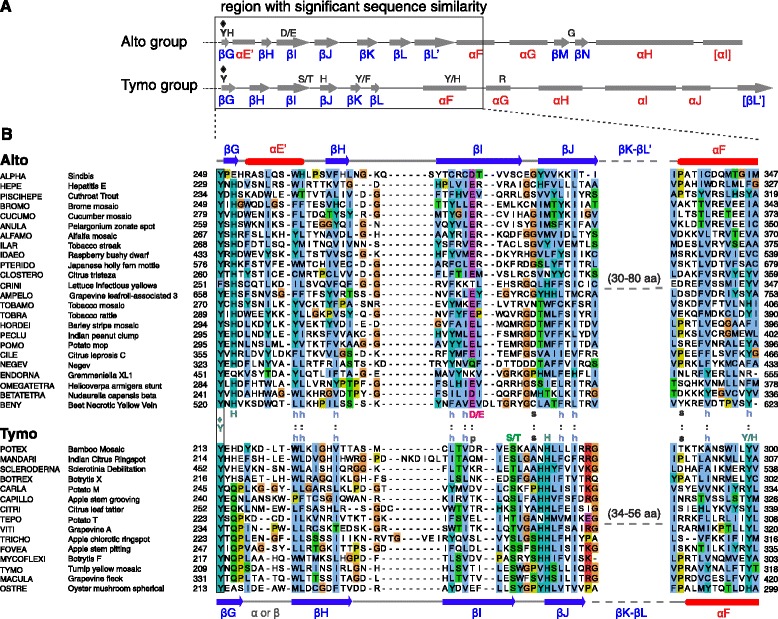
**The Iceberg region, downstream the MTase-GTase Core in the alphavirus supergroup. A**. Consensus secondary structure of the Iceberg region (which starts after the conserved Y♦ in strand βG) for the alto and tymo groups. Conventions are the same as in Figure 2. Residues conserved in each group are indicated. **B**. Sequence alignment of the region boxed in **A**, with the ClustalX coloring scheme [96]. The sequences of the tymo group were aligned to the set alignment of the alto group by using MAFFT with the --add option [94]. Substituted residues Y299 and W222 of BaMV are in bold (see text). Abbreviations: p, polar; h, hydrophobic; s, small.

Earlier studies reported some conservation in sequence [23] or secondary structure [38] downstream of the Core region in a few genera of the alto group. Since secondary structure is conserved over much greater evolutionary distances than primary sequence, we re-examined the predicted secondary structure downstream of the Core, taking advantage of a recently published software, PROMALS [39], which displays the secondary structure of multiply aligned sequences. We discovered that the region downstream of the Core has a similar secondary structure in the whole alphavirus supergroup. Figure 3A summarizes its predicted secondary structure in the alto and tymo groups (the actual alignments for each genus are in Additional file 1: Figures S2 (alto group) and S3 (tymo group), after strand βG). In both groups, this region comprises six to seven predicted β-strands (βG to βL’) followed by four to five α-helices (αF to αJ). The most noticeable difference between the two groups is the insertion, in the alto group, of a helix (αE’) between βG and βH, and of two strands (βM and βN) between helices αG and αH (Figure 3A).

The region conserved in secondary structure downstream of the Core region is longer (~155-260 aa) than the Core region itself (~140-250 aa). We called it the “Iceberg” region, akin to the immersed part of an Iceberg, which is larger than the visible part. We did not call it a “domain” because it does not appear to form a separate functional or folding unit (see below).

The Iceberg region of the alto group contains only three conserved or semi-conserved positions (Figure 3A and B; see also Additional file 1: Figure S2): H at the end of strand βG, D/E in the middle of strand βI, and G or another tiny aa (A or S) in the loop between βM and βN. The Iceberg region of the tymo group contains five conserved or semi-conserved positions (Figure 3A and Additional file 1: Figure S3): S/T in strand βI, H in strand βJ, Y/F at the end of strand βK, Y/H in helix αF, and R in helix αG. Of note, the end of the Iceberg region of *Tymoviridae* is divergent from that of other members of the tymo group, with which it has no sequence or secondary structure similarity after helix αG (see Additional file 1: Figure S3).

The Iceberg region of the alto and tymo groups have statistically significant sequence similarity over their first 90 aas (HHalign E-value: 3.3×10^-6^), confirming that they are homologous. In particular, a dozen positions are chemically similar in the Iceberg region of the alto and tymo groups (indicated by “:” in Figure 3B).

### The Iceberg region is essential for capping activity

The Iceberg region is essential for the MTase and GTase reactions, as can be seen from mutational analyses on recombinant capping domains (Table 1). In the alto group, these analyses were made on the protein nsP1 of the alphaviruses Sindbis virus and Semliki Forest virus (SFV) [11,22,21], whose Iceberg region extends from aa 250 to approximately aa 406. In the tymo group, analyses were made on a fragment (aa 1–442) of the replicase of bamboo mosaic virus (BaMV), comprising the full Iceberg region (aa 214–406) [20,40].

**Table 1:** **Published deletions or mutations within the Iceberg region of the MTase-GTase of the alphavirus supergroup that inhibit its enzymatic activities**

				**Activity compared to wild-type** ^**1**^	
**Group**	**Genus**	**Species**	**Substitution/Deletion**	**MTase** ^**2**^	**GTase** ^**3**^
Alto	Alphavirus	Semliki Forest virus [11,22]	K317A	5%	2%
			Δ270-537	<1%	<1%
			Δ430-537	<1%	<1%
		Sindbis virus [21]	Δ287-417	<1%	<1%
			Δ442-492	<1%	<1%
			W222A	5%	nd
			C234A	28%	15%
			W296A	17%	nd
			Y299A	58%	nd
			D310A	10%	5%
			W312A	37%	18%
Tymo	Potexvirus	Bamboo mosaic virus [40,20]	R316A	51%	19%
			Y338A	16%	nd
			F339A	20%	nd
			Y340A	23%	nd
			K344A	14%	14%
			W406A	14%	1%
			K409A	59%	40%

^1^In all cases, the mutant proteins were produced in *E. coli*. Only point substitutions (replacement by alanine) or deletions giving a significant reduction in activity (<60% of wild type) are shown. MTase: guanine-7-methyltransferase activity; GTase: covalent m^7^GMP binding activity; nd: not determined.

Internal deletions within the Iceberg region of alphaviruses destroyed enzymatic activity (Table 1). In addition, the most severe substitutions, K317A in SFV nsP1, and D310A and W406A in BaMV, reduced the MTAse and GTase activities by ≥90% (Table 1). These residues are highlighted in blue in Additional file 1: Figures S2 and S3, respectively. Several substitutions in the the BaMV Iceberg region also had drastic effects on the binding of the AdoMet methyl donor substrate [20]. Another important piece of evidence comes from a Sindbis virus mutant resistant to mycophenolic acid and ribavirin. These compounds lower the intracellular GTP concentration by inhibiting of the enzyme inosine monophosphate dehydrogenase, involved in the biosynthesis of GTP. Resistance to low GTP requires two mutations within Sindbis virus nsP1, S23N (just before the Core region) and V302M in the Iceberg region (in blue in Additional file 1: Figure S2) [41,42]. This strongly suggests that the Iceberg region (as well as the region upstream of the Core) takes part in binding the methyl acceptor substrate GTP.

We examined all taxa in the alphavirus supergroup to determine the boundaries of the minimal MTase-GTase domain. In alphavirus nsP1, the extension upstream of the Core region is very short (~37 aa, see Additional file 1: Figure S2), indicating that the minimal MTase-GTase domain starts very near the beginning of the Core region. In tymoviruses, the Iceberg region (aa 233–407) is almost immediately followed by a long region predicted disordered and then by a protease domain (not shown). Therefore, the minimal (smallest possible functional) MTase-GTase domain must closely correspond to the combined Core and Iceberg regions. This prediction is coherent with experimental findings. Catalytic activity is fully retained by truncated constructs of nsP1 of Sindbis virus (aa 1–448) [43] and of the BaMV replicase (aa 1–442) [44], which end only ~30-40 aa downstream of the Iceberg region. The minimal domain can be decorated with extensions necessary for catalytic activity. For instance, in brome mosaic virus, the Iceberg region ends around aa 424, but the shortest functional domain ends between aa 480 and 516 [38].

### The Iceberg region contains known and predicted membrane-binding sites

**Figure 4:**
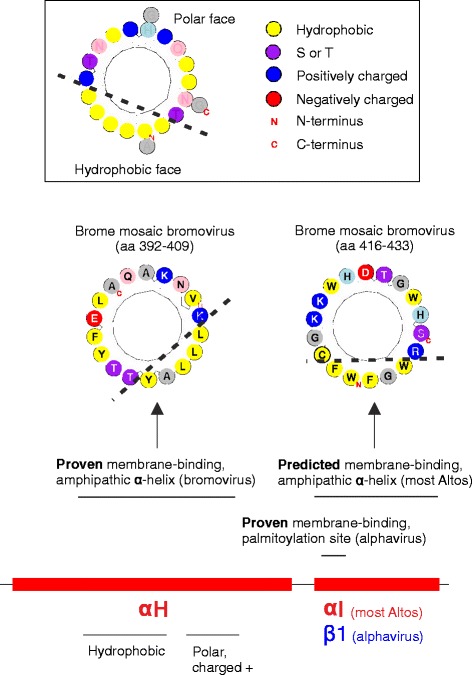
**Overview of the membrane-binding regions in the MTase-GTase of the alto group.** Known or predicted membrane association sites are indicated above the C-terminus of the Iceberg region of the alto group, with the same conventions as in Figure 3A. Amphipathic helices are depicted in helical wheel representation.

+ssRNA viruses form “viral replication factories”, compartments surrounded by remodeled cellular membranes in which viral replication takes place [3,45]. In many members of the alto group*,* it is the MTase-GTase domain that binds membranes and drives their rearrangement to form such replication factories [46,47]. This has been best described for the alphavirus SFV and for the bromovirus brome mosaic virus. SFV nsP1 binds membranes primarily through a region centered around αE’ [48,49], which forms an amphipathic α-helix [50,51], and secondarily through palmitoylation sites [52,22] located immediately after αH, in predicted strand β1 (Figure 4). In contrast, in brome mosaic virus 1a protein, the main membrane association segment is an amphipathic α-helix in the middle of αH (Figure 4) [46,53]. These amphipathic helices are doubly underlined in Additional file 1: Figure S2.

**Figure 5:**
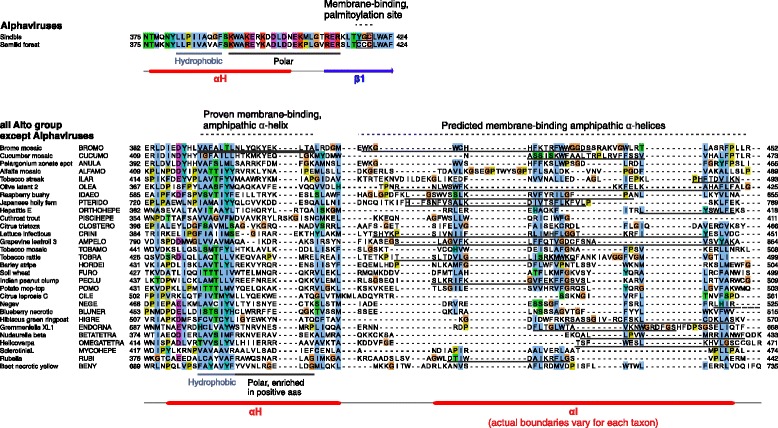
**Sequences of the C-terminus of the Iceberg region of the alto group, with known and predicted membrane-binding regions.** Sequence alignment of the last two secondary structure elements of the Iceberg region of the alto group. Conventions are the same as in Figure 3. The penultimate secondary element is αH in all taxa, and the last element is either a β-strand (β1), in alphaviruses, or an α-helix (αI) in other genera (see also Figure 4). The experimentally characterized, amphipathic helix of bromoviruses is doubly underlined. Amphipathic helices predicted by Heliquest are singly underlined. The sequences of each genus have no significant sequence similarity, and were aligned instead according to their secondary structure and hydrophobicity, using Promals [39].

We searched for other potential membrane-binding, amphipathic helices in the alto MTase-GTase by using Amphipaseek [54] and refining its predictions with Heliquest [55] (see Methods). The amphipathic helices predicted by Amphipaseek are thickly underlined in Additional file 1: Figure S2, and their sequence and location are in Additional file 1: Table S8A. The experimentally proven bromovirus amphipathic helix, located within αH (underlined in Figure 5) [46,53], is not detected by Amphipaseek or Heliquest, suggesting that it is of an unusual type. It is composed of a hydrophobic segment, followed by a polar segment with a positive charge. Strikingly, in all members of the alto group, the corresponding region also contains a hydrophobic segment followed by a positively charged segment (indicated below the alignment in Figure 5, bottom panel). The overall conservation of these features in the absence of detectable sequence conservation suggests that physico-chemical properties, but not the precise sequence, need to be conserved, owing to a functional or structural constraint. Therefore, this region of αH merits further study in other alto taxa, in particular of whether it binds membranes too.

In many taxa of the alto group, Amphipaseek [54] predicted an amphipathic α-helix in an adjacent region, within αI. Amphipaseek predictions are underlined in Additional file 1: Figure S2. They are highly unlikely to be due to a bias in the software, since αI generally has no detectable sequence similarity even in closely related taxa (Figure 5). Heliquest also predicted amphipathic helices in αI in many alto taxa, underlined in Figure 5. In particular, they are confidently predicted (Table 2) in the genera *cucumovirus* and *idaeovirus* (see helical view in Additional file 1: Figure S9). The cucumovirus predicted amphipathic helix was reported previously, and mutations designed to disrupt its membrane-binding or helical character abolished replication [56]. The bromovirus αI region also contains a predicted membrane-binding helix (Figures 4 and 5), downstream of the experimentally characterized amphipathic helix within αH. In summary, there may be at least two regions forming a membrane-binding, amphipathic helix in the MTase-GTase of the alto group (see Discussion). Known and predicted membrane-binding sites are summarized in Figure 1A.

**Table 2:** **Properties of known or selected, predicted membrane-binding, amphipathic α-helices in the MTase-GTase of the alto group**

**Genus**	**Species**	**Boundaries** ^**1**^ **(aa)**	**Predicted secondary element(s)**	**Mean Hydrophobicity (<H>)**	**Hydrophobic moment (<μH>)**	**Charge (z)**	**Heliquest Discriminating factor (D)** ^**2**^	**Status**
Alphavirus	Semliki Forest	245-264	αE’ and βH	0.44	0.28	+2	0.93	Experimentally proven, detected by Heliquest
Bromovirus	Brome mosaic	392-409	αH	0.54	0.27	+1	0.58	Experimentally proven, but not detected by Heliquest
Bromovirus	Brome mosaic	416-433	αI	0.60	0.30	+2	0.94	Predicted by Heliquest
Cucumovirus	Cucumber mosaic	446-468	αI	0.66	0.54	+3	1.50	Strongly predicted by Heliquest
Furovirus	Soil-borne wheat mosaic	291-312	βG, αE’ and βH	0.37	0.37	+4	1.67	Strongly predicted by Heliquest
Idaeovirus	Raspberry bushy dwarf	627-646	αI	0.7	0.57	+3	1.52	Strongly predicted by Heliquest

^1^Helical wheel representations for these helices are in Additional file 1: Figure S9.

^2^The Heliquest Discriminating factor D is equal to 0.944 < μH > +0.33z. The helix is predicted as “potential” lipid-binding amphipathic α-helix if 0.68 < D < 1.34, and as a reliable one if D ≥ 1.34 (see Methods for details).

In the tymo group, segment αI of the Iceberg region may also contain a membrane-binding amphipathic helix, according to Amphipaseek predictions (underlined in Additional file 1: Figure S3), most of which are supported by Heliquest (Additional file 1: Table S8B; see helical wheel views in Additional file 1: Figure S9). In particular, in the model species BaMV, a region within αI is strongly predicted by Heliquest (aa 358–379, Additional file 1: Figure S9). The only known substitution within this helix, W377A, had no effect on the MTase or GTase activity [20,40]. Note that despite their similar name and location, there is no evidence that helices αI are structurally analogous in the tymo and alto groups, in the absence of detectable sequence similarity.

### The replicases of recently discovered viruses related to *Nodaviridae* cluster in three groups: the noda, chropara, and santeuil groups

*Nodaviridae* have capped genomes but replicate in the cytoplasm, and therefore most probably encode a capping enzyme (see Background). An earlier study suggested that the N-terminus of the *Nodaviridae* replicase, upstream of the RdRp domain, was a candidate for encoding a capping activity, but could detect no significant similarity to known enzymes [30]. In recent years, new virus species encoding replicases related to *Nodaviridae* have been discovered. Their replicase clustered phylogenetically into three main groups:

1. a “noda” group, containing *Nodaviridae* (known to infect fish and insects), and two unclassified viruses of oomycetes, Sclerophthora macrospora virus A [57] and Plasmopara halstedii virus A [58]. In addition, we included in our analysis a metagenomics sequence from “betegovirus SF”, identified in waste water (Additional file 1: Table S1).
2. a “chropara” group, composed of chronic bee paralysis virus [59], anopheline-associated C virus [60], both members of the proposed genus *Chroparavirus* (P. Blanchard, personal communication), and of the unclassified Lake Sinai virus 1 and 2 [61], which infect insects;
3. a “santeuil” group composed of the unclassified Santeuil nodavirus, Orsay virus, and Le Blanc nodavirus, which infect nematodes [62,63].

The genomes of Sclerophthora macrospora virus A and of chronic bee paralysis virus are capped [57,59], like that of *Nodaviridae*, but the capping status of the other species is unknown.

### The replicase of the noda and chropara groups contains a putative MTase-GTase homologous to that of the alphavirus supergroup

We recently reported that the N-terminus of the replicase of the chropara group, upstream of the RdRp, was homologous to the Core region of the alphavirus MTase-GTase [33]. Since the RdRp domain of the noda and chropara groups are related [59], we examined the region of protein A upstream of the RdRp domain (aa 1–460 in Nodamura virus protein A). HHpred detected as first hit the PFAM family Vmethyltransf, corresponding to the MTase-GTase of the alphavirus supergroup, with marginal significance (E = 0.007). To validate this hit, we collected homologs of the N-terminus of protein A using iterative sequence searches (see Methods) and compared their alignment with that of the PFAM family Vmethyltransf. HHalign reported a significant similarity (E = 7×10^-8^), confirming that they are homologous. Thus, the replicases of the noda and chropara groups both contain a putative MTase-GTase related to that of alphaviruses. In both groups, we called the region having sequence similarity with the alphavirus enzyme the “Core” region, by analogy. In contrast, the santeuil group contained no detectable homolog of the alphavirus MTAse-GTase.

### Sequence features conserved in homologs of the alphavirus MTase-GTase

The high sensitivity of HHpred is due to the fact that it scores not only *sequence* similarity but also *secondary structure* similarity, better conserved over large evolutionary distances [64]. Consequently, the most distant homologs detected by HHpred often have few sequence motifs conserved. In particular, the sequences of the alphavirus MTase-GTase and of its Nodavirus homolog cannot be reliably aligned beyond a few conserved residues, discussed below. However, for information, we present in Additional file 1: Figure S6 an alignment of their Core region, displaying their predicted secondary structure. This alignment should be taken only as a very rough guide.

Figure 2B shows a summary of the predicted secondary structure and conserved residues of the Core region of the noda and chropara groups; positions conserved in both groups but absent in the alphavirus supergroup are in italics. The full sequence alignments are in Additional file 1: Figures S4 and S5 for the noda and chropara groups, respectively. The consensus secondary structure of the noda and chropara Core region (Figure 2B) is similar to that of the alto and tymo groups; the most noticeable difference is the absence of predicted helix αE in the noda and chropara groups (compare Figure 2A and B).

**Figure 6:**
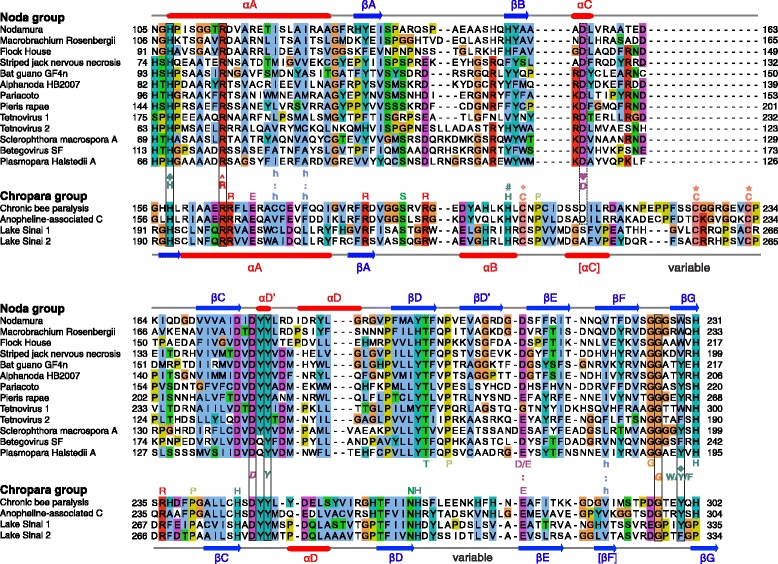
**Comparison between the MTase-GTase Core of the noda and chropara groups.** Conventions are the same as in Figures 2 and 3. Numbering of secondary elements is as for the alphavirus supergroup, and by analogy, the Core region ends at the position W/Y/F♦ in strand βG. The sequences of the chropara group were aligned to the set alignment of the noda group by using MAFFT with the --add option [94]. Conserved cysteines in the chropara group, which may be equivalent to those of the alto group, are indicated by an asterisk. The putative equivalent of the conserved D♥ of the alphavirus supergroup is boxed with a dashed line.

There is only one residue strictly conserved in the putative MTase-GTase of all groups: the N-terminal histidine (H♠) that is thought to be the covalent binding site for the m^7^GMP intermediate in the alphavirus supergroup (Figures 2 and 6). However, three other residues may be structurally or functionally equivalent in all groups: i) an arginine 7 aa downstream of H♠, strictly conserved in the noda and chropara groups (R114^ in Nodamura virus), may be analogous to the charged residue R^ or E^ of the alphavirus supergroup; ii) an aspartate conserved in helix αC of the noda group (D♥155 in Nodamura virus) may correspond to the D♥ of the alphavirus DxxR motif involved in SAM-binding. This aspartate is boxed with a dashed line in Figure 6. iii) a conserved aromatic position (W/Y/F♦) in strand βG of the noda and chropara groups (W♦229 in Nodamura virus) is consistently aligned, both by Psi-Coffee and HHalign, with the conserved Y♦ of the alphavirus supergroup.

### The putative MTase-GTase of the chropara group has noticeable similarities with its homologs from the noda and alto groups

As discussed above, there are few sequence motifs conserved between the putative MTase-GTase of the noda group and that of the alphavirus supergroup. In contrast, the chropara group presents noticeable similarities with both the noda and alto groups.

Figure 6 presents an alignment of the Core region of the noda and chropara groups (top and bottom panels, respectively). The most striking difference between them is a ~15aa insertion between αC and βC in the chropara group, which contains two conserved cysteines, spaced by 6 aa, marked by “ * “. In total, 6 residues are strictly conserved in both groups, boxed in Figure 6. In addition, the Core region of both groups has three strictly conserved residues that lack an equivalent in the alphavirus supergroup (in italics in Figures 2 and 6): a DxY motif, where x is any residue, between βC and αD’; and a glycine (G) between βF and βG (respectively D177, Y179, and G226 in Nodamura virus).

**Figure 7:**
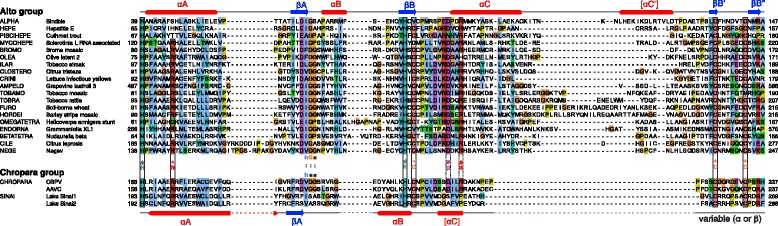
**Parallel between the MTase-GTase of the alto and chropara groups.** Conventions are the same as in Figures 2, 3 and 6. The sequences of the chropara group were aligned to the set alignment of the alto group by using MAFFT with the --add option [94].

We also noticed striking similarities between the Core regions of the chropara and alto groups (Figure 7; see also Figure 2), restricted to their N-terminal half. They both contain an H×C motif, where × is any aa. The histidine in this motif is indicated by “#” in Figure 7 (H#81 in Sindbis virus nsP1 and H#201 in the CBPV replicase). In most members of the alto and chropara groups, it is followed by a cysteine in position +2, indicated by “ ° ” in Figure 7. In both groups, the Core region also contains the pair of conserved cysteines mentioned above, indicated by “ * ”. These residues are functionally important in the alto group. In tomato mosaic tobamovirus, the cysteine pair (C*179 and C*186) formed disulfide bridges [65]. (In the closely related tobacco mosaic virus, cysteine 186 is substituted by a methionine in a few strains, including that presented in Figure 7). In the same virus, substitution of either of the three conserved cysteines by a serine strongly decreased membrane association, GTase activity, and viral replication [65]. In Sindbis virus, the mutation H#81A rendered the MTase inactive and was lethal for the virus [21]. Finally, in the bromoviruses brome mosaic virus and alfalfa mosaic virus, substitutions of the paired cysteines by a serine or alanine strongly decreased or abolished viral replication [66,67].

### The putative MTase-GTAse of Nodaviruses is involved in replication and self-interaction of the replicase

The capping activity of the nodavirus homolog of the MTase-GTase has not been demonstrated, but mutational data show its involvement in replication and self-interaction of protein A. Several residues of the Core region of protein A have been substituted experimentally in Flock House virus [68], in strands βF and βG. In particular, substitution of the aromatic position Y/F/W♦, the probable equivalent of the alphavirus Y♦ (W♦215A, in bold in Figure 6) abolished viral replication but not self-interaction of protein A; and substitution of a nearby tryptophan conserved in the noda group (W220A) abolished both self-interaction of protein A and viral replication [68]. These residues are highlighted in blue in Additional file 1: Figure S4.

### The putative MTase-GTase of nodaviruses has a C-terminal extension reminiscent of the Iceberg region of the alphavirus supergroup

**Figure 8:**
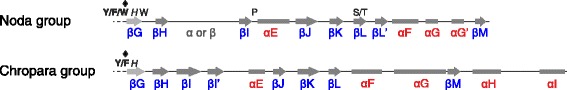
**C-terminal extension (Iceberg) of the Core MTase-GTase in the noda and chropara groups.** Conventions are the same as in Figures 2 and 3. The C-terminal extension starts after the conserved position W/Y/F♦ in strand βG.

In the noda and chropara groups, the Core region is followed by a C-terminal extension with a comparable predicted secondary structure (Figure 8; see also Additional file 1: Figures S4 and S5). This extension contains eight predicted β-strands (βH to βM), interspersed by two or three α-helices (αF to αG’). In the chropara group, it is immediately followed by a region predicted disordered, suggesting that the minimal capping domain is formed by the combination of the Core region and of this C-terminal extension, as in the alphavirus supergroup. The C-terminal extensions of the noda and chropara groups have no detectable sequence similarity to each other or to the Iceberg region. Nevertheless, their similar location and secondary structure (compare Figure 3A and Figure 8) suggest that they might be homologous but have diverged beyond recognition.

Substitutions within the C-terminal extension indicate its functional importance: in Flock House virus, the substitutions W222A and S231A abolished both self-interaction of protein A and viral replication, whereas E227A abolished only viral replication [68]. These residues are highlighted in blue in Additional file 1: Figure S4.

### Membrane association of the putative MTAse-GTase of the Noda and chropara groups differs from that of the alphavirus supergroup

The putative capping domain of the noda group associates with membranes, like that of the alphavirus supergroup [69-72], but by different mechanisms. In many species, membrane association is mediated by a segment upstream of the Core region, and sometimes by an additional segment in the Iceberg region or immediately downstream. Additional file 1: Table S8C and S8D present, respectively, membrane-binding amphipathic α-helices and transmembrane segments predicted in the noda and chropara groups. Additional file 1: Figure S9 presents helical wheel views of predicted amphipathic helices. Figure 1B summarizes membrane-binding segments in each group.

In the noda group, the mode of membrane association seems taxon-specific. It can occur through an N-terminal segment, which forms a transmembrane helix in a few species (e.g. in Flock House virus, aa 15–36 of protein A [71]), but an amphipathic helix in others, according to our predictions. For instance, aa 16–33 of Nodamura virus protein A [73,74] and aa 16–42 of Wuhan nodavirus protein A, which are part of the membrane association region, are predicted to form an amphipathic helix. In other taxa, different segments contribute instead to membrane association and mitochondrial targeting, such as region αD’-βE in Wuhan nodavirus, containing the DxY motif [72,74], and a genus-specific insertion between βH and βI in the Iceberg region of betanodaviruses [69,70]. The authors hypothesized that some of these regions span the membrane, but they are much more likely to be monotopically membrane-associated instead, since the different regions of the MTase-GTase, as well as the downstream RdRp, need to be placed on the same side of the membrane.

In contrast, all members of the chropara group have the same predicted mode of membrane association, which occurs through two regions: 1) a predicted transmembrane segment upstream of the Core region (underlined in Additional file 1: Figure S5), and 2) a predicted amphipathic, membrane-binding helix in αJ (thickly underlined in Additional file 1: Figure S5), immediately downstream of the Iceberg region, partially overlapping a short, conserved region specific to the chropara group.

## Discussion

The alphavirus supergroup contains over a dozen human pathogens. The MTase-GTase is an attractive drug target because it has a different mechanism from that of cellular enzymes [13,75], yet its membrane association and the lack of clear domain boundaries make it a difficult protein to work with. In addition, the lack of a 3D structure impedes the rational design of antiviral drugs. After the initial description of the Core region of the MTase-GTase [7], two studies identified a C-terminal extension conserved in sequence [23] or in secondary structure [38] in a few genera. We extend these results to the whole alphavirus supergroup and present precise boundaries that should guide the production of recombinant domains. We also describe previously overlooked features and conserved residues that will guide biochemical studies.

Most studies of the MTase-GTase have been carried out on two alphaviruses, Sindbis virus and SFV. However, our analysis shows that their MTase-GTase is divergent in sequence from that of the other members of the alto group and may thus not be a good representative. For instance, the MTase-GTase of alphaviruses does not encode the conserved residues R^, C^o^, or the H in position +2 of Y♦ (see Figure 3B and Figure 7); and it has three predicted β-strands instead of helix αI. Thus, additional model viruses are probably required for the alto group.

We also show that the *Nodaviridae* and related taxa contain a predicted MTase-GTase homologous to that of the alphavirus supergroup. This discovery will increase the chances of structure determination, which strongly depends on the number of homologs tested [76]. It will also facilitate the study of capping and of viral replication factories, because *Nodaviridae* are a simple, highly valuable model system of RNA virus replication. Indeed, their replication requires only one viral protein, protein A, and can take place in many types of eukaryotic cells, including those of yeast, flies, worms, mammals, and even plants [25].

### The phylogenetic affinities of *Nodaviridae*, an open question

The catalytic domain of viral RdRps is traditionally used to cluster + ssRNA viruses, since it is the only protein that they all share. Three main supergroups have been defined on the basis of the RdRp phylogeny [77]: the picornavirus-like, alphavirus-like, and the tombusvirus/flavivirus -like supergroups. Earlier studies clustered *Nodaviridae* with the picornavirus supergroup on the basis of their RdRp phylogeny [29,77]. However, the classification was presented as tentative, and nodaviral genomes have none of the hallmarks of the picornavirus supergroup [29]. Our findings further question this affinity, since members of the picornavirus supergroup do not encode an MTase-GTase [29]. In fact, our homology searches indicated a close similarity between the RdRp of the Noda and Chropara groups, while the RdRp of the Chropara group also had close similarity with that of Tombusviruses, as reported previously [60]. Thus, given the discrepant affinities of their putative MTase-GTase and RdRP, it is conceivable that the noda and chropara groups form a “nodavirus supergroup”. However, to obtain a reliable placement of the nodaviral RdRp, we will probably have to wait for more powerful sequence-based phylogeny approaches [78], or for the resolution of its 3D structure, to which novel structure-based phylogenetic approaches could be applied (e.g. [79,80]).

### Self-interaction, membrane association and membrane remodeling by the MTase-GTase domain

The MTase-GTase of the alphavirus supergroup and noda group has many functions besides capping, including self-association and membrane remodeling. In both cases, there is good evidence that it forms homodimers and probably higher multimers. In particular, the capping domain of the brome mosaic virus 1a protein forms multimers [38,81], although the site(s) of self-interaction have not been mapped so far. Likewise, the capping domain of *Nodaviridae* protein A self-interacts, perhaps through several independent regions, as suggested by deletion analyses in Wuhan Nodavirus [74] and Flock house virus [68]. Since the alphavirus and nodavirus capping domains must have a similar structure, as indicated by the current work, they may use similar interfaces to form multimers.

Multimerization of the capping domain appears essential, but not sufficient for the formation of the membrane structures that surround viral replication factories [81]. Intriguingly, these replication-induced membrane structures are closely similar in the alphavirus supergroup and in nodaviruses. They consist of “spherules”, round or bulb-shaped membrane invaginations (diameter 50–80 nm), connected to the cytoplasm by a narrow neck structure [82,83]. In an infected cell, there are thousands of spherules engaged in RNA synthesis, each containing an RNA template and several replication proteins. The viral proteins, including the capping domain, are essential for the formation of the spherule structures, but the mechanisms of membrane remodeling are still poorly understood, and several models have been proposed [84].

Membrane association of the replicase occurs mainly through the capping domain in at least two genera of the alto group. In alphavirus, the known membrane binding sites are an amphipathic helix in αE’ and a palmitoylation site downstream of αH. In bromovirus, membrane binding occurs via an amphipathic helix in αH (Figure 4). These sites are essential for virus replication and the formation of spherule structures [46,47]. We predict that many taxa could also encode a second, overlooked membrane-binding amphipathic helix in the αI region (Figures 4 and 5). In particular, bromoviruses are predicted to encode this second amphipathic helix (aa 416–433), in addition to the known one in αH (Figure 5). This is coherent with earlier observations that aa 388–422, which partially overlap with the second amphipathic helix, also contribute to membrane association of the bromovirus replicase [46]. Mutational data in a cucumovirus (closely related to bromovirus) also support the functional importance of the second helix [56].

### Limitations of our study

Our predictions of membrane-binding, amphipathic α-helices should be taken only as models to guide experiments, since predictors still suffer from a low sensitivity. For instance, they did not detect experimentally determined amphipathic helices of SFV and brome mosaic virus. Nevertheless, the potential amphipathic helix wihin αI in the alto group has strong support, since: 1) it is predicted by two programs relying on different methods, one of which, Amphipaseek, has very high specificity (above 95% [54]); 2) it is predicted in numerous taxa despite the lack of detectable sequence conservation, which seems to exclude a systematic bias in the software.

Another limitation is that we could not identify a known triphosphatase in genomes of the noda and chropara groups. Their genomes are unlikely to harbor a novel, conserved triphosphatase domain, since we found no region with conserved secondary structure outside of the MTase-GTase or RdRp domains. Therefore, these viruses probably use a different mechanism from that of the alphavirus supergroup. For instance, they may not require a triphosphatase, or may have evolved a triphosphatase activity *de novo* within the MTase-GTase or RdRp domain. Figure 1 presents a summary of the organization of the replicase proteins in the groups studied herein (alto, tymo, noda, chropara, and santeuil), showing the MTase-GTase, RdRp and helicase domain, when present.

## Conclusion

### Extending the reach of sequence-based homology detection

In conclusion, we have benefited from two major advances in sequence analysis programs, namely the incorporation of predicted secondary structure in homology detection software (HHpred [85]) and in multiple sequence alignment software (Promals [39]). These advances considerably extend the reach of sequence-based homology detection because secondary structure is conserved over considerable evolutionary distances, just as tertiary structure, and can be reasonably well predicted from sequence. For instance, the homology between the alphavirus and nodavirus MTase-GTase is detectable despite the fact that they have only 1 strictly conserved residue out of 300! Thus, sequence-based methods can play a renewed role in “unifying the viral universe” [86], along with methods based on comparing experimentally determined 3D structures. Such efforts would greatly benefit from software that could allow the simultaneous visualization on multiple alignments of additional predicted sequence features such as disordered regions, tm segments [87], coiled-coils, and low-complexity segments.

## Methods

### Homology searches

The accession numbers of the sequences used in this study, and the abbreviations of species names, are in Additional file 1: Table S1. To identify protein homologs, we used the following programs, based on sequence profile comparison, with an E-value cutoff of 10^−3^: HHpred [64], FFAS [88], HHblits [89] and Csi-blast [90,91] (5 iterations against the non-redundant NCBI database nr70). To determine whether two sets of homologous proteins were themselves homologous, we compared their Multiple Sequence Alignments (MSAs) using HHalign [92], with a cutoff E-value of 10^−5^.

### Multiple sequence alignment (MSA)

When presenting large sequence alignments, we tried to balance clarity of representation with comprehensiveness. Therefore, in the main figures, we present only alignments of selected representatives. In Additional file 1: Figures S2 to S6, we provide more comprehensive alignments, which include one representative of each genus and display their predicted secondary structure. Additional file 1: File S7 contains the corresponding alignments in text format.

We used Psi-Coffee [93] to align multiple sequences. To align a group of sequences to a reference alignment, we used the “--add” option of MAFFT [94]. All alignments are presented using Jalview [95] with the ClustalX colouring scheme [96]. We used PROMALS [39] to visualize secondary structure in the context of multiple alignments. We used two criteria to estimate the reliability of MSAs: 1) the core index, part of the standard output of Psi-coffee [93]; and 2) for the noda/chropara groups, the coherence between the alignments of each group separately and the alignment of both groups. We carried out phylogenetic analyses using phylogeny.fr [97] with default options.

### Prediction of protein structural features

We predicted disordered regions using MetaPrDOS [98], according to the principles described in [99]. We detected protein regions of low sequence complexity using SEG [100] with parameters 45/3.75/3.4 through the web server ANNIE [101].

We predicted transmembrane regions using two complementary methods, as described in [33]. For each virus, we compared the predictions of multiple programs on a single sequence (“vertical approach”), using ANNIE [101]. We also compared the prediction of a single program on several homologs (“horizontal” approach), using TM-coffee [87].

We predicted membrane-binding amphipathic α-helices using Amphipaseek [54] (parameters: high specificity/low sensitivity) and refining its predictions with Heliquest [55] as follows. For each helix predicted by Amphipaseek, we analyzed the region surrounding it by using the “analysis” function of Heliquest. Heliquest uses a Discriminating factor (D) to predict lipid-binding helices: D = 0.944 < μH > +0.33z, where < μH > is the hydrophobic moment [102] and z the net charge of the region considered. The helix is predicted as “potential” lipid-binding amphipathic α-helix if 0.68 < D < 1.34, and as a reliable one if D ≥ 1.34 [55]. On all reliably predicted amphipathic helices, we used the “screening” function of Heliquest [55] to identify similar amphipathic α-helices in other taxa. We also used Heliquest to plot helical wheel representations.

## Reviewers' comments

### Reviewer 1, first report (Valerian Dolja, Faculty of Center for Genome Research and Biocomputing, Oregon State University)

This manuscript presents two potentially interesting observations: i) identification of a conserved secondary structure region downstream from core capping enzyme of the viruses in aphavirus-like superfamily; ii) finding of a candidate capping enzyme region in viruses from family Nodaviridae that shows remote sequence and predicted structure similarity to that of a subset of viruses in alphavirus-like superfamily. These observations are likely to stimulate experimental work addressing functional significance of each of these protein regions.

Author’s response: *We thank the reviewer for his appreciation*.

I am much less enthusiastic about the searches for tentative amphipathic regions in the replicational proteins; these searches are unconvincing and should be deleted to sharpen major conclusions.

Author’s response: *We have greatly shortened and sharpened this section. In particular, we have eliminated the αE’ prediction, which we agree was only weakly supported. We did acknowledge the limits of the predictions in the Discussion section “Limitations of our study”. However, we have chosen to keep the new prediction of the amphipathic helix αI for several reasons:*

*- Our predictions are supported by two strong lines of evidence. First, they are made independently by two programs based on different principles; second, and more strikingly, the helices are predicted in the same region of αI in the absence of any detectable sequence similarity (see Figure*5*). This seems to exclude a systematic bias of the software.*

*- We acknowledge that in principle, the predicted αI helix could be truly amphipathic and yet not bind membranes, as happens in protein structures. However, from a biological perspective, the presence of a proven amphipathic membrane-binding helix nearby in the protein (in αH) increases the probability that the second amphipathic helix is also a bona fide membrane-binding helix.*

*- These predictions are meant to guide experimentalists in a field of research that is experimentally difficult. We do agree that they cannot be rigorously proven by sequence analyses since they do not come with E-values, but in that sense they arguably have more value for bench biologists to guide experiments, because they do require more expert insight and knowledge.*

In general, the manuscript would greatly benefit from removal of excessive speculations and technical details. Perhaps, this work will be best presented in a much shorter form such as ‘Hypothesis’ or ‘Discovery Note’ formats of Biology Direct.

Author’s response: *All three reviewers have made this point. We agree and have greatly shortened the article (by over 20%).*

I also propose to amend significantly the way in which taxonomic and evolutionary terms are used. I do not see any need in calling viruses in order Tymovirales ‘tymo group’, and I never heard the term ‘alto group’ , and do not consider this term proper or useful. Same applies to family Nodaviridae, not the ‘noda group’, not to mention ‘chropara’ or ‘santeuil’ group, the latter composed of a single poorly characterized virus.

Author’s response: *We did not mean to use these terms as taxonomic entities. We have clarified this point by stating more precisely: “Their replicase clustered phylogenetically into three main groups”.*

*The terms “alto” and “tymo” groups were coined in the first article describing the alphavirus MTase-GTase. We acknowledge that the term “alto” is not used often, and that “tymo” corresponds roughly to Tymovirales, but for instance oyster mushroom spherical virus is not yet taxonomically classified in it to our knowledge, though it is clearly related to Tymovirales. We used these groups for clarity since we found the first draft of our manuscript difficult to follow otherwise. The Santeuil group is in fact composed, for the moment, of three viruses (Santeuil virus, Orsay virus, and Le Blanc virus).*

I strongly disagree with the proposal to form a ‘Nodavirus supergroup’ based on yet unconfirmed hypothesis of the capping enzyme that is tentatively similar to that of the viruses in alphavirus-like superfamily. Even if confirmed, this similarity does not refute well-established affinity between RdRps of nodaviruses and viruses in picornavirus-like superfamily. There are many striking examples of horizontal gene transfer among viruses, which however, does not justify formation of new superfamilies for each such example. For instance, Potyviridae and Hypoviridae share superfamily 2 helicase with flaviviruses of the eponimous superfamily, but are confidently placed in the picornavirus-like superfamily on a strength of RdRp conservation along with presence of VPg and 3C-like protease.

Author’s response: *We do not agree that the affinity of the RdRPs of nodaviruses and the Picornavirus-like superfamily is well established. The affinity between Nodaviruses and sobemoviruses, within the Picornavirus superfamily, is in fact explicitly presented as tentative in the original article (Koonin EV: The phylogeny of RNA-dependent RNA polymerases of positive-strand RNA viruses. J Gen Virol 1991, 72(9):2197–2206) and in the most recent article: “[The] sobemovirus and nodavirus clade (clade 2) […] is only moderately supported.” (p 931 of Koonin EV, Wolf YI, Nagasaki K, Dolja VV: The Big Bang of picorna-like virus evolution antedates the radiation of eukaryotic supergroups. Nat Rev Microbiol 2008, 6:925–939). In addition, there are none of the hallmarks of the picornavirus supergroup (eg VPg and 3C-like protease) in Nodaviruses.*

*In fact, standard phylogenetic approaches are not designed for such evolutionary distances, and sequence motifs and PSSM scores by themselves, though suggestive, cannot provide a rigorously proven phylogenetic affinity. We are only aware of one recent method that attempts to rigorously evaluate distant phylogenies, PHYRN, and we hope that such methods will increasingly be developed and validated (we have unsuccessfully approached the authors to try and use PHYRN). We have added its reference when discussing this point: Bhardwaj G et al. PLoS One. 2012;7(4):e34261. PHYRN: a robust method for phylogenetic analysis of highly divergent sequences.*

*We applied these arguments to our own work and agree with your statement that it is premature to form a Nodavirus supergroup. We removed it from the title and article, and only mention it once in the Discussion, as a hypothesis.*

A rather minor correction has to do with the name of one of the viruses mentioned in the manuscript. It is Sclerophthora macrospora virus A or SmVA (not the other way around). This virus, together with later discovered Plasmopara virus, are actually hosted by oomycetes, not by fungi that belong to a different supergroup of eukaryotic organisms (Chromalveolates and Uniconts, respectively).

Author’s response: *We have corrected the mistakes. Thanks for pointing them out.*

### Reviewer 1, second report (Valerian Dolja, Faculty of Center for Genome Research and Biocomputing, Oregon State University)

I am mostly satisfied with the responses to my comments, and I agree that the moderate affinity of nodaviral RdRp with those in picornavirus-like superfamily in itself does not justify keeping nodaviruses as a part of this superfamily. Rather, nodaviruses could be considered as a deep-branching lineage within the alphavirus-like supergroup.

### Reviewer 2, first report (Eugene V. Koonin, Evolutionary Genomics Research Group, NCBI)

The authors of this manuscript report a finding that has substantial implications for the evolution of positive-strand RNA viruses, namely the presence of the capping enzyme that encompasses the methyltransferase and the guanylyl transferase domains in nodaviruses and a group they denote Chroparaviruses. To me, this is by far the most important result reported here.

Author’s response: *Thank you.*

The extension of the capping enzyme domain in alpha-like viruses is a useful but minor observation, in comparison. Accordingly, I think that it would be quite useful to change accents and to emphasize the above discovery in the title, abstract etc. The article is quite lengthy and detailed (in my opinion, excessively), and the principal message easily could be lost on the reader.

Author’s response: *We have greatly shortened the article, which we hope will give more emphasis to the main points. We did keep the order of presentation, though, since the article flowed more naturally this way. The discovery of the Iceberg extension, though less important evolutionary, should actually be of practical importance for many researchers, given the vast size of the alphavirus supergroup and the number of human pathogens and model viruses that it contains.*

I do have certain criticisms of the way the results are presented and discussed. The identification of the capping domains in nodaviruses and chroparaviruses is valid. However, to come to this conclusion, I have to reproduce the searches because the way these findings are described in the current manuscript is not really compelling. I strongly suggest that the authors present straightforward HHPred Prob values/E-values and even more important, show a multiple sequence alignment with the key motifs highlighted. This will be much more convincing than the current presentation that focuses mostly on secondary structure elements and is not easy to follow.

Author’s response: *We agree that the presentation with HHpred Evalues will be clearer and have rewritten accordingly.*

Concerning the second point, there are in fact *no* key motifs conserved in the MTase-GTase of all groups. The only residue strictly conserved in all homologs is the initial Histidine that binds m7GMP. In fact, an alignment of all homologs of the MTase-GTase (Additional file *1**: Figure S6) clearly shows a kind of “patchwork” evolution. The MTase-GTase of the chropara group has sequence motifs similar both to that of the noda group and to that of the alto group (in its N-terminal half), but the MTase-GTase of the noda group has no motif similar to that of the alto group. This is the reason why we did not present a sequence alignment between the noda group and alphavirus supergroup and had to focus on the secondary structure.*

HHpred and HHalign combine secondary structure information (conserved over much larger evolutionary distances) with sequence information. Accordingly, we have observed that alignments of homologs they detect are often much more divergent than alignments of traditional homologs detected by psi-blast or other profile-profile comparison homology detection tools that do not rely on secondary structure. Therefore, we do not expect the sequence alignment to be “convincing” by itself; rather, we have very carefully examined the matching of secondary structures and the correspondence of residues with known activity, such as the catalytic Histidine. We therefore present for the reader only the alignments of taxa in which some sequence motifs are conserved, i.e. noda/chropara and Chropara/alto. Nevertheless, for the interested reader, we do also present the alignment of *all* groups in two forms: annotated in Additional file*1**: File S7, and in text format in Additional file **1**: Table S8. We now explain these points more explicitly in the section “Sequences features conserved in homologs of the alphavirus MTase-GTase”.*

Furthermore, as far as I can see, the differences between nodaviruses and chroparaviruses are somehow glossed over in the text. These viruses have distinct genome organizations (in chroparaviruses, the capping enzyme and the RdRp are parts of different polypeptides), and the similarity of the capping enzyme sequences is not that high. The statement that they cluster in phylogenetic tress does not immediately convince me, with data not shown; I think showing such a tree with the proper bootstrap support (if such indeed exists) is more important than some of the illustrations in the current manuscript.

Author’s response: *See reply to reviewer 1 – we have removed these statements that were based, not on phylogenetic trees with bootstrap support (rare at these evolutionary distances), but on scores from homology searches and on conserved motifs.*

The absence of helicases in nodaviruses and chroparaviruses is not really surprising as it has been noticed 25 years ago that RNA viruses with genomes shorter than approximately 6 kb lack helicases that are apparently non-essential for the replication of such small genomes [1,2]. It is another matter that these viruses are the first that encompass the capping enzyme but not the helicase, and this is certainly of interest.

1. Gorbalenya AE, Koonin EV: Viral proteins containing the purine NTPbinding sequence pattern. Nucleic Acids Res 1989, 17(21):8413–8440.

2. Koonin EV, Dolja VV: Evolution and taxonomy of positive-strand RNA viruses: implications of comparative analysis of amino acid sequences. Grit Rev Biochem Mol Biol 1993, 28(5):375–430.

Author’s response: *Thank you for pointing out this fact.*

### Reviewer 2, second report (Eugene V. Koonin, Evolutionary Genomics Research Group, NCBI)

I am satisfied with the modifications made by the authors and have no further critical comments.

### Reviewer 3, first report (Sebastian Maurer-Stroh, Bioinformatics Institute, A*STAR Singapore)

This MS is an interesting piece of sequence analytic detective work where the authors systematically present a chain of evidence for an extended architecture of Alphavirus-like MTase-GTases that includes an extended “iceberg” region and varying membrane attachment factors such as palmitoyl anchors, transmembrane and amphipathic helices. They also propose existence of homologues in Nodaviruses. While several individual arguments may seem weak by themselves and only suggestive of homology at best, the overall architectural similarity based on the combined features does not seem too unconvincing.

Author’s response: *We thank the reviewer for his positive reception; this is indeed sequence detective work! In addition to bioinformatics lines of evidence, we also stressed the biological plausibility of our findings, i.e. the likely presence of a capping enzyme in Nodaviridae, and the habitual position of the enzyme, upstream of the RdRp domain.*

One of the major discussion points is the existence of an Alphavirus-like MTase-GTase homologue in Nodaviruses but the support for this in terms of significant sequence similarity is only referred to as previous work. Looking this reference up, it seems that the obtained hits came from HHpred against Pfam-A with E-values rather borderline close to generally accepted significance thresholds depending on the provided query and length. In the current MS they use additional methods such as HHalign to determine homology between families.

Suggestion 1: It would be interesting to try to strengthen and describe the Alphavirus to Nodavirus link using the same or additional approaches. Besides HHpred and HHalign, HHsenser may be useful here as it allows intermediate HMM-HMM hits in the same framework to cover greater distances in sequence space (e.g. if starting alignment created by HHpred for a query has too few sequences after removing redundancy).

Author’s response: *See reply to reviewers 1 and 2. We tried HHsenser but found that manual, iterative (cascade) searches detected more (i.e. all) homologs in each group. We now obtain robust and significant E-values. Thanks for the suggestion.*

The alignment in Figure 7 does not look very convincing. Except for a few key anchor points (H-x(6)-R and D-x-Y), the conservation of hydrophobic patterns which would be suggestive of a similar fold is rather bleak although such weak conservation would not be surprising for only remotely related fast evolving RNA viruses. It is important to strongly adhere to only aligning sequences with significant similarity not including low complexity regions as multiple alignment tools will gladly make alignments with marginal residue identities for any input sequences of similar length and composition. The text mentions that HHalign (profile to profile alignment) for the two families was significant but the Figure was created differently, with MAFFT using sequence to profile alignment. Suggestion 2: Possibly better to try MAFFT also in profile to profile mode (−−seed option) or, preferable, show the alignment provided by the same method that estimated the significant hit and show that the hit region is not dominated by low complexity.

Author’s response: *We agree that the sequence similarity per se is weak, but the significant scores reported by HHpred also include secondary structure similarity. See reply to reviewers 1 and 2; briefly, alignments of homologs detected by HHpred are often much more divergent than traditional alignments of homologs detected, for instance, by psi-blast, which does not rely on secondary structure. Therefore, we do not expect the sequence alignment to be visually “convincing” by itself. We have also checked that there is no “low complexity region” as defined by SEG. We did compare our alignment with that returned by profile-profile aligners such as Psi-coffee and HHalign, but found essentially no difference in the residues conserved in the alignment. Therefore, we have kept the alignment figures as is.*

A whole paragraph is dedicated to discuss the “conserved” residues in key anchor motifs of the MTase-Gtase core (e.g. H-x(6)-R and D-x-Y). It should be said here and critically discussed that, statistically, such short and simple motifs can easily occur by chance. For example, using ScanProsite (easy to find and use online) to search for motif H-x(6)-R only in Viruses in SwissProt (small database) already finds ~8500 hits in ~5200 viral proteins which should mostly not be methyltransferases. The ScanProsite online tool also has the nice feature of allowing searches against randomized databases (reversed, shuffled) which helps to gauge the expected number of random matches with a query motif. Despite the clear warning for potential false positives when arguing based on short simple residue patterns (e.g. only 2–3 amino acids and their distance constrained), a trained eye may find a few hidden gems using such searches for future work.

Author’s response: *We agree that the motifs are short and not meaningful per se. We only describe them to extract all information available to guide experiments, rather than as evidence, which is provided by the HHalign scores.*

Suggestion 3 (optional): Extend and further constrain the search motif (e.g. combine the motifs to reduce hits) and sift through results (filtered by, for example, biologically meaningful virus taxa with mRNA capping) to get ideas for further potential study targets to be verified with additional methods.

Author’s response: *We could not detect hidden gems using ScanProsite tool, but will keep the suggestion for future work. We suspect, though, that HHpred has become more powerful than pattern searches. It has the drawback to detect only homologs classified in PFAM families or whose structure is solved (since HHpred can use a database of profiles derived from the PDB), but the recent tool HHblits, based on the same principles, can detect in principle any homolog, and is very powerful too. We did examine other capping enzymes such as the Mononegavirales one, and found that it has a different predicted secondary structure pattern, precluding homology.*

Generally, also the additional discussed evidence with secondary structure similarity and the predicted amphipathic helix pattern would ideally be considered critically in comparison to expected random occurrence of the respective proposed patterns. If the complete architecture (e.g. core sequence, Y/iceberg regions, TM and amphipathic helices) could be combined in a probabilistic fashion with contributions of the individual features weighted by their statistical power, a better more integrative search for likely further remote homologues could be attempted. It is understood that this is not readily provided by the existing methods but would be a good idea to be implemented in one way or the other in future (obviously not the scope of this MS).

Author’s response: *We would love to have such an integrated method, as it would allow us to go even one step further in remote homology searches. Generally speaking, a method that could score contextual information, such as gene order, taxonomy, domain order… would be extremely powerful. We can only hope that readers of this exchange will develop it!*

### Reviewer 3, second report (Sebastian Maurer-Stroh, Bioinformatics Institute, A*STAR Singapore)

Overall, I am satisfied with the answers to the reviewer queries and revisions. Regarding reply to suggestion 1: “We tried HHsenser but found that manual, iterative (cascade) searches detected more (i.e. all) homologs in each group”. It is ok if the authors prefer full manual control over the iterative searches but just to add some more details for the benefit of interested readers on the efficacy of automated iterative searches as suggested. Submitting the same query as listed by the authors (http://www.ncbi.nlm.nih.gov/protein/13249661?report=fasta&to=460) to HHpred online (http://toolkit.tuebingen.mpg.de/hhpred) with PSI-BLAST option to collect query profile for later HMM-HMM comparison against database “PfamA_14Jan15” gives the top hit pfam01660 (Vmethyltransf Viral) with E-value 0.015. If the same search is preceded by HHsenser to automatically include more remote sequences into the query profile (which can be then forwarded to HHpred on same webserver), the E-value improves to 0.0017. Interestingly, running HHpred with the HHblits option (instead of PSI-BLAST) for query profile creation finds less hits for the profile (14 instead of 21) but nevertheless produces a good E-value of 0.0019. It should be noted that the latter method pairing is now the default option for HHpred at the webserver. One should never forget that E-values of search results for the same tool do depend on the database searched as well as additional parameters (especially different method steps).

Author’s response: *thank you for this detailed exposition of how E-values are sensitive to the methods and databases used. In particular, thanks for pointing out that using HHsenser upstream of HHpred is more sensitive than HHpred alone. We hope that interested readers, especially non- specialists, will take notice and use this combination of automated procedures. HHsenser is described in Nucleic Acids Res. 2006, 34:W374-8. HHsenser: exhaustive transitive profile search using HMM-HMM comparison. Söding J1, Remmert M, Biegert A, Lupas AN.*

Regarding reply to suggestion 3: “We suspect, though, that HHpred has become more powerful than pattern searches. … We did examine other capping enzymes such as the Mononegavirales one, and found that it has a different predicted secondary structure pattern, precluding homology”. Of course, HHpred is more powerful than pattern searches to establish homology/ancestry. I was indeed surprised to find many Mononegavirales sequences matching the motif but agree that common ancestry could be too far fetched here. Resolving the 3D structures of the respective domains in the different viral families should be of interest.

## Additional file

Additional file 1:
**Compilation of all supplementary figures and tables, in .zip format.**

## Abbreviations

aa: Amino acid
BaMV: Bamboo mosaic virus
MTase-GTase: Methyltransferase-guanylyltransferase
RdRp: RNA-dependent RNA polymerase
SAM: S-adenosyl-methionine
SFV: Semliki forest virus
+ssRNA virus: Positive, single-stranded RNA virus
