# Supplementary material for: Sequence analysis reveals a conserved extension in the capping enzyme of the alphavirus supergroup, and a homologous domain in nodaviruses
Source: Biol Direct. 2015 Apr 11;10:16. doi: 10.1186/s13062-015-0050-0 (PMC4392871; doi:10.1186/s13062-015-0050-0)
Supplement: Additional file 1: — Compilation of all supplementary figures and tables, in .zip format. [file 13062_2015_50_MOESM1_ESM.zip › Additional File 1/Figure S3 Tymo MTGT.docx]

**Figure S3. Alignment of the MTase-GTase of the Tymo group**

Conventions are the same as in Figure S2.

.. **N-terminal extension of the MTase-GTase**  **| Start of Core region**

6**8 75**

Conservation: 86 8 **H**♠ 8 6**E^** 8 8 5

BambooMosaic_POTEXVIRUS 3 LVSKVFDSI--TDPSLRAVLQEEAHSQVKQVLKDTALY---SRYALLPTAANTLERYAIPHNPF--STKLHTHAAAKALENDLYRTASYLLPK--ERVSFLFMKPSKMQYFRRRG-D--- 109

IndianCitrus_MANDARIVIRUS 3 TIRGAIDRI--TDTTVRTTLQEEACRQIRTELKNVEHV---NRYAIPPDAADALEHLGIGTNPF--SVKLHTHGACKAIENQLLYVVGTLLPK--ERVTMLFLKKAKLNFMKRCP-K--- 109

LoliumLatent_LOLAVIRUS 3 TLALNLAQY--NDPITKGIIAQESLRRVRPDLKAVVNV---NPYAIPTSAALVLEKLGIGTHPM--SLAVHPHAPCKAIENQILNTVGHLLPKE-QPVTFMAMKKSKLNMLRRHP-S--- 110

ShallotX_ALLEXIVIRUS 3 AVQKLFDQI--SDPNTKAGYSNACFEAAQRRPKKAMAI---APFSVTTPEALTLERFGITTSPF--ATTSHTHAADKIIENDCLTIIGHYLPKR-EAVTLIQLKRSKIHLLGRQP-S--- 110

BotrytisX_BOTREXVIRUS 3 ALRSLASDL--SDPTTKAAANEIAFKHFSDGIKSTKLS---FPYKVDESTAILLEKEGVLTNPF--STTLHTHAAEKMLENRTLNEVGKKLKVHTGPFTFIQFKTGKLGHVGRGH-N--- 111

TurnipYellow_TYMOVIRUS 2 AFQLALDAL--APTTHRDPSLHPILESTVDSIRSSIQT---YPWSIPKELLPLLNSYGIPTSGL--GTSHHPHAAHKTIETFLLCTHWSFQAT--TPSSVMFMKPSKFNKLAQVN-S--- 108

GrapevineFleck_MACULAVIRU 115 AFQPAIDFL--HNTIQKDTIASSIIAALNPSLTSSLTL---YPYALPPRWPSALNQAGIPATSY--GHQSHPHPIHKTIETHLLHEHWANRAT--LPSTVMFMKRSKFDKLRVSNAALVK 225

MaizeRayado_MARAFIVIRUS 9 HLLSGVESL--TPTTHRDTITAPIVESLATPLRRSLER---YPWSIPKEFHSFLHTCGVDISGF--GHAAHPHPVHKTIETHLLLDVWPNYAR--GPSDVMFIKPEKFAKLQSRQ-P--- 115

BotrytisF_MYCOFLEXIVIRUS 4 PLERAFASV--TQSFHKDATQGPTTNEYLVARAETQRY---APYAVRKGPAALLAQVGINTIHD--SPLSHSHPACYALNAFHFLKVIPRYLH--GKAEVWGTKDNWFRKLQAQIDSQ-- 112

DonkeyOrchidDORKYVIRUS 2 -LSEKYSAI--KDVNQQAILQTRYRAEYDNAVNETRKT---NPYYFDDASAEFLTGLGIPALPRHEATQLHRHGKNKAIENHLLANLKHKLS---GPCSFCFFKPAKLQLLGYNP-R--- 108

AppleStem_CAPILLOVIRUS 8 PLEIAINKL--P-SKQSDQLLSLTTDEIEKTLEVTNRF---FSFSITPEDQELLTKHGLTLAPI--GFKSHSHPISKMIENHLLYICVPSLLSSFKSVAFFSLRENKVDSFLKMHSVFSH 119

SclerotiniaDebilitation 241 RIGGSISEVVKTLGPDKEMIERDAVTEIKKEIAIVRKY---NPYHHSSKQQSALESYGIGSDPY--AVRSHTHAAEKAIENKLLDIVGMNLRRR-SVITMLWQKRNKAHLMGRSN-C--- 350

PotatoM_CARLAVIRUS 8 PMEDIVNC---FEPATQAVIANSAATLYKNFEEQHCQY---FNYYLSPLAKRKLSMAGIYLSPY--SAVVHSHPVCKTLENYILYSVLPSYIN--SSFYFVGIKERKLQLLKSKCKNL-- 115

AppleStemPitting_FOVEAVIR 8 AAEEVIAS---FTSEEQSRISTQAVLALTNVEKDKHDL---FNYALPELAKMRLFNSGIYLSPH--SYRPHSHPVCKTLENNILFNILPSYLD--NSFYLVSIKKNKVDFLKRRHPDL-- 115

CitrusLeaf_CITRIVIRUS 9 AIESILGN---FEKKHVDAIYNAAAQTILSHSEFRNKH---FAYSLNSYQKKIASKVGIELYPN--GYLPHSHPLSKIFENHLLFDVLPGVVNT-SRLVMCSIKESKVLVFKGIRDKSRR 119

ApricotVeinClearing 8 PQVNLLGS---FPQKHVEIIYNLQFERFKKICICRFCIFLTHSEKNQKKVASIRGWLGVPLHPT--PYLAHSHPFSKMLENHILLNVLPGHIT--GSWVFSSIKPSKVESLATKG-KK-- 117

GrapevineA_VITIVIRUS 11 AVSNLYTNG--SEESVKAIKELKSKRLLETETR-LDGL---FDYYIPDTLREILSGYGMEFSVH--SFQGHAHPVSKMIENHMLYRVAPNYFS--NNTLVVSCKEGKIKRLRLKN-AGN- 118

PotatoT_TEPOVIRUS 8 PAELFVQS---LPKEYAEACFKSHAANFQIRSDKGVGL---FDFACSSVVKERLTKAGIPVSAF--CNQEHSHPASKMIENHLLYNILPNYLNL-KNYTAISIKDSKVRKLLKNG-V--- 114

AppleChlorotic_TRICHOVIRU 8 PQEELLSR---LPQSQQEVISGFQYERIQKEEEKKVEN---FSFYLPEKTREWFTKSGVYLSPF--AYVNHSHPGCKTLENHLLFNVVASYISKYSYVACLSIKSNKMSKMERLGPNS-- 117

OysterMushroom 2 VGADLFAQI--SSSNHSDNIIGEALGRYRAHESRAVQV---ISSVLTPSEKALADRWAIPYSSS--PVGRVPHVMLKALENHRNVNILPNYLR--GRVTVLSMKPAKLAALRARC-PG-- 109

Consensus_ss: hhhhhhhh hhhhhhhhhhhhhhhhhhhhhhhh hhhhhhhhh hhhhhhhhhhhhhhhhhh eeeeee hhhhhhhhh

**αX αY αZ** **αA βA αB**

**122 125 ..Core region..**

Conservation: 9 **D**♥ **R**♣ 5 85766 5 5 57 5 67

BambooMosaic_POTEXVIRUS 110 --------------VDTFINADIVAKDLARYPLETI------------YP------------------------------RLPEITTKMAFIGDSLHYFQPEVLEHIFTSSPQLETLLAT 173

IndianCitrus_MANDARIVIRUS 110 -------------FQDIFLNQHIEPRDVSRYCDFNV------------QS------------------------------TSTSIPTHTAYISDTLHFMDRKDLVRLFINSPNLDTLYAT 174

LoliumLatent_LOLAVIRUS 111 -------------RADTFINPIYHPRDNVRYGLDPDPDQD-----ASITA------------------------------SFLEVKTSTAFMQDTLHYLTPEDLLDIFETSPKLENLIAS 182

ShallotX_ALLEXIVIRUS 111 --------------QDNFQNYCHEPKDVLRYGITHP-------------N------------------------------SCPVVNTEYAVLADTLHFMSPRQLYHLFSRNPKLERLFAT 173

BotrytisX_BOTREXVIRUS 112 -------------QGDQIINYAREAKDLSRFETLDT-------------A------------------------------VVPPVHNRVALLHDTLHFLSPRQLAQLFHHNPNLHLLYAT 175

TurnipYellow_TYMOVIRUS 109 -------------NFRELKNYRLHPNDSTRYPFTSP-------------D---------------------------------LPVFPTIFMHDALMYYHPSQIMDLFLRKPNLERLYAS 169

GrapevineFleck_MACULAVIRU 226 ----------SASNFLHLLNPILTARDADRYTHLPL------------PD------------------------------T--LPSTPLYFMHHSLMYFSPSQIAGLFLAAPFLERLYAS 291

MaizeRayado_MARAFIVIRUS 116 -------------NFAHLINYRLVPKDTTRYPSTST-------------N---------------------------------LPDCETVFMHDALMYYTPGQIADLFFLCPQLQKIYAS 176

BotrytisF_MYCOFLEXIVIRUS 113 ------------TTTLTHRNYAITARDHVRYPGT-V------------VD------------------------------HPGSCTAGTLFMHDALQYMTPLDVYTLFATSPEMHSLVAT 177

DonkeyOrchidDORKYVIRUS 109 -------------TAHEFLNPHITAKDLYRFPEDNI------------VA------------------------------DVFKFNYPTAFVQDALHYYSPGIILNIFEGNPGLRELYAT 173

AppleStem_CAPILLOVIRUS 120 ----------GKIKSLGMYNAIIDGKDKYRYGDVEFSSFRDRVIGLRDQC------------------------------LTRNKFPKVLFLHDELHFLSPFDMAFLFETIPEIDRVVAT 199

SclerotiniaDebilitation 351 --------------KDVYVNTIMEAKDLVRYDQFSF-------------G-------------------------------LPSVATSTAFIGDALHHMTPESVFDLFERSPNLMVLHAT 412

PotatoM_CARLAVIRUS 116 -------------DSVQVVNRYVTSADRMRYTNDFV------------PYG-SYEHECLVHK-GVGLDNEALRGLV---GPLRRHKAKNLFFHDELHYWSSKVLIDFL-DVMRPDKLLGT 204

AppleStemPitting_FOVEAVIR 116 -------------QMVETINRYISSIDKTRYGGFFH------------VSPSKISAKFKCDRRTGFEDDASLIDLI---PGCMEGARKRFFFHDELHYWTKEALITFL-DHVKPEVMLAS 206

CitrusLeaf_CITRIVIRUS 120 QVSDLNALNSLNNSHTSFINRLVASKDVSRYTEEAD------------AFFQSKK---------------GSPELFSRNFIKSLENKEAVFFHDEVHHWTKAQMFSFL-KSTKVKRFIFT 211

ApricotVeinClearing 118 -------------SVLKTINRLLCAKDFGRYDVDTD------------SSVIRSIS-------------REAPDILP-EPFVRAVKGRNVMIHDEVHHWTLDDMLGFL-DRARPNRFVFS 197

GrapevineA_VITIVIRUS 119 -----------RNLNFTQYNRLVHAKDHHRYENAFR------------ELDVG-------------------N-LT--NLINKESQSECIFIHDEVQYWSLDEMQRFLGSLSKVDRIVYS 193

PotatoT_TEPOVIRUS 115 -------------DSLETFNRLFSCKDALRYVDPET------------CDMD--------------------K------FIARVHHSTRIFLFDELHYWSMNSLSDFL-DRSNVKELLAT 182

AppleChlorotic_TRICHOVIRU 118 ------------VKTYDILNRLVTAKDKARYGPLAK------------PE------------------------------RSPCPKKTNIFIHDEIHYWSRDQLETFL-QVHRPKNLWAT 182

OysterMushroom 110 -------------VQFNLVNEIITPADHTRYTDPSP------------PP------------------------------D-FQGPGDMILVDDALQHWSRERIDAAF-RHFGGNRLIGT 172

Consensus_ss: eeeeeeeee eeeee hh hhhhhhhhhh eeeee

**βB**  Loop **βC** **αD’ αD βD..**

**..core region.. | Start of Iceberg region**

**213** 242 247

Conservation: 8 9 9 7 8 9 5 5 7 7 **Y**♦6 222 S/T H

BambooMosaic_POTEXVIRUS 174 IVLPPEATLRL-KSLYPEIYTLHYMP-QKFLYKPGGLSGGEYEHDYKDLT**W**L-KIGHVTTAS----------------------------MCLTVDRVESKAANHLLLIRRGRL------ 256

IndianCitrus_MANDARIVIRUS 175 IVLPVEAAYRQ-PSRYPDLYQINYDF-DGFQYIPGGHGGGAYHHEFSHLEWL-DVGHIHWRGPDN---------------------KDIQLTITAQMIESLGANHLFCFRRGNL------ 264

LoliumLatent_LOLAVIRUS 183 FVLPVEATRNM-KSLYPDLYSIHYTH-GGFQWAPSGHLGDAYFHEPWQLYWL-RCGSLTRLIEEETTTTVQPPPGFEGEAYTRVRKEVRELKIYAERVTSIGAHHLFIFSRNAK------ 293

ShallotX_ALLEXIVIRUS 174 LVLPIEAQHRL-PSLFPDVYRLEYYK-DHFAYMPGGHGGGAYVHSYGTLKWL-DTAQVGPVDYTKSSI---------------TNPWPITDYLSIEKIETKAAHHIMFIQRTRAQV---- 271

BotrytisX_BOTREXVIRUS 176 LVLPVEALHNL-PSLNPHAYTLEYFSNGDFAYMPGGHAGSGYYHSAETLHWL-RAGQIKLGH----------------------------FSLSLNKEDSFGAHHIFTVTRQFI------ 259

TurnipYellow_TYMOVIRUS 170 LVVPPEAHLSD-QSFYPKLYTYTTTR-HTLHYVPEGHEAGSYNQPSDAHSWL-RINSIRLGN----------------------------HHLSVTILESWGPVHSLLIQRGTPPPDPSL 258

GrapevineFleck_MACULAVIRU 292 LVLPAESTIGS-HPFFPSLYRYRTTG-EHLHYVLEGNPSSSYTQPLTATQWL-TTSSITAGD----------------------------LHLTVTVLESWFSVHSILITRGVRPL---- 376

MaizeRayado_MARAFIVIRUS 177 VVVPAESSFTH-LSLHPEIYRFRFQG-SDLVYEPEGNPAANYTQPRSALDWL-QTTGFTVGH----------------------------EFFSVTLLDSFGPVHSLLIQRGRPPV---- 261

BotrytisF_MYCOFLEXIVIRUS 178 AVIPPESVDRL-PAFWPELYQLAYYE-DHLCYAPDGNFADAYNQPLAAHQWM-TMKSLHGPD----------------------------FTLSVDVPASRYSHHIFVISKRPG------ 260

DonkeyOrchidDORKYVIRUS 174 VVIPVEIMHKH-ASFHPSLYTIEYHDDDEFSYIPESSAAGAYTQGLACLNWL-KYSNFSRGE----------------------------TKVSSTLLETLGAHHVLHFVRGEF------ 257

AppleStem_CAPILLOVIRUS 200 TVFPIELLFGDKVSKEPRVYTYKVHG-SSFSFYPDGVASECYEQNLANSKWPFTCSGIQWAN----------------------------RKIRVTKLQSLFAHHVFSFDRGRA------ 284

SclerotiniaDebilitation 413 IVIPPETLLKC-RSSNPELYSLRYYD-DKFVYIPEGHAGGSYVHEVKNSNWL-AISHIQRGG----------------------------KFLTVKRLETLAAHHYFVIVKGKV------ 495

PotatoM_CARLAVIRUS 205 VVYPPELLFKPTRSLNEWCYTYDIVG-DTLMFFPDGVQSEGYQQPLKGGYLL-GARSLKLPD---------------------------GTVYMVDVLCSKFPHHLISITKGEA------ 289

AppleStemPitting_FOVEAVIR 207 IVFPPEILAGAKESLNPWCYTFRIVG-KDLVFFPDGEQSEAYIQPVAGSYLL-RTGKITTPS---------------------------GDIFQLDLLKSSFSHHLISITKGEA------ 291

CitrusLeaf_CITRIVIRUS 212 VVYPPEILKKFANSQNPKVYDFKVDK-GRLFFFPDGVKTEAYEQKLNMEWLF-SASHLRSGD----------------------------CVWTVTRHKSIYAHHLFEISIGEL------ 295

ApricotVeinClearing 198 VVYPVELLAGILESQNPKMYKFQDSKSDKIVFFPDGRASEGYEQRANLRWLF-CASHFRTSG----------------------------SIWTVKRIYSAYSHHLFEVVPGNY------ 282

GrapevineA_VITIVIRUS 194 IIYPAEVEAGYSQSLFPEAYTFDLKE-GRLIWYPDGKAEGAYTQPINPWLLR-CSKTEDSKG----------------------------RSWTITKLQTVGAHHLFSAIRGSY------ 277

PotatoT_TEPOVIRUS 183 IVFPIEILLGSKRSLNPELYEFEISR-GKLHFFPDGCTSESYSQPK-DCDIL-KVNRIVTKT---------------------------GKIFSVELIHTIGANHMVMIKEGSF------ 266

AppleChlorotic_TRICHOVIRU 183 LVFPPEILAGYKSSVLPFLYQFEIHG-KDLVYMPDGVRSESYTQPLENGFLL-SSSSIIIKNRVT----------------------GVEIRYQVSLVYSLGSHHLFHIYPAEDL----- 273

OysterMushroom 173 NIHPDEVRSGH-ASRYPDLYTIEYLPNRRYGLLSHRHKSASYEASIDEAWML-DCGDFTVDD----------------------------RAYDVEFLLSYGPYHLVTVVPADGH----- 257

Consensus_ss: eee hhhh eeeeeee eeeee eeee ee ee ee eeeeeeeeee eeeeee

**..βD αE βE βF βG βH βI βJ**

**..Iceberg region..**

299 316

Conservation: Y 310 312 R5

BambooMosaic_POTEXVIRUS 257 -----------------KLSTYRSYDTPEPLVVVPKI-FLP--PKYNAQKP-ITKTKANSWIL**Y**VKSAGEPKIR**D**V**W**AKL**R**QTIANS-ELNQYEPAELLLLTNYFYVLGKLD-------- 346

IndianCitrus_MANDARIVIRUS 265 -----------------RTPRVRTFGRDTQ-VLLPKI-FRP--VDKNFNRA-IPLTLANKMLLYAKSINTVTFRDVVAKTRQLMKDK-ELETYTGADLLHMANYFFVVGALS-------- 353

LoliumLatent_LOLAVIRUS 294 -----------------ATPRVRSYSQNGK-WVTLPRIFRP--VSHNVQTP-LKQEVANSLMLYTYAVR-PSLKDVAAKVRQKFDEK-DLAEHSPLEITHLINYIYYIDQRA-------- 382

ShallotX_ALLEXIVIRUS 272 ---------------DWPLPPIWVYHASEY-VKLPLI-FYP--PEANVQKT-YPHTLIKRMQLYCFSVKAVSLRDIFAKLRQVIETQ-ELVRYSMADLIRLANYFLFITGMN-------- 362

BotrytisX_BOTREXVIRUS 260 -----------------PPAPRYLYAETEL-VTLHDI-FYP--ADSNVQRP-YPATLINRMELYCRSVKAVSLRDIFAKFRQVTETA-QLRHMRISDVIRLANYFLFSASLS-------- 348

TurnipYellow_TYMOVIRUS 259 QAPPTLMXSDLFRSYQEPRLDVVSFRIPDA-IELPQA-TFL--QQPLRDRL-VPRAVYNALFTYTRAVRTLRTSDPAAFVRMHSSKP-DHDWVTSNAWDNLQTFALLNVPLR-------- 364

GrapevineFleck_MACULAVIRU 377 ----------------ELPRDIISLPSPDA-VLLPNP-SAF--DIPLRSRL-VPRDVCESLFVYVRAVRTLRTTDPAGFIRTQSNKA-EFDWVTAEAWDHLAQFALLTAPVR-------- 466

MaizeRayado_MARAFIVIRUS 262 ----------------FQAEDIASFRVPDA-VALPAP-ASL--HQDLRHRL-VPRKVYDALFNYVRAVRL-RVTDPAGFVRTQVGKP-EYSWVTSSAWDNLQHFALQTAAVR-------- 350

BotrytisF_MYCOFLEXIVIRUS 261 -----------------LPDTHRDFMCPDL-VELPKD-FFP--GANKADKL-LPRTLVNQMVEYAASVKRATIKDATSRTRAYVKDQ-KYAAVTP-----VQQMYLSWFGSGLSQMQFPD 352

DonkeyOrchidDORKYVIRUS 258 -----------------LPQKRRLFQHPPL-VKLPPV-YCV--GRFNSHKP-FPKTLVQVLLLYAHTLKEIRDVDIWAKLRQQIPKN-AIDDYDVGDLTLLADYVVVTAKLS-------- 346

AppleStem_CAPILLOVIRUS 285 ------------------CNEFNHFDKPSC-LLAEEM-RLL--TKRFDKAV-INRSTVSSLSTYMACLKTANAASAVAKLRQLEKR--D---LYPDELNFVYSFGEHFKNFG-------- 368

SclerotiniaDebilitation 496 -----------------ETDSIRVFQSPSQ-VELLDI-FAD--RQSNVRCS-LDHAFAIKMERYVHSLKRLELADVTAKTRQLLSSE-ELLQYSPTDLVKIDNYFYFLAHTS-------- 584

PotatoM_CARLAVIRUS 290 -----------------AAPTHRAFGPFEA-VASEAL-KATLSPDYPCAFP-VSYEVVNKIYRYLRTLKKPDEQSAIAKLSQIIAEP-S-----GREIDFVECFARLVIHNS-------- 375

AppleStemPitting_FOVEAVIR 292 -----------------IGQKMRFFNGFEA-VAMKGL-NPLR-RKVESCLP-ISKNTILKIYRYLRTLKKPDLQSAMAKLSQVCKDP-N-----GYEIKFFEEFSKLCLKCD-------- 376

CitrusLeaf_CITRIVIRUS 296 -----------------VTDSKLFFSDYNS-IDMSKI-FLDR-FRSYEVFP-ISIEHLYKVYSYLLCLKKPDLESGLAKLRQIIGDDVE-----IKEFLFFEQFCKRLIERQ-------- 381

ApricotVeinClearing 283 -----------------FTDEIRFFNDFET-IDLQCI-FKSR-FLCRDFVP-ISKDLVERVYSYLICLKKPDMQSAMAKLKQLMGD--D------LDVRVQVFFRSLVHRIL-------- 365

GrapevineA_VITIVIRUS 278 -----------------LTEESYKYDNFTI-INPNDI-LRG--RRGSKPLY-LRARMIKPTLLYLLALKKSDSNSAVAKLRMLSSRE-EN----MDEALFVAQLAKQIRDT--------- 361

PotatoT_TEPOVIRUS 267 -----------------DVDSERFFDRSSA-LTT-SL-LMP--TRAGKALR-IRRKFLLRLIIYLFSLKKPDHHSAIAKIRQSSDDS-IF----CDEIMLADHVGKIFEKLD-------- 350

AppleChlorotic_TRICHOVIRU 274 -----------------MKEEVRRFGPYDL-FDVGSL-FVK--PVRVPIQD-FPLSVFKKIFIYMSSLKKPDVQSAVAKLRQLSDAD-I-----SIESVFMIQEFASRIEKN-------- 357

OysterMushroom 258 -----------------VVRTYRFFDAPEV-VQLPDI-P----GLPNLSSPWFPALMYTQTLDHAGSLKQLGERDGKARIRGLRMTP-EGRRIPYATWERLLTCAKLAGL-H-------- 344

Consensus_ss: eee ee hhhhhhhhhhhhhh hhhhhhhh hhhhhhhhhhhhhhh

**βK βL αF αG αH**

**End of sequence similarity with Alto group|**

**End of the Iceberg region and of the MTase-GTase|**

Conservation: 406

BambooMosaic_POTEXVIRUS 347 -SITSFETLLGDNILKR-LFRPAIAKI--QELRHWVSG---PTAF-----MQLYKALQLVDV---DFTFEVTKN**W**ETKQ----------------------------------------- 410

IndianCitrus_MANDARIVIRUS 354 -GVNSYDQMLGLSAWEA-CTMALKNTV--TNLWERITG---KREF-----GKLLEALEWETL---TYSRQVTQKYVGGTP---------------------------------------- 418

LoliumLatent_LOLAVIRUS 383 -YLTNDDDILSDNLLKRWIFTPIQAAY--KKAKGFLLG---PDDF-----QKLLKALEWQPV---TFDYAVDHYKSNPWRIHASRTGAKMRQLKNFLSRTTGLCEAVEDDALEGSELLKE 488

ShallotX_ALLEXIVIRUS 363 -QVSDYESPLLENLFGK-MCASIRMRL--RTFFQNLLG---KTSY-----AALLTVTDVIPV---HFTTQPKRREAVGEL---------------------------------------- 427

BotrytisX_BOTREXVIRUS 349 -GTNDYPSLVGHGLFKK-MRVSFQERV--KEVLAPLVG---HTQY-----RTLMSFVQPKPF---TFSLTPVTFHAAQGR---------------------------------------- 413

TurnipYellow_TYMOVIRUS 365 -PNVVYHVLQS--PIAS-LSLYLRQHW--RRLTATAVP---ILSF-----LTLLQRFLPLPI---P--LAEVKS---------------------------------------------- 419

GrapevineFleck_MACULAVIRU 467 -PNTYFLPLLS--PLAV-VRHWLFRKQ--RPIFATLTL---LSAS-----TAAAIPIAIARL---R--THSVTQ---------------------------------------------- 521

MaizeRayado_MARAFIVIRUS 351 -PNTSHPLFQS--PFAR-LSHWLRTHT--WALWCLASP---SASV-----SAWATASALGRL---L--PLHTDR---------------------------------------------- 405

BotrytisF_MYCOFLEXIVIRUS 353 RPVTPLDNFAT-ALWYR-LLPSRLYRL--PATIRNF------Q-L-----KAFVDQLRTPQY---LLHFKLDEYHLSSNDKYSDFYRNESRQASSKDMLSAFEAICFGGAPELEKKILGN 453

DonkeyOrchidDORKYVIRUS 347 -RHPNALAISNASLLGR-LSTHAKDAL--RRALSPFVG---PDGF-----EEHQRQLQLQPF---NYSIKTERYVSDHLP---------------------------------------- 411

AppleStem_CAPILLOVIRUS 369 -MRDDFDV--------S-VLQWVKDKF--CQVMPHFIA---ASFF-----EPTEFHLNMRKL---LNDLATKGIEVPLSV---------------------------------------- 425

SclerotiniaDebilitation 585 -RFNSSEELIGSGFFES-LVSPLKQWF--SEICEKFLG---KSNF-----HKTLEALEWKVI---NYDVKTVIYDMSKPW---------------------------------------- 49

PotatoM_CARLAVIRUS 376 -S---MCATIMPEQLKE-FMGNWLGKM--PSVLARRFSSVRAVCV-----NKFIRGLKPYSF---TLRLNEITWWNIWEN---------------------------------------- 440

AppleStemPitting_FOVEAVIR 377 -T---LNTNMIPDMKRI-VQGFFLKLF--PNPISRNFKVVQQLHL-----DNFIETLEEFNF---SINTESLSLNWKDDL---------------------------------------- 441

CitrusLeaf_CITRIVIRUS 382 -T---SWGLFGHSFFEK-LTDMALSSL--PNSIARIFPQWKKKNT-----FEFLFSLGTLVV---DVERKVCFEHVLEEW---------------------------------------- 446

ApricotVeinClearing 366 -NESECFSLFDVSIVNK-WKKKFLDFA--PDWLLNGFM---TWKS-----GNFIKKLGEHEA---ARQVQEGAYPEGWMI---------------------------------------- 430

GrapevineA_VITIVIRUS 362 -A--LYDKMGNPSLRSI-LSESFYDVA--GNLFTRLFN---RPEYDARCLEKFIRACETTEI---HIGRRFMEGVLRGSS---------------------------------------- 429

PotatoT_TEPOVIRUS 351 -PASPFGVKGVFDLLTS-IFKDIFLLD--GLFNWSDRR--KSEKF-----VEFMRALDYQTNKVVTCTFSGGVMRSGFLA---------------------------------------- 419

AppleChlorotic_TRICHOVIRU 358 -G----VESWSCSFWGC-MKDWFFDKLPYREVLEKIGL---ADDF-----TRRLMKIKPLAF---DIHTTDRPLTVRMI----------------------------------------- 419

OysterMushroom 345 -VDSEELEGITSVGLSR-LRFFIRRKA--EEWLPDWLF---ELLY-----RDFVIARRVRRA---LTQDIVVIKVPL------------------------------------------- 406

Consensus_ss: hhhhh hhhhhhhhh hhhhhhh hhhh hhhhhh ee eeeeeeeee

**........[αI]..... ....αJ....** **[βL’]**

Thickly underlined: regions predicted as membrane-binding, amphiphilic α-helices by Amphipaseek
