# Supplementary material for: Sequence analysis reveals a conserved extension in the capping enzyme of the alphavirus supergroup, and a homologous domain in nodaviruses
Source: Biol Direct. 2015 Apr 11;10:16. doi: 10.1186/s13062-015-0050-0 (PMC4392871; doi:10.1186/s13062-015-0050-0)
Supplement: Additional file 1: — Compilation of all supplementary figures and tables, in .zip format. [file 13062_2015_50_MOESM1_ESM.zip › Additional File 1/Figure S9 selected amphipathic helices predicted by Heliquest.pdf]

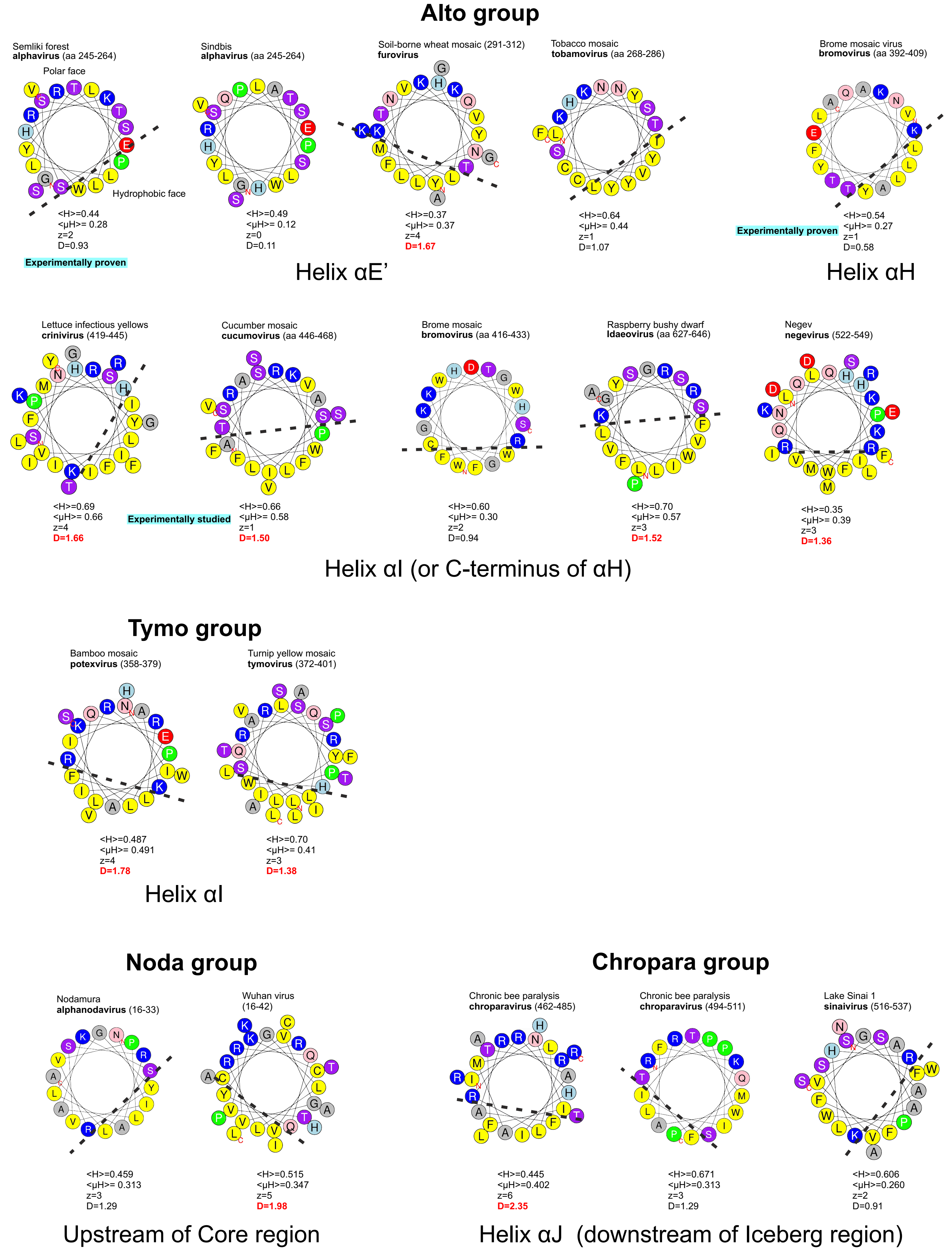
Alto group
Semliki forest
alphavirus (aa 245-264)
Polar face
Hydrophobic face
<H>=0.44
<μH>= 0.28
z=2
D=0.93
Experimentally proven
Sindbis
alphavirus (aa 245-264)
<H>=0.49
<μH>= 0.12
z=0
D=0.11
Soil-borne wheat mosaic (291-312)
furovirus
<H>=0.37
<μH>= 0.37
z=4
D=1.67
Tobacco mosaic
tobamovirus (aa 268-286)
<H>=0.64
<μH>= 0.44
z=1
D=1.07
Brome mosaic virus
bromovirus (aa 392-409)
Experimentally proven
<H>=0.54
<μH>= 0.27
z=1
D=0.58
Helix αE'
Helix αH
Lettuce infectious yellows
crinivirus (419-445)
<H>=0.69
<μH>= 0.66
z=4
D=1.66
Experimentally studied
Cucumber mosaic
cucumovirus (aa 446-468)
<H>=0.66
<μH>= 0.58
z=1
D=1.50
Brome mosaic
bromovirus (aa 416-433)
<H>=0.60
<μH>= 0.30
z=2
D=0.94
Raspberry bushy dwarf
Idaeovirus (aa 627-646)
<H>=0.70
<μH>= 0.57
z=3
D=1.52
Negev
negevirus (522-549)
<H>=0.35
<μH>= 0.39
z=3
D=1.36
Helix αI (or C-terminus of αH)
Tymo group
Bamboo mosaic
potexvirus (358-379)
<H>=0.487
<μH>= 0.491
z=4
D=1.78
Turnip yellow mosaic
tymovirus (372-401)
<H>=0.70
<μH>= 0.41
z=3
D=1.38
Helix αI
Noda group
Nodamura
alphanodavirus (16-33)
<H>=0.459
<μH>= 0.313
z=3
D=1.29
Wuhan virus
(16-42)
<H>=0.515
<μH>=0.347
z=5
D=1.98
Upstream of Core region
Chropara group
Chronic bee paralysis
chroparavirus (462-485)
<H>=0.445
<μH>=0.402
z=6
D=2.35
Chronic bee paralysis
chroparavirus (494-511)
<H>=0.671
<μH>=0.313
z=3
D=1.29
Lake Sinai 1
sinaivirus (516-537)
<H>=0.606
<μH>=0.260
z=2
D=0.91
Helix αJ (downstream of Iceberg region)
