# Supplementary material for: Sequence analysis reveals a conserved extension in the capping enzyme of the alphavirus supergroup, and a homologous domain in nodaviruses
Source: Biol Direct. 2015 Apr 11;10:16. doi: 10.1186/s13062-015-0050-0 (PMC4392871; doi:10.1186/s13062-015-0050-0)
Supplement: Additional file 1: — Compilation of all supplementary figures and tables, in .zip format. [file 13062_2015_50_MOESM1_ESM.zip › Additional File 1/Figure S2 Alto MTase GTase.doc]

**Figure S2. Sequence alignment of the MTase-GTase of the Alto group**

The alignment, made with Promals (see Methods), displays predicted secondary structure elements for each sequence. Conventions are explained in the graphical caption below. We highlighted in yellow and in blue, respectively, conserved positions and experimentally substituted residues mentioned in the text.

```
AVYATGA    α-helix (red)
CINRLEF    β-strand (blue)
IQFGTMP    coil (no regular secondary structure)
hhhhh (below alignment): region consistently predicted as α-helical
eeeee (below alignment): region consistently predicted as β-strand
```

[αZ] : secondary structure element found only in some taxons

```
 	                                             ..Region upstream of MTase-GTase            | N-term extension of the MTase-GTase ..
Conservation:
Sindbis_ALPHAVIRUS                 ------------------------------------------------------------------------------------------------------------------------
Alfalfa_ALFAMOVIRUS            14  ------------------------------MRE-PLSHAS---------------------------------------------------IQEMLRRVVEKQA-ADDT---------TA   41
GrapevineLeafroll3_AMPELO     336  -----------------FLMHLGFRSLKVIRFAGTNILHMPSLNEERTFGWKGGDVYLPNVPKTAIVAGDRTRLGGEILASVANALNQEEVYSSVVSSITNRLV-LRDQ---------SA  428
CitrusTristeza_CLOSTEROVI       4  -------------------------------NSLIITDTD---------------------------------------------------RLRAVGSAYEKVKRNIPD---------SL   32
BeetNecrotic_BENYVIRUS         19  ------------------SVQLLTSDMPIDVQWGFVHSTRCYALWKDD----------------------------------LIHLNPLLKYSQRIAKRWERLV-SGFVGPVPLDKLLSL   85
SclerotiniaL                   28  -------------------PQSRTTRPTRSSRS-ALS-------------------------------------------------------APILRLDHQNAH-RAPD---------HP   62
Helicoverpa_OMEGATETRAVIR          ------------------------------------------------------------------------------------------------------------------------
HepatitisE_HEPEVIRUS               ------------------------------------------------------------------------------------------------------------------------
CuthroatTrout_FISHHEPEVIR       3  -------------------------------------------------------------------------------------------LDCFAH----------------------R    9
PelargoniumZonate_ANULAVI       4  -------------------------------------------------------------------------------------------TSFN-VRDLINSN-GADA---------MG   21
CucumberMosaic_CUCUMOVIRU       4  -------------------------------------------------------------------------------------------SSFN-INELVASH-GDKG---------LL   21
BromeMosaic_BROMOVIRUS          3  -------------------------------------------------------------------------------------------SSID-LLKLIAEK-GADS---------QS   20
CitrusLeprosisC_CILEVIRUS      44  VASSEKPPVSFTIANGQIVSDSEVITSTEAKAAAILSVISSY----------------------------------------------PKEIREQ-LNGRIERG-FTDN---------PD  106
OliveLatent2_OLEAVIRUS          3  ------------------------------------------------------------------------------------------------SAALEKMT-GAKF---------PA   16
JapaneseHolly_PTERIDOVIRU     122  -----------------ADVPQFNLGSLPQVKAVDYLEPGS------------------------------------------------LQFEQVRGSVIRRAV-ADDD---------SN  166
RaspberryBushy_IDAEOVIRUS     119  ---------------------------------CSVFSSD---------------------------------------------------FESKLRDIRDAAL-SDAA---------SG  144
TobaccoStreak_ILARVIRUS        10  ------------------------------CDV-VVPALN---------------------------------------------------VDNLIRDYVSNVR-ADDS---------NN   37
TobaccoMosaic_TOBAMOVIRUS       4  -------------------------------------------------------------------------------------------TQTATTSALLDTV-RGNN---------SL   22
GremmeniellaXL1_ENDORNAVI     114  -----------------IGQEEDAEHIRLHVTMRKGILHFENPIGLPTGDPSPELFAKQREFVK---TYKGETVGLLDILNKHDGNTLVGLSEEPLLDRIAKDA-GISIPQ-----ITTS  207
TobaccoRattle_TOBRAVIRUS_       9  ------------------------------SQL-LNVDEM---------------------------------------------------SAEQRSHFFDLML-TKPD---------CE   36
SoilWheat_FUROVIRUS             8  ----------------------------------ILGIIS---------------------------------------------------EEDVIRAAISTSA-TKFG---------SQ   32
Potato_POMOVIRUS                3  --------------------------------QSILSKIT---------------------------------------------------SDDYVNAILHTSA-TRVG---------SD   29
BarleyStripe_HORDEIVIRUS        4  ------------------------------DEI-VRNLIS---------------------------------------------------REEVMGNLISTAS-SSVR---------SP   31
IndianPeanut_PECLUVIRUS         9  --------------------------------DMIDRYVN---------------------------------------------------DETVVQNLFMTAS-STTK---------SL   35
NudaureliaBeta_BETATETRAV          ------------------------------------------------------------------------------------------------------------------------
LettuceInfections_CRINIVI       8  ----------------------------------REDFRDLD-----------------------------------------------LRVEEFAERVLETAN-LRSD---------NR   36
Rubella_RUBIVIRUS               2  --------------------------------------------------------------------------------------------EKLLDEVLAPG--GPYN---------LT   18
Consensus_ss:                                                                                                                 hhhhhhhhhhhhh               h
```

**[αX]**

```
	                                                              .. N-term extension of the MTase-GTase ..
                                                                                                          23
Sindbis_ALPHAVIRUS              2  ------------------------------------------------------------------------------------------------EKPVVNVDVDPQSPFVVQLQKSFP   25
Alfalfa_ALFAMOVIRUS            42  IGKVFSE--------------------------------------------------------------------------AGRAYAQDALP--SDKGEVLKISFSLDATQQNILRANFP   85
GrapevineLeafroll3_AMPELO     429  LLSHLDT--------------------------------------------------------------------------KLCDMFSQRDAM-IREKPSHRCDVFLKPREREKLRELFP  473
CitrusTristeza_CLOSTEROVI      33  LVRSVEK--------------------------------------------------------------------------DLIEFNQTLVDM-HRSRPGVVVPFQMSENQQVMLTRAYP   77
BeetNecrotic_BENYVIRUS         86  LAKLMRYCVNMGVSVQEIYLSDAIVSSSYMLHVSRSAGCVSFSWLYAKLSMFASCGKFWVGSSHHTAANMIEGSRAVNGPDVAISEMVEAFH--LEVKSSLVVTVSLTPREKKILERELG  203
SclerotiniaL                   63  VNVLIGE--------------------------------------------------------------------------HLRGEVNDIIVA-SRSRWTINVAQPHTEAQVAALEASFP  107
Helicoverpa_OMEGATETRAVIR       3  AKATDVA--------------------------------------------------------------------------RVYAAADVAYAN-VLQQRAVKLDFAPPLKALETLHRLYY   47
HepatitisE_HEPEVIRUS            3  AHQFIKAPG----------------------------------------------------------------------ITTAIEQAALAAAN-SALANAVVVRPFLSHQQIEILINLMQ   51
CuthroatTrout_FISHHEPEVIR      10  DHDKVKL--------------------------------------------------------------------------AISDCIEKSAGA-YVN-TSHVITVVLTPEQLEQAKKAFS   53
PelargoniumZonate_ANULAVI      22  VRGLVDA--------------------------------------------------------------------------HATKAAEEQFEY-IKRSKKVWVRQILSASDGEKMQKRFG   66
CucumberMosaic_CUCUMOVIRU      22  ATALVDK--------------------------------------------------------------------------TAHEQLEEQLQH-QRRGRKVYIRNVLGVKDSEVIRNRYG   66
BromeMosaic_BROMOVIRUS         21  AQDIVDN--------------------------------------------------------------------------QVAQQLSAQIEY-AKRSKKINVRNKLSIEEADAFRDRYG   65
CitrusLeprosisC_CILEVIRUS     107  VDEAARC--------------------------------------------------------------------------IHSARLHNITTT-ALRKKPLLVHENVSNDMERFLNEKFL  151
OliveLatent2_OLEAVIRUS         17  VQGVIEE--------------------------------------------------------------------------FSRDRVQNVLGD-MRSRQVIKYAVGLSEASVQELRFNWP   61
JapaneseHolly_PTERIDOVIRU     167  VSASLRA--------------------------------------------------------------------------DYLEKLRQAELV-RQSLRKVRVVRSISPDQLATLRSLFP  211
RaspberryBushy_IDAEOVIRUS     145  VSQLVSC--------------------------------------------------------------------------HFEKDVRHLLAE-NANSVKIPVPQKLSDDDMRILRDHFP  189
TobaccoStreak_ILARVIRUS        38  VSRFLGE--------------------------------------------------------------------------VALKEIKSQVDTSNGDFQKLNVGFRLTPDEKNALKANFP   83
TobaccoMosaic_TOBAMOVIRUS      23  VNDLAKR--------------------------------------------------------------------------RLYDTAVEEFNA-RDRRPKVNFSKVISEEQTLIATRAYP   67
GremmeniellaXL1_ENDORNAVI     208  QFKTEMD--------------------------------------------------------------------------KLVNTRLQHAAT-NHATKVILLPTSTPVDIVEDLNNLHG  252
TobaccoRattle_TOBRAVIRUS_      37  IGQMMQR--------------------------------------------------------------------------VVVDKVDDMIRE-RKTKDPVIVHEVLSQKEQNKLMEIYP   81
SoilWheat_FUROVIRUS            33  LHSTVCD--------------------------------------------------------------------------HVKETYLDAVER-QKVKKKIDVRRDLSQEQLQLLNDLYP   77
Potato_POMOVIRUS               30  LHNAMCN--------------------------------------------------------------------------VIVDQIKDSTEK-NKKKKKIDVKRNLNEDQIQLLAELFP   74
BarleyStripe_HORDEIVIRUS_      32  LHDVLCS--------------------------------------------------------------------------HVRTIVDSVDKK-AVSRKHEDVRRNISSEELQMLINAYP   76
IndianPeanut_PECLUVIRUS        36  LHDVIAD--------------------------------------------------------------------------RAANHLKSLEKK-QKQKELVDVRRVLSSESLNALCSLYP   80
NudaureliaBeta_BETATETRAV       3  ---DAS----------------------------------------------------------------------------KQLRVLDAQE---RAKAAFQLDFIASVETLEDAQEKYE   40
LettuceInfections_CRINIVI      37  LIDNILT--------------------------------------------------------------------------RASDYINKK----SKESKELDINVCLSMDEKKMITNLFP   78
Rubella_RUBIVIRUS              19  VGSWVRD--------------------------------------------------------------------------HVRSIVEGA----------WEVRDVVTAAQKRAIVAVIP   54
Consensus_ss:                      hhhhhhh                                                                          hhhhhhhhhhhh       eee     hhhhhhhhhh
			 [αY..]							    [..αY...]	   βY	   αZ
 			                    |Start of Core region of the MTase-GTase
  			   		        39			               		         81 83         91 94
Conservation:                                      H♠  5 5  R^                            6  5                   H#Cº         D♥ R♣
Sindbis_ALPHAVIRUS             26  Q-FEVVAQQVTPN--DHANA-RAF-SHLAS-KLIELEVPT----------TATILDIGSAPARRMFSE-------HQYHCVC---PMRSPEDPDRMMKYASKLAEKACKITNKN--LHEK  117
Alfalfa_ALFAMOVIRUS            86  G-RRTVFSNSSSS--SHCFA-AAH-RLLET-DFVYRCFGNT---------VDSIIDLGGNFVSHMKVK------RHNVHCCC---PILDARDGARLTERILSLKSYVRKHPE--------  173
GrapevineLeafroll3_AMPELO     474  E-LSIQFSDSVRS--SHPFA-NAM-RSCFN-GIFSRRCG-----------NVCFFDIGGSFTYHVKAG------HVNCHVCN---PVLDVKDVKRRINEILFLSTAGGDSYVS-------  560
CitrusTristeza_CLOSTEROVI      78  E-FNINFIHSVHS--DHPVA-AGS-RALEN-HLVRKHA------------GTDYSDVGGCPLFHLRAG------HSGVHVCR---PVYDVKDAHRRVVRHHQLSKVSLDQSDG-------  163
BeetNecrotic_BENYVIRUS        204  --FVPLYKQKSRAPRNHPVL-AAL-REVMRQEYSASCNILN--------TKLKTLVVGAASREVNCYSS-----NPSVHYYF---ANKDSKDLVRTTLELLHSALATKYRN---------  294
SclerotiniaL                  108  E-YNFVSSYAHAH-- HPSTQ-AA R-HALEL-LTWKLVD----------PAHTTEIGPSYSSLFRRAR-----PAFAHYDM---PLLTVRDGARRNLAMLSRHKTLDERETR-------  194
Helicoverpa_OMEGATETRAVIR      48  P-LRFKGGTLPPT--QHPIL-AGHQRVAEE-VLHN-FARG----------RSTVLEIGPSLHSALKLHGAPNAPVADYHGC----TKYGTRDGSRHITALESRSVATGRPEFKA------  141
HepatitisE_HEPEVIRUS           52  P-RQLVFRPEVFW--NHPIQ-----RVIHN-ELELYCRAR----------SGRCLEIGAHPRSINDNP-------NVVHRCF---LRPAGRDVQRWYTAPTRGPAANCR-----------  131
CuthroatTrout_FISHHEPEVIR      54  P-QPVTYQPRVCL--PHALAKHYT-TVLEG-QLKNAIR------------GRPYIEAGPAP-HQINSR------PKDTHFCL---VRGDARNHVRFATAPNNGLSRKV------------  134
PelargoniumZonate_ANULAVI      67  G-TFDLQLSKNLC--PHSFA-GAM-RQCET-LECLSSFPE----------DSLILDFGGSWLFHWQRQ-------HNVHSCC---PVLDARDMARHQERMISMQKCVAHRPGKF------  154
CucumberMosaic_CUCUMOVIRU      67  GKYDLHLTQQEFA--PHGLA-GAL-RLCET-LDCLDSFPSSG------LRQDLVLDFGGSWVTHYLRG-------HNVHCCS---PCLGIRDKMRHAERLMNMRKIILNDPQQF------  159
BromeMosaic_BROMOVIRUS         66  GAFDLNLTQQYHA--PHSLA-GAL-RVAEH-YDCLDSFPP----------EDPVIDFGGSWWHHFSRR------DKRVHSCC---PVLGVRDAARHEERMCRMRKILQESDDF-------  154
CitrusLeprosisC_CILEVIRUS     152  G-YKIRLTFSKNV--AHNNA-AAL-RRVLR-FYMRDKVGYRKDDDIPDGYHVKNKDVGASGMDVIADE------LTDVHCCT---PDLDFRDHIRLERLKKYIYSHVCPTSKDHGII---  253
OliveLatent2_OLEAVIRUS         62  C-FTWEEESVPLP--PHPFA-AYS-RRAFT-RWAIAQCG-----------PVPIKDFGGNWFLHWQWQ-------TGVHSCC---PLLNPRDGAHQTRRELNMESYLRTHGPK-------  147
JapaneseHolly_PTERIDOVIRU     212  G-FEFEYTPRYDG--PHNMA-AVI-RTAET-LEAVLSFPK----------DEPLIDIGGDFFFHVRNG------FTHVHCCA---PILDSRDDERFSKRCMHLQKWVIDKQVSAYSSMSR  306
RaspberryBushy_IDAEOVIRUS     190  R-YELKFTQNVDG--PHNMA-AAH-RLLET-HDLLSNFPA----------DAPILDIGGNWFSHFRYG------RSNVHSCC---PMLDLRDNERHTHRLTMTESLMSSLRHRYAGTIDL  284
TobaccoStreak_ILARVIRUS        84  G-LEIAFRDSCHS--SHSFA-AAH-RVCET-LNIYNRFKTK---------TERIIDLGGNYVTHAKQG------RSNVHSCC---PILDVRDGARHTDRYISLAASVENRHR--------  171
TobaccoMosaic_TOBAMOVIRUS      68  E-FQITFYNTQNA--VHSLA-GGL-RSLEL-EYLMMQIPY----------GSLTYDIGGNFASHLFKG------RAYVHCCM---PNLDVRDIMRHEGQKDSIELYLSRLERGGKTVPNF  162
GremmeniellaXL1_ENDORNAVI     253  S-GSFVRGNMAAH--SHIYH-HAN-RLMLT-EMLASRHPR----------NALLYDIGGNINRHLDHG------RLNVHCVY---TTSTPADLSRHTLWVKKATQWAHANLKNKSVHGAT  347
TobaccoRattle_TOBRAVIRUS_      82  E-FNIVFKDDKNM--VHGFA-AAE-RKLQA-LLLLDRVPA----------LQEVDDIGGQWSFWVTRG------EKRIHSCC---PNLDIRDDQREISRQIFLTAIGDQARSGKRQMSEN  176
SoilWheat_FUROVIRUS            78  E-RHIVSSNCERG--THSFA-AAS-RKIET-DLLLSRIPK----------RSWVYDIGGNWATHVKRND-----GRKVHCCC---PTVDIRDSARKTVRWASIEKYLDEKEEIPPEIGER  173
Potato_POMOVIRUS               75  E-RRVVTSSVHRG--THSMA-AAM-RKIET-DVIFTSFPK----------NGVIYDIGGNWATHAKRDD-----GGFVHCCC---PILDFRDAQRKMTRLIDFNRFIDDAKVVSAEKAAV  170
BarleyStripe_HORDEIVIRUS_      77  E-YAVSSSACESG--THSMA-ACF-RFLET-EYLLDMVPMK---------ETFVYDIGGNWFSHMKFRA-----DREIHCCC---PILSMRDSERLETRMMAMQKYMRGSKDKPLRLLSR  173
IndianPeanut_PECLUVIRUS        81  E-FNVITSNRERG--SHTMA-AVC-RTLET-LYIKSLLPVN---------DTVVWDVGGNWLTHVKYST-----NQDVHCCC---PLLDYRDAMRKQERILSLELFARNGKEKTEEFDAC  177
NudaureliaBeta_BETATETRAV      41  G-MMFRSGTKLPS--THIKL-AID-LRVAE-KDLRRHVKN----------VPTVLEIGPSVESVRYAVQT--RDKERVHGC----TFSDARDNLRHNKIGYEAHYDRKIGPD--------  130
LettuceInfections_CRINIVI      79  D-IQMSFNQKSYS--NHGVF-NAM-RACEN-FYFSRKFK-----------NSDYIDAGGDVVSTLRSK------NHNVHICS---PRLDLKDAARHIQRATVIDGLKGYG----------  162
Rubella_RUBIVIRUS              55  ---RPVFTQMQVS--DHPALHAIS-RYTRR-HWIE---------------------WGPK---------------EALHVLIDPSPGL-LREVARVERRWVALCLHRTARKL--------  122
Consensus_ss:                         eeeee         hhh hhh hhhhh hhhhhh                eeee   hhhhhh           eeee           hhhhhhhhhhhhhhhh
			    βZ	     ....αA......	 	 βA   αB	  βB  	         αC       [αC’..]
                                     Underlined: accessible epitope in equivalent position of Beet Yellows closterovirus
									..Core region..
Conservation:
Sindbis_ALPHAVIRUS            118  IKD---------------------------------------------------------------------------------------------------------------------  120
Alfalfa_ALFAMOVIRUS                ------------------------------------------------------------------------------------------------------------------------
GrapevineLeafroll3_AMPELO          ------------------------------------------------------------------------------------------------------------------------
CitrusTristeza_CLOSTEROVI          ------------------------------------------------------------------------------------------------------------------------
BeetNecrotic_BENYVIRUS             ------------------------------------------------------------------------------------------------------------------------
SclerotiniaL                       ------------------------------------------------------------------------------------------------------------------------
Helicoverpa_OMEGATETRAVIR          ------------------------------------------------------------------------------------------------------------------------
HepatitisE_HEPEVIRUS               ------------------------------------------------------------------------------------------------------------------------
CuthroatTrout_FISHHEPEVIR          ------------------------------------------------------------------------------------------------------------------------
PelargoniumZonate_ANULAVI          ------------------------------------------------------------------------------------------------------------------------
CucumberMosaic_CUCUMOVIRU          ------------------------------------------------------------------------------------------------------------------------
BromeMosaic_BROMOVIRUS             ------------------------------------------------------------------------------------------------------------------------
CitrusLeprosisC_CILEVIRUS          ------------------------------------------------------------------------------------------------------------------------
OliveLatent2_OLEAVIRUS             ------------------------------------------------------------------------------------------------------------------------
JapaneseHolly_PTERIDOVIRU     307  RVKATLKEIPNLELLCDRLREFNDACRTPDDNGMDCSMFCHRTFQSSFETVEDSSSVPFEDAVVSGSFAVSPWDVETQVGRRLAGGKFPPKKTFDDLADVAVNWVTEGVNSVVSSLKKER  426
RaspberryBushy_IDAEOVIRUS     285  DPDAHLSRKVSDSMKE--------------------------------------------------------------------------------------------------------  300
TobaccoStreak_ILARVIRUS            ------------------------------------------------------------------------------------------------------------------------
TobaccoMosaic_TOBAMOVIRUS     163  Q-----------------------------------------------------------------------------------------------------------------------  163
GremmeniellaXL1_ENDORNAVI     348  Y-----------------------------------------------------------------------------------------------------------------------  348
TobaccoRattle_TOBRAVIRUS_     177  ELWM--------------------------------------------------------------------------------------------------------------------  180
SoilWheat_FUROVIRUS           174  IKRLQADED---------------------------------------------------------------------------------------------------------------  182
Potato_POMOVIRUS              171  AAQIKKDCD---------------------------------------------------------------------------------------------------------------  179
BarleyStripe_HORDEIVIRUS_     174  YQNILREQA---------------------------------------------------------------------------------------------------------------  182
IndianPeanut_PECLUVIRUS       178  YQQIRNFE----------------------------------------------------------------------------------------------------------------  185
NudaureliaBeta_BETATETRAV          ------------------------------------------------------------------------------------------------------------------------
LettuceInfections_CRINIVI          ------------------------------------------------------------------------------------------------------------------------
Rubella_RUBIVIRUS                  ------------------------------------------------------------------------------------------------------------------------
Consensus_ss:                    [..αC’..]
								..core region..

                                                                 136                         143
Conservation:                                                    C*                          C*
Sindbis_ALPHAVIRUS            121  ---------------LRTVLDTPDAETPSLCFHN---------------------DVTCNMRAE--------------------------------------------------------  148
Alfalfa_ALFAMOVIRUS           174  -----------------------IVGEADYCMDT---------------------FQKCSRRAD--------------------------------------------------------  193
GrapevineLeafroll3_AMPELO     561  ----------------SDLLTEAASKSVSYCSRE---------------------SQNCDSRA---------------------------------------------------------  586
CitrusTristeza_CLOSTEROVI     164  -----------------VKQVGWTVNTNSVCGNI---------------------LGECYHAS---------------------------------------------------------  188
BeetNecrotic_BENYVIRUS        295  -----------------------MESGERELMNN---------------------LKGCGYIVKRSVENAVYEVVSDKDVAEVLRYAQTVASTKKEAKKKPNTGKRKMVMSEATRRTIEL  370
SclerotiniaL                  195  ----------------ALDALRTGMPSGEFCPLS---------------------GEDCSHKSS--------------------------------------------------------  221
Helicoverpa_OMEGATETRAVIR     142  ----------------DASLLANGIASRTFCVDG---------------------VGSCAFKSR--------------------------------------------------------  168
HepatitisE_HEPEVIRUS          132  ----------------RSALRGLPAADRTYCFDG---------------------FSGCNFPAE--------------------------------------------------------  158
CuthroatTrout_FISHHEPEVIR     135  ----------------KRIYQGIQKEDATACLNG---------------------YQGCHQPRA--------------------------------------------------------  161
PelargoniumZonate_ANULAVI     155  ----------------------ESFESPDFCLLK---------------------AEDCEVQSP--------------------------------------------------------  175
CucumberMosaic_CUCUMOVIRU     160  -----------------------DGRQPDFCTQP---------------------AADCKVQAH--------------------------------------------------------  179
BromeMosaic_BROMOVIRUS        155  ------------------------DEVPNFCLNR---------------------AQDCDVQAD--------------------------------------------------------  173
CitrusLeprosisC_CILEVIRUS     254  ------------------CEGFREGSTKYRCENI---------------------GQQCYIEAP--------------------------------------------------------  278
OliveLatent2_OLEAVIRUS        148  ------------------YDKFNELSRPDLCHHR---------------------AEDCSVRAK--------------------------------------------------------  172
JapaneseHolly_PTERIDOVIRU     427  SGDISCDSSCVEEGAPEGVSVASASVASDESVRR---------------------TVSCGRRAR--------------------------------------------------------  469
RaspberryBushy_IDAEOVIRUS     301  ---FYKRWAVHPKDLIRLYAGIRDGNSSLYCHHKFGVSWNDVLWESERNNCLTVPEPECPFKAK--------------------------------------------------------  361
TobaccoStreak_ILARVIRUS       172  ------------------------ELPVDFCCHK---------------------FEECDVKAP--------------------------------------------------------  190
TobaccoMosaic_TOBAMOVIRUS     164  ---------------KEAFDRYAEIPEDAVCHNT---------------------FQTMRHQPM--------------------------------------------------------  191
GremmeniellaXL1_ENDORNAVI     349  ---------------SSIAASMIKEDNSMWCTNG---------------------IANCSHAPISV------------------------------------------------------  378
TobaccoRattle_TOBRAVIRUS_     181  ---------------YDQFRKNIAAPNAVRCNNT---------------------YHGCTCRGFSD------------------------------------------------------  210
SoilWheat_FUROVIRUS           183  ---------RIYANLKSEKAQPEDLDGKWYCGNR---------------------FEDCVFRADR-------------------------------------------------------  217
Potato_POMOVIRUS              180  ---------LISENAKKDAYDANDLNGTWFCQNK---------------------FEDCVYDHSTL------------------------------------------------------  215
BarleyStripe_HORDEIVIRUS_     183  ---------ARTTAFMAGEVNAGVLDGDVFCENT---------------------FQDCVRRVPE-------------------------------------------------------  217
IndianPeanut_PECLUVIRUS       186  -----------NTRRTAISAGKNVVMNDRYCCDV---------------------FQNCAYEPDS-------------------------------------------------------  218
NudaureliaBeta_BETATETRAV     131  -----------------AALLAAGIPTDTFCVDG---------------------FSNCEYQSP--------------------------------------------------------  156
LettuceInfections_CRINIVI     163  -------------------------ETISFCTNK---------------------TEDCAVNRD--------------------------------------------------------  180
Rubella_RUBIVIRUS             123  -----------------ATALA---ETASEAWHA---------------------DYVCALRGAPS-n----------------------------------------------------- 147
Consensus_ss:                                      hhhh         ee                            eee
```

[..αC’]	**βB’**	**βB’’**

```
								..core region..

                                                                           158
Conservation:                                                             5D/N                                                  5
Sindbis_ALPHAVIRUS            149  -------------------------------YSVMQDVYIN------APGTIYHQAMKGVRTLYWIGFDTTQFMFSAMA--------------GSYPAYNTNWADEKVLEARNIGLCSTK  217
Alfalfa_ALFAMOVIRUS           194  -------------------------------YAFAIHSTSDL----DVGELACSLDQKGVMKFICTMMVDADMLIHNE---------------GEIPNFNVRW-----------------  246
GrapevineLeafroll3_AMPELO     587  -------------------------------DAGFMVDVYDI----SPQQVAEAMDKKGALVFDIALMFPVELLYGNGE--------------VYLEELDTLV-----------------  640
CitrusTristeza_CLOSTEROVI     189  -------------------------------EAMVMVQVYDV----PLRELCRAMINKKTSVCYMTMVTPGELLDARES--------------FFIKDLDCSV-----------------  242
BeetNecrotic_BENYVIRUS        371  HELSRIVAEEKKIPNHFHFDESDFASVGNFTQLVCEDVGYNF----SVDAWLHLFEATGAQTAVGYMALPNELLFEHYPISDYYDYWEGVEKHGSLGGITISP-----------------  469
SclerotiniaL                  222  -------------------------------ALVSFFSLHDA----SPAVLDAMMARKDALVAYVAMHLPVALLATSS---------------FDDVATGAHC-----------------  274
Helicoverpa_OMEGATETRAVIR     169  -------------------------------VGIANHSLYDV----TLEELANAFENHGLHMVRAFMHMPEELLYMDN---------------VVNAELGYRF-----------------  221
HepatitisE_HEPEVIRUS          159  -------------------------------TGVALYSLHDM----SPSDVAEAMFRHGMTRLYAALHLPPEVLLPP----------------GTYRTASYLL-----------------  210
CuthroatTrout_FISHHEPEVIR     162  -------------------------------TSIAIHSTYDI----PLKEFCRGMNNHKSDLAHVIMHMPSEITFPPGV--------------FVNDMTGYRL-----------------  215
PelargoniumZonate_ANULAVI     176  -------------------------------YAISIHGAYDM----GFEGLCKAMHSHGTIMLRGTMMFDANMLVFNE---------------GVMEDLNCRW-----------------  228
CucumberMosaic_CUCUMOVIRU     180  -------------------------------FAISIHGGYDM----GFRGLCEAMNAHGTTILKGTMMFDGAMMFDDQ---------------GVIPELNCQW-----------------  232
BromeMosaic_BROMOVIRUS        174  -------------------------------WAICIHGGYDM----GFQGLCDAMHSHGVRVLRGTVMFDGAMLFDRE---------------GFLPLLKCHW-----------------  226
CitrusLeprosisC_CILEVIRUS     279  -------------------------------TLTFVHSAYDI----TAAGIVDCMIAANAHHAIMCLHFPSAILSGSTS--------------GKDDLLQYKW-----------------  332
OliveLatent2_OLEAVIRUS        173  -------------------------------AALSVDSAYDM----GLKNTCKAMHRAGIELLHGNILFDPDMLIEAKM-------------EGFVAGMNYHW-----------------  227
JapaneseHolly_PTERIDOVIRU     470  -------------------------------FGIMIHSSYDM----LPVPVCQGMVEHDMTMVKGCMIAEPAMLVQTK---------------GYLPILECNW-----------------  522
RaspberryBushy_IDAEOVIRUS     362  -------------------------------YAIMVHSGYDL----PLKELIGGMVQHGVVELHGTMIADPAMLVATS---------------GYIPALRCNW-----------------  414
TobaccoStreak_ILARVIRUS       191  -------------------------------FAMAIHSISDI----PISTVATHCVRRGVRKLIASVMMDPLMMLYDK---------------GHIPLLNVDW-----------------  243
TobaccoMosaic_TOBAMOVIRUS     192  --------------QQSGR-----------VYAIALHSIYDI----PADEFGAALLRKNVHTCYAAFHFSENLLLEDSY--------------VNLDEINACF-----------------  251
GremmeniellaXL1_ENDORNAVI     379  ------------------------------GFAMSIDTLVHL----DPSDLIKFYIDNNVAHASHAMTIPDNYAYATK---------------GVLRHNEGHW-----------------  432
TobaccoRattle_TOBRAVIRUS_     211  -------------GKKKGA-----------QYAIALHSLYDF----KLKDLMATMVEKKTKVGHAAMLFAPESMLVDE---------------GPLPSVDGYY-----------------  270
SoilWheat_FUROVIRUS           218  ------------------------------AYAMAIHSIYDI----DLSDLANALEEKRIKVMSGTFLFSVDLLLGKKS--------------GTLPTMDGFF-----------------  272
Potato_POMOVIRUS              216  -------------GDGKKE-----------AYGMAIHSIYDI----HLVDLVSAMERKKVRVLKGTFLFSADIIIGKKR--------------GELPSVNGFY-----------------  276
BarleyStripe_HORDEIVIRUS_     218  ----------------GFL-----------KTAIAVHSIYDI----KVEEFASALKRKGITQAYGCFLFPPAVLIGQKE--------------GILPSVDGHY-----------------  275
IndianPeanut_PECLUVIRUS       219  ----------------GKL-----------RVAMGVHSVYDM----TLQELVDGLERKKIEHFIGCFLFSPKLLLGQEE--------------GELPFVNGRF-----------------  276
NudaureliaBeta_BETATETRAV     157  -------------------------------LAIACHSLYPDGESNSIMDVAKGMALHGTHVIYAWMHLPVELLTLTDADNIF------EGYSIRFEETGALP-----------------  222
LettuceInfections_CRINIVI     181  -------------------------------IIIAVE-VYDM----TLRDMAKAMLSHGSRKFEFSCIIPPEIFTKECN--------------VELYEGRLKV-----------------  233
Rubella_RUBIVIRUS             148  -------------GPFYVHPED----VPHGGRAVADRCLLYY----TPMQMCELMRTIDATLLVAVDLWPVALAAHVG---------------DDWDDLGIAW-----------------  214
Consensus_ss:                                                     eeeeeeeee       hhhhhhhhhh   eeeeeeee  hhhh                          eee
```

**βC	αD	βD	αE**	variable	**βE..**

```
				...core region...				  |Start of the Iceberg region
									         249        259	      278
Conservation:                                                                             amphipathic helix Y♦H      W/Y/F	      D/E
Sindbis_ALPHAVIRUS            218  LSEGRTGKLSIMRKKELKPGS--------------------------------------RVYF-SVGS---TLYPEHRASL-QSWHLPSVFHLNGKQSYTCRCD----------------  278
Alfalfa_ALFAMOVIRUS           247  ----------EIDRKKD------------------------------------------LIHF-DFIDEPNLGYSHRFSLL-KHYLTYNAVDLGH---AAYRIE----------------  293
GrapevineLeafroll3_AMPELO     641  ----------KREGD--------------------------------------------YLAY-NVGQ-CGEMYEHSFSNV-SGFFTFSYVRTSSG--NVFKLE----------------  685
CitrusTristeza_CLOSTEROVI     243  ----------ELDPIAD------------------------------------------RVVY-CFNN---SAYTHTYSTI-CECMRTPCLVV-DG--FLFTIE----------------  286
BeetNecrotic_BENYVIRUS        470  ----------LRNGQVVGMPTGVFQPVHFDKTSA------------GLGIPGSKMGAAERVIC-HMSDGLGNGYNHVKSDW-QTLLKHPILSSSK---YNFAVEV--------------D  548
SclerotiniaL                  275  ----------RRDD----DTG--------------------------------------HWHM-SFPGSGAAGYTHSLASL-SAWSAGWK-----G--LNLTCE----------------  317
Helicoverpa_OMEGATETRAVIR     222  ----------HVIEEPMAVKDCAFQGGDLRLHFPELDFINESQERRIERLAARGSYSRRAVIF-SGDDDWGDAYLHDFHTW-LAYLLVRNYPTPFG--FSLHIE----------------  311
HepatitisE_HEPEVIRUS          211  ----------IHDGR--------------------------------------------RVVV-TYEGDTSAGYNHDVSNL-RSWIRTTKVTGD----HPLVIE----------------  254
CuthroatTrout_FISHHEPEVIR     216  ----------DTTGE--------------------------------------------TAKF-TYLEDTSNGYDHSKADW-LEWTTVTALIV-DG--FTFHVE----------------  260
PelargoniumZonate_ANULAVI     229  ----------TKEKGDPYGLRGAP--------------------------------CEDMVHF-DFIDESTLSYSHSWKNI-KSFLTEGGYQIGN---VQYVLE----------------  285
CucumberMosaic_CUCUMOVIRU     233  ----------RKIRSAFSETEDVTPLVGKLNS----------------TVFSRVRKFKTMVAF-DFINESTMSYVHDWENI-KSFLTDQTYSY-RG--MTYGIE----------------  305
BromeMosaic_BROMOVIRUS        227  ----------QRDGSGADE----------------------------------------VIKF-DFENESTLSYIHGWQDL-GSFFTESVHCI-DG--TTYLLE----------------  275
CitrusLeprosisC_CILEVIRUS     333  ----------DIVVEDGIK----------------------------------------YYKQ-KFLNDTQASYVHRLDVY-LDKFLTKVVLGSDK--RFYMFE----------------  382
OliveLatent2_OLEAVIRUS        228  ----------KKTRRST------------------------------------------GLMS-SFLEMGSSLFGVSNSSK-EGEGHH-----------DLKIK----------------  266
JapaneseHolly_PTERIDOVIRU     523  ----------RLVTPLGVPLTCHPCKLGDRFYRLS---------TTGDRPLSCPGFDDVFIEY-HFQDDSTMAYRHKFSTV-FEWMTCHIVEC-DG--ARFCLE----------------  602
RaspberryBushy_IDAEOVIRUS     415  ----------EKSKG--------------------------------------------QIWF-SFRDDSTMGYRHDWEVY-SKYLTSTVVSCGK---HFYVME----------------  459
TobaccoStreak_ILARVIRUS       244  ----------EKEDVEETGKT--------------------------------------LIHF-HFVDAPGLSYSHDFDTL-SQYMITNQVIVNNS--YSFRVE----------------  295
TobaccoMosaic_TOBAMOVIRUS     252  ----------SRDGD--------------------------------------------KLTF-SFASESTLNYCHSYSNI-LKYVCKTYFPASNR--EVYMKE----------------  297
GremmeniellaXL1_ENDORNAVI     433  ----------YRTNN--------------------------------------------GTWV-IEFVGESLSYEQKVSYT-DAYLQTLIFTAGE---MAVYNK----------------  477
TobaccoRattle_TOBRAVIRUS_     271  ----------MKKNG--------------------------------------------KIYF-GFEKDPSFSYIHDWEEY-KKYLLGKPVSY-QG--NVFYFE----------------  315
SoilWheat_FUROVIRUS           273  ----------EVEDG--------------------------------------------YVKY-GFHNDTNPGYKHNLNQL-MKYLTKTFVVAKGG--TIYYLE----------------  318
Potato_POMOVIRUS              277  ----------IIDGE--------------------------------------------SIKY-SFYDDPNCGYEHNLNSL-MLYVTKTFVKAAGG--AVYYLE----------------  322
BarleyStripe_HORDEIVIRUS_     276  ----------LVENG--------------------------------------------RIKF-FFANDPNAGYSHDLKDY-LKYVEKTYVDIED---GVFAIE----------------  320
IndianPeanut_PECLUVIRUS       277  ----------KVKKG--------------------------------------------KIRF-FFLDDTTHGYEHDLNDY-LEYIRKSFVVAKCG--HVYMLE----------------  322
NudaureliaBeta_BETATETRAV     223  ----------CTKRR--------------------------------------------KAIF-SGYNDFGSAYVHDAHHW-AGWLKHRGVDTPYG--FSILID----------------  268
LettuceInfections_CRINIVI     234  ----------TRIGD--------------------------------------------NVE--YYYGSNGETFSHSCQTL-KDILSVQVFQF-GG--RVFKKT----------------  277
Rubella_RUBIVIRUS             215  ----------HLDH---DGGC--------------------------------------PADCRGAGAGPTPGYTRPCTTRIYQVLPDTAHP---G--RLYRCGPRLWTRDCAVAELSWE  278
Consensus_ss:                                eee                                              eeee ee        ee hhhh hhhh   eee      eeeeee
```

**..βE	βF** Loop_F-G_ **βG αE’	βH	βI..**

Loop_F-G_ is negatively charged in most taxa

```
Conservation:                            s   h h                                                302             h 317                              s   h
Sindbis_ALPHAVIRUS            279  TVVSCEGYVVKKITISPGITG---------------------------------------ETVGYAVTHNSEG---FLLCKVTDTVK-------------GERVSFPVCTYIPATICDQM  343
Alfalfa_ALFAMOVIRUS           294  RKQDFGGVMVIDLTYSLGFVPKMP-----------------------------------HSNGRSCAWYNRVKG--QMVVHTVNEGY---YHHS------YQTAVRRK-VLVDKKVLTRV  366
GrapevineLeafroll3_AMPELO     686  YEGYRCGYHHLTMCRAQKSPG---------------------------------------TEVTYRSLVPSFVG--KSLVFIPVVA--------------GSSVSFKT-IVLDSDFVDRI  749
CitrusTristeza_CLOSTEROVI     287  MVSLRCSVNYYCITKSSVCPRI--------------------------------------SETKRLRYRRCDSD--LIRIKIPRYSS---KTR-------SCLPGCYY-LYLDAKFVSRV  355
BeetNecrotic_BENYVIRUS        549  LTGRYGCLATFRLTRVTGVKYV-----------------------------------------ARTIKLRPED----RYVRVLDLLHIVRSIRLKG--HAGLKEPYQY-FPVYKREVDTP  620
SclerotiniaL                  318  IVRQFGTIVHMRVVRTFGPSTE-----------------------------------------NTPISIGNL-----LSSLVVIP-----PFK-------STAHESEA-FTTDAGKYGAL  378
Helicoverpa_OMEGATETRAVIR     312  VQRRHGSSIELRITRAPPGDRM-----------------------------------------LAVVPRTSQ-----GLCRIPNIFY---YAD-------ASGTEHKT-ILTSQHKVNML  374
HepatitisE_HEPEVIRUS          255  RVRAIGCHFVLLLTAAPEPSPTPYV----------------------------------PYPRSTEVYVRSIFGPGGTPSLFPT----------------SCSTKSTF-HAVPAHIWDRL  323
CuthroatTrout_FISHHEPEVIR     261  VQRRLGSVVYLIIYRSTLPVKI----------------------------------------TRPLDPLCST-----SFVFDP-------------------YGPTSG-HTAPTSYVRSL  315
PelargoniumZonate_ANULAVI     286  RCVISYGIMSFKIFAVSGKIP--------------------------------------HTRLRHCVWFPKVRD--YVNINPSD------------------PRIWSK-VRVKLDTVREV  346
CucumberMosaic_CUCUMOVIRU     306  RCVIHAGIMTYKIIGVPGMCP--------------------------------------PELIRHCIWFPSIKD--YVGLKIPAS---------------QDLVEWKT-VRILTSTLRET  369
BromeMosaic_BROMOVIRUS        276  REMLKCNIMTYKIIATNLRCP--------------------------------------RETLRHCVWFEDISK--YVGVSIPED---------------WSLNRWKC-VRVAKTTVREV  339
CitrusLeprosisC_CILEVIRUS     383  ITEMFGSVAIIEVFRREKDFIA-------------------------------------GSKLTFNIPRTEHPN--TVTIHTWEYVTGYESLFKGRNGLKSGVMRPVS-IEVPEEFFLSV  462
OliveLatent2_OLEAVIRUS        267  MGSSSTHMVPADWEISYHFRDDCV-----------------------------------LGYTHNLADVLSIAT--GSYVKVG---------------------NTFY-ELERTGLKSGM  327
JapaneseHolly_PTERIDOVIRU     603  RDKFRDGSLYFTITRLRFPSLR-------------------------------------SELNFSCAWLSSMAS--HYVLKVPMFRS---FRYKG----FLRHSYFDE-YVVSKTLVDTV  675
RaspberryBushy_IDAEOVIRUS     460  RDKYRHGVLFYSIIKCSGSLRKG------------------------------------DHTFFHNAWFHEMYD--KYIMKVPLVKV---KDLTGD--EGSVECSWRE-VVMSRKLVDRV  535
TobaccoStreak_ILARVIRUS       296  RTACLSGVYIVEMTLSMTDGHSLAY--------------------------------LKPMRDVSCAWLSSLRK--KVFVKLAVPIS---AEWY------TEQFEVRH-ALMDESLVRYV  371
TobaccoMosaic_TOBAMOVIRUS     298  FLVTRVNTWFCKFSRIDTFLLYKGVAHKSVDSEQFYTAMEDAWHYKKTLAMCNSERILLEDSSSVNYWFPKMRD--MVIVPLFDISL------------ETSKRTRKE-VLVSKDFVFTV  402
GremmeniellaXL1_ENDORNAVI     478  VVGRKGPHMLFEHFLITRAKLE-------------------------------------SECIKHVTWFNMNMD--EIFVMIPEIDFDSAVTLMK-----REPFTMKT-VSINLRFYERL  552
TobaccoRattle_TOBRAVIRUS_     316  PWQVRGDTMLFSIYRIAGVPRRSLS----------------------------------SQEYYRRIYISRWEN--MVVVPIFDLVE------------STRELVKKD-LFVEKQFMDKC  386
SoilWheat_FUROVIRUS           319  LTEQRGDVMFFTMTDATEARMNGVV----------------------------------ADESFKCIPIDNKD---EVVFPIFEVDQ------------KTDALVFSE-ILLSRDFVQRA  388
Potato_POMOVIRUS              323  LTEMRGDTMFFTITDASEARVMGVL----------------------------------HDSSTKCLPLNKRD---LVVFPLFDIDR------------ATDELVFRE-ELLSREFVNRA  392
BarleyStripe_HORDEIVIRUS_     321  LMQMRGDTMFFKITDVTAAMYHM-KYR-----------------------------GMKRDETFKCIPLLKNS---SVVVPLFSWD--------------NRSLKITS-GLLPRTLVEQG  392
IndianPeanut_PECLUVIRUS       323  LFSMRGDTVFFKLYDVTEYSLSKTGFLST--------------------------LLPTRDHVFKAMPIPNTD---EVIVPLYWIE--------------NGEVVVER-RYLPKSLVCRG  398
NudaureliaBeta_BETATETRAV     269  IQQRFGMHTKLKITRGHSSGS-----------------------------------------ITTVFPLSKLG---LIWVPNIVKIM-----------YPKAKHEPEY-IVTDKKKYEGV  332
LettuceInfections_CRINIVI     278  LEHSRGQLHFFSICICEKIEPG---------------------------------------SVKLKTYYQRSEL-DKVTLRIPVKDS-------------FGVVTHYI-IKEDREFVSSM  343
Rubella_RUBIVIRUS             279  VAQHCGHQARVRAVRCTLPIRH----------------------------------------VRSLQP--------SARVRLPDLVH------------LAEVGRWRW-FSLPRPVFQRM  337
Consensus_ss:                      eeeee  eeeeeeee                                                eeeee        eeeeeee                   eeeeee eee hhhhhhh
			..βI   βJ					βK	   βL		    ..βL’..  αF..
						.. Iceberg region ..
                                				           371                                                     palmitoylation
End of sequence similarity with Tymo group>                                  G                            < + charge>                   420
Sindbis_ALPHAVIRUS            344  TGIMAT---------------DISPDDAQKLLVGLNQRIVINGRTN-----RNTNTMQNYLLPIIAQGFSKWAKERKDDLDNEKMLGTRERK------LTYGCLW---------------  422
Alfalfa_ALFAMOVIRUS           367  TEVAFRQFRPN-------ADAHSAIQSIATMLSSSTNHTIIGGVTL-----ISGKPLSPDDYIPVATTIYYRVKKLYNAIPEMLSLLDKGERL------STDAV--LKGSEGP---MWYS  463
GrapevineLeafroll3_AMPELO     750  YSYALNTIG---------TFENRTFEYAVGAVRSQKTHVITGSRVV-----HSKVDISPDDMWGLVVAVMAQAIKDRAKSIRSYNFIKASEGSL---AGVFKLF--FQTVGDC---FSNA  847
CitrusTristeza_CLOSTEROVI     356  YEYVVNNCV---------VVNAKTFEWTWNYIKSCKSRVVISGKII-----HRDEPIALEYLDGFSAVMLSAGVKGRQNADVSRRLAAFSGET-----SIFELV--GFAISEK---CRDL  451
BeetNecrotic_BENYVIRUS        621  VSYCFSIA-----------EKSLTVQNIANFIRHH-----IGGVSLVNKELVSAWRLNPQLVPSFAYAVYFYVVNLRGELDGMLQKLMKKGIT------WADRL--KANVSAF---LRDM  713
SclerotiniaL                  379  VDRLVLED-----------LTDAPVKKALARIAALSPAIRVGSRVQ-----SAAWDIPYLKRNPVAVAAVRAALVLSADIEFCENLAANDI---------API------RAAL---VERL  464
Helicoverpa_OMEGATETRAVIR     375  LNFMQTRP----------EKELVDMTVLMSFARARLRAIVVASEVT-----ESSWNISPADLVRTVVSLYVLHIIERRRAAVAVKTAKDDVFG-------ETSF--WESLKHV---LGSC  467
HepatitisE_HEPEVIRUS          324  MLFGATLD-----------DQAFCCSRLMTYLRGISYKVTVGTLVA-----NEGWNASEVALTAVITAAYLTICHQRYLRTQAISKGMRRLER-----EHAQKF--ITRLYSW---LFEK  417
CuthroatTrout_FISHHEPEVIR     316  HSYACGLP-----------DETFSYTRLVIYAKGLAFRAKIGAVAL-----NEGWNPDTTAFSAVVAGVFMDVAVKRYLRSKGISNCMKELKK-----EQNAGF--WSLLWQR---ICIW  409
PelargoniumZonate_ANULAVI     347  EEICFRCPKDVSK--IEVMGGESETCGIMSVLYS--STIIVNGMTM-----MAGERLDVLDYHHVAFSLMLSARRKFDMFGKAMNSLEWKGWVS----HFFKSLWPSGDLRDL---FGRY  450
CucumberMosaic_CUCUMOVIRU     370  EEIAMRCYNDK-------KAWMEQFKVILGVLSAKSSTIVINGMSM-----QSGERIDINDYHYIGFAILLHTKMKYEQLGKMYDMWNASSIS------KWFA-----ALTRP---LRVF  463
BromeMosaic_BROMOVIRUS        340  EEIAFRCFKES-------KEWTENMKAVASILSAKSSTVIINGQAI-----MAGERLDIEDYHLVAFALTLNLYQKYEKLTALRDGMEWKGWC--------------HHFKTR---FWWG  430
CitrusLeprosisC_CILEVIRUS     463  FKYGMTVD-----------SNKFVYDGLLKSGVGIAARRNIGGTTM----VDPSFPIPVRKLQTFIVTMYMLLYQEKWEA--TQGLVTMKMLAD----QYRTRS-SKNGIVRF---LTNV  557
OliveLatent2_OLEAVIRUS        328  LMYTITACKG----------LYDRASARSTPLSAKASTVIINGMSY-----QVGEKLDPISFPYLAASFYMQAQKAVFEVQQVVDLHTPNR-----------------NLWSW---FKKK  412
JapaneseHolly_PTERIDOVIRU     676  FSACLRSLYTFE-----PKDHRKNFQVVITYLASHSQSIIINGTAV-----TRREPLPAEWLNPIAMAIYYQLCVVKDDESQALLNIDNCQITK----IFHFSN-FVSALKDI---VTSF  777
RaspberryBushy_IDAEOVIRUS     536  IEVCLRGVKPINFGNCDDAVHMDNLRIIQNHLLSHSQTLVLNGSTI-----IREEAIPFKDFSPVSVTIYFEILLTRYKESLSLAWFHAGLGPD----FKLGSW--VSSLKRV---FYRI  641
TobaccoStreak_ILARVIRUS       372  SEAAFRQFSKT-------KDPETLVQYIATMLSSSSNHVVINGITM-----RSGSPIKFDEYVPLAVTFYVMAAWRYKMIAPGIDAVKTRTEKN----VDILDE--KGLIKED---FNVV  470
TobaccoMosaic_TOBAMOVIRUS     403  LNHIRTYQ-----------AKALTYANVLSFVESIRSRVIINGVTA-----RSEWDVDKSLLQSLSMTFYLHTKLAVLKDDLLISKFSLGSKT------VCQHV--WDEISLA---FGNA  495
GremmeniellaXL1_ENDORNAVI     553  LNRLLQ---------------EYSWEATMAYASSMIGKVYATSSGL-----HLKWNMTNAEVRDHCLVAYWSTNRVNESMRPLIQQAERNNRDP----DFLTGL--WTAVKNWGRDFGSH  646
TobaccoRattle_TOBRAVIRUS_     387  LDYIARLS-----------DQQLTISNVKSYLSSNNWVLFINGAAV-----KNKQSVDSRDLQLLAQTLLVKEQVARPVMRELREAILTETKPI----TSLTD-----VLGLI---SRKM  478
SoilWheat_FUROVIRUS           389  IEYTGRLK-----------PAQLTSDNVNTYLTSTNNTIIIGGSSK-----KNATKVDATLIQQITTTLIVWTELMNQRQKRVLEKLRMQMKDD----VDFMT-----LAHAT---FLKM  480
Potato_POMOVIRUS              393  LEYAFQLK-----------DNQVTAEGLISYFASTNNAVVIGGSAR-----KTSEKVDPKLLPMITTTLMVYQELQKAKQKRVLGKLKSKVKEE----LTLSG-----ILESV---VHRV  484
BarleyStripe_HORDEIVIRUS_     393  AAFIMKNK-----------EKDLNVAVLKNYLSAVNNSYIFNGSQV-----RDGVKIAPDLISKLAVTLYLREKVYRQRENSIISYFEQEMLHD----PNLKA-----MFGDF---LWFV  484
IndianPeanut_PECLUVIRUS       399  MEWLMRNK-----------ANALQYETLLNYLVSTNVSAVFNGCQV-----REGLKTDPWILCKLAMTLLVREEFNREKQKKVLEMLRLSGSEQ----ISLKR-----IFKGV---FEKF  490
NudaureliaBeta_BETATETRAV     333  CVYVGTRVQS--------SGKSITLAEIVQYIRTRLTRIILNGTVH-----EKTWTIAEQDIERLAVSIMFRKNVERAVSEKALMRAQKKCKS-----AEKQA-----LLPVW---MRRI  426
LettuceInfections_CRINIVI     344  IEYVANTGI---------KIDDKMVEWTYSQYRAKKTVTIKSGKVT-----QKETRIRKELIPGFIAIIMSEGIRAREKTHYLAKMLYTSHYKP----SIVNII--FRLIMHF---LGGT  440
Rubella_RUBIVIRUS             338  LSYCKTLS-----------PDAYYSERVFKFKNALCHSITLAGNVL-----QEGWKGTCAEEDALCAYVAFRAWQSNARLAGIMKGAKRCAADSLSVAGWLDTI--WDAIKRF---LGSV  436
Consensus_ss:                      hhhhh               hhhhhhhhhhhhh   eeeee  eee     e      hhhhhhhhhhhhhhhhhhhhhhhhhhhhhhhh          hhh    hhhhhh   hhhh
			..αF             αG	    βM  ..βN...	     αH		  ...... αI....
                                                                                                                                             or β1 (alphavirus only)
                                          End of Iceberg region |
Sindbis_ALPHAVIRUS                 --------------------------------------
Alfalfa_ALFAMOVIRUS           464  GPTF--------LSA-------LDKVNVP---------  477
GrapevineLeafroll3_AMPELO     848  VSVY-------AKA------------------------  854	- Amphipathic α-helices predicted by Amphipaseek are underscored with a thick line.
CitrusTristeza_CLOSTEROVI     452  FLGI-------QDAV-------ERCVKSY---------  466	- The experimentally known amphipathic α-helices in bromovirus and alphavirus
BeetNecrotic_BENYVIRUS        714  VDPI-------------------SFLWTW---------  723	  are underscored with a double line.
SclerotiniaL                  465  AATM-------------------PLLPAL---------  474	- NB: the oleavirus sequence has an apparent deletion of βK to αF included; we could
Helicoverpa_OMEGATETRAVIR          --------------------------------------     	  not detect an obvious sequencing error.
HepatitisE_HEPEVIRUS          418  S-------------------------------------  418
CuthroatTrout_FISHHEPEVIR     410  FGDS----NEHRHMD-------HNTLKGW---------  427
PelargoniumZonate_ANULAVI     451  FPSL----------------------------------  454
CucumberMosaic_CUCUMOVIRU     464  FSSV-------VHAL-----------------------  471
BromeMosaic_BROMOVIRUS        431  GDSSRAKVGWLRTL-----------ASR----------  447
CitrusLeprosisC_CILEVIRUS     558  FSPD-------KVGE-------TQHPSSTYDLGK----  577
OliveLatent2_OLEAVIRUS        413  FELK-------AHA---------FLFA-----------  423
JapaneseHolly_PTERIDOVIRU     778  LKFV-------LPS---------KLFEK----------  789
RaspberryBushy_IDAEOVIRUS     642  LGFP-------ANL--------LKYVLNA---------  655
TobaccoStreak_ILARVIRUS       471  NALL-------EDA--------GLIEPKLPHFTDVIKN  493
TobaccoMosaic_TOBAMOVIRUS     496  FPSV-------KER----------LLNRK---------  507
GremmeniellaXL1_ENDORNAVI     647  FDPS-------GSE----------LIQTF---------  658
TobaccoRattle_TOBRAVIRUS_     479  WKQFA-----NKIAVG----GFVGMVGTL---------  498
SoilWheat_FUROVIRUS           481  FGKVS-----YYQRA-------LRCFANW---------  497
Potato_POMOVIRUS              485  FGRQS-----LYQRG-------LGVFAKW---------  501
BarleyStripe_HORDEIVIRUS_     485  PNTLS-----SVWKN------MRKSLMEW---------  502
IndianPeanut_PECLUVIRUS       491  FGSVS-----LKARV-------LRTVARW---------  507
NudaureliaBeta_BETATETRAV     427  ANWF-------QDKFQIDEEVVRKRYLEC---------  448
LettuceInfections_CRINIVI     441  KRFI-------YES-----------LVDC---------  451
Rubella_RUBIVIRUS             437  PL---------AER-----------MEEW---------  445
Consensus_ss:                                 hhh
                            .....[αI]....
```
