# Supplementary material for: Sequence analysis reveals a conserved extension in the capping enzyme of the alphavirus supergroup, and a homologous domain in nodaviruses
Source: Biol Direct. 2015 Apr 11;10:16. doi: 10.1186/s13062-015-0050-0 (PMC4392871; doi:10.1186/s13062-015-0050-0)
Supplement: Additional file 1: — Compilation of all supplementary figures and tables, in .zip format. [file 13062_2015_50_MOESM1_ESM.zip › Additional File 1/Figure S4 Noda MTase GTase.doc]

**Figure S4. Alignment of the MTase-GTase of the Noda group**

Conventions are the same as in Figure S2.

Thickly underlined: membrane-binding, amphipathic α-helices predicted by Amphipaseek (see Table S9C)

Underlined: predicted tm segments (see Table S9D)

```
Conservation:                      9
Nodamura_ALPHANODAVIRUS         1  MLNYET-------------IINGASSA-LNIVSRALGYRVPLAKSLALVAG-SCVVYKIIVH----------------------------------------------------------   47
FlockHouseALPHANODA             1  MTLK---------------VILG----------EHQITRTELLVGIATVSGCGAVVYCIS------------------------------------------------------------   35
Macrobrachium                   1  MKCNN--------------LLYG----------VELPMCLQSSMGMVVVTG-LAIVYLVIYILIYSFTHGPRL-----------------------------------------------   48
PenaeusVannamei                 1  MKCNN--------------LLLG----------VELPVCLQSRLGMVLVTG-IAMVYLLLYIVTYAVIHGPRV-----------------------------------------------   48
PlasmoparaA                     1  MSFGTLFS-----------------------------------------------RYTLMVS----------------------------------------------------------   15
BetegovirusSF                   1  MMPNYLFEAI-------FNR--------------------------KVIMSTPNQVIDIAV-HFYSRTISYPLP----------------------------------SKTTTLLAL---   49
StripedJackBETANODA             1  MRR-----------------------------------------------------FEFELA----------------------------------------------------------    9
EpinephelusNervous              1  MRR-----------------------------------------------------FEFALA----------------------------------------------------------    9
Atlantic_Halibut                1  MHS-----------------------------------------------------YEFHLA----------------------------------------------------------    9
SclerophthoraA                  1  MPW---------------------------------------------------KPGKCHLL----------------------------------------------------------   11
Pariacoto                       1  MEEHIPLPSQYE---SPKALPPR----------VPSSRWLRS--LRPRLAN-SCLALKIRAH----------------------------------------------------------   46
AlphanodavirusHB2007            1  MFVVKRE-----------------------------------------------------------------------------------------------------------PCP---   10
BatGuano                        1  MGR-----------------------------------------------------DTLSLLL---------------------------------------------RGTALFPACC-I   21
Wuhan                           1  MSSVIKTIVVKTLQCTVKCVCVGVRQQYGLLRRHPKTI------ACALVGG-IIAHDVLHLYAAVYRVENPWEF----------------------------------VPTEGP------   73
Tetnovirus2_TETNOVIRUS          1  MQL-----------------------------------------------------QNYHL-----------------------------------------------------------    8
Tetnovirus1_TETNOVIRUS          1  MLY---------------------------------NIAHDLTILGYDISERSLSHPRIIV-----RPFLTELVSVLRDVAKEALITPVIVPTTLTGSLMAYVYYQVANTYDQHDNPLIR   82
Consensus_ss:                                                                               eeeee


Conservation:                                                                                                  5
Nodamura_ALPHANODAVIRUS        48  --RRTLVAFLV-----IGPYATVV---------------------------QHRL-------PMAL----------QRAIIEYTR----------EDREISLFPQNSIVSAEHA-----R  101
FlockHouseALPHANODA            36  ----KFWGYGA-----IAPYPQSG---------------------------GNRV-------TRAL----------QRAVIDKTK----------TPIETRFYPLDSLRTVTPK-----R   87
Macrobrachium                  49  --SRRLASYVV-----IGPYEAIA---------------------------KSRP-------AMAL----------QRAMVDLTR----------RDVRTDWYPLNNLLMNKPH-----R  102
PenaeusVannamei                49  --SRRLVSYVV-----IGPYEAIT---------------------------TSRP-------AMAL----------QRAMVDMTR----------RDIRSDWYPLQSLLDIKPF-----R  102
PlasmoparaA                    16  -------SVSVV--GLATPWRYVMS-----------------------KTLNEFG-------EEW---------------SIEPAN------------LRSLFGDIQVVEFKRQ------   63
BetegovirusSF                  50  -AATGLTMYAT-----FTVAKHIT------------------------DAVHEAW-------DSFR----------TYLIHTASRVHK-----PQEIVRRTF-RETQFFHKHAD------  110
StripedJackBETANODA            10  --RMSGAAFCV-----VTGYRLLTS-----------------------KWLADRV-------EDYR----------QRVIADRKQILR-----DAAVIRTSIQKQMELVRISVR------   71
EpinephelusNervous             10  --RMSGAAFCV-----YTGYRLLTS-----------------------KWLADRV-------EDYR----------QRIIAEKKQILR-----DAAMIRTQIQREMELVRISVR------   71
Atlantic_Halibut               10  --RMSGATLCV-----VTGYRLLTS-----------------------KWLADRI-------EDYR----------QRVIADRKKIIR-----DAAMIRTSVQKQMELVRISVR------   71
SclerophthoraA                 12  --AAGLAGVCCIAYTKRGSLRDAA----------------------------------------------------SALCEWL--ADE-----PAIVSDLSRDAFTTTCVDPVV-----A   65
Pariacoto                      47  ---ESLVKIRL-----CKPYDAQSR---------------------------------------------------SKIIEKVIE--------RRETRKTLAHQLKDLKLVPVA------   93
AlphanodavirusHB2007           11  RRKMG--GDTEYRPRASAPIRRCV--------------------------DFVRK-------VLCR----------QDIVKMSKLMHRRKTVVPTDFRKKLHHAMRNIPLLSAP------   79
BatGuano                       22  RMRRGTKMWIV-----AGGIAVFGAYYVYEQR---------RGLMNWLKWVGDCV-------VSHF----------SMRLHHT----------RRH----LFNTRFYYHPAREI------   90
Wuhan                          74  ---RGVIVLRV-----YNALRFTAA-----------------------KWRWNCWTIFGKFPFNSE----------QFSRIMETSTER-----KPDLRPLLVPAHETIEVYPCN------  141
Tetnovirus2_TETNOVIRUS          9  --------KRVY---APNPLQVVV--------------------------------------HKLL----------PRSAIRPE----------KVIVRYKLSKMDYLRHIRLAPPTQEA   59
Tetnovirus1_TETNOVIRUS         83  IPMNCIYTLSI-----CFPYRTNISLNHKLSDDLPLIRDYYGMPITQKDWPTSNV-------GWYRRYFGTLRPNDQRLIYRYP----------RCPRSIPSLVRDYDWKLE--------  172
Consensus_ss:                            eeeee     ee hhhhh                           h hh       hhhh          hhhhhhhh                h      eeeeee
                                          βY            αY                                      .......αZ.......                  βZ
```

```
				 |Start of Core region
                                        107    114                                        155                 177 179
Conservation:                         6 H♠  67 R^ 7           8   5 65           5 6  7   D♥(?)               95D7Y 5 5         8  5695 9        6    7 5
Nodamura_ALPHANODAVIRUS       102  KADNGHPISGGTRDVARETISLAIRAAGFRHYEISPARQSP--AEAASHQHYAAADLVRAATEDKIQDGDVVVAIDIDYYLRDIDRYL-GRGVPFMAYTFNPVEVAGRDGD--SFFRIT-  215
FlockHouseALPHANODA            88  VADNGHAVSGAVRDAARRLIDESITAVGGSKFEVNPNPNSS--TGLRNHFHFAVGDLAQDFRNDTPADDAFIVGVDVDYYVTEPDVLL-EHMRPVVLHTFNPKKVSGFDAD--SPFTIK-  201
Macrobrachium                 103  LSENGHKTSGAVRDAARNLITSAITSLGMDKYEISPGGHTV-DEQLASHRHYAVNDLHRASADDAVKENAVIVAIDTDYYLRDPSIYF-SNNNPFILHTFQPITVAGKDGD--VRFTIS-  217
PenaeusVannamei               103  INDNGHKVSGAVRDSARNLITNAVSAMGFDKYEVSPGGHTV-DEQFASHRHYAVNDLHRSSGDDPIRENNVLVFIDNDYYLREPSMYF-SNPNPFILFTFQPITVAGYDGD--VRYTIS-  217
PlasmoparaA                    64  -PGHPHGAAAADRSAGSYFIERFARDVGREVVYYQCSNSDLRNGRSGAREWYWMKDAYVQPKLFSLSSSSMVSIIDVDQYVD-MNWFLTEHFCPVLLYTFQPSVCAADRGE--YSYTFNS  179
BetegovirusSF                 111  -VNHTHGPSAADRSSASEFATNFAYSLGVTPFFIQMSAADQRHSKRGNRTYFWSKDVHVKPSNEKPNPEDVRVLVDVDQYFD-MPYLLANDPAVYLLYTFQPHKAASTCLD--YSFTFDA  226
StripedJackBETANODA            72  -KGHSHQEAATERNSATDTMIGVVEKCGYEPYIISPSPREK--EYHGSRQFYSLADFRQDYRRDEITDRHVIVMTDVDYYVD-MHELV-GLGVPILLYTFQPSTVSGEVKD--GYFTIT-  183
EpinephelusNervous             72  -KGHSHQEAATERNSATETMLGVVEKCGYEPYVISPSPREV--GYHGSRQFYSLADFRQDYRRDDITDRHIIVMTDVDYYVD-MHELI-GLGVPILLYTFQPSTVSGEVKD--GYFTIT-  183
Atlantic_Halibut               72  -KGHSHQEAATERNSATETMLSVVEKSGYEPYIISPSPREA--EYHGSRQFYSLADFRQDYRRDEITDRHIIVMTDVDYYVD-MNELI-GLGVPVLMYTFQPSTVSGEVKD--GYFTIT-  183
SclerophthoraA                 66  ISGHTHASAASLRTAATRYAQNVAQYCGTEVYVVGMSRSDQRKDMKGSRQWYWTKDVNAANRNDRPGHRDIRFLCDVDYYVD-MPAMLVAEAKPVLLYTVVPEEATSSANDESAFYFEA-  183
Pariacoto                      94  -RDHTHGRAAKFRTSANIWMNEAMRAAGYEPYNVSMSNHDI---ERGNRYFYFAKDLTIPYRNDPVSDNTGFVFCDVDYYAD-MEKWM-QHFKPMLLYTLVPESLSYHCDD--HSFHVN-  204
AlphanodavirusHB2007           80  -ADHTHPDAARYRTSVACRIEEVILNAGFRPYSVSMSKRDK---YDGCRYYFMQRDYDKEFRDDPITSGNVIMMIDVDYYVD-FNRYL-QFGNPVMIYTFVPTSAGGPTTD--GTFSIE-  190
BatGuano                       91  -PGHSHPSAASIRNGAVFSMDNYASITGATFYTVSYSDRDR---SRGQRLYYQPRDYCLEARNCDMRPTDIIRMVDVDYYVD-MHYWL-GLGHPVLLYTFVPTRAGGKTFD--GSYSFS-  201
Wuhan                         142  -NIHSHPRSAEFRSSANEYLVRSVRRAGYPPYVVSSSKRDF---CDGNRFFYCPKDFGMQFRNDPISNNHALVFTDVDYYAN-MNRWL-QLFLPTCLYTLVPTRLNYSNDE--FAYRFE-  252
Tetnovirus2_TETNOVIRUS         60  NKLHPHPMSAELRRRALQAVRYMCKQLNKQMHVISPGPNESLLADASTRYHYWARDLMVAESNHPLTHDSLLYLQDVDYYTD-MNYIL-GAGLPVVLYTIIPRKAASRKQE--YAYRFE-  174
Tetnovirus1_TETNOVIRUS        173  -SVSPHPEEAAQRNAARFNLPSMALSMGYTPFTISGRPSEA---TLGFNLVYNYRDTERLLRGDVLTDRNAIILVDTDYYID-MPKLL-TTGLPILMYTFVPQRLAGSTQG--TNYYIDK  284
Consensus_ss:                                hhhhhhhhhhhhhhhh    eeee              eeeeehhhhhhhh         eeeeee  hh   hhhhh h    eeeeee  eeeeeee    eeeeee
					    αA	      βA    [αB]    βB αC		 βC   αD’ αD       βD    βD’     βE

                                                 | start of Iceberg region
                                           226 229231                                                                                       287
Conservation:                      5  5 6 7 9G  ♦ H 5976  5                                                      5                   7 57   P
Nodamura_ALPHANODAVIRUS       216  NNQVTFDVSGGGSWSHEVWDWCAFGEFIETRDASW------------------------------LAWFA----RAVGLTKSQIHKVHYCRPWPQSPHRALVWCLPVASYW-RFTFI--P  298
FlockHouseALPHANODA           202  NNLVEYKVSGGAAWVHPVWDWCEAGEFIASRVRTS-----------------------------WKEWFLQLPLRMIGLEKVGYHKIHHCRPWTDCPDRALVYTIPQYVIW-RFNWI--D  289
Macrobrachium                 218  DNQVDYRVDGGGRWQHKVWNWCDYGEFLYLKNILGI-LSI--------------------------NWWL----SFFGIRKVIYQKIQHARPWVDCPNRALVWGLPQFTRY-MITWL--P  303
PenaeusVannamei               218  NNEVDYRVDGGDRWHHKVWNWCDYGEFICFPMKPRW-FTF--------------------------DWLI----SCLGVQKVTYQKIHHSRPWVDCPNRALVWGVPQFTSY-IISWL--P  303
PlasmoparaA                   180  NNEVIYRVAGGAEYVHKVWNYDTDVVTTTRLFAGKSWTKWRN----------------------WIPFVT----CAYLVDKR-----------QMDADHQIIGLFPVKRWFGPLALL--A  260
BetegovirusSF                 227  DGRVNYQVNGGGSFRHHVWNYAKDVFTVERTMFGV-------------------------------PIST----TTYLIDRR-----------LTSENHELILLTPTCTVDFPFAFL--L  298
StripedJackBETANODA           184  DDHVHYRVAGGKDVRHRIWNYNQDTMFVRSKPRGFW-ASLKQILRDITGITALCGYLYLKLGIAPFGDQV----TLFTVDQF-----------KMGEHRNIVSIVPFATC--RSNLL--K  283
EpinephelusNervous            184  DDSVHYRVAGGKDVRHRIWNYNHDTMYVCSRPRGFW-ANLMQILRDITGVTAICSFLYTKLGIAPFGDPV----TMFTVDQF-----------KMGEHRNIVSIVPFATC--RSNLL--K  283
Atlantic_Halibut              184  DDSVHYRVAGGKDVRHRIWNYNQDTMYTVSRPTGFW-ENLKRILRDITGITALCGFLYNKLGMAPFGDPV----TMFTVDQF-----------KMGEHRNIVSIVPFATC--RSNLL--K  283
SclerophthoraA                184  DGSLRTMVAGGGGYSHCIWDYAADSFLVVKKICGV-------------------------------PIRA----VAYAVERK-----------QVGKHRQMVLLAPVRAFS-GLAAILAY  256
Pariacoto                     205  DDRVFFDVRGGASYSHQLWDYTGDTICVRGKNKEL---------------------------------------LVFTIEQKCI---------QGDPHRRIIFLEPAARVA-WPFYK--P  273
AlphanodavirusHB2007          191  NDHIVYRVSGGATYRHEVWDHNHDFVSVKDKHGNL---------------------------------------LTYVVEQHTC---------EFDPQRRIIGYYPVTSVP-AHCFV--K  259
BatGuano                      202  GNRVKYRVNGGATYEHQLWDYNHDFITVDKWTGV----------------------------------------WVYSVDQKI----------TTDPERRVVQLTPRCFVPKPLSWF--V  269
Wuhan                         253  NNEIVYNVTGGGEYRHSLWDYKGDTVTVVDEYGNL---------------------------------------LIFDIEQRRV---------QGDEQHRLIWLLPKAKIT-DPLWIVGP  323
Tetnovirus2_TETNOVIRUS        175  NGQFLFTSNGGTAFSHLLWDYDLDTCAIERSPQTWL-SWFLG----------------------PRERMV----TFFEKTEV-----------YLGMDRYLVFFIPRYRVS-GWTYD--K  253
Tetnovirus1_TETNOVIRUS        285  HSQVHFRAAGGTTWNHYLWDYNHDYLTSPSVNNKI---------------------------------------VSYQVYQT-----------QLSENRRLIILFPKTVA--PYLR----  348
Consensus_ss:                        eeeeee     eeeeeee    eeeeee                                            eeeee h                  eeeee   hhh
				βF	 βG	  βH		 	   variable     α or β              βI  α or β
                                                ^    ^      ^    ^
                                                W215 W220   E227 S231
```

(residues substituted experimentally)

```
                                                                      333
Conservation:                             7 5                         S/T5  5       5        5                                            5 5   5
Nodamura_ALPHANODAVIRUS       299  TDLHTRTLRRVRYQDTSR-PGWNSIVSTGSE-GLNISLGREGAD--HCVTIPKVHYDMLMGLSSAQ---SLSSRMI-GLKYT----------------DPSVLATVAQYYQ---------  385
FlockHouseALPHANODA           290  TELHVRKLKRIEYQDETK-PGWNRLEYVTDKNELLVSIGREGEH--AQITIEKEKLDMLSGLSATQ---SVNARLI-GMGHK----------------DPQYTSMIVQYYT---------  377
Macrobrachium                 304  IEMNARELSRVNYQCASR-PGWNCLVDHTST-KLVASIGREGND--CHVELAKEDLDVVLGLSTMQ---SVTSRLL-QMGYK----------------QPQTLATVCQFYN---------  390
PenaeusVannamei               304  MELHARNLTRINYQCGAR-PGWNCLVDHTKG-KLTASIGREGND--SHVEISKEDLDIVLGLSTQQ---SVTSRLI-AMGYK----------------QPQTLALVCQFYS---------  390
PlasmoparaA                   261  RCLKGRPLSRFKIVR----GDFLRLLNHGHN-GMTISTGKVESP--LCGTITAREDAALASLARTTKSGLTLMHVKKLMPDD------------------TLGATYVYEYH---------  346
BetegovirusSF                 299  SQLGSNPLLRLNPIV----GNFIRLDVRGV--ELLTSTARISEY--VCCTIPRVADDRVSEINRLTKHDLSPASVE-SAITP-----------------LPIEPEDTTYYREMAVVLCSF  392
StripedJackBETANODA           284  ISEYGAELDYMRYQQRNNNANFNAVTYISQE-GPLISLGLEGNF--ASVQLPLQDFENIRTAYELSKNNNLSDTVR-RSARS------------------CKEAAIIHKCL---------  372
EpinephelusNervous            284  ISEYGAELEYMRYQQRNNIANFNAVTYISEN-GPLISLGLEGNF--ASVQLPLQDFENIRTAYELSKTNNLSDTVR-RSGRP------------------CKEAAIIHKCL---------  372
Atlantic_Halibut              284  ISEYGAELEYMRYQQRNNIANFNAVTYISEE-GPLISLGLEGNF--ASVQLPLQDFENVRTAYELSKTNNLSDTVR-RSGRP------------------CKEAAIIHKCL---------  372
SclerophthoraA                257  LLLETKELRRFNPIVEAGGEKFVRFNVMSSDGALMVTTARPGSS--LCATVSCADDDAIATVARLGSTNLMLPTTA-SWVKD------------------RPAAAVLTDYH---------  346
Pariacoto                     274  MKVEVGLKRKCMTAGQVN-VLYEPI-------DDKISLSASGSR--HTVETTGRTLAAITARMKNKTSPPMVADVE-RILRDA-------GDK-----EACVNAPILFELI---------  361
AlphanodavirusHB2007          260  PD--TLGVKRLKFTSDNM-NSVRDVV------SNTISVACHGSP--HSVTIPINLFDAIKIRHDRATK-PVIADVE-RLLQAA-------EPKI---PQASTKAPILFGLL---------  347
BatGuano                      270  PRNPLRRLNVLHES------DWSVLTSQDSSGTQYISIAKDAST--YAVTMPRELYEAIKIRHDTSGNARNISDIE-RMLLKH-------GFE-----DPHIKAAILYDCI---------  359
Wuhan                         324  TEWYDKLLVRKTVQQGRLKILWEPI-------ADDLSIGLHGSN--YSVSLKGELFEAIRTRISCKDSTPYVSDVE-RMLREA-------KHK-----NFLTDAPILYHCF---------  412
Tetnovirus2_TETNOVIRUS        254  MNLGISRLVRRSVTSNG-----VTIWPDRAT-PGLLSLALQGEFNYQTISIKREMLETVRLKMNLIGDTFK----------------------------SSTHAEISRYLV---------  330
Tetnovirus1_TETNOVIRUS        349  -RVEATPLVRARFNQGE-----YCLMKTKTE-QQEISISM-GDE--HVLTLPLDVFFELRGRFRNWKQADILYNVA-CLEQNAKVLLQNKGYKTEDVNDVVARISVLHAYL---------  448
Consensus_ss:                      hhh    eeeeee         eeeeee      eeeee       eeeee hhhhhhhhhhhh       hhhhh hh                        hhhhhhhh
				      βJ	        βK      βL	    βL’    αF		  αG	    	            αG’


                  end of Iceberg region |                                                        | Start of RdRP
Conservation:                                                                                 5  7             597        6           5 775             7 8
Nodamura_ALPHANODAVIRUS       386  GKNVEVADADRIGR--AINPKVHWPAHVEV-----------DEAEVSARVYASPLVSDENMMPMIKRWETLSLSLDRRVTFQR----NPKVPGKRLRAYAIEFVDLVVPER----GVGVP  484
FlockHouseALPHANODA           378  GKKVVSPISPTVYK--PTMPRVHWPVTSDA-----------DVPEVSARQYTLPIVSDCMMMPMIKRWETMSESIERRVTFVA----NDKKPSDRIAKIAETFVKLMNGPF----KDLDP  476
Macrobrachium                 391  SAAYDILSCSIVAR--PSQPPVHWPLASEI-----------DQPTTSFRNYSNNIVTCGNLCPQLKRWEVLSNSLEHRVTMVA----NNKVPTPRIARFAEEYVRLVVPEA----NVGVP  489
PenaeusVannamei               391  GNANSTLDSHIVAR--PSQPPVHWPIASEI-----------DIPTTSFRNYSNPVVCCSNLCPQLKRWEVLSNSLEHRVTMVN----NTKVPNVRIARLAEEFVRLVVPEN----GVGVP  489
PlasmoparaA                   347  SQK-LPEATVVSYSGAKKDGVRGYQFNPM-------NYD--PLAKQSLVSFMSP-IIDGGFCPDMTE-DNVKQAIAGRIVDVK----SVTTMDRFLMKTIDEFAHLLLKSAGVTAQSLLP  450
BetegovirusSF                 393  YRECTERKRDSVNP--PNRDVLRVQHGRYD-----------PEAPETMLPFMSP-LLVGAYSFDKTS-DNEKQTFVGRVVEPV----NNKTTTPLMERCMNEFVDKMFPNK----HLLVP  489
StripedJackBETANODA           373  QAG-CDLASEVVHK--PGELARHYQALGDT-----YDIDPSEQGKCYAREYAPGPLTQTAVFPSESR-SNELATIDGRIAGPQAKAKSREHITPKMHKVARDFVRHLVPTA----GLGRP  479
EpinephelusNervous            373  QAE-CAVVSEVVHK--PGDLARHYQAVGSA-----YDTDPAEQGKCYAREYAPGPLTQTAVFPSESR-SNELATIDGRIAGPQAKAKSREHITPKMRKVARDFVHHLVPIA----GTGRP  479
Atlantic_Halibut              373  QAE-CVLASEVVHK--PGDLARHYQAVGSL-----YDVDPAEQGKCYAREYAPGPLTQTAVFPTESR-SNERATIDGRIAGPQAKAKSREHITPRMRKVARDFVRHLVPEA----GLGRP  479
SclerophthoraA                347  RKC-GKKAKFVVYP--VEQGVRAYQYKPD----------EFDQAARPKLQAFMSPIVHGAFAPVLNR-AGEERCVEGRINSLRK---PEPHPSNFRDRCIDEFADLVVSGM-----LLEP  444
Pariacoto                     362  PEA---KFRVNVVK--TTATPTHFQPLGP-------LRT--EDGETCGHAVTTTLATAPALLPMRGV-NSDVATVNGRVKKPA----NTVIPFKEYKEYASEFVEFLVPEP----GVGHP  458
AlphanodavirusHB2007          348  NAK---PSANIGAT-SSIPPAKTFQTLKP-------LAA--EDGKPCGRAVAPPLTTAPAALPARGV-NNDHATIIGRVVKTR----NTTLPPHEWKKYDDELVNHLVPRA----GIGVP  445
BatGuano                      360  SQV-RIPTTAPIVT--ACGMASHYQTLKP-------LIM--ETGKLYARSVGPPIAP-EAVFPVVSY-NNDVACIEGRVEKVR----NNTIPSGIYNQFAREFLEQMIPEDE--VGKGVA  459
Wuhan                         413  QDD--VIIRPNVVK--TGSFPVTYNAIPKK-----APSVT-EDPKMPGQVVTTPLVSQPALFAGKGF-NADKACIEGRIEKVR----NVTKFPIKYVRYAAEFVKLVIPETI--AGTGVP  515
Tetnovirus2_TETNOVIRUS        331  NNK-VVPVEESALA--ATILCRLFGKIDIQLGPDWYSTGLDDIIRTGGQRLHPPLLNHPALLPNRSA-KVEQITIDERIVGPQ----NVKNP-HQYRRFAEEFVEMSIPEP----NVGVP  437
Tetnovirus1_TETNOVIRUS        449  ----RDHANQELYT--LEHYTDMLHYRYDN-----GDLWG-TTLKHAQRAAMLPLTTAPNVAPVTDA-DADKTMVFGRVDTVH----NKIVPPHVFNSYAHEFIELTLNGV----HNLHP  547
Consensus_ss:                            ee  eee                                             hhh       hhhhhhh  ee              hhhhhhhhhhhhhh
                                           βM
```
