# Supplementary material for: Sequence analysis reveals a conserved extension in the capping enzyme of the alphavirus supergroup, and a homologous domain in nodaviruses
Source: Biol Direct. 2015 Apr 11;10:16. doi: 10.1186/s13062-015-0050-0 (PMC4392871; doi:10.1186/s13062-015-0050-0)
Supplement: Additional file 1: — Compilation of all supplementary figures and tables, in .zip format. [file 13062_2015_50_MOESM1_ESM.zip › Additional File 1/Figure S5 Chropara MTase GTase.doc]

**Figure S5. Alignment of the MTase-GTase of the Chropara group.** Conventions are the same as in Figure S2.

Underlined: predicted transmembrane (tm) segment Thickly underlined: Amphipaseek predicted membrane-binding, amphipathic α-helix

```
                                                                                                 N-terminal extension..
ChronicBee                      1  -MN---------------------LSNVNDVLSSVSSTLRPGEGGMRQIEYIFD-------------PPSLPTRIYNSVHDWFY----PPL------------HPIAAAIKAAYTNF-GV   68
AnophelineC                     1  -MD---------------------IKKIIEHL-------DPKNETFYSIEQAFD-------------PSP----IWVRATRWFRVRFSPEP------------NLLVRGIKTAANSLVGG   62
LakeSinai1                      1  MVKLAFAAFVSLYIICQVPPISYWIEDLIDDF-------DTMCSHEYAAADAYNNFVHRHRVAAYAAGVR----IHRYRTPWYCAASRSVPVVHPLNWLMTQYDLTTGHVRELLDRL---  106
LakeSinai2                      1  -MKVVFLVSFAVFLICQVPPVSYWLEDALDEY-------NTMCSYEYAAAEAYNNFIHKHRVAAYAAGVR----ILRYRTPWYCFASRSVPVVHPFSWLMGQYDATIDRVGTLMQRL---  105
Consensus_ss:                             hhhhhhhh     hhhhhhhhhhhh       h     hhhhhhhhhhhhhhhhhhhhh   e    eeee   eeeee         hhhhhh  hhhhhhhhhhhhhh

                                                          .. N-terminal extension                              | Core region...
                                                                                                                                            158     166
                                                         Transmembrane segment                                                              H♠      R^
ChronicBee                     69  QTKAAATAVVNAT---VPKTVISGLKICASSIG-----VTIVICSAG--------YWFYRTRHLKIVQVTNNGTMCQINELREYFETLCGPAGAHMDLPDSIPGHHLRIAAERRFLERAC  172
AnophelineC                    63  IPRVRNGAVNGAG---HAKTVARAAWDTAKYHS-----TVLVISAVGTCAAIYAATWIWRSRRVLFVQVINDGTMLPIPTLKDHFATRLSK--RPEDYGPTLPGLHLRIAAERRRAEQAV  172
LakeSinai1                    107  -------EVVNVTLRDGLRTVADTAFTAYMYYQIMWCQLALIVLLVGLFSAIIC------SSRVRVVRVRNRVRGYSITDLRNEFDESTSD----ITLP--LSRGHSCLNFQRRVAESWC  207
LakeSinai2                    106  -------EIANVSISDGLRTVADTALKAYFYYQIIWFQLFSIIMLVGFFSAVIC------SSRVRVVRIRNRVRGYSVAELRSDFEESMSA----ILAP--LSRGHSCLNFQRRVVESWA  206
Consensus_ss:                             hee eehhhhhhhhhhhhhhhhhhhhhhhhhhhhhhhhhhhhhhhhh        eeeeeeee    ee hhhhhhhhhhhhh     hhh       ehhhhhhhhhhhhhh
						    α0				     βZ		  αZ	variable	  αA...

                                                              201203                     226    233           248 250
                                                               H#Cº      D♥?              C*     C*             D Y
ChronicBee                    173  CEVFQQIGVRFRDVGGSRVRGEDYAHLKHLCNPCIDSSDILRDAKNPEPP--FSSCGGRGEVCPSRHDFPGALLCHSDYYL-YDELSYVIRGHTFIINHSFLEENKHFH-NEAFITKKGD  288
AnophelineC                   173  FEVFDDIKLRFRDVGGSLSRGKDYVQLKHVCNPVLDSADILRRAKADECP--FDTCKGVGQKCPQRAAFPGALLCHSDYYMDQEDLVACVRSHTFVINHRYTADSKVNHLGEMEVAVEGP  290
LakeSinai1                    208  LDQLLRYFHGVRFISASTGRWAELGHRIHRCSPVVM-----DGSFVPEATHHGVLCRRQPSACRDRFEIPACVISHADYYMSPDQLASTVTGPTFIINHDYLAPDSLSV-AEATTRVANG  321
LakeSinai2                    207  IDQLLRYFRCFRSVASSQGRWAEVGHRLHRCSPVVM-----DGAFVPEYDQRFSACRRHPSVCPDRFDTPAAILSHVDYYMTPDQLAAAVTGPTFIVNHDYSSIDTLSV-AEVSLRSAGG  320
Consensus_ss:                      hhhhhhhh eeee       hhhhhhhhhh   eeehhhhhh          hhhhh   hh         eeee    h  hhhhhhhh   eeeee              eeeee
			      ..αA    βA	           αB 	   [αC]      variable      βC      αD    βD           βE

                   end of Core region |
                                          297300    Iceberg region...
                                           G Y/F♦
ChronicBee                    289  GVIMSTPDGTEY-QHPYNVWKAEGCIIGSGGAAVYARVYRGPSMDVYYAYPASGTYHSDDPHRLKRSDQFENLRLSSGNIARLSFKSVKIQTTSGD------IILDCPRDPFERAVAKCG  401
AnophelineC                   291  YVKGGTSDGTRY-SHAYNLWQAEGSVVGKEGAFVYTRVWTGPSMDIYYAYPAPGTYRKDDPQALKRSTDINMVKLTTGHFAHITDHQVSVRTTDGT------LVMAFPRTELEKTVLKCG  403
LakeSinai1                    322  HVTSSVREGPIYGPHPYYLWNNEGVVVSSAGAFRYYRVGRLFDTSVYYAFPAAGTYSCDDSTNLRRST--------VGDLHYYSPHEKKFVSYSADDTNYHVFGTSVPRSLADYCAATFC  433
LakeSinai2                    321  LVTASVRDGPTFGPHPYYRWSDEGVVVASSGAFQYFRIGRLFDTTLYYAFPTSGTYSQDDPSALRRST--------SGDLHYYSPHEKRFVSYTADDTHYHVFGVSVPRSLADYCAATFC  432
Consensus_ss:                      eeeee           eeeee   eeee    eeeeeeeeee  eeeeee          hhhhhh      ee     eeee   eeeeeeee     eeeeeee  hhhhhhhhhhhh
			       βF    	   βG	 βH      βI    βI’        αE          βJ    βK	  βL     αF

                                                          End of Iceberg region   |
ChronicBee                    402  SGVRDLKFLPTLSSYIISKLTMHDLEINNAQDILELANIMCERFAFDFYSHSHLEYDPTSINIFTRLR------------------RHAMLFARAIRHTLARNLTCSIINRPILRQF---  500
AnophelineC                   404  GAIRDSKYISTVTSYVTAKLTAGDHNIASAADLTEIALAACETYALDIYSRHTRNYDPADLGTFKRLWLGFQDSTHRGRLWVTSAYTHDYVF----RHP--L------------SAL---  502
LakeSinai1                    434  RSARDDKFYDGLRSYYQNRCRAIGFTDARDTLILDFIIHLCDEASLKTFGFSRLSVAPSSWTAYCL----------------------SWLL-VKVNHMMPLALTSFVVS--ALHRFFGA  528
LakeSinai2                    433  RAVRDDKFYDSLRSYYQNRCRAIGFADARDTLMLDFIIHLCDDASLRTFGFSRLSSAPSSWSAYCL----------------------SWIL-VKVNHMMPLALTSYVLN--VLHRFFGA  527
Consensus_ss:                      h    hhhhhhhhhhhhhhhhee      hhhhhhhhhhhh    hh            hhhhhhhhh                    hhhh hhhh   hhhhhhhhhh  hhhhhh
				        αG     βM      αH			 αI                        αJ

                               Chropara-specific region
                                      PW       P            L      P  F  534
ChronicBee                    501  --TPWAFKSITMPAYIKHRRARHIDLRLASDKPKPF  534
AnophelineC                   503  --TPWLFRRVEMPGYVRHFTNTRVNLKSLNPDPRPF  536
LakeSinai1                    529  KSAPWNWASIHLPTYDMVTS--PFRLRMFGRNPTTF  562
LakeSinai2                    528  KAAPWNWATIHLPTYDMVTS--PFRLRLFGRNPTVF  561
Consensus_ss:                           hhheee  hhhhhh   eeeeeee
                                                                              αK    βN
```
