# Supplementary material for: Sequence analysis reveals a conserved extension in the capping enzyme of the alphavirus supergroup, and a homologous domain in nodaviruses
Source: Biol Direct. 2015 Apr 11;10:16. doi: 10.1186/s13062-015-0050-0 (PMC4392871; doi:10.1186/s13062-015-0050-0)
Supplement: Additional file 1: — Compilation of all supplementary figures and tables, in .zip format. [file 13062_2015_50_MOESM1_ESM.zip › Additional File 1/Figure S6 Alto Tymo Noda Chropara MTase GTase - Core region.doc]

**Supplemental Figure S6. Alignment of the core region of the MTase-GTase all groups (Alto, Tymo, Noda and Chropara).**

Conventions are the same as in Supporting Figure S1. Only the core region of the MTase-GTase is represented.

```
Conservation:                                                    H♠   5                                    5
HepatitisE_HEPEVIRUS_9626       1  -HQQIEILINLMQP--RQLVFR--PEVFWNHP----IQR-VIHNELE-LYCRAR----------SGRCLEIGAH-----PRSINDNP---   61
PlasmoparaA_301070430_gb_       1  --------------------FK--RQPGHPHGA-AAADR-SAGSYFI-ERFARD---------VGREVVYYQCS-----NSDLRNGRSG-   50
StripedJack_BETANODAVIRUS       1  --------------------IS--VRKGHSHQE-AATER-NSATDTM-IGVVEK---------CGYEPYIISPS-----PREKEYHG---   48
SclerophthoraA_45476494_r       1  --------------------VV--AISGHTHAS-AASLR-TAATRYA-QNVAQY---------CGTEVYVVGMS-----RSDQRKDMKG-   50
BatGuanoGF4n_297598937_gb       1  -------------------PAR--EIPGHSHPS-AASIR-NGAVFSM-DNYASI---------TGATFYTVSYS-----DRDRSRG----   48
Alphanoda_HB2007_29531469       1  ------------------PLLS--APADHTHPD-AARYR-TSVACRI-EEVILN---------AGFRPYSVSMS-----KRDKYDG----   49
LakeSinai1_335057597_gb_A       1  -------------------------PLSRGHSC-LNFQR-RVAESWCLDQLLRY--------FHGVRFISASTG-----RWAELG-----   45
LakeSinai2_335057590_gb_A       1  -------------------------PLSRGHSC-LNFQR-RVVESWAIDQLLRY--------FRCFRSVASSQG-----RWAEVG-----   45
SclerotiniaL_190694814_gb       1  -EAQVAALEASFP----EYNFV--SSYAHAHHP-STQAA-RHALELL-TWKLVD----------PAHTTEIGPS-----YSSLFRRARP-   64
FlockHouse_351066202_gb_A       1  --------------------PK--RAINNGHAV-SGAVR-DAARRLI-DEAITS---------VGGSKFEVNPN-----PNSSTGLR---   48
BlackBeetle_51014189_ref_       1  --------------------PK--RAVDNGHAV-SGAVR-DAARRLI-DESITA---------VGGSKFEVNPN-----PNSSTGLR---   48
DrosophilaNodaSW2009_2622       1  --------------------PK--RAFNNGHAV-SGAVR-DAARRLI-DEAITS---------VGGSKFEVNPN-----PNSSTGLR---   48
MacrobrachiumRosenbergii_       1  --------------------PH--RLSENGHKT-SGAVR-DAARNLI-TSAITS---------LGMDKYEISPG-----GHTVDEQLA--   49
Nodamura_ALPHANODAVIRUS_9       1  --------------------HA--RKADNGHPI-SGGTR-DVARETI-SLAIRA---------AGFRHYEISPA-----RQSPAEAA---   48
Tetnovirus1_TETNOVIRUS_30       1  --------------------WK--LESVSPHPE-EAAQR-NAARFNL-PSMALS---------MGYTPFTISGR-----PSEATLG----   47
BeetNecrotic_BENYVIRUS_23       1  -PREKKILERELG---FVPLYKQKSRAPRNHPV-LAALR-EVMRQEY-SASCNIL-------NTKLKTLVVGAA-----SREVNCYSSN-   70
Helicoverpa_OMEGATETRAVIR       1  -LKALETLHRLYYP--LRFKGG--TLPPTQHPI-LAGHQ-RVAEEVL-HNFAR----------GRSTVLEIGPS-----LHSALKLHGAP   67
NudaureliaBeta_BETATETRAV       1  -VETLEDAQEKYEG--MMFRSG--TKLPSTHIK-LAIDL-RVAEKDL-RRHVK----------NVPTVLEIGPS-----VESVRYAVQTR   67
CitrusLeprosisC_CILEVIRUS       1  -NDMERFLNEKFLG--YKIRLT--FSKNVAHNN-AAALR-RVLRFYM-RDKVGYRKDDDIPDGYHVKNKDVGAS-----GMDVIADEL--   75
BotrytisF_MYCOFLEXIVIRUS_       1  -KGPAALLAQVG----INTIHD--SPLSHSHP--A---CYALNAFHF-LKVIPRYL------HGKAEVWGTKDN-----WFRKLQAQIDS   66
Sindbis_ALPHAVIRUS_251215       1  --PFVVQLQKSFPQ--FEVVAQ--QVTPNDHAN-ARAFS-HLASKLI-ELEVP----------TTATILDIGSA-----PARRMFSE---   63
TurnipYellow_TYMOVIRUS_21       1  -KELLPLLNSYG----IPTSGL--GTSHHPHA--A---HKTIETFLL-CTHWSFQA------TTPSSVMFMKPS-----KFNKLAQVNS-   65
GrapevineFleck_MACULAVIRU       1  -PRWPSALNQAG----IPATSY--GHQSHPHP-----IHKTIETHLL-HEHWANRA------TLPSTVMFMKRS-----KFDKLRVSNAA   66
MaizeRayado_MARAFIVIRUS_1       1  -KEFHSFLHTCG----VDISGF--GHAAHPHP-----VHKTIETHLL-LDVWPNYA------RGPSDVMFIKPE-----KFAKLQSRQP-   65
SclerotiniaDebilitation_S       1  -SKQQSALESYG----IGSDPY--AVRSHTHA--A---EKAIENKLL-DIVGMNLR-----RRSVITMLWQKRN-----KAHLMGRSN--   65
ShallotX_ALLEXIVIRUS_2052       1  -TPEALTLERFG----ITTSPF--ATTSHTHA--A---DKIIENDCL-TIIGHYLP-----KREAVTLIQLKRS-----KIHLLGRQP--   65
BotrytisX_BOTREXVIRUS_375       1  -ESTAILLEKEG----VLTNPF--STTLHTHA--A---EKMLENRTL-NEVGKKLKV----HTGPFTFIQFKTG-----KLGHVGRGHN-   67
LoliumLatent_LOLAVIRUS_16       1  -TSAALVLEKLG----IGTHPM--SLAVHPHA-----PCKAIENQIL-NTVGHLLP-----KEQPVTFMAMKKS-----KLNMLRRHPS-   66
IndianCitrus_MANDARIVIRUS       1  -PDAADALEHLG----IGTNPF--SVKLHTHG--A---CKAIENQLL-YVVGTLLP------KERVTMLFLKKA-----KLNFMKRCPK-   65
BambooMosaic_POTEXVIRUS_9       1  -PTAANTLERYA----IPHNPF--STKLHTHA--A---AKALENDLY-RTASYLLP------KERVSFLFMKPS-----KMQYFRRRG--   64
PelargoniumZonate_ANULAVI       1  -ASDGEKMQKRFGG-TFDLQL---SKNLCPHSF-AGAMR-QCETLEC-LSSFPE----------DSLILDFGGS-----WLFHWQRQ---   64
BromeMosaic_BROMOVIRUS_96       1  IEEADAFRDRYGGA--FDLNLT--QQYHAPHSL-AGALR-VAEHYDC-LDSFPP----------EDPVIDFGGS-----WWHHFSRRD--   66
CucumberMosaic_CUCUMOVIRU       1  VKDSEVIRNRYGGK--YDLHLT--QQEFAPHGL-AGALR-LCETLDC-LDSFPSSG------LRQDLVLDFGGS-----WVTHYLRG---   69
JapaneseHolly_PTERIDOVIRU       1  -PDQLATLRSLFPG--FEFEYT--PRYDGPHNM-AAVIR-TAETLEA-VLSFPK----------DEPLIDIGGD-----FFFHVRNGF--   65
RaspberryBushy_IDAEOVIRUS       1  -DDDMRILRDHFPR--YELKFT--QNVDGPHNM-AAAHR-LLETHDL-LSNFPA----------DAPILDIGGN-----WFSHFRYGR--   65
TobaccoStreak_ILARVIRUS_2       1  -PDEKNALKANFPG--LEIAFR--DSCHSSHSF-AAAHR-VCETLNI-YNRFKT---------KTERIIDLGGN-----YVTHAKQGR--   66
Alfalfa_ALFAMOVIRUS_96269       1  -ATQQNILRANFPG--RRTVFS--NSSSSSHCF-AAAHR-LLETDFV-YRCFGN---------TVDSIIDLGGN-----FVSHMKVKR--   66
CuthroatTrout_FISHHEPEVIR       1  -PEQLEQAKKAFSP--QPVTYQ--PRVCLPHAL-AKHYT-TVLEGQL-KNAIR-----------GRPYIEAGPA-----PHQ-INSRP--   63
TobaccoRattle_TOBRAVIRUS_       1  -QKEQNKLMEIYPE--FNIVFK--DDKNMVHGF-AAAER-KLQALLL-LDRVPA----------LQEVDDIGGQ-----WSFWVTRGE--   65
SoilWheat_FUROVIRUS_96323       1  -QEQLQLLNDLYPE--RHIVSS--NCERGTHSF-AAASR-KIETDLL-LSRIPK----------RSWVYDIGGN-----WATHVKRNDG-   66
Potato_POMOVIRUS_20376948       1  -EDQIQLLAELFPE--RRVVTS--SVHRGTHSM-AAAMR-KIETDVI-FTSFPK----------NGVIYDIGGN-----WATHAKRDDG-   66
IndianPeanut_PECLUVIRUS_3       1  -SESLNALCSLYPE--FNVITS--NRERGSHTM-AAVCR-TLETLYI-KSLLPV---------NDTVVWDVGGN-----WLTHVKYSTN-   67
BarleyStripe_HORDEIVIRUS_       1  -SEELQMLINAYPE--YAVSSS--ACESGTHSM-AACFR-FLETEYL-LDMVPM---------KETFVYDIGGN-----WFSHMKFRAD-   67
TobaccoMosaic_Tobamovirus       1  -EEQTLIATRAYPE--FQITFY--NTQNAVHSL-AGGLR-SLELEYL-MMQIPY----------GSLTYDIGGN-----FASHLFKGR--   65
LettuceInfections_CRINIVI       1  -MDEKKMITNLFPD--IQMSFN--QKSYSNHGV-FNAMR-ACENFYF-SRKFK-----------NSDYIDAGGD-----VVSTLRSKN--   64
Rubella_RUBIVIRUS_3362846       1  -AAQKRAIVAVIP----RPVFT--QMQVSDHPA-LHAISRYTRRHW--IEWGP----------KEALHVLIDPS-----PGL--------   57
GremmeniellaXL1_ENDORNAVI       1  -VDIVEDLNNLHGS--GSFVRG--NMAAHSHIY-HHANR-LMLTEML-ASRHP----------RNALLYDIGGN-----INRHLDHGR--   65
CitrusTristeza_CLOSTEROVI       1  -ENQQVMLTRAYPE--FNINFI--HSVHSDHPV-AAGSR-ALENHLV-RKHA------------GTDYSDVGGC-----PLFHLRAGH--   63
GrapevineLeafroll3_AMPELO       1  -PREREKLRELFPE--LSIQFS--DSVRSSHPF-ANAMR-SCFNGIF-SRRC-----------GNVCFFDIGGS-----FTYHVKAGH--   64
OysterMushroom_28261418_r       1  -PSEKALADRWA----IPYSSS--PVGRVPHV-----MLKALENHRN-VNILPNYL------RGRVTVLSMKPA-----KLAALRARCP-   65
Tetnovirus2_302171585_gb_       1  ------------------PTQE--ANKLHPHPM-SAELR-RRALQAV-RYMCKQ---------LNKQMHVISPG-----PNESLLADAS-   52
Pariacoto_20260794_ref_NP       1  ------------------------VARDHTHGR-AAKFR-TSANIWM-NEAMRA---------AGYEPYNVSMS-----NHDIERG----   45
PierisRapae_63029695_gb_A       1  ------------------------CNNIHSHPR-SAEFR-SSANEYL-VRSVRR---------AGYPPYVVSSS-----KRDFCDG----   45
OliveLatent2_OLEAVIRUS_20       1  -EASVQELRFNWPCFTWEEESV--PLPPHPFA--A-YSR-RAFTRWA-IAQCGP-----------VPIKDFGGN-----WFLHWQWQ---   63
AppleStem_CAPILLOVIRUS_96       1  -PEDQELLTKHG----LTLAPI--GFKSHSHP-----ISKMIENHLL-YICVPSLLS----SFKSVAFFSLREN-----KVDSFLKMHSV   68
GrapevineA_VITIVIRUS__275       1  -DTLREILSGYG----MEFSVH--SFQGHAHP-----VSKMIENHML-YRVAPNYF------SNNTLVVSCKEG-----KIKRLRLKNAG   66
PotatoT_TEPOVIRUS_1943515       1  -SVVKERLTKAG----IPVSAF--CNQEHSHP--A---SKMIENHLL-YNILPNYL-----NLKNYTAISIKDS-----KVRKLLKNGV-   66
PotatoM_CARLAVIRUS_962609       1  -PLAKRKLSMAG----IYLSPY--SAVVHSHP-----VCKTLENYIL-YSVLPSYI------NSSFYFVGIKER-----KLQLLKSKCKN   66
AppleStemPitting_FOVEAVIR       1  -ELAKMRLFNSG----IYLSPH--SYRPHSHP-----VCKTLENNIL-FNILPSYL------DNSFYLVSIKKN-----KVDFLKRRHPD   66
AppleChlorotic_TRICHOVIRU       1  -EKTREWFTKSG----VYLSPF--AYVNHSHP-----GCKTLENHLL-FNVVASYIS----KYSYVACLSIKSN-----KMSKMERLGPN   68
CitrusLeaf_CITRIVIRUS_208       1  -SYQKKIASKVG----IELYPN--GYLPHSHP-----LSKIFENHLL-FDVLPGVV-----NTSRLVMCSIKES-----KVLVFKGIRDK   67
ChronicBee_188572672_ref_       1  --------PDSI--------------PGH-HLRIAAERR-FLERACC-EVFQQ----------IGVRFRDVGGSRVRGEDYAHLK-----   50
Consensus_ss:                        hhhhhhhh       ee            h  hhhhh hhhhhhh hhhhhh            eeeee        hhhhhh
```

**αA.......	βA	αB**

```
Conservation:                                                   D♥[R♣]
HepatitisE_HEPEVIRUS_9626      62  ----------------NVVHRCFLRPAGRDVQRWYT--------APTRGPAANCRRS--------------------------------A   95
PlasmoparaA_301070430_gb_      51  ----------------AREWYW-----MKDAYVQPKLF----------------------------------------------------   67
StripedJack_BETANODAVIRUS      49  ----------------SRQFYS-----LADFRQDYRRD----------------------------------------------------   65
SclerophthoraA_45476494_r      51  ----------------SRQWYW-----TKDVNAANRND----------------------------------------------------   67
BatGuanoGF4n_297598937_gb      49  ----------------QRLYYQ-----PRDYCLEARNC----------------------------------------------------   65
Alphanoda_HB2007_29531469      50  ----------------CRYYFM-----QRDYDKEFRDD----------------------------------------------------   66
LakeSinai1_335057597_gb_A      46  ----------------HRIHRCSPVVMDGSF----------------VPEAT--------------------------------------   65
LakeSinai2_335057590_gb_A      46  ----------------HRLHRCSPVVMDGAF----------------VPEYD--------------------------------------   65
SclerotiniaL_190694814_gb      65  ----------------AFAHYDMPLLTVRDGARRNLAMLSRHKTLDERETRALDALR---------------------------------  105
FlockHouse_351066202_gb_A      49  ----------------KHFHFA-----VGDLAQDFRND----------------------------------------------------   65
BlackBeetle_51014189_ref_      49  ----------------NHFHFA-----VGDLAQDFRND----------------------------------------------------   65
DrosophilaNodaSW2009_2622      49  ----------------NHFHFA-----VGDLAQDFRND----------------------------------------------------   65
MacrobrachiumRosenbergii_      50  ----------------SHRHYA-----VNDLHRASADD----------------------------------------------------   66
Nodamura_ALPHANODAVIRUS_9      49  ----------------SHQHYA-----AADLVRAATED----------------------------------------------------   65
Tetnovirus1_TETNOVIRUS_30      48  ----------------FNLVYN-----YRDTERL-------------LRGDV--------------------------------------   65
BeetNecrotic_BENYVIRUS_23      71  ---------------PSVHYYFA-NKDSKDLVRTTLELLHSALATK-YRN----------------------------------------  103
Helicoverpa_OMEGATETRAVIR      68  N------------APVADYHGC-TKYGTRDGSRHITALESRSVATGRPEFKADASLL---------------------------------  111
NudaureliaBeta_BETATETRAV      68  --------------DKERVHGC-TFSDARDNLRHNKIGYEAHYDRKIGPDAALLAAGI--------------------------------  110
CitrusLeprosisC_CILEVIRUS      76  ----------------TDVHCCTPDLDFRDHIRLERLKKYIYSHVCPTSKDHGIICE---------------------------------  116
BotrytisF_MYCOFLEXIVIRUS_      67  --------------QTTTLTHRNYAITARDHVRYPG------------------------------------------------------   88
Sindbis_ALPHAVIRUS_251215      64  ----------------HQYHCVCPMRSPEDPDRMMKYASKLAEKACKITNKNLHEKIKDL---------------------------RTV  110
TurnipYellow_TYMOVIRUS_21      66  ----------------NFRELKNYRLHPNDSTRYPF------------------------------------------------------   85
GrapevineFleck_MACULAVIRU      67  LV----------KSASNFLHLLNPILTARDADRYTH------------------------------------------------------   92
MaizeRayado_MARAFIVIRUS_1      66  ----------------NFAHLINYRLVPKDTTRYPS------------------------------------------------------   85
SclerotiniaDebilitation_S      66  ----------------CKDVYVNTIMEAKDLVRYDQ------------------------------------------------------   85
ShallotX_ALLEXIVIRUS_2052      66  ----------------SQDNFQNYCHEPKDVLRYGI------------------------------------------------------   85
BotrytisX_BOTREXVIRUS_375      68  ----------------QGDQIINYAREAKDLSRFET------------------------------------------------------   87
LoliumLatent_LOLAVIRUS_16      67  ----------------RADTFINPIYHPRDNVRYGLD-----------------------------------------------------   87
IndianCitrus_MANDARIVIRUS      66  ----------------FQDIFLNQHIEPRDVSRYCDF-----------------------------------------------------   86
BambooMosaic_POTEXVIRUS_9      65  ----------------DVDTFINADIVAKDLARYPLE-----------------------------------------------------   85
PelargoniumZonate_ANULAVI      65  ----------------HNVHSCCPVLDARDMARHQERMISMQKCVAHRPGKF--------------------------------------  100
BromeMosaic_BROMOVIRUS_96      67  ----------------KRVHSCCPVLGVRDAARHEERMCRMRKILQESDDFD--------------------------------------  102
CucumberMosaic_CUCUMOVIRU      70  ----------------HNVHCCSPCLGIRDKMRHAERLMNMRKIILNDPQQFDG------------------------------------  107
JapaneseHolly_PTERIDOVIRU      66  ----------------THVHCCAPILDSRDDERFSKRCMHLQKWVIDKQVSAYSSMSRRVKATLKEIPNLELLCDRLREFNDACRTPDDN  139
RaspberryBushy_IDAEOVIRUS      66  ----------------SNVHSCCPMLDLRDNERHTHRLTMTESLMSSLRHRYAGTIDLDPDAHLSRKVSDSMKEFYKRWAVHPKDLIRLY  139
TobaccoStreak_ILARVIRUS_2      67  ----------------SNVHSCCPILDVRDGARHTDRYISLAASVENRHRE---------------------------------------  101
Alfalfa_ALFAMOVIRUS_96269      67  ----------------HNVHCCCPILDARDGARLTERILSLKSYVRKHPEIV--------------------------------------  102
CuthroatTrout_FISHHEPEVIR      64  ----------------KDTHFCLVRGDARNHVRFATAPNNGLSRKVKRIYQG--------------------------------------   99
TobaccoRattle_TOBRAVIRUS_      66  ----------------KRIHSCCPNLDIRDDQREISRQIFLTAIGDQARSGKRQMSENELW------------------------MYDQF  115
SoilWheat_FUROVIRUS_96323      67  ----------------RKVHCCCPTVDIRDSARKTVRWASIEKYLDEKEEIPPEIGERIKRLQADED-------------RIYANLKSEK  127
Potato_POMOVIRUS_20376948      67  ----------------GFVHCCCPILDFRDAQRKMTRLIDFNRFIDDAKVVSAEKAAVAAQIKKDCD-------------LISENAKKDA  127
IndianPeanut_PECLUVIRUS_3      68  ----------------QDVHCCCPLLDYRDAMRKQERILSLELFARNGKEKTEEFDACYQQIRNF----------------ENTRRTAIS  125
BarleyStripe_HORDEIVIRUS_      68  ----------------REIHCCCPILSMRDSERLETRMMAMQKYMRGSKDKPLRLLSRYQNILREQA-------------ARTTAFMAGE  128
TobaccoMosaic_Tobamovirus      66  ----------------AYVHCCMPNLDVRDIMRHEGQKDSIELYLSRLERGGKTVPNFQK---------------------------EAF  112
LettuceInfections_CRINIVI      65  ----------------HNVHICSPRLDLKDAARHIQRATVIDGLKGYG------------------------------------------   96
Rubella_RUBIVIRUS_3362846      58  ---------------------------LREVARVERRWVALCLHRTAR----KLATALA-------------------------------   85
GremmeniellaXL1_ENDORNAVI      66  ----------------LNVHCVYTTSTPADLSRHTLWVKKATQWAHANLKNKSVHGATYS---------------------------SIA  112
CitrusTristeza_CLOSTEROVI      64  ----------------SGVHVCRPVYDVKDAHRRVVRHHQLSKVSLDQSDGVK-------------------------------------  100
GrapevineLeafroll3_AMPELO      65  ----------------VNCHVCNPVLDVKDVKRRINEILFLSTAGGDSYV----SSD--------------------------------L  102
OysterMushroom_28261418_r      66  ---------------GVQFNLVNEIITPADHTRYTD------------------------------------------------------   86
Tetnovirus2_302171585_gb_      53  ----------------TRYHYW-----ARDLMVAESNH----------------------------------------------------   69
Pariacoto_20260794_ref_NP      46  ----------------NRYFYF-----AKDLTIPYRND----------------------------------------------------   62
PierisRapae_63029695_gb_A      46  ----------------NRFFYC-----PKDFGMQFRND----------------------------------------------------   62
OliveLatent2_OLEAVIRUS_20      64  ----------------TGVHSCCPLLNPRDGAHQTRRELNMESYLRTHGPKY--------------------------------------   99
AppleStem_CAPILLOVIRUS_96      69  FS----------HGKIKSLGMYNAIIDGKDKYRYGDVEFSSFR-----------------------------------------------  101
GrapevineA_VITIVIRUS__275      67  N-------------RNLNFTQYNRLVHAKDHHRYENAFRELDV-----------------------------------------------   96
PotatoT_TEPOVIRUS_1943515      67  ----------------DSLETFNRLFSCKDALRYVDPETCDMDK----------------------------------------------   94
PotatoM_CARLAVIRUS_962609      67  ---------------LDSVQVVNRYVTSADRMRYTNDFVPYGS-----------------------------------------------   94
AppleStemPitting_FOVEAVIR      67  ---------------LQMVETINRYISSIDKTRYGGFFHVSPSK----------------------------------------------   95
AppleChlorotic_TRICHOVIRU      69  --------------SVKTYDILNRLVTAKDKARYGPLAKPE-------------------------------------------------   95
CitrusLeaf_CITRIVIRUS_208      68  SRRQVSDLNALNSLNNSHTSFINRLVASKDVSRYTEEADAFFQ-----------------------------------------------  110
ChronicBee_188572672_ref_      51  -------------------HLCNPCIDSSDILRDAKNPE---------------------------------------------------   70
Consensus_ss:                                        eeee        hhhhh
```

**βB	αC**	variable

```
Conservation:
HepatitisE_HEPEVIRUS_9626      96  LRGLPA------------------------------------------------------------------------------------  101
PlasmoparaA_301070430_gb_          ------------------------------------------------------------------------------------------
StripedJack_BETANODAVIRUS          ------------------------------------------------------------------------------------------
SclerophthoraA_45476494_r          ------------------------------------------------------------------------------------------
BatGuanoGF4n_297598937_gb          ------------------------------------------------------------------------------------------
Alphanoda_HB2007_29531469          ------------------------------------------------------------------------------------------
LakeSinai1_335057597_gb_A          ------------------------------------------------------------------------------------------
LakeSinai2_335057590_gb_A          ------------------------------------------------------------------------------------------
SclerotiniaL_190694814_gb     106  ---TGM------------------------------------------------------------------------------------  108
FlockHouse_351066202_gb_A          ------------------------------------------------------------------------------------------
BlackBeetle_51014189_ref_          ------------------------------------------------------------------------------------------
DrosophilaNodaSW2009_2622          ------------------------------------------------------------------------------------------
MacrobrachiumRosenbergii_          ------------------------------------------------------------------------------------------
Nodamura_ALPHANODAVIRUS_9          ------------------------------------------------------------------------------------------
Tetnovirus1_TETNOVIRUS_30          ------------------------------------------------------------------------------------------
BeetNecrotic_BENYVIRUS_23          ------------------------------------------------------------------------------------------
Helicoverpa_OMEGATETRAVIR     112  --ANGI------------------------------------------------------------------------------------  115
NudaureliaBeta_BETATETRAV          ------------------------------------------------------------------------------------------
CitrusLeprosisC_CILEVIRUS     117  -GFREG------------------------------------------------------------------------------------  121
BotrytisF_MYCOFLEXIVIRUS_          ------------------------------------------------------------------------------------------
Sindbis_ALPHAVIRUS_251215     111  LDTPDA------------------------------------------------------------------------------------  116
TurnipYellow_TYMOVIRUS_21          ------------------------------------------------------------------------------------------
GrapevineFleck_MACULAVIRU          ------------------------------------------------------------------------------------------
MaizeRayado_MARAFIVIRUS_1          ------------------------------------------------------------------------------------------
SclerotiniaDebilitation_S          ------------------------------------------------------------------------------------------
ShallotX_ALLEXIVIRUS_2052          ------------------------------------------------------------------------------------------
BotrytisX_BOTREXVIRUS_375          ------------------------------------------------------------------------------------------
LoliumLatent_LOLAVIRUS_16          ------------------------------------------------------------------------------------------
IndianCitrus_MANDARIVIRUS          ------------------------------------------------------------------------------------------
BambooMosaic_POTEXVIRUS_9          ------------------------------------------------------------------------------------------
PelargoniumZonate_ANULAVI     101  ---ESF------------------------------------------------------------------------------------  103
BromeMosaic_BROMOVIRUS_96          ------------------------------------------------------------------------------------------
CucumberMosaic_CUCUMOVIRU          ------------------------------------------------------------------------------------------
JapaneseHolly_PTERIDOVIRU     140  GMDCSMFCHRTFQSSFETVEDSSSVPFEDAVVSGSFAVSPWDVETQVGRRLAGGKFPPKKTFDDLADVAVNWVTEGVNSVVSSLKKERSG  229
RaspberryBushy_IDAEOVIRUS     140  AGIRDG------------------------------------------------------------------------------------  145
TobaccoStreak_ILARVIRUS_2          ------------------------------------------------------------------------------------------
Alfalfa_ALFAMOVIRUS_96269          ------------------------------------------------------------------------------------------
CuthroatTrout_FISHHEPEVIR     100  ---IQK------------------------------------------------------------------------------------  102
TobaccoRattle_TOBRAVIRUS_     116  RKNIAA------------------------------------------------------------------------------------  121
SoilWheat_FUROVIRUS_96323     128  AQPEDL------------------------------------------------------------------------------------  133
Potato_POMOVIRUS_20376948     128  YDANDL------------------------------------------------------------------------------------  133
IndianPeanut_PECLUVIRUS_3     126  AGKNVV------------------------------------------------------------------------------------  131
BarleyStripe_HORDEIVIRUS_     129  VNAGVL------------------------------------------------------------------------------------  134
TobaccoMosaic_Tobamovirus     113  DRYAEI------------------------------------------------------------------------------------  118
LettuceInfections_CRINIVI          ------------------------------------------------------------------------------------------
Rubella_RUBIVIRUS_3362846          ------------------------------------------------------------------------------------------
GremmeniellaXL1_ENDORNAVI     113  ASMIKE------------------------------------------------------------------------------------  118
CitrusTristeza_CLOSTEROVI     101  QVGWTV------------------------------------------------------------------------------------  106
GrapevineLeafroll3_AMPELO     103  LTEAAS------------------------------------------------------------------------------------  108
OysterMushroom_28261418_r          ------------------------------------------------------------------------------------------
Tetnovirus2_302171585_gb_          ------------------------------------------------------------------------------------------
Pariacoto_20260794_ref_NP          ------------------------------------------------------------------------------------------
PierisRapae_63029695_gb_A          ------------------------------------------------------------------------------------------
OliveLatent2_OLEAVIRUS_20     100  DKFNEL------------------------------------------------------------------------------------  105
AppleStem_CAPILLOVIRUS_96          ------------------------------------------------------------------------------------------
GrapevineA_VITIVIRUS__275          ------------------------------------------------------------------------------------------
PotatoT_TEPOVIRUS_1943515          ------------------------------------------------------------------------------------------
PotatoM_CARLAVIRUS_962609          ------------------------------------------------------------------------------------------
AppleStemPitting_FOVEAVIR          ------------------------------------------------------------------------------------------
AppleChlorotic_TRICHOVIRU          ------------------------------------------------------------------------------------------
CitrusLeaf_CITRIVIRUS_208     111  ---SKK------------------------------------------------------------------------------------  113
ChronicBee_188572672_ref_          ------------------------------------------------------------------------------------------
Consensus_ss:
```

variable

```
Conservation:
HepatitisE_HEPEVIRUS_9626     102  -----------------------ADRTYCFDG-------------FSGCNFPAET-----------------------------------  120
PlasmoparaA_301070430_gb_      68  ------------------------------------------------SLSSSSM-----------------------------------   74
StripedJack_BETANODAVIRUS      66  ------------------------------------------------EITDRHV-----------------------------------   72
SclerophthoraA_45476494_r      68  ------------------------------------------------RPGHRDI-----------------------------------   74
BatGuanoGF4n_297598937_gb      66  ------------------------------------------------DMRPTDI-----------------------------------   72
Alphanoda_HB2007_29531469      67  ------------------------------------------------PITSGNV-----------------------------------   73
LakeSinai1_335057597_gb_A      66  -----------------------HHGVLCRRQ-------------PSACRDRFEIP----------------------------------   85
LakeSinai2_335057590_gb_A      66  -----------------------QRFSACRRH-------------PSVCPDRFDTP----------------------------------   85
SclerotiniaL_190694814_gb     109  -----------------------PSGEFCPLS-------------GEDCSHKSSA-----------------------------------  127
FlockHouse_351066202_gb_A      66  ------------------------------------------------TPAEDAF-----------------------------------   72
BlackBeetle_51014189_ref_      66  ------------------------------------------------TPADDAF-----------------------------------   72
DrosophilaNodaSW2009_2622      66  ------------------------------------------------TPAEDAF-----------------------------------   72
MacrobrachiumRosenbergii_      67  ------------------------------------------------AVKENAV-----------------------------------   73
Nodamura_ALPHANODAVIRUS_9      66  ------------------------------------------------KIQDGDV-----------------------------------   72
Tetnovirus1_TETNOVIRUS_30      66  -------------------------------------------------LTDRNA-----------------------------------   71
BeetNecrotic_BENYVIRUS_23     104  -------------------------MESGERE-------------LMNNLKGCGYIVKRSVENAVYEVVSDKDVAEVLRYAQTVASTKKE  155
Helicoverpa_OMEGATETRAVIR     116  -----------------------ASRTFCVDG-------------VGSCAFKSRV-----------------------------------  134
NudaureliaBeta_BETATETRAV     111  -----------------------PTDTFCVDG-------------FSNCEYQSPL-----------------------------------  129
CitrusLeprosisC_CILEVIRUS     122  -----------------------STKYRCENI-------------GQQCYIEAPT-----------------------------------  140
BotrytisF_MYCOFLEXIVIRUS_      89  --------------------------TVVDH----------------PGSCTAGT-----------------------------------  101
Sindbis_ALPHAVIRUS_251215     117  -----------------------ETPSLCFHN-------------DVTCNMRAEY-----------------------------------  135
TurnipYellow_TYMOVIRUS_21      86  --------------------------TSPDL-------------------PVFPT-----------------------------------   95
GrapevineFleck_MACULAVIRU      93  --------------------------LPLPD-----------------TLPSTPL-----------------------------------  104
MaizeRayado_MARAFIVIRUS_1      86  --------------------------TSTNL-------------------PDCET-----------------------------------   95
SclerotiniaDebilitation_S      86  --------------------------FSFGL-----------------PSVATST-----------------------------------   97
ShallotX_ALLEXIVIRUS_2052      86  -------------------------THPNSC-----------------PVVNTEY-----------------------------------   98
BotrytisX_BOTREXVIRUS_375      88  -------------------------LDTAVV-----------------PPVHNRV-----------------------------------  100
LoliumLatent_LOLAVIRUS_16      88  -----------------------PDPDQDASI-------------TASFLEVKTST----------------------------------  107
IndianCitrus_MANDARIVIRUS      87  -------------------------NVQSTS-----------------TSIPTHT-----------------------------------   99
BambooMosaic_POTEXVIRUS_9      86  -------------------------TIYPRL-----------------PEITTKM-----------------------------------   98
PelargoniumZonate_ANULAVI     104  -----------------------ESPDFCLLK-------------AEDCEVQSPY-----------------------------------  122
BromeMosaic_BROMOVIRUS_96     103  -----------------------EVPNFCLNR-------------AQDCDVQADW-----------------------------------  121
CucumberMosaic_CUCUMOVIRU     108  -----------------------RQPDFCTQP-------------AADCKVQAHF-----------------------------------  126
JapaneseHolly_PTERIDOVIRU     230  DISCDSSCVEEGAPEGVSVASASVASDESVRR-------------TVSCGRRARF-----------------------------------  271
RaspberryBushy_IDAEOVIRUS     146  -----------------------NSSLYCHHK-------------FGVSWNDVLWESERNNCLTVP------------------------  175
TobaccoStreak_ILARVIRUS_2     102  -----------------------LPVDFCCHK-------------FEECDVKAPF-----------------------------------  120
Alfalfa_ALFAMOVIRUS_96269     103  -----------------------GEADYCMDT-------------FQKCSRRADY-----------------------------------  121
CuthroatTrout_FISHHEPEVIR     103  -----------------------EDATACLNG-------------YQGCHQPRAT-----------------------------------  121
TobaccoRattle_TOBRAVIRUS_     122  -----------------------PNAVRCNNT-------------YHGCTCRGFSDGKKK------------------------------  145
SoilWheat_FUROVIRUS_96323     134  -----------------------DGKWYCGNR-------------FEDCVFRADRA----------------------------------  153
Potato_POMOVIRUS_20376948     134  -----------------------NGTWFCQNK-------------FEDCVYDHSTLGDGK------------------------------  157
IndianPeanut_PECLUVIRUS_3     132  -----------------------MNDRYCCDV-------------FQNCAYEPDSGKL--------------------------------  153
BarleyStripe_HORDEIVIRUS_     135  -----------------------DGDVFCENT-------------FQDCVRRVPEGFL--------------------------------  156
TobaccoMosaic_Tobamovirus     119  -----------------------PEDAVCHNT-------------FQTMRHQPMQQSG--------------------------------  140
LettuceInfections_CRINIVI      97  -----------------------ETISFCTNK-------------TEDCAVNRDI-----------------------------------  115
Rubella_RUBIVIRUS_3362846      86  -----------------------ETAS---------------------EAWHADY-----------------------------------   96
GremmeniellaXL1_ENDORNAVI     119  -----------------------DNSMWCTNG-------------IANCSHAPISVG---------------------------------  139
CitrusTristeza_CLOSTEROVI     107  -----------------------NTNSVCGNI-------------LGECYHASEA-----------------------------------  125
GrapevineLeafroll3_AMPELO     109  -----------------------KSVSYCSRE-------------SQNCDSRADA-----------------------------------  127
OysterMushroom_28261418_r      87  --------------------------PSPP----------------PDFQGPGDM-----------------------------------   99
Tetnovirus2_302171585_gb_      70  ------------------------------------------------PLTHDSL-----------------------------------   76
Pariacoto_20260794_ref_NP      63  ------------------------------------------------PVSDNTG-----------------------------------   69
PierisRapae_63029695_gb_A      63  ------------------------------------------------PISNNHA-----------------------------------   69
OliveLatent2_OLEAVIRUS_20     106  -----------------------SRPDLCHHR-------------AEDCSVRAKA-----------------------------------  124
AppleStem_CAPILLOVIRUS_96     102  -----------------------DRVIGLRDQ-------------CLTRNKFPKV-----------------------------------  120
GrapevineA_VITIVIRUS__275      97  ---------------------------GNLTN-------------LINKESQSEC-----------------------------------  111
PotatoT_TEPOVIRUS_1943515      95  ---------------------------------------------FIARVHHSTR-----------------------------------  104
PotatoM_CARLAVIRUS_962609      95  -----------------------YEHECLVHKGVGLDNEALRGLVGPLRRHKAKN-----------------------------------  126
AppleStemPitting_FOVEAVIR      96  ----------------------ISAKFKCDRRTGFEDDASLIDLIPGCMEGARKR-----------------------------------  128
AppleChlorotic_TRICHOVIRU      96  ----------------------------------------------RSPCPKKTN-----------------------------------  104
CitrusLeaf_CITRIVIRUS_208     114  -----------------------GSPELFSRN-------------FIKSLENKEA-----------------------------------  132
ChronicBee_188572672_ref_      71  -----------------------PPFSSCGGR-------------GEVCPSRHDFP----------------------------------   90
Consensus_ss:                                                                            e
```

variable	**βC..**

```
Conservation:                                                                                  5                           5
HepatitisE_HEPEVIRUS_9626     121  --------------------------------------------------------GVALY------------------------SLHDM  130
PlasmoparaA_301070430_gb_      75  --------------------------------------------------------VSIID------------------------VDQYV   84
StripedJack_BETANODAVIRUS      73  --------------------------------------------------------IVMTD------------------------VDYYV   82
SclerophthoraA_45476494_r      75  --------------------------------------------------------RFLCD------------------------VDYYV   84
BatGuanoGF4n_297598937_gb      73  --------------------------------------------------------IRMVD------------------------VDYYV   82
Alphanoda_HB2007_29531469      74  --------------------------------------------------------IMMID------------------------VDYYV   83
LakeSinai1_335057597_gb_A      86  -------------------------------------------------------ACVISH------------------------ADYYM   96
LakeSinai2_335057590_gb_A      86  -------------------------------------------------------AAILSH------------------------VDYYM   96
SclerotiniaL_190694814_gb     128  --------------------------------------------------------LVSFF------------------------SLHDA  137
FlockHouse_351066202_gb_A      73  --------------------------------------------------------IVGVD------------------------VDYY-   81
BlackBeetle_51014189_ref_      73  --------------------------------------------------------IVGVD------------------------VDYY-   81
DrosophilaNodaSW2009_2622      73  --------------------------------------------------------IVGVD------------------------VDYY-   81
MacrobrachiumRosenbergii_      74  --------------------------------------------------------IVAID------------------------TDYY-   82
Nodamura_ALPHANODAVIRUS_9      73  --------------------------------------------------------VVAID------------------------IDYY-   81
Tetnovirus1_TETNOVIRUS_30      72  --------------------------------------------------------IILVD------------------------TDYYI   81
BeetNecrotic_BENYVIRUS_23     156  AKKKPNTGKRKMVMSEATRRTIELHELSRIVAEEKKIPNHFHFDESDFASVGNFTQLVCED------------------------VGYNF  221
Helicoverpa_OMEGATETRAVIR     135  --------------------------------------------------------GIANH------------------------SLYDV  144
NudaureliaBeta_BETATETRAV     130  --------------------------------------------------------AIACH------------------------SLYPD  139
CitrusLeprosisC_CILEVIRUS     141  --------------------------------------------------------LTFVH------------------------SAYDI  150
BotrytisF_MYCOFLEXIVIRUS_     102  --------------------------------------------------------LFMHD------------------------ALQYM  111
Sindbis_ALPHAVIRUS_251215     136  --------------------------------------------------------SVMQD------------------------VYIN-  144
TurnipYellow_TYMOVIRUS_21      96  --------------------------------------------------------IFMHD------------------------ALMYY  105
GrapevineFleck_MACULAVIRU     105  --------------------------------------------------------YFMHH------------------------SLMYF  114
MaizeRayado_MARAFIVIRUS_1      96  --------------------------------------------------------VFMHD------------------------ALMYY  105
SclerotiniaDebilitation_S      98  --------------------------------------------------------AFIGD------------------------ALHHM  107
ShallotX_ALLEXIVIRUS_2052      99  --------------------------------------------------------AVLAD------------------------TLHFM  108
BotrytisX_BOTREXVIRUS_375     101  --------------------------------------------------------ALLHD------------------------TLHFL  110
LoliumLatent_LOLAVIRUS_16     108  --------------------------------------------------------AFMQD------------------------TLHYL  117
IndianCitrus_MANDARIVIRUS     100  --------------------------------------------------------AYISD------------------------TLHFM  109
BambooMosaic_POTEXVIRUS_9      99  --------------------------------------------------------AFIGD------------------------SLHYF  108
PelargoniumZonate_ANULAVI     123  --------------------------------------------------------AISIH------------------------GAYDM  132
BromeMosaic_BROMOVIRUS_96     122  --------------------------------------------------------AICIH------------------------GGYDM  131
CucumberMosaic_CUCUMOVIRU     127  --------------------------------------------------------AISIH------------------------GGYDM  136
JapaneseHolly_PTERIDOVIRU     272  --------------------------------------------------------GIMIH------------------------SSYDM  281
RaspberryBushy_IDAEOVIRUS     176  ----------------------------------------------EPECPFKAKYAIMVH------------------------SGYDL  195
TobaccoStreak_ILARVIRUS_2     121  --------------------------------------------------------AMAIH------------------------SISDI  130
Alfalfa_ALFAMOVIRUS_96269     122  --------------------------------------------------------AFAIH------------------------STSDL  131
CuthroatTrout_FISHHEPEVIR     122  --------------------------------------------------------SIAIH------------------------STYDI  131
TobaccoRattle_TOBRAVIRUS_     146  ----------------------------------------------------GAQYAIALH------------------------SLYDF  159
SoilWheat_FUROVIRUS_96323     154  -------------------------------------------------------YAMAIH------------------------SIYDI  164
Potato_POMOVIRUS_20376948     158  ----------------------------------------------------KEAYGMAIH------------------------SIYDI  171
IndianPeanut_PECLUVIRUS_3     154  ------------------------------------------------------RVAMGVH------------------------SVYDM  165
BarleyStripe_HORDEIVIRUS_     157  ------------------------------------------------------KTAIAVH------------------------SIYDI  168
TobaccoMosaic_Tobamovirus     141  -----------------------------------------------------RVYAIALH------------------------SIYDI  153
LettuceInfections_CRINIVI     116  --------------------------------------------------------IIAV-------------------------EVYDM  124
Rubella_RUBIVIRUS_3362846      97  --------------------------------------------------------VCALRGAPSGPFYVHPEDVPHGGRAVADRCLLYY  130
GremmeniellaXL1_ENDORNAVI     140  -------------------------------------------------------FAMSID------------------------TLVHL  150
CitrusTristeza_CLOSTEROVI     126  ---------------------------------------------------------MVMV------------------------QVYDV  134
GrapevineLeafroll3_AMPELO     128  ---------------------------------------------------------GFMV------------------------DVYDI  136
OysterMushroom_28261418_r     100  --------------------------------------------------------ILVDD------------------------ALQHW  109
Tetnovirus2_302171585_gb_      77  --------------------------------------------------------LYLQD------------------------VDYYT   86
Pariacoto_20260794_ref_NP      70  --------------------------------------------------------FVFCD------------------------VDYYA   79
PierisRapae_63029695_gb_A      70  --------------------------------------------------------LVFTD------------------------VDYYA   79
OliveLatent2_OLEAVIRUS_20     125  --------------------------------------------------------ALSVD------------------------SAYDM  134
AppleStem_CAPILLOVIRUS_96     121  --------------------------------------------------------LFLHD------------------------ELHFL  130
GrapevineA_VITIVIRUS__275     112  --------------------------------------------------------IFIHD------------------------EVQYW  121
PotatoT_TEPOVIRUS_1943515     105  --------------------------------------------------------IFLFD------------------------ELHYW  114
PotatoM_CARLAVIRUS_962609     127  --------------------------------------------------------LFFHD------------------------ELHYW  136
AppleStemPitting_FOVEAVIR     129  --------------------------------------------------------FFFHD------------------------ELHYW  138
AppleChlorotic_TRICHOVIRU     105  --------------------------------------------------------IFIHD------------------------EIHYW  114
CitrusLeaf_CITRIVIRUS_208     133  --------------------------------------------------------VFFHD------------------------EVHHW  142
ChronicBee_188572672_ref_      91  -------------------------------------------------------GALLCH------------------------SDYY-  100
Consensus_ss:                                                                              eeeee
```

**..βC**	variable

```
Conservation:                                                  5
HepatitisE_HEPEVIRUS_9626     131  ----SPSDVAEAMFRH-GMTRLYAALHLP--PEVLLP---PGTYRTA-------------SYLLIHDGR---------------------  176
PlasmoparaA_301070430_gb_      85  ----DMNWFL---TEH-FCPVLLYTFQ-P--SVCAADR--------G-------------EYSYTFNSNN--------------------  122
StripedJack_BETANODAVIRUS      83  ----DMHELVGL-----GVPILLYTFQ-P--STVSGEV--------K-------------DGYFTITDD---------------------  118
SclerophthoraA_45476494_r      85  ----DMPAMLVAE----AKPVLLYTVV-P--EEATSSA------NDE-------------SAFYFEADG---------------------  123
BatGuanoGF4n_297598937_gb      83  ----DMHYWLGL-----GHPVLLYTFV-P--TRAGGKT--------F-------------DGSYSFSGN---------------------  118
Alphanoda_HB2007_29531469      84  ----DFNRYLQF-----GNPVMIYTFV-P--TSAGGPT--------T-------------DGTFSIEND---------------------  119
LakeSinai1_335057597_gb_A      97  ----SPDQLASTVT--------GPTFIIN--HDYLAPD--SLSVA---------------EATTRVANG---------------------  134
LakeSinai2_335057590_gb_A      97  ----TPDQLAAAVT--------GPTFIVN--HDYSSID--TLSVA---------------EVSLRSAGG---------------------  134
SclerotiniaL_190694814_gb     138  ----SPAVLDAMMARK-DALVAYVAMHLP--VALLATS--SFDDVAT-------------GAHCRRDDDT--------------------  185
FlockHouse_351066202_gb_A      82  ------VTEPDVLLEH-MRPVVLHTFN-P--KKVSGFD--A-------------------DSPFTIKNN---------------------  119
BlackBeetle_51014189_ref_      82  ------VTEPDVLLEH-MRPVVLHTFN-P--KKVSGFD--A-------------------DSPFTIKNN---------------------  119
DrosophilaNodaSW2009_2622      82  ------VTEPDVLLEH-MRPVVLHTFN-P--KKVSGFD--A-------------------DSPFTIKNN---------------------  119
MacrobrachiumRosenbergii_      83  ------LRDPSIYFSN-NNPFILHTFQ-P--ITVAGKD--G-------------------DVRFTISDN---------------------  120
Nodamura_ALPHANODAVIRUS_9      82  ------LRDIDRYLGR-GVPFMAYTFN-P--VEVAGRD--G-------------------DSFFRITNN---------------------  119
Tetnovirus1_TETNOVIRUS_30      82  ----DMPKLLTT-----GLPILMYTFV-P--QRLAGST--Q-------------------GTNYYIDKHS--------------------  118
BeetNecrotic_BENYVIRUS_23     222  ----SVDAWLHLFEAT-GAQTAVGYMALP--NELLFEH--YPISDYYDYWEGVEKHGSLGGITISPLRNG--------------------  282
Helicoverpa_OMEGATETRAVIR     145  ----TLEELANAFENH-GLHMVRAFMHMP--EELLYMD--NVVNAEL-------------GYRFHVIEEPMAVKDCAFQGGDLRLHFPEL  212
NudaureliaBeta_BETATETRAV     140  GESNSIMDVAKGMALH-GTHVIYAWMHLP--VELLTLT--DADNIFE-------------GYSIRFEETGALPC----------------  195
CitrusLeprosisC_CILEVIRUS     151  ----TAAGIVDCMIAA-NAHHAIMCLHFP--SAILSGS-TSGKDDLL-------------QYKWDIVV-EDG------------------  200
BotrytisF_MYCOFLEXIVIRUS_     112  ----TPLDVYTLFATSPEMHSLVATAVIP--PESVDRL--PAFWPE--------------LYQLAYYED---------------------  158
Sindbis_ALPHAVIRUS_251215     145  -----APGTIYHQAMK-GVRTLYW-IGFDTTQFMFSAM--AGSYPAY-------------NTNWADEKVLEARNIGLCSTKLSEGR----  208
TurnipYellow_TYMOVIRUS_21     106  ----HPSQIMDLFLRKPNLERLYASLVVP--PEAHLSD--QSFYPK--------------LYTYTTTRH---------------------  152
GrapevineFleck_MACULAVIRU     115  ----SPSQIAGLFLAAPFLERLYASLVLP--AESTIGS--HPFFPS--------------LYRYRTTGE---------------------  161
MaizeRayado_MARAFIVIRUS_1     106  ----TPGQIADLFFLCPQLQKIYASVVVP--AESSFTH--LSLHPE--------------IYRFRFQGS---------------------  152
SclerotiniaDebilitation_S     108  ----TPESVFDLFERSPNLMVLHATIVIP--PETLLKC--RSSNPE--------------LYSLRYYDD---------------------  154
ShallotX_ALLEXIVIRUS_2052     109  ----SPRQLYHLFSRNPKLERLFATLVLP--IEAQHRL--PSLFPD--------------VYRLEYYKD---------------------  155
BotrytisX_BOTREXVIRUS_375     111  ----SPRQLAQLFHHNPNLHLLYATLVLP--VEALHNL--PSLNPH--------------AYTLEYFSNG--------------------  158
LoliumLatent_LOLAVIRUS_16     118  ----TPEDLLDIFETSPKLENLIASFVLP--VEATRNM--KSLYPD--------------LYSIHYTHG---------------------  164
IndianCitrus_MANDARIVIRUS     110  ----DRKDLVRLFINSPNLDTLYATIVLP--VEAAYRQ--PSRYPD--------------LYQINYDFD---------------------  156
BambooMosaic_POTEXVIRUS_9     109  ----QPEVLEHIFTSSPQLETLLATIVLP--PEATLRL--KSLYPE--------------IYTLHYMPQ---------------------  155
PelargoniumZonate_ANULAVI     133  ----GFEGLCKAMHSH-GTIMLRGTMMFD--ANMLVFN--EGVMEDL-------------NCRWTKEKGDPYGLR---------------  185
BromeMosaic_BROMOVIRUS_96     132  ----GFQGLCDAMHSH-GVRVLRGTVMFD--GAMLFDR--EGFLPLL-------------KCHWQRDGSGA-------------------  180
CucumberMosaic_CUCUMOVIRU     137  ----GFRGLCEAMNAH-GTTILKGTMMFD--GAMMFDD--QGVIPEL-------------NCQWRKIRSAFSETEDVTPLVGK-------  197
JapaneseHolly_PTERIDOVIRU     282  ----LPVPVCQGMVEH-DMTMVKGCMIAE--PAMLVQT--KGYLPIL-------------ECNWRLVTPLGVPLTCHPCKLGDRFYR---  346
RaspberryBushy_IDAEOVIRUS     196  ----PLKELIGGMVQH-GVVELHGTMIAD--PAMLVAT--SGYIPAL-------------RCNWEKSKG---------------------  242
TobaccoStreak_ILARVIRUS_2     131  ----PISTVATHCVRR-GVRKLIASVMMD--PLMMLYD--KGHIPLL-------------NVDWEKEDVEET------------------  180
Alfalfa_ALFAMOVIRUS_96269     132  ----DVGELACSLDQK-GVMKFICTMMVD--ADMLIHN--EGEIPNF-------------NVRWEIDRKK--------------------  179
CuthroatTrout_FISHHEPEVIR     132  ----PLKEFCRGMNNH-KSDLAHVIMHMP--SEITFPP-GVFVNDMT-------------GYRLDTTGE---------------------  179
TobaccoRattle_TOBRAVIRUS_     160  ----KLKDLMATMVEK-KTKVGHAAMLFA--PESMLVD--EGPLPSV-------------DGYYMKKNG---------------------  206
SoilWheat_FUROVIRUS_96323     165  ----DLSDLANALEEK-RIKVMSGTFLFS--VDLLLGK-KSGTLPTM-------------DGFFEVEDG---------------------  212
Potato_POMOVIRUS_20376948     172  ----HLVDLVSAMERK-KVRVLKGTFLFS--ADIIIGK-KRGELPSV-------------NGFYIIDGE---------------------  219
IndianPeanut_PECLUVIRUS_3     166  ----TLQELVDGLERK-KIEHFIGCFLFS--PKLLLGQ-EEGELPFV-------------NGRFKVKKG---------------------  213
BarleyStripe_HORDEIVIRUS_     169  ----KVEEFASALKRK-GITQAYGCFLFP--PAVLIGQ-KEGILPSV-------------DGHYLVENG---------------------  216
TobaccoMosaic_Tobamovirus     154  ----PADEFGAALLRK-NVHTCYAAFHFS--ENLLLED-SYVNLDEI-------------NACFSRDGD---------------------  201
LettuceInfections_CRINIVI     125  ----TLRDMAKAMLSH-GSRKFEFSCIIP--PEIFTKE-CNVELYEG-------------RLKVTRIGD---------------------  172
Rubella_RUBIVIRUS_3362846     131  ----TPMQMCELMRTI-DATLLVAVDLWP--VALAAHV--GDDWDDL-------------GIAWHLDHDG--------------------  178
GremmeniellaXL1_ENDORNAVI     151  ----DPSDLIKFYIDN-NVAHASHAMTIP--DNYAYAT--KGVLRHN-------------EGHWYRTNN---------------------  197
CitrusTristeza_CLOSTEROVI     135  ----PLRELCRAMINK-KTSVCYMTMVTP--GELLDAR-ESFFIKDL-------------DCSVELDPIA--------------------  183
GrapevineLeafroll3_AMPELO     137  ----SPQQVAEAMDKK-GALVFDIALMFP--VELLYGN-GEVYLEEL-------------DTLVKREGD---------------------  184
OysterMushroom_28261418_r     110  ----SRERIDAAFRHF-GGNRLIGTNIHP--DEVRSGH--ASRYPD--------------LYTIEYLPNR--------------------  156
Tetnovirus2_302171585_gb_      87  ----DMNY----ILGA-GLPVVLYTII-P--RKAASRK--------Q-------------EYAYRFENG---------------------  122
Pariacoto_20260794_ref_NP      80  ----DMEK----WMQH-FKPMLLYTLV-P--ESLSYHC--------D-------------DHSFHVNDD---------------------  115
PierisRapae_63029695_gb_A      80  ----NMNR----WLQL-FLPTCLYTLV-P--TRLNYSN--------D-------------EFAYRFENN---------------------  115
OliveLatent2_OLEAVIRUS_20     135  ----GLKNTCKAMHRA-GIELLHGNILFD--PDMLIEAKMEGFVAGM-------------NYHWKKTRRSTGLMSSFLEMGSSLFGVSNS  204
AppleStem_CAPILLOVIRUS_96     131  ----SPFDMAFLFETIPEIDRVVATTVFP--IELLFGD--KVSKEPR-------------VYTYKVHGS---------------------  178
GrapevineA_VITIVIRUS__275     122  ----SLDEMQRFLGSLSKVDRIVYSIIYP--AEVEAGY--SQSLFPE-------------AYTFDLKEG---------------------  169
PotatoT_TEPOVIRUS_1943515     115  ----SMNSLSDFLDRS-NVKELLATIVFP--IEILLGS--KRSLNPE-------------LYEFEISRG---------------------  161
PotatoM_CARLAVIRUS_962609     137  ----SSKVLIDFLDVM-RPDKLLGTVVYP--PELLFKP--TRSLNEW-------------CYTYDIVGD---------------------  183
AppleStemPitting_FOVEAVIR     139  ----TKEALITFLDHV-KPEVMLASIVFP--PEILAGA--KESLNPW-------------CYTFRIVGK---------------------  185
AppleChlorotic_TRICHOVIRU     115  ----SRDQLETFLQVH-RPKNLWATLVFP--PEILAGY--KSSVLPF-------------LYQFEIHGK---------------------  161
CitrusLeaf_CITRIVIRUS_208     143  ----TKAQMFSFLKST-KVKRFIFTVVYP--PEILKKF--ANSQNPK-------------VYDFKVDKG---------------------  189
ChronicBee_188572672_ref_     101  ----LYDELSYVIR--------GHTFIIN--HSFLEEN--KHFHN---------------EAFITKKGD---------------------  138
Consensus_ss:                           hhhhhhhhhh     eeeeeee    hhhh                          eeeeee
```

**αD	βD**	variable	**βE**

```
Conservation:                                                                                              Y♦  6
HepatitisE_HEPEVIRUS_9626     177  --------------------------RVVVTYEG---------------------------------DTSAGY-NHDVSNL--  197
PlasmoparaA_301070430_gb_     123  --------------------------EVIYRVA-----------------------------------GGAEY-VHKVWNYDT  143
StripedJack_BETANODAVIRUS     119  --------------------------HVHYRVA-----------------------------------GGKDV-RHRIWNYNQ  139
SclerophthoraA_45476494_r     124  --------------------------SLRTMVA-----------------------------------GGGGY-SHCIWDYAA  144
BatGuanoGF4n_297598937_gb     119  --------------------------RVKYRVN-----------------------------------GGATY-EHQLWDYNH  139
Alphanoda_HB2007_29531469     120  --------------------------HIVYRVS-----------------------------------GGATY-RHEVWDHNH  140
LakeSinai1_335057597_gb_A     135  --------------------------HVTSSVR-----------------------------------EGPIYGPHPYYLWNN  156
LakeSinai2_335057590_gb_A     135  --------------------------LVTASVR-----------------------------------DGPTFGPHPYYRWSD  156
SclerotiniaL_190694814_gb     186  -------------------------GHWHMSFPG---------------------------------SGAAGY-THSLASL--  207
FlockHouse_351066202_gb_A     120  --------------------------LVEYKVS-----------------------------------GGAAW-VHPVWDWCE  140
BlackBeetle_51014189_ref_     120  --------------------------LVEYKVS-----------------------------------GGAAW-VHPVWDWCE  140
DrosophilaNodaSW2009_2622     120  --------------------------LVEYKVS-----------------------------------GGAAW-VHPVWDWCE  140
MacrobrachiumRosenbergii_     121  --------------------------QVDYRVD-----------------------------------GGGRW-QHKVWNWCD  141
Nodamura_ALPHANODAVIRUS_9     120  --------------------------QVTFDVS-----------------------------------GGGSW-SHEVWDWCA  140
Tetnovirus1_TETNOVIRUS_30     119  --------------------------QVHFRAA-----------------------------------GGTTW-NHYLWDYNH  139
BeetNecrotic_BENYVIRUS_23     283  --------------------------QVVGMPTGVFQPVHFDKTSAGLGIPGSKMGAAERVICHMSDGLGNGY-NHVKSDW--  336
Helicoverpa_OMEGATETRAVIR     213  D---FINESQERRIERLAARGSYSRRAVIFSGDD---------------------------------DWGDAY-LHDFHTW--  256
NudaureliaBeta_BETATETRAV     196  ----------------------TKRRKAIFSGYN---------------------------------DFGSAY-VHDAHHW--  220
CitrusLeprosisC_CILEVIRUS     201  ------------------------IKYYKQKFLN---------------------------------DTQASY-VHRLDVY--  223
BotrytisF_MYCOFLEXIVIRUS_     159  --------------------------HLCYAPDG---------------------------------NFADAY-NQPLAAH--  179
Sindbis_ALPHAVIRUS_251215     209  ----------TGKLSIMRKKELKPGSRVYFSVG------------------------------------STLY-PEHRASL--  242
TurnipYellow_TYMOVIRUS_21     153  --------------------------TLHYVPEG---------------------------------HEAGSY-NQPSDAH--  173
GrapevineFleck_MACULAVIRU     162  --------------------------HLHYVLEG---------------------------------NPSSSY-TQPLTAT--  182
MaizeRayado_MARAFIVIRUS_1     153  --------------------------DLVYEPEG---------------------------------NPAANY-TQPRSAL--  173
SclerotiniaDebilitation_S     155  --------------------------KFVYIPEG---------------------------------HAGGSY-VHEVKNS--  175
ShallotX_ALLEXIVIRUS_2052     156  --------------------------HFAYMPGG---------------------------------HGGGAY-VHSYGTL--  176
BotrytisX_BOTREXVIRUS_375     159  --------------------------DFAYMPGG---------------------------------HAGSGY-YHSAETL--  179
LoliumLatent_LOLAVIRUS_16     165  --------------------------GFQWAPSG---------------------------------HLGDAY-FHEPWQL--  185
IndianCitrus_MANDARIVIRUS     157  --------------------------GFQYIPGG---------------------------------HGGGAY-HHEFSHL--  177
BambooMosaic_POTEXVIRUS_9     156  --------------------------KFLYKPGG---------------------------------LSGGEY-EHDYKDL--  176
PelargoniumZonate_ANULAVI     186  --------------------GAPCEDMVHFDFID---------------------------------ESTLSY-SHSWKNI--  212
BromeMosaic_BROMOVIRUS_96     181  ------------------------DEVIKFDFEN---------------------------------ESTLSY-IHGWQDL--  203
CucumberMosaic_CUCUMOVIRU     198  ------------LNSTVFSRVRKFKTMVAFDFIN---------------------------------ESTMSY-VHDWENI--  232
JapaneseHolly_PTERIDOVIRU     347  ---------LSTTGDRPLSCPGFDDVFIEYHFQD---------------------------------DSTMAY-RHKFSTV--  384
RaspberryBushy_IDAEOVIRUS     243  --------------------------QIWFSFRD---------------------------------DSTMGY-RHDWEVY--  263
TobaccoStreak_ILARVIRUS_2     181  -----------------------GKTLIHFHFVD---------------------------------APGLSY-SHDFDTL--  204
Alfalfa_ALFAMOVIRUS_96269     180  -------------------------DLIHFDFID---------------------------------EPNLGY-SHRFSLL--  201
CuthroatTrout_FISHHEPEVIR     180  --------------------------TAKFTYLE---------------------------------DTSNGY-DHSKADW--  200
TobaccoRattle_TOBRAVIRUS_     207  --------------------------KIYFGFEK---------------------------------DPSFSY-IHDWEEY--  227
SoilWheat_FUROVIRUS_96323     213  --------------------------YVKYGFHN---------------------------------DTNPGY-KHNLNQL--  233
Potato_POMOVIRUS_20376948     220  --------------------------SIKYSFYD---------------------------------DPNCGY-EHNLNSL--  240
IndianPeanut_PECLUVIRUS_3     214  --------------------------KIRFFFLD---------------------------------DTTHGY-EHDLNDY--  234
BarleyStripe_HORDEIVIRUS_     217  --------------------------RIKFFFAN---------------------------------DPNAGY-SHDLKDY--  237
TobaccoMosaic_Tobamovirus     202  --------------------------KLTFSFAS---------------------------------ESTLNY-CHSYSNI--  222
LettuceInfections_CRINIVI     173  --------------------------NVEYYYG----------------------------------SNGETF-SHSCQTL--  192
Rubella_RUBIVIRUS_3362846     179  --------------------------GCPADCRGA------------------------------GAGPTPGY-TRPCTT---  201
GremmeniellaXL1_ENDORNAVI     198  --------------------------GTWVIEFV---------------------------------GESLSY-EQKVSYT--  218
CitrusTristeza_CLOSTEROVI     184  -------------------------DRVVYCFN------------------------------------NSAY-THTYSTI--  202
GrapevineLeafroll3_AMPELO     185  --------------------------YLAYNVG----------------------------------QCGEMY-EHSFSNV--  204
OysterMushroom_28261418_r     157  --------------------------RYGLLSHR---------------------------------HKSASY-EASIDEA--  177
Tetnovirus2_302171585_gb_     123  --------------------------QFLFTSN-----------------------------------GGTAF-SHLLWDYDL  143
Pariacoto_20260794_ref_NP     116  --------------------------RVFFDVR-----------------------------------GGASY-SHQLWDYTG  136
PierisRapae_63029695_gb_A     116  --------------------------EIVYNVT-----------------------------------GGGEY-RHSLWDYKG  136
OliveLatent2_OLEAVIRUS_20     205  SKEGEGHHDLKIKMGSSSTHMVPADWEISYHFRD---------------------------------DCVLGY-THNLADV--  251
AppleStem_CAPILLOVIRUS_96     179  --------------------------SFSFYPDG---------------------------------VASECY-EQNLANS--  199
GrapevineA_VITIVIRUS__275     170  --------------------------RLIWYPDG---------------------------------KAEGAY-TQPINPW--  190
PotatoT_TEPOVIRUS_1943515     162  --------------------------KLHFFPDG---------------------------------CTSESY-SQPKDC---  181
PotatoM_CARLAVIRUS_962609     184  --------------------------TLMFFPDG---------------------------------VQSEGY-QQPLKGG--  204
AppleStemPitting_FOVEAVIR     186  --------------------------DLVFFPDG---------------------------------EQSEAY-IQPVAGS--  206
AppleChlorotic_TRICHOVIRU     162  --------------------------DLVYMPDG---------------------------------VRSESY-TQPLENG--  182
CitrusLeaf_CITRIVIRUS_208     190  --------------------------RLFFFPDG---------------------------------VKTEAY-EQKLNME--  210
ChronicBee_188572672_ref_     139  --------------------------GVIMSTP-----------------------------------DGTEY-QHPYNVWKA  159
Consensus_ss:                                                eeeee                                         e eee
			            βF			   βG
```
