# Supplementary material for: Sequence analysis reveals a conserved extension in the capping enzyme of the alphavirus supergroup, and a homologous domain in nodaviruses
Source: Biol Direct. 2015 Apr 11;10:16. doi: 10.1186/s13062-015-0050-0 (PMC4392871; doi:10.1186/s13062-015-0050-0)
Supplement: Additional file 1: — Compilation of all supplementary figures and tables, in .zip format. [file 13062_2015_50_MOESM1_ESM.zip › Additional File 1/File S7 Alignments of the Core region of each group, in FASTA format.doc]

**File S7**. **Sequence alignment of the MTase‑GTase of selected representatives**. They were made using Psi-Coffee.

- Alto group: p1-12
- Tymo group: p13-16
- Alto and Tymo groups [= Alphavirus supergroup]: p17-25
- Noda group: p26-28
- Chropara group:p29
- Noda and Chropara groups [= Nodavirus supergroup]: p30-33
- Alphavirus and Nodavirus supergroups: p34-37

**Alto group**

>Sindbis_ALPHAVIRUS_251215/1-422 _R_ Sindbis_ALPHAVIRUS_251215_1-422.prf _R_ Sindbis_ALPHAVIRUS_251215_1-422.prf

MEKP--------------------------------------------------------------------

------------------------------------------------------------------------

------------------------------------------------------------------------

------------------------------------------------------------------------

------------------------------------------------------------------------

------------------------------------------------------------------------

------------------------------------------------------------------------

------------------------------------------------------------------------

------------------------------------------------------VVNVDVDPQSPFVVQLQK

SFPQ-FEV-VAQQV-TPNDHAN-ARAF-SHLASKLIELEVPT-------------TA--TILDIGSAPARR-

---MF-S--------EHQYHCVCPMRSPEDPDRMMKYASKL-AEKACKITNK-N--LHEK------------

---------------------------------------I-K---D-LRT--VL-DT-P-----------DA

ETPSLCFHN---------------------------------------------------------------

-------------------------------------------------DVTCNMR---------AEY----

------------------------------------------------------------------------

-----------SVMQ-DVYINA----PGTIYHQAM--KGVRTLY-WIGFDTTQFMFSAM--A-GS-Y-PAYN

TN--WADEKVLEARN---------------------------------------------------------

--------------------IGLCSTKLSEGRTGKLSIMRKKELKPGS--RVYFSV---GSTLYPEHRASL-

------------------Q-SWHLPSVFHLN---GKQSYTCRC--DTVVSCEGYVVKKITISPGI---T---

---------GETVGYAVTHN------------------------------S--EGFL-------LCK-V---

TD-T---VK------GERV------------SFPVCTYIPATICDQMTGIMAT---------------DI--

-SPDDAQKLLVG-LNQRIVINGRTN-RNTNTMQNYLLPIIAQGFSKWAKERKDDLDNE--------------

------------------------------KM------LGT-RERKLTYGCLW

>HepatitisE_HEPEVIRUS_9626/1-418 _R_ HepatitisE_HEPEVIRUS_9626_1-418.prf _R_ HepatitisE_HEPEVIRUS_9626_1-418.prf

MEAHQFIKAP--GI----------------------------------------------------------

------------------------------------------------------------------------

------------------------------------------------------------------------

------------------------------------------------------------------------

------------------------------------------------------------------------

------------------------------------------------------------------------

------------------------------------------------------------------------

------------------------------------------------------------------------

----------------------TTAIEQA--------ALAAANS------ALANAVVVRPFLSHQQIEILIN

LMQP-RQL-VFRPE-VFWNHPI-QRVI-HNELELYCR---A-----------R--SG--RCLEIGAHPRS--

--I--ND-N------PNVVHRCFLRPAGRDVQRWYTAPTRG-PAANC-----RR------------------

-----------------------------------------SAL---------R--G-L-----------PA

ADRTYCFDG---------------------------------------------------------------

-------------------------------------------------FSGCNFPA---------ET----

------------------------------------------------------------------------

-----------GVALYSLH-DM----SPSDVAEAMFRHGMTRLYAALHLPPEVLL--PP--G-TY---RTAS

YL--LIHD----------------------------------------------------------------

----------------------------------------------GR--RVVVTYEGDTSAGYNHDVSNL-

------------------R-SWIRTTKVT-G--D-H---PLVI--ERVRAIGCHFVLLLTAAPEPS-PT---

--------P-------YVPY-------------------------------PRSTEV-------YVR-S---

IF-G---PG------GTPSLFPTSCS-----TKSTFHAVPAHIWDRLMLFGATL-----------DDQAF--

-CCSRLMTYLRG-ISYKVTVGTLVA-NEGWNASEVALTAVITAAYLTICHQRYLRTQAISKGM-R--RLER-

EHAQKFIT-----------------------------------RLYSWLFEKS

>CuthroatTrout_FISHHEPEVIR/1-427 _R_ CuthroatTrout_FISHHEPEVIR_1-427.prf _R_ CuthroatTrout_FISHHEPEVIR_1-427.prf

MDLDCFAHRD--HDK---------------------------------------------------------

------------------------------------------------------------------------

------------------------------------------------------------------------

------------------------------------------------------------------------

------------------------------------------------------------------------

------------------------------------------------------------------------

------------------------------------------------------------------------

------------------------------------------------------------------------

-----------------VKLAISDCIE----------------KSA-G-AYVNTSHVITVVLTPEQLEQAKK

AFSP-QPV-TYQPR-VCLPHAL-AKHY-TTVLEGQLKNAI------------R--GR--PYIEAGPAP-HQ-

--I--NS-R------PKDTHFCLVRGDARNHVRFATAPNNGLSRKV----K---------------------

-----------------------------------------RIY---------Q--G-I-----------QK

EDATACLNG---------------------------------------------------------------

-------------------------------------------------YQGCHQPR---------AT----

------------------------------------------------------------------------

-----------SIAIHSTY-DI----PLKEFCRGMNNHKSDLAHVIMHMPSEITF--PP--G-VF-V-ND-M

TG--YR------------------------------------------------------------------

------------------------------------------LDTTGE--TAKFTYLEDTSNGYDHSKADW-

------------------L-EWTTVTALIV---DGF---TFHV--EVQRRLGSVVYLIIYRSTLPV-KIT--

----R---P-------LDPL-------------------------------CSTSFV-------FDP-Y---

GP-T------------------------------SGHTAPTSYVRSLHSYACGL-----------PDETF--

-SYTRLVIYAKG-LAFRAKIGAVAL-NEGWNPDTTAFSAVVAGVFMDVAVKRYLRSKGISNCM-K--ELKK-

EQNAGFWS------LLW-Q---RIC-----IWFG-DS-NEH-RHMDHNTLKGW

>SclerotiniaL_190694814_gb/1-474 _R_ SclerotiniaL_19_694814_gb_1-474.prf _R_ SclerotiniaL_19_694814_gb_1-474.prf

MIKQKQQPKPD-WQT--------------VPP----------------------------------------

-----------------------------------------KQ-----------------------------

--RH--------------------------------------------------------------------

------------------------------------------------------------------------

---------------------------------------------SPG--IKG-PQ--SRTTRPTRSSR---

--------------------SALSA-----------------------------------------------

------------------P----------------------I------------------------------

--------------LRL--------------------------------------------------DHQNA

--------------HRAPDHPVNVLIGEH--------LRGEVNDII-VASRSRWTINVAQPHTEAQVAALEA

SFPE-YNF-VSS-Y-AHAHHPS-TQAA-RHALELLTWKLV------------D--PA--HTTEIGPSYSSL-

--F-RRA-R------PAFAHYDMPLLTVRDGARRNLAMLSR-HKTLDERET---------------------

-----------------------------------------RAL---------DALR-T-----------GM

PSGEFCPLS---------------------------------------------------------------

-------------------------------------------------GEDCSHKS---------SA----

------------------------------------------------------------------------

-----------LVSFFSLH-DA----SPAVLDAMMARKDALVAYVAMHLPVALLA--TS--S-FD-D-VATG

AH--CR------------------------------------------------------------------

------------------------------------------RDDDTG--HWHMSFPGSGAAGYTHSLASL-

------------------S-AWSAG----W---KGL---NLTC--EIVRQFGTIVHMRVVRTFGPS-TEN--

-----TP-I-------SIGN-------------------------------LLSSLV-------VIP-P---

FK-S---TA------HES-------E-----AFTTDAGKYGALVDRLVL------------------EDLTD

APVKKALARIAA-LSPAIRVGSRVQ-SAAWDIPYLKRNPVAVAAVRAALVLSADIEF-CENLA-A-------

----NDIA------PIR-------A-----ALVE-R------LAATMPLLPAL

>NudaureliaBeta_BETATETRAV/1-448 _R_ NudaureliaBeta_BETATETRAV_1-448.prf _R_ NudaureliaBeta_BETATETRAV_1-448.prf

MEDASKQLRV--------------------------------------------------------------

------------------------------------------------------------------------

------------------------------------------------------------------------

------------------------------------------------------------------------

------------------------------------------------------------------------

------------------------------------------------------------------------

------------------------------------------------------------------------

------------------------------------------------------------------------

--------------------------------------------LD-AQERAKAAFQLDFIASVETLEDAQE

KYEG-MMF-RSGTK-LPSTHIKLAIDL-RVAEKD-LRRHVK-----------N--VP--TVLEIGPSVESVR

YAVQT-R-D------KERVHGCT-FSDARDNLRHNKIGYEA-H-YDRKIGPD-A------------------

------------------------------------------AL-----------LA-A-----------GI

PTDTFCVDG---------------------------------------------------------------

-------------------------------------------------FSNCEYQS---------PL----

------------------------------------------------------------------------

-----------AIACHSLY-PDGESNSIMDVAKGMALHGTHVIYAWMHLPVELLT--LT--DADNIF-EGYS

IR--FEET------------------GAL--------------------PCT---KR---------------

-----------------------------------------------R--KAIFSGYNDFGSAYVHDAHHW-

------------------A-GWLKHRGVDTP--YGF---SILI--DIQQRFGMHTKLKITRGHSSG-SIT--

----T-----------VFPL-------------------------------SKLGLI-------WVP-N---

IV-K---I-----MYPKA-------K-----HEPEYIVTDKKKYEGVCVYVGTRVQ-------S-SGKSI--

-TLAEIVQYIRT-RLTRIILNGTVH-EKTWTIAEQDIERLAVSIMFRKNVERAVSEKALMRAQ-K--KCKS-

AEKQALLP------VWM-R---RIA-----NWFQ-DKFQID-EEVVRKRYLEC

>CitrusTristeza_CLOSTEROVI/1-466 _R_ CitrusTristeza_CLOSTEROVI_1-466.prf _R_ CitrusTristeza_CLOSTEROVI_1-466.prf

GEDNSLIITD---------------------T----------------------------------------

------------------------------------------------------------------------

------------------------------------------------------------------------

------------------------------------------------------------------------

------------------------------------------------------------------------

------------------------------------------------------------------------

------------------------------------------------------------------------

---------------------------------------------DRLR--------------AVGSAYEKV

--K-----------RNIPDSLLVRSVEKD--------LIEFNQTLV-DMHRSRPGVVVPFQMSENQQVMLTR

AYPE-FNI-NFIHS-VHSDHPV-AAGS-RALENHLVRKH-A--------------GT--DYSDVGGCPLFH-

--L-R-A-G------HSGVHVCRPVYDVKDAHRRVVRHHQL-SKVSLDQSDG-V------------------

------------------------------------------KQ-----------VG-W-----------TV

NTNSVCGNI---------------------------------------------------------------

-------------------------------------------------LGECYHAS---------EA----

------------------------------------------------------------------------

-----------MV-MVQVY-DV----PLRELCRAMINKKTSVCYMTMVTPGELLD--AR--ESFF-I-KDLD

CS--VEL-------------------------------------------DP---IA---------------

-----------------------------------------------D--RVVYCF---NNSAYTHTYSTI-

------------------C-ECMRTPCLVV---DGF---LFTI--EMVSLRCSVNYYCITKSSVCP-RIS--

----ETK-R-----LRYRRCD------------------------------SDLIRI-------KIP-R---

YS-S---K-----T-RSC-------L-----PGCYYLYLDAKFVSRVYEYVVNNCV-------V--VNAK--

-TFEWTWNYIKS-CKSRVVISGKII-HRDEPIALEYLDGFSAVMLSAGVKGRQNADVSRRLAA-F--SGE--

---TSIFE------LVG-F---AIS-----EKCR-DLFLGI-QDAVERCVKSY

>GrapevineLeafroll3_AMPELO/1-854 _R_ GrapevineLeafroll3_AMPELO_1-854.prf _R_ GrapevineLeafroll3_AMPELO_1-854.prf

MDYIRPLRVF--S-FPHVNNTLEYVRYNKANGDVGAFLTTMKFIGNVKL-SDFTPRCA--------AMIY--

--------------------------------------------IGKLTKGVKRTFVPPPVKGFARQYAVVS

GSVSA-LRG------------------------------------------DGKKVLMEARTSTSATSDVSD

FDVVFEAVSNALLVVHYHRVVPYAPVKREQPKPAVKQDEQKPKRQASHWAVKPTAVGVHVPLPKKQEALEPA

QSVPQQSLEEKAALTFGLFFSKGGGDES--------DA------VILRKGKLF----NR-ALNVPIDVKNTF

VWAKIWDEASRRRGYFYVKDRAVKFFPIVRGRATIEDFIVNTAPGCDVALPRIELWSMRERAFVCTTKGWCW

FNNERLRGEIYRRRCFSSSFSIGFLMHLGFRSLKVIRFAGTN---------------ILHMPSLN-------

---EERTFGWKGGDVYLPNVPKTAIVAGDRTRLGGEILASVANALNQEE--VYSS--------VVSSITNRL

--------------VLRDQSALLSHLDTK--------LCDMFSQRD-AMIREKPSHRCDVFLKPREREKLRE

LFPE-LSI-QFSDS-VRSSHPF-ANAM-RSCFNGIFSRR-C-----------G--NV--CFFDIGGSFTYH-

--V-K-A-G------HVNCHVCNPVLDVKDVKRRINEILFL-STAGGDSYVS-S------------------

-----------------------------------------DLL-----------TE-A-----------AS

KSVSYCSRE---------------------------------------------------------------

-------------------------------------------------SQNCDSRA---------DA----

------------------------------------------------------------------------

-----------GF-MVDVY-DI----SPQQVAEAMDKKGALVFDIALMFPVELLY--GN--GEVY-L-EELD

TL--VKR------------------------------------------------EG---------------

-----------------------------------------------D--YLAYNV-GQCGEMYEHSFSNV-

------------------S-GFFTFSYVRTS--SGN---VFKL--EYEGYRCGYHHLTMCRAQKSP-GTE--

----VTY-R------SLVPSF------------------------------VGKSLV-------FIP-V---

VA-G---S-----S-----------------VSFKTIVLDSDFVDRIYSYALNTIG-------T--FENR--

-TFEYAVGAVRS-QKTHVITGSRVV-HSKVDISPDDMWGLVVAVMAQAIKDRAKSIRSYNFIK----ASE--

---GSLAG-------VF-K---LFF-----QTV-----GDC-FSNAVSVYAKA

>LettuceInfections_CRINIVI/1-451 _R_ LettuceInfections_CRINIVI_1-451.prf _R_ LettuceInfections_CRINIVI_1-451.prf

ASNEAVQRED--FRD-LDL-----------------------------------------------------

------------------------------------------------------------------------

------------------------------------------------------------------------

------------------------------------------------------------------------

------------------------------------------------------------------------

------------------------------------------------------------------------

------------------------------------------------------------------------

-------------------------------------------------------RVEE----FAERVLETA

--------------NLRSDNRLIDNILTR--------ASDYINK----KSKESKELDINVCLSMDEKKMITN

LFPD-IQM-SFNQK-SYSNHGV-FNAM-RACENFYFSRKF-K-------------NS--DYIDAGGDVVST-

---LR-S-K------NHNVHICSPRLDLKDAARHIQRATVI-DGLKG-------------------------

----------------------------------------------------------------------YG

ETISFCTNK---------------------------------------------------------------

-------------------------------------------------TEDCAVN---------RDI----

------------------------------------------------------------------------

-----------IIAV-EVY-DM----TLRDMAKAMLSHGSRKFEFSCIIPPEIFT--KE--C--N-V-ELYE

GRLKVTRI----------------------------------------------------------------

----------------------------------------------GD--NVEYYY-GSNGETFSHSCQTL-

------------------K-DILSVQVFQFG---GR---VFKK--TLEHSRGQLHFFSICICEKIE-PG---

---------SVKLKTYYQRS------------------------------ELDKVTL-------RIP-V---

KD-S---F---------GV------------VTHYIIKEDREFVSSMIEYVANTGI---------KIDDK--

-MVEWTYSQYRA-KKTVTIKSGKVT-QKETRIRKELIPGFIAIIMSEGIRAREKTHYL-AKML-Y-T-S---

HYKPSIVN---II----FR---LIM-----HFL-----GGT-KRFIYESLVDC

>TobaccoStreak_ILARVIRUS_2/1-493 _R_ TobaccoStreak_ILARVIRUS_2_1-493.prf _R_ TobaccoStreak_ILARVIRUS_2_1-493.prf

MDSLSLPTVC--DVV--------------V------------------------------------------

------------------------------------------------------------------------

------------------------------------------------------------------------

------------------------------------------------------------------------

------------------------------------------------------------------------

------------------------------------------------------------------------

------------------------------------------------------------------PAL---

-------------------------------------------------------NVDN----LIRDYVSNV

--------------RADDSNNVSRFLGEV--------ALKEIKSQVDTSNGDFQKLNVGFRLTPDEKNALKA

NFPG-LEI-AFRDS-CHSSHSF-AAAH-RVCETLNIYNRFKT----------K--TE--RIIDLGGNYVTH-

--A-K-Q-G------RSNVHSCCPILDVRDGARHTDRYISL-AASVENRHR---------------------

-----------------------------------------------------------------------E

LPVDFCCHK---------------------------------------------------------------

-------------------------------------------------FEECDVKA---------PF----

------------------------------------------------------------------------

-----------AMAIHSIS-DI----PISTVATHCVRRGVRKLIASVMMDPLMML--YD--K-GH-I-PLLN

VD--WEKEDVE-------------------------------------------------------------

-------------------------------------------ETGKT--LIHFHFVDAPGLSYSHDFDTL-

------------------S-QYMITNQVIVN--NSY---SFRV--ERTACLSGVYIVEMTLSMTDG-HSLAY

LKPMRDV-S-----CAWLSSL------------------------------RKKVFV-------KLA-V---

PISA---EWY------TE-------Q-----FEVRHALMDESLVRYVSEAAFRQFS-------KTKDPET--

-LVQYIATMLSS-SSNHVVINGITM-RSGSPIKFDEYVPLAVTFYVMAAWRYKMIAPGIDAVK-T---RT--

EKNVDILDEKGLIKEDF-NVVNALL-----EDAG--L-IEP-KLPHFTDVIKN

>Alfalfa_ALFAMOVIRUS_96269/1-477 _R_ Alfalfa_ALFAMOVIRUS_96269_1-477.prf _R_ Alfalfa_ALFAMOVIRUS_96269_1-477.prf

MNADAQSTDA--S-----------------------------------------------------------

------------------------------------------------------------------------

------------------------------------------------------------------------

------------------------------------------------------------------------

------------------------------------------------------------------------

------------------------------------------------------------------------

------------------------------------------------------------------------

---------------------------------------------LSMREPLSHASIQE----MLRRVVEKQ

--------------AADDTTAIGKVFSEA--------GRAYAQDAL-P-SDKGEVLKISFSLDATQQNILRA

NFPG-RRT-VFSNS-SSSSHCF-AAAH-RLLETDFVYRCFGN----------T--VD--SIIDLGGNFVSH-

--M-K-V-K------RHNVHCCCPILDARDGARLTERILSL-KSYVRKHP----------------------

----------------------------------------------------------E-----------IV

GEADYCMDT---------------------------------------------------------------

-------------------------------------------------FQKCSRRA---------DY----

------------------------------------------------------------------------

-----------AFAIHSTS-DL----DVGELACSLDQKGVMKFICTMMVDADMLI--HN--E-GE-I-PNFN

VR--WEI-------------------------------------------DR---KK---------------

-----------------------------------------------D--LIHFDFIDEPNLGYSHRFSLL-

------------------K-HYLTYNAVDL---GHA---AYRI--ERKQDFGGVMVIDLTYSLGFV-PKM--

-PHSNGR-S-----CAWYNRV------------------------------KGQMVV-------HTV-N---

EGYY---H-----H-SYQ-------T-----AVRRKVLVDKKVLTRVTEVAFRQFR-------PNADAHS--

-AIQSIATMLSS-STNHTIIGGVTL-ISGKPLSPDDYIPVATTIYYRVKKLYNAIPEMLSLLD-K--GER--

---LSTDA-------VL-K---GSE-----GPM--WYSGPT-FLSALDKVNVP

>BromeMosaic_BROMOVIRUS_96/1-447 _R_ BromeMosaic_BROMOVIRUS_96_1-447.prf _R_ BromeMosaic_BROMOVIRUS_96_1-447.prf

MSSSI-DLLK--LIA-EKGA----------------------------------------------------

--------------DSQ-------------------------------------------------------

------------------------------------------------------------------------

------------------------------------------------------------------------

------------------------------------------------------------------------

------------------------------------------------------------------------

------------------------------------------------------------------------

------------------------------------------------------------------------

----SAQDI-VDNQ-----------------------VAQQLSAQI-EYAKRSKKINVRNKLSIEEADAFRD

RYGGAFDL-NLTQQ-YHAPHSL-AGAL-RVAEHYDCLDSFP-----------P--ED--PVIDFGGSWWHH-

--F-S-R-R------DKRVHSCCPVLGVRDAARHEERMCRM-RKILQE------------------------

---------------------------------------S-------------D-DF--------------D

EVPNFCLNR---------------------------------------------------------------

-------------------------------------------------AQDCDVQA---------DW----

------------------------------------------------------------------------

-----------AICIHGGY-DM----GFQGLCDAMHSHGVRVLRGTVMFDGAMLF--DR--E-GF-L-PLLK

CH--WQRDGS--------------------------------------------------------------

--------------------------------------------GADE--VIKFDFENESTLSYIHGWQDL-

------------------G-SFFTESVHCI---DGT---TYLL--EREMLKCNIMTYKIIATNLR-CPRE--

----TLR-H-----CVWFEDI------------------------------SKYVGV-------SIP-E---

DW---------------S-------L-----NRWKCVRVAKTTVREVEEIAFRCFKE-------SKEWTE--

-NMKAVASILSA-KSSTVIINGQAI-MAGERLDIEDYHLVAFALTLNLYQKYEKLTALRDGM-EW--KG---

-WCHHFKT------RFW-W---------------GGDSSRA-KVGWLRTLASR

>PelargoniumZonate_ANULAVI/1-454 _R_ PelargoniumZonate_ANULAVI_1-454.prf _R_ PelargoniumZonate_ANULAVI_1-454.prf

MAATSFNVRD--LIN-SNGA----------------------------------------------------

--------------DAM-------------------------------------------------------

------------------------------------------------------------------------

------------------------------------------------------------------------

------------------------------------------------------------------------

------------------------------------------------------------------------

------------------------------------------------------------------------

------------------------------------------------------------------------

----GVRGL-VDAH-----------------------ATKAAEEQF-EYIKRSKKVWVRQILSASDGEKMQK

RFGGTFDL-QL-SK-NLCPHSF-AGAM-RQCETLECLSSFP-----------E--DS--LILDFGGSWLFH-

--W-Q-R--------QHNVHSCCPVLDARDMARHQERMISM-QKCVAHR-----------------------

---------------------------------------P-------------G-KF-E-----------SF

ESPDFCLLK---------------------------------------------------------------

-------------------------------------------------AEDCEVQS---------PY----

------------------------------------------------------------------------

-----------AISIHGAY-DM----GFEGLCKAMHSHGTIMLRGTMMFDANMLV--FN--E-GV-M-EDLN

CR--WTKEKGDPYG----------------------------------------------------------

-------------------LR---------------------GAPCED--MVHFDFIDESTLSYSHSWKNI-

------------------K-SFLTEGGYQI---GNV---QYVL--ERCVISYGIMSFKIFAVSGK-IPHT--

----RLR-H-----CVWFPKV------------------------------RDYVNI-------NPS-----

-----------------D-------P-----RIWSKVRVKLDTVREVEEICFRCPKD-----VSKIEVMG--

-GESETCGIMSVLYSSTIIVNGMTM-MAGERLDVLDYHHVAFSLMLSARRKFDMFGKAMNSL-EW--KG---

-WVSHFFK------SLW-P------------------SGDL-RDLFGRYFPSL

>CucumberMosaic_CUCUMOVIRU/1-471 _R_ CucumberMosaic_CUCUMOVIRU_1-471.prf _R_ CucumberMosaic_CUCUMOVIRU_1-471.prf

MATSSFNINE--LVA-SHGD----------------------------------------------------

--------------KGL-------------------------------------------------------

------------------------------------------------------------------------

------------------------------------------------------------------------

------------------------------------------------------------------------

------------------------------------------------------------------------

------------------------------------------------------------------------

------------------------------------------------------------------------

----LATAL-VDKT-----------------------AHEQLEEQL-QHQRRGRKVYIRNVLGVKDSEVIRN

RYGGKYDL-HLTQQ-EFAPHGL-AGAL-RLCETLDCLDSFPSSG-------LR--QD--LVLDFGGSWVTH-

--Y-L-R--------GHNVHCCSPCLGIRDKMRHAERLMNM-RKIILND-----------------------

---------------------------------------P-------------Q-QF-D------------G

RQPDFCTQP---------------------------------------------------------------

-------------------------------------------------AADCKVQA---------HF----

------------------------------------------------------------------------

-----------AISIHGGY-DM----GFRGLCEAMNAHGTTILKGTMMFDGAMMF--DD--Q-GV-I-PELN

CQ--WRKIRSAFSET---------------------------------------------------------

----EDVTPLVGKLNSTVFSR---------------------VRKFKT--MVAFDFINESTMSYVHDWENI-

------------------K-SFLTDQTYSY---RGM---TYGI--ERCVIHAGIMTYKIIGVPGM-CPPE--

----LIR-H-----CIWFPSI------------------------------KDYVGL-------KIP-A---

SQ---------------D-------L-----VEWKTVRILTSTLRETEEIAMRCYND-------KKAWME--

-QFKVILGVLSA-KSSTIVINGMSM-QSGERIDINDYHYIGFAILLHTKMKYEQLGKMYDMW-NA-------

----SSIS------KWF-A----------------ALTRPL-RVFFSSVVHAL

>OliveLatent2_OLEAVIRUS_20/1-423 _R_ OliveLatent2_OLEAVIRUS_2__1-423.prf _R_ OliveLatent2_OLEAVIRUS_2__1-423.prf

MDSAALEKMT--GAK---------------------------------------------------------

---------------FP-------------------------------------------------------

------------------------------------------------------------------------

------------------------------------------------------------------------

------------------------------------------------------------------------

------------------------------------------------------------------------

------------------------------------------------------------------------

------------------------------------------------------------------------

----AVQGV-IEEF-----------------------SRDRVQNVL-GDMRSRQVIKYAVGLSEASVQELRF

NWPC-FTW-EEESV-PLPPHPF-AAYS-RRAFTRWAIAQCG--------------PV--PIKDFGGNWFLH-

--W-Q-W--------QTGVHSCCPLLNPRDGAHQTRRELNM-ESYLRTH-----------------------

---------------------------------------G-PKY---------D-KF-N-----------EL

SRPDLCHHR---------------------------------------------------------------

-------------------------------------------------AEDCSVRA---------KA----

------------------------------------------------------------------------

-----------ALSVDSAY-DM----GLKNTCKAMHRAGIELLHGNILFDPDMLI--EAKME-GF-V-AGMN

YH--WKKTRRSTGLM-SSFLEMGSS-LFG--------------------VSNSSKEG---------------

----EGHHDLKIKM-GSSSTH---------------------MVPADW--EISYHFRDDCVLGYTHNLADV-

------------------L-SIATGSYVKV---GNT---FYEL--ERTGLKSGMLMYTITACKGL-YD----

------------------------------------------------------------------------

------------------------------------------------------------------------

-RASARSTPLSA-KASTVIINGMSY-QVGEKLDPISFPYLAASFYMQAQKAVFEVQQVVDLH-TP-------

------NR------NLW-S----------------WFKKKF-E-LKAHAFLFA

>BarleyStripe_HORDEIVIRUS_/1-502 _R_ BarleyStripe_HORDEIVIRUS__1-5_2.prf _R_ BarleyStripe_HORDEIVIRUS__1-5_2.prf

MASDEIVR-N--LI----------------------------------------------------------

------------------------------------------------------------------------

------------------------------------------------------------------------

------------------------------------------------------------------------

------------------------------------------------------------------------

------------------------------------------------------------------------

------------------------------------------------------------------------

-------------------------------------------------------SREE----VMGNLISTA

--------------SSSVRSPLHDVLCSH--------VRTIVDSVD-KKAVSRKHEDVRRNISSEELQMLIN

AYPE-YAV-SSSAC-ESGTHSM-AACF-RFLETEYLLDMVPM----------K--ET--FVYDIGGNWFSH-

---MK-FRA------DREIHCCCPILSMRDSERLETRMMAM-QKYMRGSKDK-PLRLLSRYQNILRE-----

---------------------------------------QAARTTAFMAG--EV-NA-G-----------VL

DGDVFCENT---------------------------------------------------------------

-------------------------------------------------FQDCVRRVPEG----FLKT----

------------------------------------------------------------------------

-----------AIAVHSIY-DI----KVEEFASALKRKGITQAYGCFLFPPAVLI--GQK-E-GI-L-PSVD

GH--YLVE----------------------------------------------------------------

----------------------------------------------NG--RIKFFFANDPNAGYSHDLKDY-

------------------L-KYVEKTYVDI---EDG---VFAI--ELMQMRGDTMFFKITDVTAAM-YHM--

----------K----Y-RGM-------------------KRDETFKCIPLL-KNSSV-------VVP-L---

FS-W---DN------R--S------------LKITSGLLPRTLVEQGAAFIMKN-----------KEKDL--

-NVAVLKNYLSA-VNNSYIFNGSQV-RDGVKIAPDLISKLAVTLYLREKVYRQRENSIISYFE-Q-E-M---

LHDPNLKA---MF----GD---FLW-----FVPN-TL-SSV-WKNMRKSLMEW

>IndianPeanut_PECLUVIRUS_3/1-507 _R_ IndianPeanut_PECLUVIRUS_3_1-5_7.prf _R_ IndianPeanut_PECLUVIRUS_3_1-5_7.prf

MAQSNIYS-D--MID-RYV-----------------------------------------------------

------------------------------------------------------------------------

------------------------------------------------------------------------

------------------------------------------------------------------------

------------------------------------------------------------------------

------------------------------------------------------------------------

------------------------------------------------------------------------

-------------------------------------------------------NDET----VVQNLFMTA

--------------SSTTKSLLHDVIADR--------AANHLKSLE-KKQKQKELVDVRRVLSSESLNALCS

LYPE-FNV-ITSNR-ERGSHTM-AAVC-RTLETLYIKSLLPV----------N--DT--VVWDVGGNWLTH-

---VK-YST------NQDVHCCCPLLDYRDAMRKQERILSL-ELFARNGKEK-TEEFDACYQQIRNF-----

---------------------------------------ENTRRTAIS-----A-GK-N-----------VV

MNDRYCCDV---------------------------------------------------------------

-------------------------------------------------FQNCAYEPDSG----KLRV----

------------------------------------------------------------------------

-----------AMGVHSVY-DM----TLQELVDGLERKKIEHFIGCFLFSPKLLL--GQE-E-GE-L-PFVN

GR--FKVK----------------------------------------------------------------

----------------------------------------------KG--KIRFFFLDDTTHGYEHDLNDY-

------------------L-EYIRKSFVVAK--CGH---VYML--ELFSMRGDTVFFKLYDVTEYS-LSK--

----------TGFLST-LLP-------------------TRDHVFKAMPIP-NTDEV-------IVP-L---

YW-I---EN------G--E------------VVVERRYLPKSLVCRGMEWLMRN-----------KANAL--

-QYETLLNYLVS-TNVSAVFNGCQV-REGLKTDPWILCKLAMTLLVREEFNREKQKKVLEMLR-L-S-G---

SEQISLKR---IF----KG---VFE-----KFFG-SV--SL-KARVLRTVARW

>Potato_POMOVIRUS_20376948/1-501 _R_ Potato_POMOVIRUS_2_376948_1-5_1.prf _R_ Potato_POMOVIRUS_2_376948_1-5_1.prf

MDQSILS-----KI----------------------------------------------------------

------------------------------------------------------------------------

------------------------------------------------------------------------

------------------------------------------------------------------------

------------------------------------------------------------------------

------------------------------------------------------------------------

------------------------------------------------------------------------

-------------------------------------------------------TSDD----YVNAILHTS

--------------ATRVGSDLHNAMCNV--------IVDQIKDST-EKNKKKKKIDVKRNLNEDQIQLLAE

LFPE-RRV-VTSSV-HRGTHSM-AAAM-RKIETDVIFTSFPK-------------NG--VIYDIGGNWATH-

---AK-RDD------GGFVHCCCPILDFRDAQRKMTRLIDF-NRFIDDAKVV-SAEKAAVAAQIKKD-----

---------------------------------------CDLISENAKKD--AY-DA-N-----------DL

NGTWFCQNK---------------------------------------------------------------

-------------------------------------------------FEDCVYDHSTLGDGKKEAY----

------------------------------------------------------------------------

-----------GMAIHSIY-DI----HLVDLVSAMERKKVRVLKGTFLFSADIII--GKK-R-GE-L-PSVN

GF--YIID----------------------------------------------------------------

----------------------------------------------GE--SIKYSFYDDPNCGYEHNLNSL-

------------------M-LYVTKTFVKAA--GGA---VYYL--ELTEMRGDTMFFTITDASEAR-VMG--

-------------------V-------------------LHDSSTKCLPLN-KRDLV-------VFP-L---

FD-I---DR------ATDE------------LVFREELLSREFVNRALEYAFQL-----------KDNQV--

-TAEGLISYFAS-TNNAVVIGGSAR-KTSEKVDPKLLPMITTTLMVYQELQKAKQKRVLGKLK-S-K-V---

KEELTLSG---IL----ES---VVH-----RVFG-RQ--SL-YQRGLGVFAKW

>SoilWheat_FUROVIRUS_96323/1-497 _R_ SoilWheat_FUROVIRUS_96323_1-497.prf _R_ SoilWheat_FUROVIRUS_96323_1-497.prf

MPIDSSSILG--II----------------------------------------------------------

------------------------------------------------------------------------

------------------------------------------------------------------------

------------------------------------------------------------------------

------------------------------------------------------------------------

------------------------------------------------------------------------

------------------------------------------------------------------------

-------------------------------------------------------SEED----VIRAAISTS

--------------ATKFGSQLHSTVCDH--------VKETYLDAV-ERQKVKKKIDVRRDLSQEQLQLLND

LYPE-RHI-VSSNC-ERGTHSF-AAAS-RKIETDLLLSRIPK-------------RS--WVYDIGGNWATH-

---VK-RND------GRKVHCCCPTVDIRDSARKTVRWASI-EKYLDEKEEI-PPEIGERIKRLQAD-----

---------------------------------------EDRIYANLKSE--KA-QP-E-----------DL

DGKWYCGNR---------------------------------------------------------------

-------------------------------------------------FEDCVFRA-------DRAY----

------------------------------------------------------------------------

-----------AMAIHSIY-DI----DLSDLANALEEKRIKVMSGTFLFSVDLLL--GKK-S-GT-L-PTMD

GF--FEVE----------------------------------------------------------------

----------------------------------------------DG--YVKYGFHNDTNPGYKHNLNQL-

------------------M-KYLTKTFVVAK--GGT---IYYL--ELTEQRGDVMFFTMTDATEAR-MNG--

-------------------V-------------------VADESFKCIPID-NKDEV-------VFP-I---

FE-V---DQ------KTDA------------LVFSEILLSRDFVQRAIEYTGRL-----------KPAQL--

-TSDNVNTYLTS-TNNTIIIGGSSK-KNATKVDATLIQQITTTLIVWTELMNQRQKRVLEKLR-M-Q-M---

KDDVDFMT---LA----HA---TFL-----KMFG-KV--SY-YQRALRCFANW

>TobaccoRattle_TOBRAVIRUS_/1-498 _R_ TobaccoRattle_TOBRAVIRUS__1-498.prf _R_ TobaccoRattle_TOBRAVIRUS__1-498.prf

MANGNFKLSQ--LLN-VDE-----------------------------------------------------

------------------------------------------------------------------------

------------------------------------------------------------------------

------------------------------------------------------------------------

------------------------------------------------------------------------

------------------------------------------------------------------------

------------------------------------------------------------------------

-------------------------------------------------------MSAE----QRSHFFDLM

--------------LTKPDCEIGQMMQRV--------VVDKVDDMI-RERKTKDPVIVHEVLSQKEQNKLME

IYPE-FNI-VFKDD-KNMVHGF-AAAE-RKLQALLLLDRVPA-------------LQ--EVDDIGGQWSFW-

---VT-R-G------EKRIHSCCPNLDIRDDQREISRQIFL-TAIGDQARSG-KRQMSEN------------

---------------------------------------ELW---M-YDQ--FR-KN-I-----------AA

PNAVRCNNT---------------------------------------------------------------

-------------------------------------------------YHGCTCRGFSDGKKKGAQY----

------------------------------------------------------------------------

-----------AIALHSLY-DF----KLKDLMATMVEKKTKVGHAAMLFAPESML--VD--E-GP-L-PSVD

GY--YMKK----------------------------------------------------------------

----------------------------------------------NG--KIYFGFEKDPSFSYIHDWEEY-

------------------K-KYLLGKPVSYQ---GN---VFYF--EPWQVRGDTMLFSIYRIAGVP-RR---

---------SLSSQEYYRRI----------------------------YISRWENMV-------VVP-I---

FD-L---VE------STRE------------LVKKDLFVEKQFMDKCLDYIARL-----------SDQQL--

-TISNVKSYLSS-NNWVLFINGAAV-KNKQSVDSRDLQLLAQTLLVKEQVARPVMRELREAIL-T-E-T---

KPITSLTD---VLGLISRK---MWK-----QFA-----NKIAVGGFVGMVGTL

>TobaccoMosaic_TOBAMOVIRUS/1-507 _R_ TobaccoMosaic_Tobamovirus_1-5_7.prf _R_ TobaccoMosaic_TOBAMOVIRUS_1-5_7.prf

MAYTQTATTS--AL----------------------------------------------------------

------------------------------------------------------------------------

------------------------------------------------------------------------

------------------------------------------------------------------------

------------------------------------------------------------------------

------------------------------------------------------------------------

------------------------------------------------------------------------

---------------------------------------------------------------------LDT

--------------VRGNNSLVNDLAKRR--------LYDTAVEEF-NARDRRPKVNFSKVISEEQTLIATR

AYPE-FQI-TFYNT-QNAVHSL-AGGL-RSLELEYLMMQIPY-------------GS--LTYDIGGNFASH-

---LF-K-G------RAYVHCCMPNLDVRDIMRHEGQKDSI-ELYLSRLERG-GKTVPNF------------

---------------------------------------Q-------KEA--FD-RY-A-----------EI

PEDAVCHNT---------------------------------------------------------------

-------------------------------------------------FQTMRHQPMQ---QSGRVY----

------------------------------------------------------------------------

-----------AIALHSIY-DI----PADEFGAALLRKNVHTCYAAFHFSENLLL--EDS-Y-VN-L-DEIN

AC--FSRD----------------------------------------------------------------

----------------------------------------------GD--KLTFSFASESTLNYCHSYSNI-

------------------L-KYVCKTYFPAS--NRE---VYMK--EFLVTRVNTWFCKFSRIDTFL-LYK--

----GVAHKSVDSEQFYTAMEDAWHYKKTLAMCNSERILLEDSSSVNYWFPKMRDMV-------IVP-L---

FD-I---SL------ETSK------------RTRKEVLVSKDFVFTVLNHIRTY-----------QAKAL--

-TYANVLSFVES-IRSRVIINGVTA-RSEWDVDKSLLQSLSMTFYLHTKLAVLKDDLLISKFS-L-G-S---

---KTVCQ---HV----WD---EIS-----LAF-----GNAFPSVKERLLNRK

>NegevNGEVIRUS|AFI24684.1|_1-550/1-550 hypothetical protein 1 [Negev virus] _R_ NegevNGEVIRUS_AFI24684.1__1-55__1-55_.prf

MSSLQDKFG---LAS-SATATI-----------------------SAS---------SSDVLPPSAA---TG

--------------KSLG----IDDFSHNC-----------------------------------------V

LDQLRQNTALDYEDISK-------------------------------------------------------

------------------------------------------------------------------------

------------------------------------------------------------------------

------------------------------------------------------------------------

------------------------------------------------------------------------

--------------------------------------------------------------NVNSNILKQL

LTSPQTQEIIVSAM-----------------------LN-DTVDAL-NAKNRLTKVPVYQRLSKEDQDRLRK

TFCM-FNL-DFSKAYDCAGHPF-WRAY-RTLSERKMLRQAGITPGS-RPAKGY--DA--VYKDVGGNPVTH-

--L-N-R-G------ECYCHTCMPLLSNNDDKRHSAYKERL-RRYSTHK-----------------------

---------------------------------------HSPCF---------N-LH-L-----------SQ

DSRVICNRM---------------------------------------------------------------

-------------------------------------------------SQNCSVKS---------EV----

------------------------------------------------------------------------

-----------LIFLHSTY-DM----TLSDIADAMHRADARTAYGVMHFNPCVLY--EK--K-GK-LLNGMN

FE--KRVVNGR-------------------------------------------------------------

-----------------------------------------------M--RIRFWYTNDSQEGYEHDFLNY-

------------------V-ALLRTFRIASTSKDTR---YYNV--QFDTTDDDTAFFVIRQSISGTIPRS--

----HPF-R-----VFTNEAL------------------------------ADKMIVYSWHWDTINP-G---

NI-S---SS------VIS-------R-----MRPQRIIVPRKLFYKMCAFADT-----------LPDSKF--

-TVKNILIAGTS-FNTREVISGQSI-GVVDPIEPAELKMLAVCIYVLTYISNYECTKSLSTMIADEDRVRE-

ESSSGFFS------RLF-R---LHIRLPWNHRV-QKFQQKM-DRLIDEMKSFE

>CitrusLeprosisC_CILEVIRUS/1-577 _R_ CitrusLeprosisC_CILEVIRUS_1-577.prf _R_ CitrusLeprosisC_CILEVIRUS_1-577.prf

MSTHSTVETL--DVN-RVRELLRSHREASSHCDEPRFTA-THTKGRVV---------ASSEKPPVSFTIANG

QIVSDSEVITSTEAKAAAILSVISSYPKE-------------------------------------------

--------------IRE-------------------------------------------------------

------------------------------------------------------------------------

------------------------------------------------------------------------

------------------------------------------------------------------------

------------------------------------------------------------------------

--------------------------------------------------------------QLNGRIERGF

TDNPDVDEA-ARCI-----------------------HSARLHNIT-TTALRKKPLLVHENVSNDMERFLNE

KFLG-YKI-RLTFS-KNVAHNN-AAAL-RRVLRFYMRDKVGYRKDDDIPD-GY--HV--KNKDVGASGMDV-

--I-A-D-E------LTDVHCCTPDLDFRDHIRLERLKKYI-YSHVCPTSKD--------------------

---------------------------------------HGIIC---------E-GF-R-----------EG

STKYRCENI---------------------------------------------------------------

-------------------------------------------------GQQCYIEA---------PT----

------------------------------------------------------------------------

-----------LTFVHSAY-DI----TAAGIVDCMIAANAHHAIMCLHFPSAILS--GST-S-GK-D-DLLQ

YK--WDIVVED-------------------------------------------------------------

---------------------------------------------GIK--YYKQKFLNDTQASYVHRLDVY-

------------------LDKFLTKVVLGS---DKR---FYMF--EITEMFGSVAIIEVFRREKDFIAGS--

----KLT-F-----NIPRTEH------------------------------PNTVTIHTWEYVTGYESL---

-F-KGRNGL------KSG-------V-----MRPVSIEVPEEFFLSVFKYGMT-----------VDSNKF--

-VYDGLLKSGVG-IAARRNIGGTTMVDPSFPIPVRKLQTFIVTMYMLLYQEKWEATQGLVTMKMLADQY---

-RTRSSKN------GIV-R---FLT-----NVFSPDKVGET-QHPSSTYDLGK

>GremmeniellaXL1_ENDORNAVI/1-658 _R_ GremmeniellaXL1_ENDORNAVI_1-658.prf _R_ GremmeniellaXL1_ENDORNAVI_1-658.prf

MHTNTYNTTN--PIN-SPNAAM------------------------------------NNINKRKA------

----------------GL----YKSTANKNHSRNNECTEPKG---------I-----RPSFKDWGSI-KKVS

RTKIVKGTSDSKVEIKNKVMATNSCWNSIFGYDLGVDAMIDIDMIKMSLIGQ--------------------

------------------------------------------------------------------------

--------------------------EE--------DAEHIRLHVTMRKGILHFE---N-PIGLPT------

------------------------------------------------GDPSPELFA-KQREFVKTYK----

------------------------------------------------------------------------

-----------GETVGLL----------------------------------------DILNKHDGNTLVG-

----LSEEPLLDRIAKDAGISIPQITTSQFKTEMDKLVN-TRLQHA-ATNHATKVILLPTSTPVDIVEDLNN

LHGS-GSF-VRGNM-AAHSHIY-HHAN-RLMLTEMLASRHP-----------R--NA--LLYDIGGNINRH-

--L-D-H-G------RLNVHCVYTTSTPADLSRHTLWVKKA-TQWAHANLKN-K------------S-----

----------------------------------VHGATYSSIA---------A-SM-I-----------KE

DNSMWCTNG---------------------------------------------------------------

-------------------------------------------------IANCSHAPI------SVGF----

------------------------------------------------------------------------

-----------AMSIDTLV-HL----DPSDLIKFYIDNNVAHASHAMTIPDNYAY--AT--K-GV-L-RHNE

GH--WYRTN-N-------------------------------------------------------------

-----------------------------------------------G--TWVIEFVGE-SLSYEQKVSYT-

------------------D-AYLQTLIFTA---GEM---AVYN--KVVGRKGPHMLFEHFLITRAKLESE--

----CIK-H-----VTWFNMN------------------------------MDEIFV-------MIP-E---

ID-F---DS------AVT-------LMKREPFTMKTVSINLRFYERLLNRLL---------------QEY--

-SWEATMAYASS-MIGKVYATSSGL-HLKWNMTNAEVRDHCLVAYWSTNRVNESMRPLIQQA-ER--NN---

-RDPDFLT------GLW-T---AVK-----NWG-RDFGSHF-DPSGSELIQTF

>RaspberryBushy_IDAEOVIRUS/1-655 _R_ RaspberryBushy_IDAEOVIRUS_1-655.prf _R_ RaspberryBushy_IDAEOVIRUS_1-655.prf

MECLDFSKLW--FST--------------AAG-----------LQQRCYYDCV----A--------WECL--

---------------------------------------GDDDLQI-----------------FISGLNRLI

ESVAVSCTG---------------------------------------------------------------

------------------------------------------------------------------------

-------------------------DEDLDFVVDSCNE------FVTGRDLKS----FF-AADLPV------

----------------------------------------------------RE--VSS-------------

---------------------------------------VGC---------------ISHFIPGSVSGLNVS

DLLDNQLYGCSV----------------------------------------FSSDFES----KLRDIRDAA

--------------LSDAASGVSQLVSCH--------FEKDVRHLLA-ENANSVKIPVPQKLSDDDMRILRD

HFPR-YEL-KFTQN-VDGPHNM-AAAH-RLLETHDLLSNFP-----------A--DA--PILDIGGNWFSH-

--F-R-Y-G------RSNVHSCCPMLDLRDNERHTHRLTMT-ESLMSSLRHR-Y------------AGTIDL

DPDAHLSRKVSDSMKEF---------YK---RWAVHPKDLIRLY-----------AGIR-----------DG

NSSLYCHHKFGVSWN---------------------------------------------------------

----------------DV------------------LWESERNNCLTVPEPECPFKA---------KY----

------------------------------------------------------------------------

-----------AIMVHSGY-DL----PLKELIGGMVQHGVVELHGTMIADPAMLV--AT--S-GY-I-PALR

CN--WEKS----------------------------------------------------------------

----------------------------------------------KG--QIWFSFRDDSTMGYRHDWEVY-

------------------S-KYLTSTVVS-C--GKH---FYVM--ERDKYRHGVLFYSIIKCSGSL-RKG--

--DHTFF-H-----NAWFHEM------------------------------YDKYIM-------KVP-L---

VK-V---KDLTGDE-GSV-------E-----CSWREVVMSRKLVDRVIEVCLRGVKPINFGNCDDAVHMD--

-NLRIIQNHLLS-HSQTLVLNGSTI-IREEAIPFKDFSPVSVTIYFEILLTRYKESLSLAWFH-A---GL--

GPDFK---LGSWVS--------SLK-----RVFY-RI-LGF-PANLLKYVLNA

>Helicoverpa_OMEGATETRAVIR/1-467 _R_ Helicoverpa_OMEGATETRAVIR_1-467.prf _R_ Helicoverpa_OMEGATETRAVIR_1-467.prf

MYAKATDVAR--VY----------------------------------------------------------

------------------------------------------------------------------------

------------------------------------------------------------------------

------------------------------------------------------------------------

------------------------------------------------------------------------

------------------------------------------------------------------------

------------------------------------------------------------------------

------------------------------------------------------------------------

----------------------AAAD--V--------AYANV--------LQQRAVKLDFAPPLKALETLHR

LYYP-LRF-KGGTL-PPTQHPI-LAGH-QRVAEEVLHNFAR-----------G--RS--TVLEIGPSLHSA-

--L--KL-HGAPNAPVADYHGCT-KYGTRDGSRHITALESR-SVATGRPEFKAD------------------

-----------------------------------------ASL---------L--A-N-----------GI

ASRTFCVDG---------------------------------------------------------------

-------------------------------------------------VGSCAFKS---------RV----

------------------------------------------------------------------------

-----------GIANHSLY-DV----TLEELANAFENHGLHMVRAFMHMPEELLY--MD--N-VV-N-AELG

YR--FHVIEEPMAVKDC-AFQGGDLRLHFPELDFINESQERRIE----------------------------

----------------RLAAR---------------------GSYSRR--AVIFSGDDDWGDAYLHDFHTW-

------------------L-AYLLVRNYPTP--FGF---SLHI--EVQRRHGSSIELRITRAPPGD-RML--

--------A-------VVPR-------------------------------TSQGLC-------RIP-N---

IF-Y---YA------DAS-------G-----TEHKTILTSQHKVNMLLNFMQTR-----------PEKEL--

VDMTVLMSFARA-RLRAIVVASEVT-ESSWNISPADLVRTVVSLYVLHIIERRRAAVAVKTAK-D--DVFG-

--ETSFWE-----------------------------------S-LKHVLGSC

>JapaneseHolly_PTERIDOVIRU/1-789 _R_ JapaneseHolly_PTERIDOVIRU_1-789.prf _R_ JapaneseHolly_PTERIDOVIRU_1-789.prf

MDKLLSLFTIPSVFL----SA--------LTD-----------TGGFCYLNCF----D--------VSLY--

---------------------------------------PGRIFDI-----------------LAAQFAMLS

TAMSV-KRH------------------------------------------VGEY-----------------

------------------------------------------------------------------------

------------------KFLLT-GKE--------LKE------IVIRHGNMR----IR-SFRVV-------

----------------------------------------------------Y---STS-------------

---------------------------------------ERC---------------VCHIDKSTGALIDAA

WLNDNDLFGADVPQFNLGSLPQ-------------------VKAVDYLE--PGSLQFEQ----VRGSVIRRA

--------------VADDDSNVSASLRAD--------YLEKLRQAEL-VRQSLRKVRVVRSISPDQLATLRS

LFPG-FEF-EYTPR-YDGPHNM-AAVI-RTAETLEAVLSFP-----------K--DE--PLIDIGGDFFFH-

--V-R-N-G------FTHVHCCAPILDSRDDERFSKRCMHL-QKWVIDKQVS-A------------------

--YSSMSRRVKATLKEIPNLELLCDRLREFNDACRTPD-------------------DN-----------GM

DCSMFCHRTFQSSFETVEDSSSVPFEDAVVSGSFAVSPWDVETQVGRRLAGGKFPPKKTFDDLADVAVNWVT

EGVNSVVSSLKKERSGDISCDSSCVEEGAPEGVSVASASVASDESV-RRTVSCGRRA---------RF----

------------------------------------------------------------------------

-----------GIMIHSSY-DM----LPVPVCQGMVEHDMTMVKGCMIAEPAMLV--QT--K-GY-L-PILE

CN--WRLVTPLGVPLTCHPCKLGDR-FYR--------------------LST---TGDRPLSC---------

------------------------------------------PGFDDV--FIEYHFQDDSTMAYRHKFSTV-

------------------F-EWMTCHIVE-C--DGA---RFCL--ERDKFRDGSLYFTITRLRFPS-LRS--

---ELNF-S-----CAWLSSM------------------------------ASHYVL-------KVP-M---

FR-S---FRYKGF---LR-------H-----SYFDEYVVSKTLVDTVFSACLRSLYT-----FEPKDHRK--

-NFQVVITYLAS-HSQSIIINGTAV-TRREPLPAEWLNPIAMAIYYQLCVVKDDESQALLNID-N--CQI--

TKIFH---FSNFVS--------ALK-----DIVT-SF-LK---FVLPSKLFEK

>Rubella_RUBIVIRUS_3362846/1-445 _R_ Rubella_RUBIVIRUS_3362846_1-445.prf _R_ Rubella_RUBIVIRUS_3362846_1-445.prf

MEKLLDEVLA--P-----------------------------------------------------------

------------------------------------------------------------------------

------------------------------------------------------------------------

------------------------------------------------------------------------

------------------------------------------------------------------------

------------------------------------------------------------------------

------------------------------------------------------------------------

------------------------------------------------------------------------

--------------GGPYNLTVGSWVRDH--------VRSIVEG----------AWEVRDVVTAAQKRAIVA

VIPR-P---VFTQM-QVSDHPA-LHAISRYTRRH--------------------------WIEWGPKEALH-

---VL-I-D------------PSPGL-LREVARVERRWVAL-CLHR-T--AR---KLA--------------

------------------------------------------------TALAET-AS-EAWHADYVCALRGA

PSGPFYVHP---------------------------------------------------------------

--------------------------------------------------EDVPHG---------GRA----

------------------------------------------------------------------------

-----------VADR-CLL-YY----TPMQMCELMRTIDATLLVAVDLWPVALAA--HV--G-DD-W-DDLG

IAWHLDHD----------------------------------------------------------------

----------------------------------------------GGCPADCRGAGAGPTPGYTRPCTTRI

YQVLPDTAHPGRLYRCGPR-LWTRDCAV-------A---ELSW--EVAQHCGHQARVRAVRCTLP-------

----------I----RHVRS------------------------------LQPSARV-------RLP-D---

LV-H---L--------AEV------------GRWRWFSLPRPVFQRMLSYCKTL-----------SPDAY--

-YSERVFKFKNA-LCHSITLAGNVL-QEGWKGTCAEEDALCAYVAFRAWQSNARLAGIMKGAK-R-C-AADS

LSVAGWLD---TI----WD---AIK-----RFL-----GS---VPLAERMEEW

>BeetNecrotic_BENYVIRUS_23/1-723 _R_ BeetNecrotic_BENYVIRUS_23_1-723.prf _R_ BeetNecrotic_BENYVIRUS_23_1-723.prf

MADSFGFTPME-VLL--------------FGG----------------------------------------

------------------------------------------------------------------------

------------------------------------------------------------------------

------------------------------------------------------------------------

--------------------------------------------------E----SVQLLTSDMPIDVQ---

-----WGFVHSTRCYALWKDDLIHLNPLLKYSQRIAKRWERLVSGFVGPVPLDKLLSLLAK-----------

----------LMRYCVNMGV---------------------SVQEIYLSDAIVSSSYMLHVSRSAGC-VSFS

WLYAKLSMFASCGKFWVGSSHH-------------------TAANMI--------------------EGSRA

--------------VNGPDVAISEM-----------------VEAF-H-LEVKSSLVVTVSLTPREKKILER

ELGF-VPLYKQKSR-APRNHPV-LAAL-REVMRQEYSASC------------NILNTKLKTLVVGAASREV-

--N-CYS-S------NPSVHYYFANKDSKDLVRTTLELLHS-ALATKYR-----------------------

----------------------------------------------------------N-----------ME

SGERELMNN---------------------------------------------------------------

-------------------------------------------------LKGCGYIV---------KRSVEN

AVYEVVSDKDVAEVLRYAQTVASTKKEAKKKPNTGKRKMVMSEATRRTIELHELSRIVAEEKKIPNHFHFDE

SDFASVGNFTQLVCEDVGY-NF----SVDAWLHLFEATGAQTAVGYMALPNELLF--EH--Y-PI-S-DY-Y

DY--WEGVEKH-----------------------------GSLGGITISPLR---NGQVVGMPTGVFQPVHF

DKTSA---------GLGIPGS---------------------KMGAAE--RVICHMSDGLGNGYNHVKSDW-

------------------Q-TLLKHPILSS---SKY---NFAVEVDLTGRYGCLATFRLTRVTGVK-YVA--

----RTI-K-------LRPE---------------------------------DRYV-------RVLDLLHI

VR-S---IRLKG-H-AGL-------K-----EPYQYFPVYKREVDTPVSYCFSI-----------AEKSL--

-TVQNIANFIRH-HIGGVSLVNKEL-VSAWRLNPQLVPSFAYAVYFYVVNLRGELDGMLQKLM-K--KG---

---ITWAD------RLK-A---NVS-----AFLR-D------MVDPISFLWTW

**Tymo group**

>BambooMosaic_POTEXVIRUS_9/1-410

MALVSKVFDSI--TDPSLRAVLQEEAHSQVKQVLKDTALY---SRYALLPTAANTLERYAIPHNPF--STKL

HTHAAAKALENDLYRTASYLLPK--ERVSFLFMKPSKMQYFRRRG-D-----------------VDTFINAD

IVAKDLARYPLETI------------YP------------------------------RLPEITTKMAFIGD

SLHYFQPEVLEHIFTSSPQLETLLATIVLPPEATLRL-KSLYPEIYTLHYMP-QKFLYKPGGLSGGEYEHDY

KDLTWL-KIGHVTTAS----------------------------MCLTVDRVESKAANHLLLIRRGRL----

-------------------KLSTYRSYDTPEPLVVVPKI-FLP--PKYNAQKP-ITKTKANSWILYVKSAGE

PKIRDVWAKLRQTIANS-ELNQYEPAELLLLTNYFYVLGKLD---------SITSFETLLGDNILKR-LFRP

AIAKI--QELRHWVSG---PTAF-----MQLYKALQLVDV---DFTFEVTKNWETKQ

>IndianCitrus_MANDARIVIRUS/1-417

MATIRGAIDRI--TDTTVRTTLQEEACRQIRTELKNVEHV---NRYAIPPDAADALEHLGIGTNPF--SVKL

HTHGACKAIENQLLYVVGTLLPK--ERVTMLFLKKAKLNFMKRCP-K----------------FQDIFLNQH

IEPRDVSRYCDFNV------------QS------------------------------TSTSIPTHTAYISD

TLHFMDRKDLVRLFINSPNLDTLYATIVLPVEAAYRQ-PSRYPDLYQINYDF-DGFQYIPGGHGGGAYHHEF

SHLEWL-DVGHIHWRGPDN---------------------KDIQLTITAQMIESLGANHLFCFRRGNL----

-------------------RTPRVRTFGRDTQ-VLLPKI-FRP--VDKNFNRA-IPLTLANKMLLYAKSINT

VTFRDVVAKTRQLMKDK-ELETYTGADLLHMANYFFVVGALS---------GVNSYDQMLGLSAWEA-CTMA

LKNTV--TNLWERITG---KREF-----GKLLEALEWETL---TYSRQVTQKYVGGT

>LoliumLatent_LOLAVIRUS_16/1-447

MTTLALNLAQY--NDPITKGIIAQESLRRVRPDLKAVVNV---NPYAIPTSAALVLEKLGIGTHPM--SLAV

HPHAPCKAIENQILNTVGHLLPKE-QPVTFMAMKKSKLNMLRRHP-S----------------RADTFINPI

YHPRDNVRYGLDPDPDQD-----ASITA------------------------------SFLEVKTSTAFMQD

TLHYLTPEDLLDIFETSPKLENLIASFVLPVEATRNM-KSLYPDLYSIHYTH-GGFQWAPSGHLGDAYFHEP

WQLYWL-RCGSLTRLIEEETTTTVQPPPGFEGEAYTRVRKEVRELKIYAERVTSIGAHHLFIFSRNAK----

-------------------ATPRVRSYSQNGK-WVTLPRIFRP--VSHNVQTP-LKQEVANSLMLYTYAVR-

PSLKDVAAKVRQKFDEK-DLAEHSPLEITHLINYIYYIDQRA---------YLTNDDDILSDNLLKRWIFTP

IQAAY--KKAKGFLLG---PDDF-----QKLLKALEWQPV---TFDYAVDHYKSNPW

>ShallotX_ALLEXIVIRUS_2052/1-426

MTAVQKLFDQI--SDPNTKAGYSNACFEAAQRRPKKAMAI---APFSVTTPEALTLERFGITTSPF--ATTS

HTHAADKIIENDCLTIIGHYLPKR-EAVTLIQLKRSKIHLLGRQP-S-----------------QDNFQNYC

HEPKDVLRYGITHP-------------N------------------------------SCPVVNTEYAVLAD

TLHFMSPRQLYHLFSRNPKLERLFATLVLPIEAQHRL-PSLFPDVYRLEYYK-DHFAYMPGGHGGGAYVHSY

GTLKWL-DTAQVGPVDYTKSSI---------------TNPWPITDYLSIEKIETKAAHHIMFIQRTRAQV--

-----------------DWPLPPIWVYHASEY-VKLPLI-FYP--PEANVQKT-YPHTLIKRMQLYCFSVKA

VSLRDIFAKLRQVIETQ-ELVRYSMADLIRLANYFLFITGMN---------QVSDYESPLLENLFGK-MCAS

IRMRL--RTFFQNLLG---KTSY-----AALLTVTDVIPV---HFTTQPKRREAVGE

>BotrytisX_BOTREXVIRUS_375/1-412

MSALRSLASDL--SDPTTKAAANEIAFKHFSDGIKSTKLS---FPYKVDESTAILLEKEGVLTNPF--STTL

HTHAAEKMLENRTLNEVGKKLKVHTGPFTFIQFKTGKLGHVGRGH-N----------------QGDQIINYA

REAKDLSRFETLDT-------------A------------------------------VVPPVHNRVALLHD

TLHFLSPRQLAQLFHHNPNLHLLYATLVLPVEALHNL-PSLNPHAYTLEYFSNGDFAYMPGGHAGSGYYHSA

ETLHWL-RAGQIKLGH----------------------------FSLSLNKEDSFGAHHIFTVTRQFI----

-------------------PPAPRYLYAETEL-VTLHDI-FYP--ADSNVQRP-YPATLINRMELYCRSVKA

VSLRDIFAKFRQVTETA-QLRHMRISDVIRLANYFLFSASLS---------GTNDYPSLVGHGLFKK-MRVS

FQERV--KEVLAPLVG---HTQY-----RTLMSFVQPKPF---TFSLTPVTFHAAQG

>TurnipYellow_TYMOVIRUS_21/1-419

-MAFQLALDAL--APTTHRDPSLHPILESTVDSIRSSIQT---YPWSIPKELLPLLNSYGIPTSGL--GTSH

HPHAAHKTIETFLLCTHWSFQAT--TPSSVMFMKPSKFNKLAQVN-S----------------NFRELKNYR

LHPNDSTRYPFTSP-------------D---------------------------------LPVFPTIFMHD

ALMYYHPSQIMDLFLRKPNLERLYASLVVPPEAHLSD-QSFYPKLYTYTTTR-HTLHYVPEGHEAGSYNQPS

DAHSWL-RINSIRLGN----------------------------HHLSVTILESWGPVHSLLIQRGTPPPDP

SLQAPPTLMXSDLFRSYQEPRLDVVSFRIPDA-IELPQA-TFL--QQPLRDRL-VPRAVYNALFTYTRAVRT

LRTSDPAAFVRMHSSKP-DHDWVTSNAWDNLQTFALLNVPLR---------PNVVYHVLQS--PIAS-LSLY

LRQHW--RRLTATAVP---ILSF-----LTLLQRFLPLPI---P--LAEVKS-----

>GrapevineFleck_MACULAVIRU/113-521

PAAFQPAIDFL--HNTIQKDTIASSIIAALNPSLTSSLTL---YPYALPPRWPSALNQAGIPATSY--GHQS

HPHPIHKTIETHLLHEHWANRAT--LPSTVMFMKRSKFDKLRVSNAALVK----------SASNFLHLLNPI

LTARDADRYTHLPL------------PD------------------------------T--LPSTPLYFMHH

SLMYFSPSQIAGLFLAAPFLERLYASLVLPAESTIGS-HPFFPSLYRYRTTG-EHLHYVLEGNPSSSYTQPL

TATQWL-TTSSITAGD----------------------------LHLTVTVLESWFSVHSILITRGVRPL--

------------------ELPRDIISLPSPDA-VLLPNP-SAF--DIPLRSRL-VPRDVCESLFVYVRAVRT

LRTTDPAGFIRTQSNKA-EFDWVTAEAWDHLAQFALLTAPVR---------PNTYFLPLLS--PLAV-VRHW

LFRKQ--RPIFATLTL---LSAS-----TAAAIPIAIARL---R--THSVTQ-----

>MaizeRayado_MARAFIVIRUS_1/7-405

GGHLLSGVESL--TPTTHRDTITAPIVESLATPLRRSLER---YPWSIPKEFHSFLHTCGVDISGF--GHAA

HPHPVHKTIETHLLLDVWPNYAR--GPSDVMFIKPEKFAKLQSRQ-P----------------NFAHLINYR

LVPKDTTRYPSTST-------------N---------------------------------LPDCETVFMHD

ALMYYTPGQIADLFFLCPQLQKIYASVVVPAESSFTH-LSLHPEIYRFRFQG-SDLVYEPEGNPAANYTQPR

SALDWL-QTTGFTVGH----------------------------EFFSVTLLDSFGPVHSLLIQRGRPPV--

------------------FQAEDIASFRVPDA-VALPAP-ASL--HQDLRHRL-VPRKVYDALFNYVRAVRL

-RVTDPAGFVRTQVGKP-EYSWVTSSAWDNLQHFALQTAAVR---------PNTSHPLFQS--PFAR-LSHW

LRTHT--WALWCLASP---SASV-----SAWATASALGRL---L--PLHTDR-----

>OysterMushroom_28261418_r/1-406

-MVGADLFAQI--SSSNHSDNIIGEALGRYRAHESRAVQV---ISSVLTPSEKALADRWAIPYSSS--PVGR

VPHVMLKALENHRNVNILPNYLR--GRVTVLSMKPAKLAALRARC-PG---------------VQFNLVNEI

ITPADHTRYTDPSP------------PP------------------------------D-FQGPGDMILVDD

ALQHWSRERIDAAF-RHFGGNRLIGTNIHPDEVRSGH-ASRYPDLYTIEYLPNRRYGLLSHRHKSASYEASI

DEAWML-DCGDFTVDD----------------------------RAYDVEFLLSYGPYHLVTVVPADGH---

-------------------VVRTYRFFDAPEV-VQLPDI-P----GLPNLSSPWFPALMYTQTLDHAGSLKQ

LGERDGKARIRGLRMTP-EGRRIPYATWERLLTCAKLAGL-H---------VDSEELEGITSVGLSR-LRFF

IRRKA--EEWLPDWLF---ELLY-----RDFVIARRVRRA---LTQDIVVIKVPL--

>BotrytisF_MYCOFLEXIVIRUS_/2-412

AVPLERAFASV--TQSFHKDATQGPTTNEYLVARAETQRY---APYAVRKGPAALLAQVGINTIHD--SPLS

HSHPACYALNAFHFLKVIPRYLH--GKAEVWGTKDNWFRKLQAQIDSQ--------------TTTLTHRNYA

ITARDHVRYPGT-V------------VD------------------------------HPGSCTAGTLFMHD

ALQYMTPLDVYTLFATSPEMHSLVATAVIPPESVDRL-PAFWPELYQLAYYE-DHLCYAPDGNFADAYNQPL

AAHQWM-TMKSLHGPD----------------------------FTLSVDVPASRYSHHIFVISKRPG----

-------------------LPDTHRDFMCPDL-VELPKD-FFP--GANKADKL-LPRTLVNQMVEYAASVKR

ATIKDATSRTRAYVKDQ-KYAAVTP-----VQQMYLSWFGSGLSQMQFPDRPVTPLDNFAT-ALWYR-LLPS

RLYRL--PATIRNF------Q-L-----KAFVDQLRTPQY---LLHFKLDEYHLSSN

>DonkeyOrchidDORKYVIRUS_55/1-410

-M-LSEKYSAI--KDVNQQAILQTRYRAEYDNAVNETRKT---NPYYFDDASAEFLTGLGIPALPRHEATQL

HRHGKNKAIENHLLANLKHKLS---GPCSFCFFKPAKLQLLGYNP-R----------------TAHEFLNPH

ITAKDLYRFPEDNI------------VA------------------------------DVFKFNYPTAFVQD

ALHYYSPGIILNIFEGNPGLRELYATVVIPVEIMHKH-ASFHPSLYTIEYHDDDEFSYIPESSAAGAYTQGL

ACLNWL-KYSNFSRGE----------------------------TKVSSTLLETLGAHHVLHFVRGEF----

-------------------LPQKRRLFQHPPL-VKLPPV-YCV--GRFNSHKP-FPKTLVQVLLLYAHTLKE

IRDVDIWAKLRQQIPKN-AIDDYDVGDLTLLADYVVVTAKLS---------RHPNALAISNASLLGR-LSTH

AKDAL--RRALSPFVG---PDGF-----EEHQRQLQLQPF---NYSIKTERYVSDHL

>AppleStem_CAPILLOVIRUS_96/6-424

RNPLEIAINKL--P-SKQSDQLLSLTTDEIEKTLEVTNRF---FSFSITPEDQELLTKHGLTLAPI--GFKS

HSHPISKMIENHLLYICVPSLLSSFKSVAFFSLRENKVDSFLKMHSVFSH----------GKIKSLGMYNAI

IDGKDKYRYGDVEFSSFRDRVIGLRDQC------------------------------LTRNKFPKVLFLHD

ELHFLSPFDMAFLFETIPEIDRVVATTVFPIELLFGDKVSKEPRVYTYKVHG-SSFSFYPDGVASECYEQNL

ANSKWPFTCSGIQWAN----------------------------RKIRVTKLQSLFAHHVFSFDRGRA----

--------------------CNEFNHFDKPSC-LLAEEM-RLL--TKRFDKAV-INRSTVSSLSTYMACLKT

ANAASAVAKLRQLEKR--D---LYPDELNFVYSFGEHFKNFG---------MRDDFDV--------S-VLQW

VKDKF--CQVMPHFIA---ASFF-----EPTEFHLNMRKL---LNDLATKGIEVPLS

>SclerotiniaDebilitation_S/239-648

DCRIGGSISEVVKTLGPDKEMIERDAVTEIKKEIAIVRKY---NPYHHSSKQQSALESYGIGSDPY--AVRS

HTHAAEKAIENKLLDIVGMNLRRR-SVITMLWQKRNKAHLMGRSN-C-----------------KDVYVNTI

MEAKDLVRYDQFSF-------------G-------------------------------LPSVATSTAFIGD

ALHHMTPESVFDLFERSPNLMVLHATIVIPPETLLKC-RSSNPELYSLRYYD-DKFVYIPEGHAGGSYVHEV

KNSNWL-AISHIQRGG----------------------------KFLTVKRLETLAAHHYFVIVKGKV----

-------------------ETDSIRVFQSPSQ-VELLDI-FAD--RQSNVRCS-LDHAFAIKMERYVHSLKR

LELADVTAKTRQLLSSE-ELLQYSPTDLVKIDNYFYFLAHTS---------RFNSSEELIGSGFFES-LVSP

LKQWF--SEICEKFLG---KSNF-----HKTLEALEWKVI---NYDVKTVIYDMSKP

>PotatoM_CARLAVIRUS_962609/6-439

RTPMEDIVNC---FEPATQAVIANSAATLYKNFEEQHCQY---FNYYLSPLAKRKLSMAGIYLSPY--SAVV

HSHPVCKTLENYILYSVLPSYIN--SSFYFVGIKERKLQLLKSKCKNL---------------DSVQVVNRY

VTSADRMRYTNDFV------------PYG-SYEHECLVHK-GVGLDNEALRGLV---GPLRRHKAKNLFFHD

ELHYWSSKVLIDFL-DVMRPDKLLGTVVYPPELLFKPTRSLNEWCYTYDIVG-DTLMFFPDGVQSEGYQQPL

KGGYLL-GARSLKLPD---------------------------GTVYMVDVLCSKFPHHLISITKGEA----

-------------------AAPTHRAFGPFEA-VASEAL-KATLSPDYPCAFP-VSYEVVNKIYRYLRTLKK

PDEQSAIAKLSQIIAEP-S-----GREIDFVECFARLVIHNS---------S---MCATIMPEQLKE-FMGN

WLGKM--PSVLARRFSSVRAVCV-----NKFIRGLKPYSF---TLRLNEITWWNIWE

>AppleStemPitting_FOVEAVIR/6-440

RTAAEEVIAS---FTSEEQSRISTQAVLALTNVEKDKHDL---FNYALPELAKMRLFNSGIYLSPH--SYRP

HSHPVCKTLENNILFNILPSYLD--NSFYLVSIKKNKVDFLKRRHPDL---------------QMVETINRY

ISSIDKTRYGGFFH------------VSPSKISAKFKCDRRTGFEDDASLIDLI---PGCMEGARKRFFFHD

ELHYWTKEALITFL-DHVKPEVMLASIVFPPEILAGAKESLNPWCYTFRIVG-KDLVFFPDGEQSEAYIQPV

AGSYLL-RTGKITTPS---------------------------GDIFQLDLLKSSFSHHLISITKGEA----

-------------------IGQKMRFFNGFEA-VAMKGL-NPLR-RKVESCLP-ISKNTILKIYRYLRTLKK

PDLQSAMAKLSQVCKDP-N-----GYEIKFFEEFSKLCLKCD---------T---LNTNMIPDMKRI-VQGF

FLKLF--PNPISRNFKVVQQLHL-----DNFIETLEEFNF---SINTESLSLNWKDD

>CitrusLeaf_CITRIVIRUS_208/7-445

KTAIESILGN---FEKKHVDAIYNAAAQTILSHSEFRNKH---FAYSLNSYQKKIASKVGIELYPN--GYLP

HSHPLSKIFENHLLFDVLPGVVNT-SRLVMCSIKESKVLVFKGIRDKSRRQVSDLNALNSLNNSHTSFINRL

VASKDVSRYTEEAD------------AFFQSKK---------------GSPELFSRNFIKSLENKEAVFFHD

EVHHWTKAQMFSFL-KSTKVKRFIFTVVYPPEILKKFANSQNPKVYDFKVDK-GRLFFFPDGVKTEAYEQKL

NMEWLF-SASHLRSGD----------------------------CVWTVTRHKSIYAHHLFEISIGEL----

-------------------VTDSKLFFSDYNS-IDMSKI-FLDR-FRSYEVFP-ISIEHLYKVYSYLLCLKK

PDLESGLAKLRQIIGDDVE-----IKEFLFFEQFCKRLIERQ---------T---SWGLFGHSFFEK-LTDM

ALSSL--PNSIARIFPQWKKKNT-----FEFLFSLGTLVV---DVERKVCFEHVLEE

>ApricotVeinClearing_57135/6-429

RTPQVNLLGS---FPQKHVEIIYNLQFERFKKICICRFCIFLTHSEKNQKKVASIRGWLGVPLHPT--PYLA

HSHPFSKMLENHILLNVLPGHIT--GSWVFSSIKPSKVESLATKG-KK---------------SVLKTINRL

LCAKDFGRYDVDTD------------SSVIRSIS-------------REAPDILP-EPFVRAVKGRNVMIHD

EVHHWTLDDMLGFL-DRARPNRFVFSVVYPVELLAGILESQNPKMYKFQDSKSDKIVFFPDGRASEGYEQRA

NLRWLF-CASHFRTSG----------------------------SIWTVKRIYSAYSHHLFEVVPGNY----

-------------------FTDEIRFFNDFET-IDLQCI-FKSR-FLCRDFVP-ISKDLVERVYSYLICLKK

PDMQSAMAKLKQLMGD--D------LDVRVQVFFRSLVHRIL---------NESECFSLFDVSIVNK-WKKK

FLDFA--PDWLLNGFM---TWKS-----GNFIKKLGEHEA---ARQVQEGAYPEGWM

>GrapevineA_VITIVIRUS_1-17/9-428

RVAVSNLYTNG--SEESVKAIKELKSKRLLETETR-LDGL---FDYYIPDTLREILSGYGMEFSVH--SFQG

HAHPVSKMIENHMLYRVAPNYFS--NNTLVVSCKEGKIKRLRLKN-AGN------------RNLNFTQYNRL

VHAKDHHRYENAFR------------ELDVG-------------------N-LT--NLINKESQSECIFIHD

EVQYWSLDEMQRFLGSLSKVDRIVYSIIYPAEVEAGYSQSLFPEAYTFDLKE-GRLIWYPDGKAEGAYTQPI

NPWLLR-CSKTEDSKG----------------------------RSWTITKLQTVGAHHLFSAIRGSY----

-------------------LTEESYKYDNFTI-INPNDI-LRG--RRGSKPLY-LRARMIKPTLLYLLALKK

SDSNSAVAKLRMLSSRE-EN----MDEALFVAQLAKQIRDT----------A--LYDKMGNPSLRSI-LSES

FYDVA--GNLFTRLFN---RPEYDARCLEKFIRACETTEI---HIGRRFMEGVLRGS

>PotatoT_TEPOVIRUS_1943515/6-418

RTPAELFVQS---LPKEYAEACFKSHAANFQIRSDKGVGL---FDFACSSVVKERLTKAGIPVSAF--CNQE

HSHPASKMIENHLLYNILPNYLNL-KNYTAISIKDSKVRKLLKNG-V----------------DSLETFNRL

FSCKDALRYVDPET------------CDMD--------------------K------FIARVHHSTRIFLFD

ELHYWSMNSLSDFL-DRSNVKELLATIVFPIEILLGSKRSLNPELYEFEISR-GKLHFFPDGCTSESYSQPK

-DCDIL-KVNRIVTKT---------------------------GKIFSVELIHTIGANHMVMIKEGSF----

-------------------DVDSERFFDRSSA-LTT-SL-LMP--TRAGKALR-IRRKFLLRLIIYLFSLKK

PDHHSAIAKIRQSSDDS-IF----CDEIMLADHVGKIFEKLD---------PASPFGVKGVFDLLTS-IFKD

IFLLD--GLFNWSDRR--KSEKF-----VEFMRALDYQTNKVVTCTFSGGVMRSGFL

>AppleChlorotic_TRICHOVIRU/6-419

RTPQEELLSR---LPQSQQEVISGFQYERIQKEEEKKVEN---FSFYLPEKTREWFTKSGVYLSPF--AYVN

HSHPGCKTLENHLLFNVVASYISKYSYVACLSIKSNKMSKMERLGPNS--------------VKTYDILNRL

VTAKDKARYGPLAK------------PE------------------------------RSPCPKKTNIFIHD

EIHYWSRDQLETFL-QVHRPKNLWATLVFPPEILAGYKSSVLPFLYQFEIHG-KDLVYMPDGVRSESYTQPL

ENGFLL-SSSSIIIKNRVT----------------------GVEIRYQVSLVYSLGSHHLFHIYPAEDL---

-------------------MKEEVRRFGPYDL-FDVGSL-FVK--PVRVPIQD-FPLSVFKKIFIYMSSLKK

PDVQSAVAKLRQLSDAD-I-----SIESVFMIQEFASRIEKN---------G----VESWSCSFWGC-MKDW

FFDKLPYREVLEKIGL---ADDF-----TRRLMKIKPLAF---DIHTTDRPLTVRMI

**Alphavirus supergroup (Alto+Tymo groups)**

>Sindbis_ALPHAVIRUS_251215/39-249 _R_ Sindbis_ALPHAVIRUS_251215_1-422.prf

HANA---RAFSHLASKLIE----LEV--------------------P-TT--AT--ILDIGSAPARR---M-

FSEH-------------------QYHCVCPMRSPEDPDRMMKYASKLAEK--AC-----------KITNKN-

--------------LHE------------------------------KIKDLRT-VLDT-PD----------

-AETPSLCF------------------HN-------------------------------------------

---------------------------------------------------------------------DVT

CNMRA---------E---------------------------------------------------------

------------------------------YSVMQ--DVYIN----APGTIYH---QAMKGVRTLYWIGFDT

TQFMFSAM--AG-SY-PAY-------------------------------------------------NTNW

AD-------------------------------------------------EKVLEARNIGLCSTKLSEGRT

GKLSIMRKKELKPG--------------------------------------------SR-VYFSV------

G---STLY

>HepatitisE_HEPEVIRUS_9626/65-229 _R_ HepatitisE_HEPEVIRUS_9626_1-418.prf

HP--IQ-RVIHNE----LE----LYC-R---A-R---------------S--GR--CLEIGAHPRS----I-

NDN--------P----------NVVHRCFLRPAGRDVQRWYTAPTRGPAA--NC-----------R------

-------------------------------------------RS--ALR-------GL--P----------

-AADRTYCF------------------DG-------------------------------------------

---------------------------------------------------------------------FSG

CNFPA---------E---------------------------------------------------------

------------------------------TGVALY-SLHDM----SPSDVAEAM-F-RHGMTRLYAA-LHL

PPEVLLPP--GT-YR-T-A-S-----------------------------------------------YLL-

---------------------------------------------------I--------------------

-----------HDG--------------------------------------------RR-VVVTY------

EGDTSAGY

>CuthroatTrout_FISHHEPEVIR/67-234 _R_ CuthroatTrout_FISHHEPEVIR_1-427.prf

HA--LA-KHYTTV----LE----GQL-KNAIR-----------------G--RP--YIEAGPAP-HQ---I-

-NSR-------P----------KDTHFCLVRGDARNHVRFATAPNNG-----LS-----------R------

-------------KVK-------R-----------------------IYQ-------GI--Q----------

-KEDATACL------------------NG-------------------------------------------

---------------------------------------------------------------------YQG

CHQPR---------AT--------------------------------------------------------

-------------------------------SIAIH-STYDI----PLKEFCRGM-N-NHKSDLAHVI-MHM

PSEITFPP--GV-FV-NDMTG-----------------------------------------------YRL-

---------------------------------------------------D--------------------

-----------TTG--------------------------------------------ET-AKFTY------

LEDTSNGY

>NudaureliaBeta_BETATETRAV/54-241 _R_ NudaureliaBeta_BETATETRAV_1-448.prf

HI--KLAIDLRV-----AE----KDLRRHVKNVP-----------T----------VLEIGPSVESVRYAVQ

TRD--------K----------ERVHGC-TFSDARDNLRHNKIGYEAH-Y--DR-----------KIGPDA-

--------------------------------------------A--L---------LA-AG----------

-IPTDTFCV------------------DG-------------------------------------------

---------------------------------------------------------------------FSN

CEYQS---------P---------------------------------------------------------

------------------------------LAIACH-SLYPDGESNSIMDVAKGM-A-LHGTHVIYAW-MHL

PVELLTLTD-AD-NIFEGY-S-----------------------------------------------IRFE

-ETG--------------------------------------------ALPC--------------------

-----------TKR--------------------------------------------RK-AIFSG------

YNDFGSAY

>Helicoverpa_OMEGATETRAVIR/61-284 _R_ Helicoverpa_OMEGATETRAVIR_1-467.prf

HP--IL-AGHQRV----AE----EVL-HNFAR-G---------------R--ST--VLEIGPSLHSA---L-

KLHGA-PNAP-V----------ADYHGCT-KYGTRDGSRHITALESRSVA--TG-----------R------

------------PEFK-------A-------------------DA--SLL-------AN--G----------

-IASRTFCV------------------DG-------------------------------------------

---------------------------------------------------------------------VGS

CAFKS---------R---------------------------------------------------------

------------------------------VGIANH-SLYDV----TLEELANAF-E-NHGLHMVRAF-MHM

PEELLYMD--NV-VN-AEL-G-----------------------------------------------YRF-

---------------------------------------------------H--------------------

-----------VIEEPMAVKDCAFQGGDLRLHFPELDFINESQERRIERLAARGSYSRRA-VIFSG------

DDDWGDAY

>CitrusTristeza_CLOSTEROVI/91-260 _R_ CitrusTristeza_CLOSTEROVI_1-466.prf

HP--VA-AGSRA-----LE----NHLVRKHA-GT-----------D----------YSDVGGCPLFH---L-

RAG--------H----------SGVHVCRPVYDVKDAHRRVVRHHQLSKV--SL-----------DQSDGV-

--------------------------------------------K--Q---------VG-WT----------

-VNTNSVCG------------------NI-------------------------------------------

---------------------------------------------------------------------LGE

CYHAS---------E---------------------------------------------------------

------------------------------AMV-MV-QVYDV----PLRELCRAM-I-NKKTSVCYMT-MVT

PGELLDARE-SF-FI-KDL-D-----------------------------------------------CSVE

-LD---------------------------------------------------------------------

-----------PIA--------------------------------------------DR-VVYCF------

---NNSAY

>LettuceInfections_CRINIVI/92-251 _R_ LettuceInfections_CRINIVI_1-451.prf

HG--VF-NAMRA-----CE----NFYFSRKFK-N-----------SD---------YIDAGGDVVST-----

LRS-----------------KNHNVHICSPRLDLKDAARHIQRATVIDG--LKG------------------

-------------------------------------------------------------Y----------

-GET----------------IS--FCTNK-------------------------------------------

---------------------------------------------------------------------TED

CAVN----------R---------------------------------------------------------

------------------------------DIIIAV-EVYDM----TLRDMAKAM-L-SHGSRKFEFS-CII

PPEIFTKEC-NVELY-EGR-------------------------------------------------LKVT

-R----------------------------------------------------------------------

------------IG--------------------------------------------DN-VEYY-------

YGSNGETF

>BarleyStripe_HORDEIVIRUS_/90-294 _R_ BarleyStripe_HORDEIVIRUS__1-5_2.prf

HS--MA-ACFRF-----LE----TEYLLDMVP-M---------------K-ETF--VYDIGGNWFSH----M

KFRA-------D----------REIHCCCPILSMRDSERLETRMMAMQKY--MR-----------GSKDKPL

-------------RLLSRYQNILR-------------------EQAARTTAFMAGEVNA-GV----------

-LDGDVFCE------------------NT-------------------------------------------

---------------------------------------------------------------------FQD

CVRRVPEG----FLKT--------------------------------------------------------

-------------------------------AIAVH-SIYDI----KVEEFASAL-K-RKGITQAYGC-FLF

PPAVLIGQK-EG-IL-PSV-D-----------------------------------------------GHYL

------------------------------------------------------------------------

-----------VEN--------------------------------------------GR-IKFFF------

ANDPNAGY

>IndianPeanut_PECLUVIRUS_3/94-295 _R_ IndianPeanut_PECLUVIRUS_3_1-5_7.prf

HT--MA-AVCRT-----LE----TLYIKSLLP-V---------------N-DTV--VWDVGGNWLTH----V

KYST-------N----------QDVHCCCPLLDYRDAMRKQERILSLELF--AR-----------NGKEKTE

-------------EFDACYQQIRN-------------------FENTRRTAISA---GK-NV----------

-VMNDRYCC------------------DV-------------------------------------------

---------------------------------------------------------------------FQN

CAYEPDSG----KLRV--------------------------------------------------------

-------------------------------AMGVH-SVYDM----TLQELVDGL-E-RKKIEHFIGC-FLF

SPKLLLGQE-EG-EL-PFV-N-----------------------------------------------GRFK

------------------------------------------------------------------------

-----------VKK--------------------------------------------GK-IRFFF------

LDDTTHGY

>Potato_POMOVIRUS_20376948/88-295 _R_ Potato_POMOVIRUS_2_376948_1-5_1.prf

HS--MA-AAMRK-----IE----TDVIFTSFP-K---------------N--GV--IYDIGGNWATH----A

KRDD-------G----------GFVHCCCPILDFRDAQRKMTRLIDFNRF--ID-----------DAKVVSA

-------------EKAAVAAQIKK-------------------DCDLISENAKKDAYDA-ND----------

-LNGTWFCQ------------------NK-------------------------------------------

---------------------------------------------------------------------FED

CVYDHSTLGDGKKEAY--------------------------------------------------------

-------------------------------GMAIH-SIYDI----HLVDLVSAM-E-RKKVRVLKGT-FLF

SADIIIGKK-RG-EL-PSV-N-----------------------------------------------GFYI

------------------------------------------------------------------------

-----------IDG--------------------------------------------ES-IKYSF------

YDDPNCGY

>SoilWheat_FUROVIRUS_96323/91-291 _R_ SoilWheat_FUROVIRUS_96323_1-497.prf

HS--FA-AASRK-----IE----TDLLLSRIP-K---------------R--SW--VYDIGGNWATH----V

KRND-------G----------RKVHCCCPTVDIRDSARKTVRWASIEKY--LD-----------EKEEIPP

-------------EIGERIKRLQA-------------------DEDRIYANLKSEKAQP-ED----------

-LDGKWYCG------------------NR-------------------------------------------

---------------------------------------------------------------------FED

CVFRA-------DRAY--------------------------------------------------------

-------------------------------AMAIH-SIYDI----DLSDLANAL-E-EKRIKVMSGT-FLF

SVDLLLGKK-SG-TL-PTM-D-----------------------------------------------GFFE

------------------------------------------------------------------------

-----------VED--------------------------------------------GY-VKYGF------

HNDTNPGY

>TobaccoRattle_TOBRAVIRUS_/95-289 _R_ TobaccoRattle_TOBRAVIRUS__1-498.prf

HG--FA-AAERK-----LQ----ALLLLDRVP-A---------------L--QE--VDDIGGQWSFW----V

TRG--------E----------KRIHSCCPNLDIRDDQREISRQIFLTAI--GD-----------QARSGKR

-------------QMSE--------------------------NELWMYDQFR----KN-IA----------

-APNAVRCN------------------NT-------------------------------------------

---------------------------------------------------------------------YHG

CTCRGFSDGKKKGAQY--------------------------------------------------------

-------------------------------AIALH-SLYDF----KLKDLMATM-V-EKKTKVGHAA-MLF

APESMLVD--EG-PL-PSV-D-----------------------------------------------GYYM

------------------------------------------------------------------------

-----------KKN--------------------------------------------GK-IYFGF------

EKDPSFSY

>TobaccoMosaic_Tobamovirus/81-270 _R_ TobaccoMosaic_Tobamovirus_1-5_7.prf

HS--LA-GGLRS-----LE----LEYLMMQIP-Y---------------G--SL--TYDIGGNFASH----L

FKG--------R----------AYVHCCMPNLDVRDIMRHEGQKDSIELY--LS-----------RLERGGK

-------------TVPN---------------------------------FQKEAFDRY-AE----------

-IPEDAVCH------------------NT-------------------------------------------

---------------------------------------------------------------------FQT

MRHQPMQQ---SGRVY--------------------------------------------------------

-------------------------------AIALH-SIYDI----PADEFGAAL-L-RKNVHTCYAA-FHF

SENLLLEDS-YV-NL-DEI-N-----------------------------------------------ACFS

------------------------------------------------------------------------

-----------RDG--------------------------------------------DK-LTFSF------

ASESTLNY

>TobaccoStreak_ILARVIRUS_2/97-268 _R_ TobaccoStreak_ILARVIRUS_2_1-493.prf

HS--FA-AAHRV-----CE----TLNIYNRFK---------------TKT--ER--IIDLGGNYVTH---A-

KQG--------R----------SNVHSCCPILDVRDGARHTDRYISLAAS--VE-----------NRH----

-------------------------------------------------------------R----------

-ELPVDFCC------------------HK-------------------------------------------

---------------------------------------------------------------------FEE

CDVKA---------P---------------------------------------------------------

------------------------------FAMAIH-SISDI----PISTVATHC-V-RRGVRKLIAS-VMM

DPLMMLYD--KG-HI-PLL-N-----------------------------------------------VDWE

-KEDVE------------------------------------------------------------------

------------------------------------------------------ETGKTL-IHFHF------

VDAPGLSY

>Alfalfa_ALFAMOVIRUS_96269/99-267 _R_ Alfalfa_ALFAMOVIRUS_96269_1-477.prf

HC--FA-AAHRL-----LE----TDFVYRCFG---------------NTV--DS--IIDLGGNFVSH---M-

KVK--------R----------HNVHCCCPILDARDGARLTERILSLKSY--VR-----------KHPE---

-------------------------------------------------------------I----------

-VGEADYCM------------------DT-------------------------------------------

---------------------------------------------------------------------FQK

CSRRA---------D---------------------------------------------------------

------------------------------YAFAIH-STSDL----DVGELACSL-D-QKGVMKFICT-MMV

DADMLIHN--EG-EI-PNF-N-----------------------------------------------VRWE

-IDR--------------------------------------------------------------------

--------------------------------------------------------KKDL-IHFDF------

IDEPNLGY

>BromeMosaic_BROMOVIRUS_96/80-249 _R_ BromeMosaic_BROMOVIRUS_96_1-447.prf

HS--LA-GALRV-----AE----HYDCLDSFP----------------PE--DP--VIDFGGSWWHH---F-

SRR--------D----------KRVHSCCPVLGVRDAARHEERMCRMRKI--LQ-----------E-SDDF-

------------------------------------------------------------------------

-DEVPNFCL------------------NR-------------------------------------------

---------------------------------------------------------------------AQD

CDVQA---------D---------------------------------------------------------

------------------------------WAICIH-GGYDM----GFQGLCDAM-H-SHGVRVLRGT-VMF

DGAMLFDR--EG-FL-PLL-K-----------------------------------------------CHWQ

-RDGS-------------------------------------------------------------------

-------------------------------------------------------GADEV-IKFDF------

ENESTLSY

>CucumberMosaic_CUCUMOVIRU/81-279 _R_ CucumberMosaic_CUCUMOVIRU_1-471.prf

HG--LA-GALRL-----CE----TLDCLDSFP-S-----------SGLRQ--DL--VLDFGGSWVTH---Y-

LR---------G----------HNVHCCSPCLGIRDKMRHAERLMNMRKI--IL-----------NDPQQF-

------------------------------------------------------------D-----------

-GRQPDFCT------------------QP-------------------------------------------

---------------------------------------------------------------------AAD

CKVQA---------H---------------------------------------------------------

------------------------------FAISIH-GGYDM----GFRGLCEAM-N-AHGTTILKGT-MMF

DGAMMFDD--QG-VI-PEL-N-----------------------------------------------CQWR

-KIRSAFSETEDVTPLVGKLNS-----------------------TVFSRV---------------------

------------------------------------------------------RKFKTM-VAFDF------

INESTMSY

>PelargoniumZonate_ANULAVI/80-259 _R_ PelargoniumZonate_ANULAVI_1-454.prf

HS--FA-GAMRQ-----CE----TLECLSSFP----------------ED--SL--ILDFGGSWLFH---W-

QR---------Q----------HNVHSCCPVLDARDMARHQERMISMQKC--VA-----------HRPGKF-

------------------------------------------------------------ES----------

-FESPDFCL------------------LK-------------------------------------------

---------------------------------------------------------------------AED

CEVQS---------P---------------------------------------------------------

------------------------------YAISIH-GAYDM----GFEGLCKAM-H-SHGTIMLRGT-MMF

DANMLVFN--EG-VM-EDL-N-----------------------------------------------CRWT

-KEKGDPYG---------------------------------------LRG---------------------

------------------------------------------------------APCEDM-VHFDF------

IDESTLSY

>SclerotiniaL_190694814_gb/120-295 _R_ SclerotiniaL_19_694814_gb_1-474.prf

HP--ST-QAARHA----LE----LLT-WKLVD-----------------P--AH--TTEIGPSYSSL---F-

RRAR-------P----------AFAHYDMPLLTVRDGARRNLAMLSRHKT--LD-----------E------

-------------RET-------R-----------------------ALD-------ALRTG----------

-MPSGEFCP------------------LS-------------------------------------------

---------------------------------------------------------------------GED

CSHKS---------S---------------------------------------------------------

------------------------------ALVSFF-SLHDA----SPAVLDAMM-A-RKDALVAYVA-MHL

PVALLATS--SF-D--DVATGA---------------------------------------------HCRR-

---------------------------------------------------D--------------------

-----------DDT--------------------------------------------GH-WHMSF------

PGSGAAGY

>CitrusLeprosisC_CILEVIRUS/165-355 _R_ CitrusLeprosisC_CILEVIRUS_1-577.prf

HN--NA-AALRR-----VL----RFYMRDKVGYRKDDDIPDGYHVK----------NKDVGASGMDV---I-

ADE--------L----------TDVHCCTPDLDFRDHIRLERLKKYIYSH--VC-----------PTSKDH-

-----------------------G--------------------I--ICE-------GF-RE----------

-GSTKYRCE------------------NI-------------------------------------------

---------------------------------------------------------------------GQQ

CYIEA---------P---------------------------------------------------------

------------------------------TLTFVH-SAYDI----TAAGIVDCM-I-AANAHHAIMC-LHF

PSAILSGST-SG-KD-DLL-Q-----------------------------------------------YKWD

-IV---------------------------------------------------------------------

-----------VED------------------------------------------GIKY-YKQKF------

LNDTQASY

>GremmeniellaXL1_ENDORNAVI/266-451 _R_ GremmeniellaXL1_ENDORNAVI_1-658.prf

HI--YH-HANRL-----ML----TEMLASRHP-R---------------N--AL--LYDIGGNINRH----L

DHG--------R----------LNVHCVYTTSTPADLSRHTLWVKKATQW--AH-----------ANLKNKS

-------------VHGA---------------------------------TYSSIAASM-IK----------

-EDNSMWCT------------------NG-------------------------------------------

---------------------------------------------------------------------IAN

CSHAPI------SVGF--------------------------------------------------------

-------------------------------AMSID-TLVHL----DPSDLIKFY-I-DNNVAHASHA-MTI

PDNYAYAT--KG-VL-RHN-E-----------------------------------------------GHWY

-R----------------------------------------------------------------------

-----------TNN--------------------------------------------GT-WVIEF------

VGE-SLSY

>RaspberryBushy_IDAEOVIRUS/203-433 _R_ RaspberryBushy_IDAEOVIRUS_1-655.prf

HN--MA-AAHRL-----LE----THDLLSNFP----------------AD--AP--ILDIGGNWFSH---F-

RYG--------R----------SNVHSCCPMLDLRDNERHTHRLTMTESL--MS-----------SLRHRYA

GTIDLDPDAHLSRKVS-------DSMKE-----FYKRWAVHPKDLIRLYA-------GI-RD----------

-GNSSLYCH------------------HKFGV----------------------------------------

----------------SWNDV----------------------------------LW-ESERNNCLTVPEPE

CPFKA---------K---------------------------------------------------------

------------------------------YAIMVH-SGYDL----PLKELIGGM-V-QHGVVELHGT-MIA

DPAMLVAT--SG-YI-PAL-R-----------------------------------------------CNWE

-KS---------------------------------------------------------------------

---------------------------------------------------------KGQ-IWFSF------

RDDSTMGY

>JapaneseHolly_PTERIDOVIRU/225-576 _R_ JapaneseHolly_PTERIDOVIRU_1-789.prf

HN--MA-AVIRT-----AE----TLEAVLSFP----------------KD--EP--LIDIGGDFFFH---V-

RNG--------F----------THVHCCAPILDSRDDERFSKRCMHLQKW--VI-----------DKQVSA-

-------YSSMSRRVK-------ATLKEIPNLELLCDRLREFNDACRT-P-------DD-NG----------

-MDCSMFCH------------------RTFQSSFETVEDSSSVPFEDAVVSGSFAVSPWDVETQVGRRLAGG

KFPPKKTFDDLADVAVNWVTEGVNSVVSSLKKERSGDISCDSSCVEEGAPEGVSVASASVASDES-VRRTVS

CGRRA---------R---------------------------------------------------------

------------------------------FGIMIH-SSYDM----LPVPVCQGM-V-EHDMTMVKGC-MIA

EPAMLVQT--KG-YL-PIL-E-----------------------------------------------CNWR

-LVTPLGVPLTCH---PCKLGD------------------------RFYRLS--------------------

-----------TTGDRPLS-------------------C--------------PGFDDVF-IEYHF------

QDDSTMAY

>GrapevineLeafroll3_AMPELO/487-658 _R_ GrapevineLeafroll3_AMPELO_1-854.prf

HP--FA-NAMRS-----CF----NGIFSRRCGNV-----------C----------FFDIGGSFTYH---V-

KAG--------H----------VNCHVCNPVLDVKDVKRRINEILFLSTA--GG-----------DSYVSS-

-----------------------D--------------------L--L---------TE-AA----------

-SKSVSYCS------------------RE-------------------------------------------

---------------------------------------------------------------------SQN

CDSRA---------D---------------------------------------------------------

------------------------------AGF-MV-DVYDI----SPQQVAEAM-D-KKGALVFDIA-LMF

PVELLYGNG-EV-YL-EEL-D-----------------------------------------------TLVK

------------------------------------------------------------------------

-----------REG--------------------------------------------DY-LAYNV------

-GQCGEMY

>Rubella_RUBIVIRUS_3362846/66-237 _R_ Rubella_RUBIVIRUS_3362846_1-445.prf

HP--ALHA---------ISRYTRRHWIE-WGP-K-----------EAL-----H-VLIDPSPGL--------

---------------------------------LREVARVERRWVALCLH---RTARKLATALAET------

---------------------------------------------------------AS-EAWHADYVCALR

GAPSGPFYV-----------------HP--------------------------------------------

-----------------------------------------------------------------------E

DVPHG---------G---------------------------------------------------------

------------------------------RAVADR-CLLYY----TPMQMCELM-R-TIDATLLVAV-DLW

PVALAAHVG-DD-WD-DLG-------------------------------------------------IAWH

-L----------------------------------------------------------------------

------------DH--------------------------------------------DGGC---PADCRGA

GAGPTPGY

>BeetNecrotic_BENYVIRUS_23/218-520 _R_ BeetNecrotic_BENYVIRUS_23_1-723.prf

HP--VL-AALREV----MR----QEY-SASCN-------------IL--NTKLK--TLVVGAASREV---N-

CYSS-------N----------PSVHYYFANKDSKDLVRTTLELLHSALA--TK------------------

-------------YRN------------------------------------------M-------------

-ESGERELM------------------NN-------------------------------------------

---------------------------------------------------------------------LKG

CGYIV---------KRSVENAVYEVVSDKDVAEVLRYAQTVASTKKEAKKKPNTGKRKMVMSEATRRTIELH

ELSRIVAEEKKIPNHFHFDESDFASVGNFTQLVCED-VGYNF----SVDAWLHLF-E-ATGAQTAVGY-MAL

PNELLFEH--YP-I--SDYYDYWEGVEKHGSLGGITISPLRNGQVVGMPTGVFQPVHFDKTSAGLGIPGSK-

---------------------------------------------------M--------------------

-----------GAA--------------------------------------------ER-VICHM------

SDGLGNGY

>BambooMosaic_POTEXVIRUS_9/66-213 _R_ BambooMosaic_POTEXVIRUS_9_1-411.prf

HTHA---------AAKALE----NDLYRTASY-L-----------LP-KE---RVSFLFMKPSKMQY---F-

RRRG-------D-----------VDTFINADIVAKDLARYPLETIYP-------------------------

------------------------------------------------------------------------

------------------------------------------------------------------------

----------------------------------------------------------------------RL

PEITT---------K---------------------------------------------------------

------------------------------MAFIGD-SLHYF----QPEVLEHIF-TSSPQLETLLAT-IVL

PPEATLRL--KS-LY-PEI-------------------------------------------------YTLH

------------------------------------------------------------------------

-----------YMP--------------------------------------------QK-FLYKP------

GGLSGGEY

>IndianCitrus_MANDARIVIRUS/66-214 _R_ IndianCitrus_MANDARIVIRUS_1-418.prf

HTHG---------ACKAIE----NQLLYVVGT-L-----------LP-KE---RVTMLFLKKAKLNF---M-

KRCP-------K----------FQDIFLNQHIEPRDVSRYCDFNVQS-------------------------

------------------------------------------------------------------------

------------------------------------------------------------------------

----------------------------------------------------------------------TS

TSIPT---------H---------------------------------------------------------

------------------------------TAYISD-TLHFM----DRKDLVRLF-INSPNLDTLYAT-IVL

PVEAAYRQ--PS-RY-PDL-------------------------------------------------YQIN

------------------------------------------------------------------------

-----------YDF--------------------------------------------DG-FQYIP------

GGHGGGAY

>LoliumLatent_LOLAVIRUS_16/66-222 _R_ LoliumLatent_LOLAVIRUS_16_1-448.prf

HPHA---------PCKAIE----NQILNTVGH-L-----------LP-KE--QPVTFMAMKKSKLNM---L-

RRHP-------S----------RADTFINPIYHPRDNVRYGLDPDPD-------------------------

------------------------------------------------------------------------

-----QDAS------------------IT-------------------------------------------

---------------------------------------------------------------------ASF

LEVKT---------S---------------------------------------------------------

------------------------------TAFMQD-TLHYL----TPEDLLDIF-ETSPKLENLIAS-FVL

PVEATRNM--KS-LY-PDL-------------------------------------------------YSIH

------------------------------------------------------------------------

-----------YTH--------------------------------------------GG-FQWAP------

SGHLGDAY

>BotrytisX_BOTREXVIRUS_375/66-216 _R_ BotrytisX_BOTREXVIRUS_375_1-413.prf

HTHA---------AEKMLE----NRTLNEVGK-K-----------LK-VH-TGPFTFIQFKTGKLGH---V-

GRGH-------N----------QGDQIINYAREAKDLSRFETLDTA--------------------------

------------------------------------------------------------------------

------------------------------------------------------------------------

----------------------------------------------------------------------VV

PPVHN---------R---------------------------------------------------------

------------------------------VALLHD-TLHFL----SPRQLAQLF-HHNPNLHLLYAT-LVL

PVEALHNL--PS-LN-PHA-------------------------------------------------YTLE

------------------------------------------------------------------------

-----------YFS-------------------------------------------NGD-FAYMP------

GGHAGSGY

>ShallotX_ALLEXIVIRUS_2052/66-213 _R_ ShallotX_ALLEXIVIRUS_2_52_1-427.prf

HTHA---------ADKIIE----NDCLTIIGH-Y-----------LP-KR--EAVTLIQLKRSKIHL---L-

GRQP------------------SQDNFQNYCHEPKDVLRYGITHPN--------------------------

------------------------------------------------------------------------

------------------------------------------------------------------------

----------------------------------------------------------------------SC

PVVNT---------E---------------------------------------------------------

------------------------------YAVLAD-TLHFM----SPRQLYHLF-SRNPKLERLFAT-LVL

PIEAQHRL--PS-LF-PDV-------------------------------------------------YRLE

------------------------------------------------------------------------

-----------YYK--------------------------------------------DH-FAYMP------

GGHGGGAY

>BotrytisF_MYCOFLEXIVIRUS_/67-217 _R_ BotrytisF_MYCOFLEXIVIRUS__1-411.prf

HS--HP-A-CYA-----LN----AFHFLKVIP-R-----------YLHGK--AE--VWGTKDNWFRK----L

QAQ--------I------DSQTTTLTHRNYAITARDHVRYPGTVVD--------------------------

------------------------------------------------------------------------

----------------------------H-------------------------------------------

---------------------------------------------------------------------PGS

C-----------TAGT--------------------------------------------------------

-------------------------------LFMHD-ALQYM----TPLDVYTLF-ATSPEMHSLVAT-AVI

PPESVDRL--PA-FW-PEL-------------------------------------------------YQLA

------------------------------------------------------------------------

-----------YYE--------------------------------------------DH-LCYAP------

DGNFADAY

>OysterMushroom_28261418_r/67-213 _R_ OysterMushroom_28261418_r_1-4_9.prf

HV--M----LKA-----LE----NHRNVNIL-PN-----------YL--RGRVT--VLSMKPAKLAA---L-

RARC-------P---------GVQFNLVNEIITPADHTRYTDPSPP--------------------------

------------------------------------------------------------------------

------------------------------------------------------------------------

----------------------------------------------------------------------PD

FQGPG---------D---------------------------------------------------------

------------------------------MIL-VDDALQHW----SRERIDAAF-R-HFGGNRLIGT-NIH

PDEVRSGHA-SR--Y-PDL-Y-----------------------------------------------TIEY

------------------------------------------------------------------------

-----------LPN--------------------------------------------RR-YGLLS------

HRHKSASY

>MaizeRayado_MARAFIVIRUS_1/72-216 _R_ MaizeRayado_MARAFIVIRUS_1_1-4_2.prf

HP--HP--VHKT-----IE----THLLLDVWP-N-----------YA--RGPSD--VMFIKPEKFAK---L-

QSRQ-------P----------NFAHLINYRLVPKDTTRYPSTST---------------------------

------------------------------------------------------------------------

------------------------------------------------------------------------

------------------------------------------------------------------------

NLPDC---------E---------------------------------------------------------

------------------------------TVFMHD-ALMYY----TPGQIADLFFL-CPQLQKIYAS-VVV

PAESSFTH--LS-LH-PEI-------------------------------------------------YRF-

---------------------------------------------------R--------------------

-----------FQG--------------------------------------------SD-LVYEP------

EGNPAANY

>TurnipYellow_TYMOVIRUS_21/65-209 _R_ TurnipYellow_TYMOVIRUS_21_1-469.prf

HP--HA--AHKT-----IE----TFLLCTHWS-F-----------QA--TTPSS--VMFMKPSKFNK---L-

AQVN-------S----------NFRELKNYRLHPNDSTRYPFTSP---------------------------

------------------------------------------------------------------------

------------------------------------------------------------------------

------------------------------------------------------------------------

DLPVF---------P---------------------------------------------------------

------------------------------TIFMHD-ALMYY----HPSQIMDLFLR-KPNLERLYAS-LVV

PPEAHLSD--QS-FY-PKL-------------------------------------------------YTY-

---------------------------------------------------T--------------------

-----------TTR--------------------------------------------HT-LHYVP------

EGHEAGSY

>GrapevineFleck_MACULAVIRU/178-331 _R_ GrapevineFleck_MACULAVIRU_1-541.prf

HP--HP--IHKT-----IE----THLLHEHWA-N-----------RA--TLPST--VMFMKRSKFDK---L-

RVSNAALVKSAS----------NFLHLLNPILTARDADRYTHLPL---------------------------

------------------------------------------------------------------------

------------------------------------------------------------------------

----------------------------------------------------------------------PD

TLPST---------P---------------------------------------------------------

------------------------------LYFMHH-SLMYF----SPSQIAGLFLA-APFLERLYAS-LVL

PAESTIGS--HP-FF-PSL-------------------------------------------------YRY-

---------------------------------------------------R--------------------

-----------TTG--------------------------------------------EH-LHYVL------

EGNPSSSY

>AppleStem_CAPILLOVIRUS_96/70-240 _R_ AppleStem_CAPILLOVIRUS_96_1-425.prf

HS--HP--ISKM-----IE----NHLLYICVP-S-----------LL-SS-FKSVAFFSLRENKVDS---F-

LKMHSVF----------SHGKIKSLGMYNAIIDGKDKYRYGDVEFSSFRDRVIG------------------

-------------------------------------------------------------L----------

-RD-----------------QC--LTRNK-------------------------------------------

---------------------------------------------------------------------FP-

--------------K---------------------------------------------------------

------------------------------VLFLHD-ELHFL----SPFDMAFLF-ETIPEIDRVVAT-TVF

PIELLFGDK-VS-KE-PRV-------------------------------------------------YTYK

-V----------------------------------------------------------------------

------------HG--------------------------------------------SS-FSFYP------

DGVASECY

>GrapevineA_VITIVIRUS__275/73-234 _R_ GrapevineA_VITIVIRUS__275_1-429.prf

HA--HP--VSKM-----IE----NHMLYRVAP-N-----------YF-SN---NTLVVSCKEGKIKR---L-

RLKNA-------------GNRNLNFTQYNRLVHAKDHHRYENAFRELDVGNLT-------------------

------------------------------------------------------------------------

--------------------NL--INKES-------------------------------------------

---------------------------------------------------------------------QS-

--------------E---------------------------------------------------------

------------------------------CIFIHD-EVQYW----SLDEMQRFL-GSLSKVDRIVYS-IIY

PAEVEAGYS-QS-LF-PEA-------------------------------------------------YTFD

-L----------------------------------------------------------------------

------------KE--------------------------------------------GR-LIWYP------

DGKAEGAY

>CitrusLeaf_CITRIVIRUS_208/71-252 _R_ CitrusLeaf_CITRIVIRUS_2_8_1-445.prf

HS--HP--LSKI-----FE----NHLLFDVLP-G-----------VV-NT-SR-LVMCSIKESKVLV---F-

KGIRDKSRRQVSDLNALNSLNNSHTSFINRLVASKDVSRYTEEADAFFQSKK--------------------

------------------------------------------------------------------------

-GSP-ELFSR----------NF--IKSLE-------------------------------------------

---------------------------------------------------------------------NK-

--------------E---------------------------------------------------------

------------------------------AVFFHD-EVHHW----TKAQMFSFL-K-STKVKRFIFT-VVY

PPEILKKFA-NS-QN-PKV-------------------------------------------------YDFK

-V----------------------------------------------------------------------

------------DK--------------------------------------------GR-LFFFP------

DGVKTEAY

>AppleChlorotic_TRICHOVIRU/70-223 _R_ AppleChlorotic_TRICHOVIRU_1-418.prf

HS--HP--GCKT-----LE----NHLLFNVVA-S-----------YI-SK-YSYVACLSIKSNKMSK---M-

ERLG--------------PNSVKTYDILNRLVTAKDKARYGPLAKPE-------------------------

------------------------------------------------------------------------

------------------------RSPCP-------------------------------------------

---------------------------------------------------------------------KK-

--------------T---------------------------------------------------------

------------------------------NIFIHD-EIHYW----SRDQLETFL-Q-VHRPKNLWAT-LVF

PPEILAGYK-SS-VL-PFL-------------------------------------------------YQFE

-I----------------------------------------------------------------------

------------HG--------------------------------------------KD-LVYMP------

DGVRSESY

>AppleStemPitting_FOVEAVIR/70-247 _R_ AppleStemPitting_FOVEAVIR_1-44_.prf

HS--HP--VCKT-----LE----NNILFNILP-S-----------YL-DN-S--FYLVSIKKNKVDF---L-

KRRH---------------PDLQMVETINRYISSIDKTRYGGFFHVSPSKIS--------------------

------------------------------------------------------------------------

-AKF-KCDRRTGFEDDASLIDLIPGCMEG-------------------------------------------

---------------------------------------------------------------------AR-

--------------K---------------------------------------------------------

------------------------------RFFFHD-ELHYW----TKEALITFL-D-HVKPEVMLAS-IVF

PPEILAGAK-ES-LN-PWC-------------------------------------------------YTFR

-I----------------------------------------------------------------------

------------VG--------------------------------------------KD-LVFFP------

DGEQSEAY

>PotatoM_CARLAVIRUS_962609/70-245 _R_ PotatoM_CARLAVIRUS_9626_9_1-439.prf

HS--HP--VCKT-----LE----NYILYSVLP-S-----------YI-NS-S--FYFVGIKERKLQL---L-

KSKC---------------KNLDSVQVVNRYVTSADRMRYTNDFVPYGSYEH--------------------

------------------------------------------------------------------------

-ECL-V--HKGVGLDNEALRGLVGPLRRH-------------------------------------------

---------------------------------------------------------------------KA-

--------------K---------------------------------------------------------

------------------------------NLFFHD-ELHYW----SSKVLIDFL-D-VMRPDKLLGT-VVY

PPELLFKPT-RS-LN-EWC-------------------------------------------------YTYD

-I----------------------------------------------------------------------

------------VG--------------------------------------------DT-LMFFP------

DGVQSEGY

>PotatoT_TEPOVIRUS_1943515/70-223 _R_ PotatoT_TEPOVIRUS_1943515_1-419.prf

HS--HP--ASKM-----IE----NHLLYNILP-N-----------YLNLK--NY-TAISIKDSKVRK---L-

LKNG-------V----------DSLETFNRLFSCKDALRYVDPE----------------------------

-------------------------------------------------------------------TCD--

---------------------------MDK------------------------------------------

---------------------------------------------------------------------FIA

RVHHS---------T---------------------------------------------------------

------------------------------RIFLFD-ELHYW----SMNSLSDFL-D-RSNVKELLAT-IVF

PIEILLGSK-RS-LN-PEL-------------------------------------------------YEFE

-I----------------------------------------------------------------------

------------SR--------------------------------------------GK-LHFFP------

DGCTSESY

>SclerotiniaDebilitation_S/306-452 _R_ SclerotiniaDebilitation_S_1-649.prf

HTHA---------AEKAIE----NKLLDIVGM-N-----------LR-RR--SVITMLWQKRNKAHL---M-

GRSN------------------CKDVYVNTIMEAKDLVRYDQFSF---------------------------

------------------------------------------------------------------------

------------------------------------------------------------------------

----------------------------------------------------------------------GL

PSVAT---------S---------------------------------------------------------

------------------------------TAFIGD-ALHHM----TPESVFDLF-ERSPNLMVLHAT-IVI

PPETLLKC--RS-SN-PEL-------------------------------------------------YSLR

------------------------------------------------------------------------

-----------YYD--------------------------------------------DK-FVYIP------

EGHAGGSY

**Noda group**

>Nodamura_ALPHANODAVIRUS_9/1-399 _R_ Nodamura_ALPHANODAVIRUS_9_1-414.prf _R_ Nodamura_ALPHANODAVIRUS_9_1-414.prf

MLNYET-I----INGASSALNIVSRALGY-----R--------VPLAKSLAL---------------VAGSC

VVYKIIVH------------------RRTL----VAFLVIGPYATVVQ-HRLPMALQRAI--IE-----YTR

----------------------------------------------EDR-EISLF-P---------------

----------------Q-NSIVSAEH-A-RKADNGHPISGGTRDVARETISLAIRAAGFRHYEISPARQSP-

-AEAASHQHYAAADLVRAATEDKIQDGDVVVAIDIDYYLRDIDRY-LGRGVPFMAYTFNPVEVAGRD-GDSF

FRIT-NNQVTFDVSGGGSWSHEVWDWCAFGEFIETRDA-S---WLAWFA----RAVGLT-------------

-----KSQIHKVHYCRPWPQSPHRALVWCLPVASYWRFTFI--PTDLHTRTLRRVRYQD-TSR---PGWNSI

VSTG-SEGLNISLGREG--ADHCVTIPKVHYDMLMGLSS---------------AQSLSSRMIGL--KY---

T--DPSVLATVAQY-YQGKNVEVADADR-IG--R

>MacrobrachiumRosenbergii_/1-404 _R_ MacrobrachiumRosenbergii__1-419.prf _R_ MacrobrachiumRosenbergii__1-419.prf

MKCNN--L----LYGVE---------LPV-----C--------LQSSMGMVV---------------VTGLA

-IVYLISY---ILIYSFT---HGPRLSRRL----ASYVVIGPYEAIAK-SRPAMALQRAM--VD-----LTR

----------------------------------------------RDV-RTDWY-P---------------

----------------L-NNLLMNKP-H-RLSENGHKTSGAVRDAARNLITSAITSLGMDKYEISPGGHTV-

DEQLASHRHYAVNDLHRASADDAVKENAVIVAIDTDYYLRDPSIY-FSNNNPFILHTFQPITVAGKD-GDVR

FTIS-DNQVDYRVDGGGRWQHKVWNWCDYGEFLIFKEHPRI-LSINWWL----SFLGIR-------------

-----KVIYQKIQHARPWVDCPNRALVWGLPQFTSYMITWL--PIEMNARELGRVNYQC-ASR---PGWNCL

VDHT-STKLVASIGREG--NDCHVELAKEDLDVVLGLST---------------MQSVTSRLLQM--GY---

K--QPQTLATVCQF-YNSAAYDILSCSI-VA--R

>DrosophilaNodaSW2009_2622/1-391 _R_ DrosophilaNodaSW2__9_2622_1-4_6.prf _R_ DrosophilaNodaSW2__9_2622_1-4_6.prf

MTLKV--I----LGEHQ----------IT-----R--------TELLVGLAT---------------VSGCG

AVAYCI---------------------SKF----WGYGAIAPYPQSGG-NRVTRALQRAV--ID-----KTK

----------------------------------------------TPI-ETRFY-P---------------

----------------L-DSLRTVTP-K-RAFNNGHAVSGAVRDAARRLIDEAITSVGGSKFEVNPNPNSS-

-TGLRNHFHFAVGDLAQDFRNDTPAEDAFIVGVDVDYYVTEPDVL-LEHMRPVVLHTFNPKKVSGFD-ADSP

FTIK-NNLVEYKVSGGAAWVHPVWDWCEAGEFIASRVRTS---WKEWFLQLPLRIVGLE-------------

-----KVGYHKIHHCRPWTDCPDRTLVYTIPQYTVWRFNWI--DTELHVRKLKRIEYQD-ETK---PGWNRL

EYVTDKNELMVSIGREG--EHAQITIEKEKLDMLSGLSA---------------TQSVNARLIGM--GH---

K--DPKYTSMIVQY-YTGKKVVSPISPT-VY--K

>BlackBeetle_51014189_ref_/1-391 _R_ BlackBeetle_51_14189_ref__1-4_6.prf _R_ BlackBeetle_51_14189_ref__1-4_6.prf

MTLKV--I----LGEHQ----------IT-----R--------TELPVGIAT---------------VSGCG

AVVYCI---------------------SKF----WGYGAIAPYPQSGG-NRVTRALQRAV--ID-----KTK

----------------------------------------------TPI-ETRFY-P---------------

----------------L-DSLRTVTP-K-RAVDNGHAVSGAVRDAARRLIDESITAVGGSKFEVNPNPNSS-

-TGLRNHFHFAVGDLAQDFRNDTPADDAFIVGVDVDYYVTEPDVL-LEHMRPVVLHTFNPKKVSGFD-ADSP

FTIK-NNLVEYKVSGGAAWVHPVWDWCEAGEFIASRVRTS---WKEWFLQLPLRMIGLE-------------

-----KVGYHKIHHCRPWTDCPDRALVYTIPQYVIWRFNWI--DTELHVRKLKRIEYQD-ETK---PGWNRL

EYVTDRNELLVSIGREG--EHAQITIEKEKLDMLSGLSA---------------TQSVNVRLIGM--GH---

K--DPQYTSMIVQY-YTGKKVVSPISPT-VY--K

>FlockHouse_351066202_gb_A/1-391 _R_ FlockHouse_351_662_2_gb_A_1-4_6.prf _R_ FlockHouse_351_662_2_gb_A_1-4_6.prf

MTLKV--I----LGDHQ----------IT-----R--------TELLVGLAT---------------VSGCG

AIAYCI---------------------SKF----WGYGAIAPYPQSGG-NRVTRALQRAV--ID-----KTK

----------------------------------------------TPI-GTRFY-P---------------

----------------L-DSLRTVTP-K-RAINNGHAVSGAVRDAARRLIDEAITSVGGSKFEVNPNPNSS-

-TGLRKHFHFAVGDLAQDFRNDTPAEDAFIVGVDVDYYVTEPDVL-LEHMRPVVLHTFNPKKVSGFD-ADSP

FTIK-NNLVEYKVSGGAAWVHPVWDWCEAGEFIASRVRTS---WKEWVLQLPLRIVGLE-------------

-----KVGYHKIHHCRPWTDCPDRSLVYTIPQYTVWRFNWI--DTELHVRKLKRIEYQD-ETK---PGWNRL

EYVTDKNQLMVSIGREG--EHAQITIEKEKLDMLSGLSA---------------TQSVNARLIGM--GH---

K--DPQYTSMIVQY-YTGKRVVSPISPT-VY--K

>StripedJack_BETANODAVIRUS/1-385 _R_ StripedJack_BETANODAVIRUS_1-4_2.prf _R_ StripedJack_BETANODAVIRUS_1-4_2.prf

MRRFE--FE---LAR----------------------------------------------------MSGAA

FCVVTGY---------------------RL----LTSKWLADRVEDY-RQR-------VI--AD-----RKQ

-------------------------------------I----LRDAAVI-RTSI------------------

----------------Q-KQMELVRI--SVRKGHSHQEAATERNSATDTMIGVVEKCGYEPYIISPSPREK-

-EYHGSRQFYSLADFRQDYRRDEITDRHVIVMTDVDYYVD-MHEL-VGLGVPILLYTFQPSTVSGEV-KDGY

FTIT-DDHVHYRVAGGKDVRHRIWNYNQD--TMFVRSKPRG-FWASL-KQILRDITGITALCGYLYLKLGIA

PFGDQVTLF-TVDQ---FKMGEHRNIVSIVPFATCRSNL-L--KISEYGAELDYMRYQQRNNN---ANFNAV

TYIS-QEGPLISLGLEG--NFASVQLPLQDFENIRTAYE----------L-SKNN-NLSDTVRRS--A----

---RSCKEAAIIHK-CLQA-GCDLASEV-VH--K

>BatGuanoGF4n_297598937_gb/1-373 _R_ BatGuanoGF4n_297598937_gb_1-389.prf _R_ BatGuanoGF4n_297598937_gb_1-389.prf

MGRDT--LSLL-LRGTA----------LFPACCIR--------MRRGTKMWI---------------VAG--

-----------------------------------GIAVFGAYYVYEQ-RRGLMNWLKWV--GDCVVSHFSM

RL-HHTRRH------------------------LF-------------------------------------

------------------NTRFYYHP-AREIPGHSHPSAASIRNGAVFSMDNYASITGATFYTVSYSDRD--

-RSRGQRLYYQPRDYCLEARNCDMRPTDIIRMVDVDYYVD-MHYW-LGLGHPVLLYTFVPTRAGGKT-FDGS

YSFS-GNRVKYRVNGGATYEHQLWDYNHD--FITVDKWT----G-VWV------------------------

--------Y-SVDQK--ITTDPERRVVQLTPRCFVPKPLSW----FVPRNPLRRLNVLH---E---SDWSVL

TSQDSSGTQYISIAKDA--STYAVTMPRELYEAIKIRHDTSGN--------ARNISDIERMLLKH--GF---

E--DPHIKAAILYD-CISQVRIPTTAPI-VT--A

>Alphanoda_HB2007_29531469/1-358 _R_ Alphanoda_HB2__7_29531469_1-376.prf _R_ Alphanoda_HB2__7_29531469_1-376.prf

MFVVK--RE-P-CPRRK----------------------------M-----------------------G--

-----------------------------------GDTEYRP--------RA-SAPIRRC--V-----DFVR

KVLCRQDIV------------------------KMSKLM---HRRKTVV-PTDFRKK---------------

----------------L-HHAMRNIPLLSAPADHTHPDAARYRTSVACRIEEVILNAGFRPYSVSMSKRD--

-KYDGCRYYFMQRDYDKEFRDDPITSGNVIMMIDVDYYVD-FNRY-LQFGNPVMIYTFVPTSAGGPT-TDGT

FSIE-NDHIVYRVSGGATYRHEVWDHNHD--FVSVKDKHG---N-LLT------------------------

--------Y-VVEQHT-CEFDPQRRIIGYYPVTSVPAHCFV----KPDTLGVKRLKFTS-------DNMNSV

RDVV---SNTISVACHG--SPHSVTIPINLFDAIKIRHDRAT---------KPVIADVERLLQAA--EP---

KIPQASTKAPILFG-LL-N-A-KPSANI-GA--T

>PierisRapae_63029695_gb_A/1-424 _R_ PierisRapae_63_29695_gb_A_1-442.prf _R_ PierisRapae_63_29695_gb_A_1-442.prf

MSSVIKTIV---VKTLQCTV----KCVC-VGVRQQYGLLRR--HPKTIACAL---------------VGGI-

-IAHDVLH-L------------------YA----AVYRVENPWEFVP-TEGPR-----GVIVLR-----VYN

--------ALRFTAAKWRWNCWTIFGKFPFNSEQFSRIMETSTERKPDL-RPLLV-P---------------

----------------AHETIEVY----PCNNIHSHPRSAEFRSSANEYLVRSVRRAGYPPYVVSSSKRDF-

--CDGNRFFYCPKDFGMQFRNDPISNNHALVFTDVDYYAN-MNRW-LQLFLPTCLYTLVPTRLNYSN-DEFA

YRFE-NNEIVYNVTGGGEYRHSLWDYKGD--TVTVVDEYGN-L-----------------------------

------LIF-DIEQRR-VQGDEQHRLIWLLPKAKITDPLWIVGPTEWYDKLLVRKTVQ----Q---GRLKIL

WEPI---ADDLSIGLHG--SNYSVSLKGELFEAIRTRISCKDS--------TPYVSDVERMLREA--KH---

K--NFLTDAPILYH-CFQD-DVIIRPNV-VK---

>Pariacoto_20260794_ref_NP/1-372 _R_ Pariacoto_2_26_794_ref_NP_1-389.prf _R_ Pariacoto_2_26_794_ref_NP_1-389.prf

MEEHI--PL---PSQYE-SP----KALP-PRVPS-----SR----WLRSLRPRL-------------ANSC-

-LALKIRAHE------------------SL----VKIRLCKPYDAQ--------------------------

---------------------------------SRSKIIEKVIERRETR-KTLAH-Q---------------

----------------LK-DLKLV----PVARDHTHGRAAKFRTSANIWMNEAMRAAGYEPYNVSMSNHDI-

--ERGNRYFYFAKDLTIPYRNDPVSDNTGFVFCDVDYYAD-MEKW-MQHFKPMLLYTLVPESLSYHC-DDHS

FHVN-DDRVFFDVRGGASYSHQLWDYTGD--TICVRGKNKE-L-----------------------------

------LVF-TIEQKC-IQGDPHRRIIFLEPAARVAWPFYK--PMKVE-VGLKRKCMT----A---GQVNVL

YEPI---DDKISLSASG--SRHTVETTGRTLAAITARMKNKTS--------PPMVADVERILRDA--GD---

K--EACVNAPILFE-LIPE-AKF-RVNV-VK---

>Tetnovirus1_TETNOVIRUS_30/1-460 _R_ Tetnovirus1_TETNOVIRUS_3__1-474.prf _R_ Tetnovirus1_TETNOVIRUS_3__1-474.prf

MLYNI--AHDLTILGYDISE----RSLSHPRIIV-----RPFLTELVSVLRDVAKEALITPVIVPTTLTGSL

-MAYVYYQVANTYDQHDNPLIRIPM--NCI----YTLSICFPYRTNISLNH-------KL--SD-----DLP

-----------------------L------------IR----DYYGMPI-TQKDW-PTSNVGWYRRYFGTLR

PNDQRLIYRYPRCPRSIPSLVRDYDW--KLESVSPHPEEAAQRNAARFNLPSMALSMGYTPFTISGRPSEA-

--TLGFNLVYNYRDTERLLRGDVLTDRNAIILVDTDYYID-MPKL-LTTGLPILMYTFVPQRLAGST-QGTN

YYIDKHSQVHFRAAGGTTWNHYLWDYNHD--YLTSPSVNNK-I-----------------------------

------VSY-QVYQ---TQLSENRRLIILFPKTVAPYLR-R-----VEATPLVRARFN----Q---GEYCLM

KTKT-E-QQEISISM-G--DEHVLTLPLDVFFELRGRFRNWKQADILYNV-ACLEQNAKVLLQNK--GY---

K--TEDVNDVVARISVLHA-YLRDHANQELYTLE

>SclerophthoraA_45476494_r/1-358 _R_ SclerophthoraA_45476494_r_1-375.prf _R_ SclerophthoraA_45476494_r_1-375.prf

MPWKP--GK------CHLLA----AG-----------------------LAG---------------VCCI-

--AYTKRG---SLRDAA----------SAL----CEWLADEPAI----------------------------

------------------------------------------VS---DLSRDA-------------------

----------------F-TTTCVDPV--VAISGHTHASAASLRTAATRYAQNVAQYCGTEVYVVGMSRSDQR

KDMKGSRQWYWTKDVNAANRNDRPGHRDIRFLCDVDYYVD-MPAMLVAEAKPVLLYTVVPEEATSSANDESA

FYFEADGSLRTMVAGGGGYSHCIWDYAAD--SFLVVKKICGV---------PIRA-----------------

------VAY-AVER---KQVGKHRQMVLLAPVRAFSGLAAILAYLLLETKELRRFNPIV---EAGGEKFVRF

NVMSSDGALMVTTARPG--SSLCATVSCADDDAIATVAR--------------LG-STNLMLPTTASWV---

K--DRP-AAAVLTD-YHRK-CGK-KAKF-VV--Y

>PlasmoparaA_301070430_gb_/1-358 _R_ PlasmoparaA_3_1_7_43__gb__1-378.prf _R_ PlasmoparaA_3_1_7_43__gb__1-378.prf

MSFGT--LF----SRYTLMV----SS-----------------------V-S---------------VVGL-

--A----T---PWRYVM----------SKTLNEFGEEWSIEPA-----------------------------

-----------------------------------------------NL-RSL-------------------

----------------F-GDIQVVEF--KRQPGHPHGAAAADRSAGSYFIERFARDVGREVVYYQCSNSDLR

NGRSGAREWYWMKDAYVQPKLFSLSSSSMVSIIDVDQYVD-MNWFLTEHFCPVLLYTFQPSVCAADR-GEYS

YTFNSNNEVIYRVAGGAEYVHKVWNYDTD--VVTTTRLFAGKSWTKWRNWIPFVT-----------------

------CAY-LVDK---RQMDADHQIIGLFPVKRWFGPLAL-LARCLKGRPLSRFKIV----R---GDFLRL

LNHG-HNGMTISTGKVE--SPLCGTITAREDAALASLAR--------------TT-KSGLTLMHVKKLM---

P--DDTLGATYVYE-YHSQ-KLP-EATV-VS--Y

>Tetnovirus2_302171585_gb_/1-354 _R_ Tetnovirus2_3_2171585_gb__1-368.prf _R_ Tetnovirus2_3_2171585_gb__1-368.prf

MQLQN--YH---LKR----------------------------------------------------VYAPN

PLQVVVH---------------------KL----LPRSAIRPE-----------------------------

---------------------------------------------KVIV-RYKLS-KMDY------------

----------------L-RHIRLAPPTQEANKLHPHPMSAELRRRALQAVRYMCKQLNKQMHVISPGPNESL

LADASTRYHYWARDLMVAESNHPLTHDSLLYLQDVDYYTD-MNYI-LGAGLPVVLYTIIPRKAASRK-QEYA

YRFE-NGQFLFTSNGGTAFSHLLWDYDLD--TCAIERSPQ--TWLSWFLGPRERM-----------------

-----VTFF-EKTE---VYLGMDRYLVFFIPRYRVSGWTYD--KMNLGISRLVRRSVT----S---NGVTIW

PDRA-T-PGLLSLALQGEFNYQTISIKREMLETVRLKMNLIGDT-----FKSSTHAEISRYLVNN--KVVPV

E--ESALAATILCR-LFGK-I-------------

**Chropara group**

>ChronicBee|188572672|ref|YP_001911136.1|/1-534 hypothetical protein CBPV_s1gp1 [Chronic bee paralysis virus]

M-NLSNVNDV--------------LSSVSSTLRPGEGGMRQIEYIFD---------PPSLPTRIYNSVHDWF

------YPPLHP---IAAAIK-AAYTNFGVQTKAAATAVVNATVPKTVISGLKICASSIGVT-----IVICS

AG--------YWFYRTRHLKIVQVTNNGTMCQINELREYFETLCGPAGAHMDLPDSIPGHHLRIAAERRFLE

RACCEVFQQIGVRFRDVGGSRVRGEDYAHLKHLCNPCIDSSDILRDAKNPEPPFSSCGGRGEVCPSRHDFPG

ALLCHSDYYL-YDELSYVIRGHTFIINHSFLEENKHFH-NEAFITKKGDGVIMSTPDGTEY-QHPYNVWKAE

GCIIGSGGAAVYARVYRGPSMDVYYAYPASGTYHSDDPHRLKRSDQFENLRLSSGNIARLSFKSVKIQTTSG

DII------LDCPRDPFERAVAKCGSGVRDLKFLPTLSSYIISKLTMHDLEINNAQD--ILELANIMCERFA

FDFYSHSHLEYDPTSINIFTRLRRHAMLFARAIRHTLARNLTCSIINRPILRQFTPWAFKSITMPAYIKHRR

ARHIDLRLASDKPKPF

>AnophelineC|545716005|gb|AGW51752.1|/1-536 RNA-dependent RNA polymerase-like protein [Anopheline-associated C virus]

M-D---------------------IKKIIEHLDPKNETFYSIEQAFD---------PSPIWVRATRWFRVRF

------SPEPNL---LVRGIKTAANSLVGGIPRVRNGAVNGAGHAKTVARAAWDTAKYHSTV-----LVISA

VGTCAAIYAATWIWRSRRVLFVQVINDGTMLPIPTLKDHFATRL--SKRPEDYGPTLPGLHLRIAAERRRAE

QAVFEVFDDIKLRFRDVGGSLSRGKDYVQLKHVCNPVLDSADILRRAKADECPFDTCKGVGQKCPQRAAFPG

ALLCHSDYYMDQEDLVACVRSHTFVINHRYTADSKVNHLGEMEVAVEGPYVKGGTSDGTRY-SHAYNLWQAE

GSVVGKEGAFVYTRVWTGPSMDIYYAYPAPGTYRKDDPQALKRSTDINMVKLTTGHFAHITDHQVSVRTTDG

TLV------MAFPRTELEKTVLKCGGAIRDSKYISTVTSYVTAKLTAGDHNIASAAD--LTEIALAACETYA

LDIYSRHTRNYDPADLGTFKRLWLGFQDSTHRGRLWVTSAYTHDYVFRHPLSALTPWLFRRVEMPGYVRHFT

NTRVNLKSLNPDPRPF

>LakeSinai1|335057597|gb|AEH26192.1|/1-562 Orf1 [Lake Sinai virus 1]

MVKLAFAAFVSLYIICQVPPISYWIEDLIDDFDTMCSHEYAAADAYNNFVHRHRVAAYAAGVRIHRYRTPWY

CAASRSVPVVHPLNWLMTQYDLTTGHVRELLDRLEVVNVTLRDGLRTVADTAFTAYMYYQIMWCQLALIVLL

VGLFSAIIC------SSRVRVVRVRNRVRGYSITDLRNEFDEST------SDITLPLSRGHSCLNFQRRVAE

SWCLDQLLRYFHGVRFISASTGRWAELGHRIHRCSPVVMDGSFVPEATHH---GVLCRRQPSACRDRFEIPA

CVISHADYYMSPDQLASTVTGPTFIINHDYLAPDSLSV-AEATTRVANGHVTSSVREGPIYGPHPYYLWNNE

GVVVSSAGAFRYYRVGRLFDTSVYYAFPAAGTYSCDDSTNLRRS--------TVGDLHYYSPHEKKFVSYSA

DDTNYHVFGTSVPRSLADYCAATFCRSARDDKFYDGLRSYYQNRCRA--IGFTDARDTLILDFIIHLCDEAS

LKTFGFSRLSVAPSSWTAYCLSWL-LVKVNHMMPLALTS-FVVSALHRFFGAKSAPWNWASIHLPTY--DMV

TSPFRLRMFGRNPTTF

>LakeSinai2|335057590|gb|AEH26187.1|/1-561 Orf1 [Lake Sinai virus 2]

M-KVVFLVSFAVFLICQVPPVSYWLEDALDEYNTMCSYEYAAAEAYNNFIHKHRVAAYAAGVRILRYRTPWY

CFASRSVPVVHPFSWLMGQYDATIDRVGTLMQRLEIANVSISDGLRTVADTALKAYFYYQIIWFQLFSIIML

VGFFSAVIC------SSRVRVVRIRNRVRGYSVAELRSDFEESM------SAILAPLSRGHSCLNFQRRVVE

SWAIDQLLRYFRCFRSVASSQGRWAEVGHRLHRCSPVVMDGAFVPEYDQR---FSACRRHPSVCPDRFDTPA

AILSHVDYYMTPDQLAAAVTGPTFIVNHDYSSIDTLSV-AEVSLRSAGGLVTASVRDGPTFGPHPYYRWSDE

GVVVASSGAFQYFRIGRLFDTTLYYAFPTSGTYSQDDPSALRRS--------TSGDLHYYSPHEKRFVSYTA

DDTHYHVFGVSVPRSLADYCAATFCRAVRDDKFYDSLRSYYQNRCRA--IGFADARDTLMLDFIIHLCDDAS

LRTFGFSRLSSAPSSWSAYCLSWI-LVKVNHMMPLALTS-YVLNVLHRFFGAKAAPWNWATIHLPTY--DMV

TSPFRLRLFGRNPTVF

**Nodavirus supergroup (Noda+Chropara groups)**

>Nodamura_ALPHANODAVIRUS_9/1-243 _R_ Nodamura_ALPHANODAVIRUS_9_1-414.prf _R_ Nodamura_ALPHANODAVIRUS_9_1-414.prf _R_ Nodamura_ALPHANODAVIRUS_9_1-414.prf _R_ Nodamura_ALPHANODAVIRUS_9_1-414.prf

MLNYETI--IN-----G------------ASSALN----------IVSR-ALG----------YR-------

-------------VP---------------------------------------------------------

-------------------LA-K-------SLALVA------------GS-CV-VYKIIVH-----------

RR-----TLVAFLVIG--------PYATVVQHR--LPMALQRAII-E-------YTREDREI----------

------------------------------------------SLFPQ-NSIVSAEH--ARKADNGHPISGGT

RDVARETISLAIRAAGFRHYEISPARQSP--AEAASHQ-----HYAAADLVRAATEDKI-------------

--QDGDVVVAIDIDYYLRDI-DRY--LGRGVPF--MAYTFNPVEVAGRDG-DSFFRIT-NNQVTFDVSGGGS

W-SHEVWDWCAFGEFI

>MacrobrachiumRosenbergii_/1-245 _R_ MacrobrachiumRosenbergii__1-419.prf _R_ MacrobrachiumRosenbergii__1-419.prf _R_ MacrobrachiumRosenbergii__1-419.prf _R_ MacrobrachiumRosenbergii__1-419.prf

MKCNN-L--LY-----G------------VEL--------------------P----------VC-------

-------------LQ---------------------------------------------------------

-------------------SS-M-------GMVVVT------------GL-AI-VYLISYILIYSFTHGPRL

SR-----RLASYVVIG--------PYEAIAKSR--PAMALQRAMV-D-------LTRRDVRT----------

------------------------------------------DWYPL-NNLLMNKP--HRLSENGHKTSGAV

RDAARNLITSAITSLGMDKYEISPGGHTVD-EQLASHR-----HYAVNDLHRASADDAV-------------

--KENAVIVAIDTDYYLRDP-SIY--FSNNNPF--ILHTFQPITVAGKDG-DVRFTIS-DNQVDYRVDGGGR

W-QHKVWNWCDYGEFL

>DrosophilaNodaSW2009_strain_of_FlockHouse/1-229 _R_ DrosophilaNodaSW2__9_2622_1-4_6.prf _R_ DrosophilaNodaSW2__9_2622_1-4_6.prf _R_ DrosophilaNodaSW2__9_2622_1-4_6.prf _R_ DrosophilaNodaSW2__9_2622_1-4_6.prf

MTLKV-I--LG-----E------------HQI-------------------------------TR-------

-------------TE---------------------------------------------------------

-------------------LL-V-------GLATVS------------GC-GAVAYCIS-------------

-------KFWGYGAIA--------PYPQSGGNR--VTRALQRAVI-D-------KTKTPIET----------

------------------------------------------RFYPL-DSLRTVTP--KRAFNNGHAVSGAV

RDAARRLIDEAITSVGGSKFEVNPNPNSS--TGLRNHF-----HFAVGDLAQDFRNDTP-------------

--AEDAFIVGVDVDYYVTEP-DVL--LEHMRPV--VLHTFNPKKVSGFDA-DSPFTIK-NNLVEYKVSGGAA

W-VHPVWDWCEAGEFI

>StripedJack_BETANODAVIRUS/1-211 _R_ StripedJack_BETANODAVIRUS_1-4_2.prf _R_ StripedJack_BETANODAVIRUS_1-4_2.prf _R_ StripedJack_BETANODAVIRUS_1-4_2.prf _R_ StripedJack_BETANODAVIRUS_1-4_2.prf

MRRFE-F--EL---A-RM------------------------------------------------------

------------------------------------------------------------------------

-----------------------------------S------------GA-AFCVVTGYRL-----------

----------------------------------------------------------------L--TSKWL

ADRVE---------DYRQR-VIADRK----QILRD-AAVIRT---SIQKQMELVRI--SVRKGHSHQEAATE

RNSATDTMIGVVEKCGYEPYIISPSPREK--EYHGSRQ-----FYSLADFRQDYRRDEI-------------

--TDRHVIVMTDVDYYVDMH-ELV---GLGVPI--LLYTFQPSTVSGEVK-DGYFTIT-DDHVHYRVAGGKD

V-RHRIWNYNQDTMFV

>BatGuanoGF4n_297598937_gb/1-229 _R_ BatGuanoGF4n_297598937_gb_1-389.prf _R_ BatGuanoGF4n_297598937_gb_1-389.prf _R_ BatGuanoGF4n_297598937_gb_1-389.prf _R_ BatGuanoGF4n_297598937_gb_1-389.prf

MGRDT-L--SL-L----LR---------GTA----------------LF--P-----------AC-CIR---

--M-----------R---------------------------------------------------------

-------------------RG-T-------KMWIVA------------GG-IA-VFGAYYV-----------

----------------------YEQRRG------------------------------------LMNWLKWV

GDCVV--------S-HFSMRLHHT---------------RRHLFNTRFYYH-PA----REIPGHSHPSAASI

RNGAVFSMDNYASITGATFYTVSYSDRDR---SRGQRL-----YYQPRDYCLEARNCDM-------------

--RPTDIIRMVDVDYYVDMH-YWL---GLGHPV--LLYTFVPTRAGGKTF-DGSYSFS-GNRVKYRVNGGAT

Y-EHQLWDYNHDFITV

>Alphanoda_HB2007_29531469/1-218 _R_ Alphanoda_HB2__7_29531469_1-376.prf _R_ Alphanoda_HB2__7_29531469_1-376.prf _R_ Alphanoda_HB2__7_29531469_1-376.prf _R_ Alphanoda_HB2__7_29531469_1-376.prf

MFVVK-R--EPCP-R-RKMGGDTEYRPRASA----------------PI--R-----------RC--VDF--

--V-----------R---------------------------------------------------------

-------------------KV---------------------------------------L-----------

----------------------CRQDI---------------------------------------------

-------------V-KMSKLMHRR------KTVV--PTDFRKKLHHAMRNIPLL----SAPADHTHPDAARY

RTSVACRIEEVILNAGFRPYSVSMSKRDK---YDGCRY-----YFMQRDYDKEFRDDPI-------------

--TSGNVIMMIDVDYYVDFN-RYL---QFGNPV--MIYTFVPTSAGGPTT-DGTFSIE-NDHIVYRVSGGAT

Y-RHEVWDHNHDFVSV

>Pariacoto_20260794_ref_NP/1-232 _R_ Pariacoto_2_26_794_ref_NP_1-389.prf _R_ Pariacoto_2_26_794_ref_NP_1-389.prf _R_ Pariacoto_2_26_794_ref_NP_1-389.prf _R_ Pariacoto_2_26_794_ref_NP_1-389.prf

MEEHI-P--LP---S-Q------------YE----------------SPKAL-PPR-------VPSSRW---

LRS---------LRP---------------------------------------------------------

-------------------RL-A-------NS-C------------------L-ALKIRAHE-S--------

----------------LVKIRLCKPYD---------------------------------------------

-------------AQSRSK-II-E------KVIE--RRETRKTLAHQLKDLKLV----PVARDHTHGRAAKF

RTSANIWMNEAMRAAGYEPYNVSMSNHDI---ERGNRY-----FYFAKDLTIPYRNDPV-------------

--SDNTGFVFCDVDYYADME-KWM---QHFKPM--LLYTLVPESLSYHCD-DHSFHVN-DDRVFFDVRGGAS

Y-SHQLWDYTGDTICV

>PierisRapae_63029695_gb_A/1-280 _R_ PierisRapae_63_29695_gb_A_1-442.prf _R_ PierisRapae_63_29695_gb_A_1-442.prf _R_ PierisRapae_63_29695_gb_A_1-442.prf _R_ PierisRapae_63_29695_gb_A_1-442.prf

MSSVI-KTIVV---K-T------------LQ----------------CT--V-----------KCVCVG---

VRQ---QYGLLRRHP---------------------------------------------------------

-------------------KT-I-------AC-ALV------------GG-II-AHDVLHLY-A--------

----------------A-VYRVENPWEFVPTEGPRGVI-VL---------------RVYNALRFT--AAKWR

WNCWTIFGKFPFNSEQFSR-IMET------STER--KPDLRPLLVPAHETIEVY----PCNNIHSHPRSAEF

RSSANEYLVRSVRRAGYPPYVVSSSKRDF---CDGNRF-----FYCPKDFGMQFRNDPI-------------

--SNNHALVFTDVDYYANMN-RWL---QLFLPT--CLYTLVPTRLNYSND-EFAYRFE-NNEIVYNVTGGGE

Y-RHSLWDYKGDTVTV

>SclerophthoraA_45476494_r/1-211 _R_ SclerophthoraA_45476494_r_1-375.prf _R_ SclerophthoraA_45476494_r_1-375.prf _R_ SclerophthoraA_45476494_r_1-375.prf _R_ SclerophthoraA_45476494_r_1-375.prf

MPWKP-G--KC------------------HLL---------------AA-----------------------

------------------------------------------------------------------------

---------------------------------GLA------------GV-CCI---AYTKR----------

----------GSLRDA--------AS----ALC--E-WL------ADEPAIVSDLSRDAF------------

------------------------------------------------TTTCVDPV--VAISGHTHASAASL

RTAATRYAQNVAQYCGTEVYVVGMSRSDQRKDMKGSRQ-----WYWTKDVNAANRNDRP-------------

--GHRDIRFLCDVDYYVDMP-AML--VAEAKPV--LLYTVVPEEATSSANDESAFYFEADGSLRTMVAGGGG

Y-SHCIWDYAADSFLV

>BetegovirusSF|AHA86934.1|_1-420/1-254 gp1 [Betegovirus SF] _R_ BetegovirusSF_AHA86934.1__1-42__1-42_.prf _R_ BetegovirusSF_AHA86934.1__1-42__1-42_.prf

MMPNY-L--FE-AIFNR------------KVI---------------MS--T-----------PNQV--I--

-DIAVHFYSRTISYPLP-------------------------------------------------------

-------------------SKTT-------TLLALA------------AT-GLTMYATFTVA----------

----------KHITDA--------VHEAWDSFR--TYLIHTASRVHKPQEI----VRRTF------------

------------------------------------------------RETQFFHK--HADVNHTHGPSAAD

RSSASEFATNFAYSLGVTPFFIQMSAADQRHSKRGNRT-----YFWSKDVHVKPSNEKP-------------

--NPEDVRVLVDVDQYFDMP-YLL--ANDPAVY--LLYTFQPHKAASTCL-DYSFTFDADGRVNYQVNGGGS

F-RHHVWNYAKDVFTV

>PlasmoparaA_301070430_gb_/1-207 _R_ PlasmoparaA_3_1_7_43__gb__1-378.prf _R_ PlasmoparaA_3_1_7_43__gb__1-378.prf _R_ PlasmoparaA_3_1_7_43__gb__1-378.prf _R_ PlasmoparaA_3_1_7_43__gb__1-378.prf

MSFGT-L--FS-----R------------YTL---------------MV--S--------------------

------------------------------------------------------------------------

---------------------------------SVS------------VV-GLATPWRYVMS----------

----------KTLNE----------------FG--EEWS-------IEPAN----LRSLF------------

------------------------------------------------GDIQVVEF--KRQPGHPHGAAAAD

RSAGSYFIERFARDVGREVVYYQCSNSDLRNGRSGARE-----WYWMKDAYVQPKLFSL-------------

--SSSSMVSIIDVDQYVDMN-WFL--TEHFCPV--LLYTFQPSVCAADRG-EYSYTFNSNNEVIYRVAGGAE

Y-VHKVWNYDTDVVTT

>Tetnovirus2_302171585_gb_/1-202 _R_ Tetnovirus2_3_2171585_gb__1-368.prf _R_ Tetnovirus2_3_2171585_gb__1-368.prf _R_ Tetnovirus2_3_2171585_gb__1-368.prf _R_ Tetnovirus2_3_2171585_gb__1-368.prf

MQLQN-Y--HL---K-R------------VY----------------APNPL-QVV-------VH-------

------------------------------------------------------------------------

---------------------------------------------------------------K--------

----------------LLPRSAIRP-----------------------------------------------

-----------------------E------KVIV--RYKLSK--MDYLRHIRLAPPTQEANKLHPHPMSAEL

RRRALQAVRYMCKQLNKQMHVISPGPNESLLADASTRY-----HYWARDLMVAESNHPL-------------

--THDSLLYLQDVDYYTDMN-YIL---GAGLPV--VLYTIIPRKAASRKQ-EYAYRFE-NGQFLFTSNGGTA

F-SHLLWDYDLDTCAI

>Tetnovirus1_TETNOVIRUS_30/1-312 _R_ Tetnovirus1_TETNOVIRUS_3__1-474.prf _R_ Tetnovirus1_TETNOVIRUS_3__1-474.prf _R_ Tetnovirus1_TETNOVIRUS_3__1-474.prf _R_ Tetnovirus1_TETNOVIRUS_3__1-474.prf

MLYNI-A--HD---L-TIL---------GYDI---------------SERSLSHPRIIVRPFLTELVSVLRD

VAK----EALI--TP---------------------------------------------------------

-------------------VI-V-------PT-TLT------------GS-LM-AYVYYQVA-NT-------

-YDQHDNPLIRIPMNCIYTLSICFPYRTN-----------ISL-N-HKLSDDLPLIRDYYGMPIT--QKDWP

TSNVG---------WYR-R-YFGTLRPNDQRLIYRYPRCPRS---I-PSLVRDYDW--KLESVSPHPEEAAQ

RNAARFNLPSMALSMGYTPFTISGRPSEA---TLGFNL-----VYNYRDTERLLRGDVL-------------

--TDRNAIILVDTDYYIDMP-KLL---TTGLPI--LMYTFVPQRLAGSTQ-GTNYYIDKHSQVHFRAAGGTT

W-NHYLWDYNHDYLTS

>ChronicBee_188572672_ref_/1-314 _R_ ChronicBee_188572672_ref__1-514.prf _R_ ChronicBee_188572672_ref__1-514.prf

MNLSN-V--ND-VLS-S------------VSSTLRPGEGGMRQIEYIFD---P----------PSLPTRI--

-YNSVHDWFYPPLHPIAAAIK-AAYTNFGVQTKAAATAV---------------------------------

------------------VNA-TVPKTVISGLKICASSIGVTIVICSAG---------YW------------

----------------------------FYRTR--HLK-IVQVTN-NGTMCQINELREYFET----------

------------------------------------------LCGPA-GAHM-DLP--DSIPGH-HLRIAAE

RRFLERACCEVFQQIGVRFRDVGGSRVRG--EDYAHLKHLCNPCIDSSDILRDAKNPEPPFSSCGGRGEVCP

SRHDFPGALLCHSDYYLYDELSYV--IRGHTFI--INHSFLEENKHF-HN-EAFITKK-GDGVIMSTPDGTE

Y-QHPYNVWKAEGCII

>Anopheline-associated_1-5/1-316 _R_ Anopheline-associated_1-5_1-516.prf _R_ Anopheline-associated_1-5_1-516.prf

MDIKK-I--IE-HLD--------------------PKNETFYSIEQAFD---P----------SPIWVRA--

-TRWFRVRFSPEPNLLVRGIKTAANSLVGGIPRVRNGAV---------------------------------

------------------NGA-GHAKTVARAAWDTAKYHSTVLVISAVGTCAA-IYAATW------------

----------------------------IWRSR--RVL-FVQVIN-DGTMLPIPTLKDHFAT----------

------------------------------------------RLSKR-P--E-DYG--PTLPGL-HLRIAAE

RRRAEQAVFEVFDDIKLRFRDVGGSLSRG--KDYVQLKHVCNPVLDSADILRRAKADECPFDTCKGVGQKCP

QRAAFPGALLCHSDYYMDQE-DLVACVRSHTFV--INHRYTADSKVNHLG-EMEVAVE-GPYVKGGTSDGTR

Y-SHAYNLWQAEGSVV

>LakeSinai1_335057597_gb_A/1-348 _R_ LakeSinai1_335_57597_gb_A_1-544.prf _R_ LakeSinai1_335_57597_gb_A_1-544.prf

MVKLA-F--AA-F---V------------SLY-------------IICQ--V-----------PPISYWI--

-EDLIDDFDTMCSHEYAAA---DAYNNFVHRHRVAAYAAGVRIHRYRTPWYCAASRSVPVVHPLNWLMTQYD

LTTGHVRELLDRLEVVNVTLR-D-------GLRTVA------------DT-AFTAYMYYQIM-W--------

CQ-----LALIVLLVG--------LFSAIICSS--RVR-VVRVRN-RVRGYSITDLRNEFD-----------

---------------------------------------------ES-TSDI---T--LPLSR-GHSCLNFQ

RRVAESWCLDQLLRYFHGVRFISASTGRW--AELGHRIHRCSPVVMDGSFVPEAT---HHGVLCRRQPSACR

DRFEIPACVISHADYYMSPD-QLA--STVTGPTFIINHDYLAPD-SLSVA-EATTRVA-NGHVTSSVREGPI

YGPHPYYLWNNEGVVV

>LakeSinai2_335057590_gb_A/1-347 _R_ LakeSinai2_335_5759__gb_A_1-543.prf _R_ LakeSinai2_335_5759__gb_A_1-543.prf

MKVVF-L--V--S---F------------AVF-------------LICQ--V-----------PPVSYWL--

-EDALDEYNTMCSYEYAAA---EAYNNFIHKHRVAAYAAGVRILRYRTPWYCFASRSVPVVHPFSWLMGQYD

ATIDRVGTLMQRLEIANVSIS-D-------GLRTVA------------DT-ALKAYFYYQII-W--------

FQ-----LFSIIMLVG--------FFSAVICSS--RVR-VVRIRN-RVRGYSVAELRSDFE-----------

---------------------------------------------ES-MSAI---L--APLSR-GHSCLNFQ

RRVVESWAIDQLLRYFRCFRSVASSQGRW--AEVGHRLHRCSPVVMDGAFVPEYD---QRFSACRRHPSVCP

DRFDTPAAILSHVDYYMTPD-QLA--AAVTGPTFIVNHDYSSID-TLSVA-EVSLRSA-GGLVTASVRDGPT

FGPHPYYRWSDEGVVV

**Alphavirus+Nodavirus supergroups**

>Sindbis_ALPHAVIRUS|25121506|ref|NP_740670.1|/39-213 nsp1 nonstructural protein [Sindbis virus] _R_ Sindbis_ALPHAVIRUS_251215_6_ref_NP_74_67_.1__1-54_.prf _R_ Sindbis_ALPHAVIRUS_251215_6_ref_NP_74_67_.1__1-54_.prf

HANARAFSHLAS-KLIELE-VP---------TTATIL--DIGSAP--ARR--MFSE------H----QYHCV

CPMRSPEDPDRMMKYASKLAEK-A-CKITNKNLHEKIKDLRTVLDTPDAE----------------------

-----------------------------------------------------TPSLCFH-NDVTC--NM--

R--------------AEYSVMQDVYIN-APGTIY-HQAM-K-GVRTLYWIGFDTTQ--FMFSAMA--GSYP-

AYNTNWADEKVLEARNIGLCSTKLSEGRTGKLSIMRKK--E-------------------------------

---------------------LK--P--------------------------------------------GS

R--VY----FS-VGSTLY-PE

>Rubella_RUBIVIRUS_3362846/66-174 _R_ Rubella_RUBIVIRUS_3362846_1-445.prf _R_ Rubella_RUBIVIRUS_3362846_1-445.prf _R_ Rubella_RUBIVIRUS_3362846_1-445.prf

HPALHAISRYTR-RHWIEW-GP---------KEALHV--LIDPSPGLLRE--VARVE-----RR---WVALC

L-HRTAR----------KL-----------------------ATAL--AE----------------------

-----------------------------------------------------TASEAWH-ADYVC--ALRG

APSGPFYVHPEDVPHGGRAVADRCLLYYTPMQMC-ELMRTI-DATLLVAVD--LWPVALAAH-VG--DDWD-

DL---------------------------------GIA--W-------------------------------

---------------------HL--DH-------------------------------------------DG

GC-PADCRGAGAGPTPGY-TR

>HepatitisE_HEPEVIRUS|9626448|ref|NP_056779.1|/65-167 polyprotein [Hepatitis E virus] _R_ HepatitisE_HEPEVIRUS_9626448_ref_NP__56779.1__1-431.prf _R_ HepatitisE_HEPEVIRUS_9626448_ref_NP__56779.1__1-431.prf

HPIQRVIHNELE-LYCRAR-SG------------RCL--EIGAHP--R-S--INDN------PN---VVHRC

FLRPAGRDVQRWYTAPTRGPAA----NCRR----------SALR-GLPAA----------------------

-----------------------------------------------------DRTYCFD-GFSGC--NF--

PA-ETG-------------VALYSLHDMSPSDVA-EAMFRH-GMTRLYAAL--HLPPEVLLP-PG--TYRTA

-----------------------------------SYL----------------------------------

----------------------LIHD--------------------------------------------GR

R--VV-V-TYEGDTSAGY-NH

>CitrusTristeza_CLOSTEROVIRUS|26251497_1-450/91-172 replicase [Citrus tristeza virus] _R_ CitrusTristeza_CLOSTEROVIRUS_26251497_1-45__1-45_.prf _R_ CitrusTristeza_CLOSTEROVIRUS_26251497_1-45__1-45_.prf

HPVAAGSRALEN-HLVRKH-AG---------T--DYS--DVGGCP--LFH--LRAG------HS---GVHVC

RPVYDVKDAHRRVVRHHQLSKV-S-LDQSD----------GVKQVGWTVN----------------------

-----------------------------------------------------TNSVCGN-ILGEC--YH--

AS-EAM-------------VMV-QVYDVPLRELC-RAMINK-KTSVCYMTM--VTPGELLDA-RE--SFFIK

-----------------------------------DLDCSV-------------------------------

---------------------ELDPI--------------------------------------------AD

R--VV-----YCFNNSAY-TH

>Helicoverpa_OMEGATETRAVIRUS|9632271_1-419/61-226 RNA-dependent RNA polymerase [Helicoverpa armigera stunt virus] _R_ Helicoverpa_OMEGATETRAVIRUS_9632271_1-419_1-419.prf _R_ Helicoverpa_OMEGATETRAVIRUS_9632271_1-419_1-419.prf

HPILAGHQRVAE-EVLHNF-AR---------GRSTVL--EIGPSL--HSA--LKLHGAPNAPVA---DYHGC

T-KYGTRDGSRHITALESRSVATGRPEF----------KADASLLANGIA----------------------

-----------------------------------------------------SRTFCVD-GVGSC--AF--

--------------KSRVGIANHSLYDVTLEELA-NAFENH-GLHMVRAFM--HMPEELLYM-DN--VVNAE

L----------------------------------GYR--F-------------------------------

---------------------HV--IEEPMAVKDCAFQGGDLRLHFPELDFINESQERRIERLAARGSYSRR

A--VI-F-SGDDDWGDAY-LH

>BromeMosaic_BROMOVIRUS|9626932_1-428/80-172 replicase [Brome mosaic virus] _R_ gi_9626932_1-428_1-428.prf _R_ BromeMosaic_BROMOVIRUS_9626932_1-428_1-428.prf

HSLAGALRVAEH-YDCLDS-FP---------PEDPVI--DFGGSW--WHH--FSRR------DK---RVHSC

CPVLGVRDAARHEERMCRMRKILQE-------------------SDDFDE----------------------

-----------------------------------------------------VPNFCLN-RAQDC--DV--

--------------QADWAICIHGGYDMGFQGLC-DAMHSH-GVRVLRGTV--MFDGAMLFD-RE--GFLPL

L----------------------------------KCH--W-------------------------------

---------------------QR--DGS----------------------------------------GADE

V--IK-F-DFENESTLSY-IH

>TobaccoMosaic_TOBAMOVIRUS|9626126_1-460/81-192 Replicase [Tobacco mosaic virus] _R_ TobaccoMosaic_TOBAMOVIRUS_9626126_1-46__1-46_.prf _R_ TobaccoMosaic_TOBAMOVIRUS_9626126_1-46__1-46_.prf

HSLAGGLRSLEL-EYLMMQ-I---------PYGSLTY--DIGGNF--ASH--LFKG------RA---YVHCC

MPNLDVRDIMRHEGQKDSIELY-L-SRLERGGKTVPNFQKEAFDRYAEIP----------------------

-----------------------------------------------------EDAVCHN-TFQTM--RH--

QP-MQQ-------SGRVYAIALHSIYDIPADEFG-AALLRK-NVHTCYAAF--HFSENLLLE-DSYVNLDEI

-----------------------------------NAC----------------------------------

----------------------FSRD--------------------------------------------GD

K--LT-F-SFASESTLNY-CH

>CitrusLeprosis_CILEVIRUS|109255263|ref|YP_654565.1|/165-193 methyltransferase; MTR [Citrus leprosis virus C] _R_ CitrusLeprosis_CILEVIRUS_1_9255263_ref_YP_654565.1__1-4_3.prf _R_ CitrusLeprosis_CILEVIRUS_1_9255263_ref_YP_654565.1__1-4_3.prf

HNNAAALRRVLR-FYMRDKVGYRKDDDIPDGYHVKNK--DVGASG--MDV--IADE------LT---DVHCC

TPDLDFRDHIRLERLKKYIYSH-V-CPTSK-------DHGIICEGFREGS----------------------

-----------------------------------------------------TKYRCEN-IGQQC--YI--

E--------------APTLTFVHSAYDITAAGIV-DCMIAA-NAHHAIMCL--HFPSAILSG-ST--SGKDD

LL---------------------------------QYK--W-------------------------------

---------------------DIVVE--------------------------------------------DG

IKYYK-Q-KFLNDTQASY-VH

>BambooMosaic_POTEXVIRUS|9627984_1-500/68-148 replicase [Bamboo mosaic virus] _R_ BambooMosaic_POTEXVIRUS_9627984_1-5___1-348.prf _R_ BambooMosaic_POTEXVIRUS_9627984_1-5___1-348.prf

HAAAKALENDLY-RTASYLLPKE---------RVSFL--FMKPSK--MQY--FRR-R-----GD---VDTFI

NADIVAKDLARYPLET--------------------------------------------------------

----------------------------------------------------------IYPRLPEI--TT--

----------------KMAFIGDSLHYFQPEVLE-HIFTSSPQLETLLATI--VLPPEATLR-LK--SLYPE

I-----------------------------------YT--L-------------------------------

---------------------HY--M--------------------------------------------PQ

K--FL-Y-KPGGLSGGEY-EH

>BotrytisF_MYCOFLEXIVIRUS|81968259_1-500/69-151 RecName: Full=RNA replication protein; Includes: RecName: Full=RNA-directed RNA polymerase; Includes: RecName: Full=Helicase; Includes: RecName: Full=Methyltransferase _R_ BotrytisF_MYCOFLEXIVIRUS_81968259_1-5___1-358.prf _R_ BotrytisF_MYCOFLEXIVIRUS_81968259_1-5___1-358.prf

HPACYALNAFHF-LKVIPRYLHG---------KAEVW--GTKDNW--FRK--LQAQIDSQ--TT---TLTHR

NYAITARDHVRYPGTV--------------------------------------------------------

----------------------------------------------------------VD-HPGSC--TA--

----------------GTLFMHDALQYMTPLDVY-TLFATSPEMHSLVATA--VIPPESVDR-LP--AFWPE

L-----------------------------------YQ--L-------------------------------

---------------------AY--Y--------------------------------------------ED

H--LC-Y-APDGNFADAY-NQ

>PotatoM_CARLAVIRUS|9626090_1-500/72-176 polyprotein [Potato virus M] _R_ PotatoM_CARLAVIRUS_9626_9__1-5___1-39_.prf _R_ PotatoM_CARLAVIRUS_9626_9__1-5___1-39_.prf

HPVCKTLENYILYSVLPSY-IN---------S--SFYFVGIKERK--LQL--LKSK------CKNLDSVQVV

NRYVTSADRMRYTNDFVPYGS--Y-EHECL----------VHKGVGLDNE----------------------

-----------------------------------------------------ALRGLVG-PL--R--RH--

KA-KNL-------------FFHDELHYWSSKVLI-DFLDVM-RPDKLLGTV--VYPPELLFK-PT-RSLNEW

-----------------------------------CYT----------------------------------

----------------------YDIV--------------------------------------------GD

T--LM-F-FPDGVQSEGY-QQ

>TurnipYellow_TYMOVIRUS|21686952_1-500/67-145 replicase/papain-like protease [Turnip yellow mosaic virus] _R_ TurnipYellow_TYMOVIRUS_21686952_1-5___1-362.prf _R_ TurnipYellow_TYMOVIRUS_21686952_1-5___1-362.prf

HAAHKTIETFLL-CTHWSFQATT---------PSSVM--FMKPSK--FNK--LAQVN-----SN---FRELK

NYRLHPNDSTRYPFTS--------------------------------------------------------

----------------------------------------------------------PD----LP--VF--

----------------PTIFMHDALMYYHPSQIM-DLFLRKPNLERLYASL--VVPPEAHLS-DQ--SFYPK

L-----------------------------------YT--Y-------------------------------

---------------------TT--T--------------------------------------------RH

T--LH-Y-VPEGHEAGSY-NQ

>OysterMushroom_OSTREVIRUS|28261418_1-500/67-149 RNA-dependent RNA polymerase [Oyster mushroom spherical virus] _R_ OysterMushroom_28261418_1-5___1-353.prf _R_ OysterMushroom_28261418_1-5___1-353.prf

HVMLKALENHRNVNILPNY-LR---------G--RVTVLSMKPAK--LAA--LRAR------CPGV-QFNLV

NEIITPADHTRYTDPSPPP-----------------------------------------------------

--------------------------------------------------------------D--F--QG--

PG-DMI-------------LVDDALQHWSRERID-AAFRHF-GGNRLIGTN--IHPDEVRSG-HA--SRYPD

-----------------------------------LYT----------------------------------

----------------------IEYL-----------------------------P--------------NR

R--YG-L-LSHRHKSASY-EA

>GremmeniellaXL1_ENDORNAVIRUS|90403573_210-600/266-188 putative polyprotein [Gremmeniella abietina type B RNA virus XL1] _R_ GremmeniellaXL1_ENDORNAVIRUS_9_4_3573_21_-6___1-391.prf _R_ GremmeniellaXL1_ENDORNAVIRUS_9_4_3573_21_-6___1-391.prf

HIYHHANRLMLT-EMLASR-HP---------RNALLY--DIGGNI--NRH--LDHG------RL---NVHCV

YTTSTPADLSRHTLWVKKATQWAH-ANLKNKSVHGATYS-SIAASMIKED----------------------

-----------------------------------------------------NSMWCTN-GIANC--SH--

AP-----------ISVGFAMSIDTLVHLDPSDLI-KFYIDN-NVAHASHAM--TIPDNYAYA-TK--GVLRH

NE---------------------------------GHW--Y-------------------------------

---------------------RT--N--------------------------------------------NG

TW-VI----EFVGESLSY-EQ

>BeetNecrotic_BENYVIRUS|23821045_1-700/218-391 Replicase [contains methyltransferase, helicase and papain-like proteinase domains] [Beet necrotic yellow vein virus] _R_ BeetNecrotic_BENYVIRUS_23821_45_1-7___87-7__.prf _R_ BeetNecrotic_BENYVIRUS_23821_45_1-7___87-7__.prf

HPVLAALREVMR-QEYSAS-CN-----I-LNTKLKTL--VVGAAS--REVNCYS-S------NP---SVHYY

FANKDSKDLVRTTLELLHSALATKYRNM----------ESGERELMNNLKGCGYIVKRSVENAVYEVVSDKD

VAEVLRYAQTVASTKKEAKKKPNTGKRKMVMSEATRRTIELHELSRIVAEEKKIPNHFHF-DESDFASVG--

--------------NFTQLVCEDVGYNFSVDAWL-HLFEAT-GAQTAVGYM--ALPNELLFE-HY-----PI

------------------------------------SD-YYDYWEGVEKHGSLGGITISPLRNGQVVGMPTG

VFQPVHFDKTSAGLGIPGSKMG-AAE--------------------------------------------RV

I--CH----MSDGLGNGY-NH

>Nodamura_ALPHANODAVIRUS_9/107-125 _R_ Nodamura_ALPHANODAVIRUS_9_1-414.prf _R_ Nodamura_ALPHANODAVIRUS_9_1-414.prf

HPISGGTRDVAR-ETISLA-IR--------AAGFRHY--EISPAR--QSP--AE---------A---ASHQ-

--HYAAADLVRAAT------------ED----------K---------------------------------

--------------------------------------------------------------------IQ--

--------------DGDVVVAIDIDYYLRDIDRYLG----R-GVP-FMAYT--FNPVEVAGR-DG-------

-----------------------------------DSF--F-------------------------------

---------------------R-I-T--------------------------------------------NN

Q--VT----FDVSGGGSW-SH

>StripedJack_BETANODAVIRUS/76-124 _R_ StripedJack_BETANODAVIRUS_1-4_2.prf _R_ StripedJack_BETANODAVIRUS_1-4_2.prf

HQEAATERNSAT-DTMIGV-VE--------KCGYEPY--IISPSP--REK--EY---------H---GSRQ-

--FYSLADFRQDYR------------RD----------E---------------------------------

--------------------------------------------------------------------IT--

--------------DRHVIVMTDVDYYVDMHEL-VG----L-GVP-ILLYT--FQPSTVSGE-VK-------

-----------------------------------DGY--F-------------------------------

---------------------T-I-T--------------------------------------------DD

H--VH----YRVAGGKDV-RH

>Tetnovirus1/177-124 _R_ Tetnovirus1_TETNOVIRUS_3__1-474.prf _R_ Tetnovirus1_TETNOVIRUS_3__1-474.prf

HPEEAAQRNAAR-FNLPSM-AL--------SMGYTPF--TISGRP--SEAT------------L---GFNL-

--VYNYRDTERLLR------------GD----------V---------------------------------

--------------------------------------------------------------------LT--

--------------DRNAIILVDTDYYIDMPKLL-T----T-GLP-ILMYT--FVPQRLAGS-TQ-----GT

------------------------------------NY----------------------------------

---------------------Y-IDK--------------------------------------------HS

Q--VH----FRAAGGTTW-NH

>PlasmoparaA_301070430_gb_/68-128 _R_ PlasmoparaA_3_1_7_43__gb__1-378.prf _R_ PlasmoparaA_3_1_7_43__gb__1-378.prf

HGAAAADRSAGS-YFIERF-AR--------DVGREVVYYQCSNSD--LRN--GR---------S---GARE-

--WYWMKDAYVQPK------------LF----------S---------------------------------

--------------------------------------------------------------------LS--

--------------SSSMVSIIDVDQYVDMNWFLTE----H-FCP-VLLYT--FQPSVCAAD-RG-------

-----------------------------------EYS--Y-------------------------------

---------------------T-FNS--------------------------------------------NN

E--VI----YRVAGGAEY-VH

>SclerophthoraA_45476494_r/71-129 _R_ SclerophthoraA_45476494_r_1-375.prf _R_ SclerophthoraA_45476494_r_1-375.prf

HASAASLRTAAT-RYAQNV-AQ--------YCGTEVY--VVGMSR--SDQRKDM---------K---GSRQ-

--WYWTKDVNAANR------------ND----------R---------------------------------

--------------------------------------------------------------------PG--

--------------HRDIRFLCDVDYYVDMPAMLVA----E-AKP-VLLYT--VVPEEATSS-AN-----DE

-----------------------------------SAF----------------------------------

---------------------Y-FEA--------------------------------------------DG

S--LR----TMVAGGGGY-SH

>Tetnovirus2_302171585_gb_/65-126 _R_ Tetnovirus2_3_2171585_gb__1-368.prf _R_ Tetnovirus2_3_2171585_gb__1-368.prf

HPMSAELRRRAL-QAVRYM-CK--------QLNKQMH--VISPGP--NESLLAD---------A---STRY-

--HYWARDLMVAES------------NH----------P---------------------------------

--------------------------------------------------------------------LT--

--------------HDSLLYLQDVDYYTDMNYI-LG----A-GLP-VVLYT--IIPRKAASR-KQ-------

-----------------------------------EYA----------------------------------

---------------------Y-RFE--------------------------------------------NG

Q--FL----FTSNGGTAF-SH

>ChronicBee_CHROPARAVIRUS|188572672_ref_/158-145 _R_ ChronicBee_188572672_ref__1-44_.prf _R_ ChronicBee_188572672_ref__1-44_.prf

HLRIAAERRFLE-RACCEV-FQ--------QIGVRFR--DVGGSR--VRG--ED-Y------AH---LKHLC

NPCIDSSDILRDAKNPE--------------------------------P----------------------

-----------------------------------------------------PFSSCGG-RGEVC--PS--

RH-D-----------FPGALLCHSDYYLY-DELS-YVI-RG-HTF--------IINHSFLEE---NKHFHNE

------------------------------------AF----------------------------------

----------------------ITKK--------------------------------------------GD

G--VI----MSTPDGTEY-QH

>LakeSinai1_SINAIVIRUS|335057597_gb_A/193-144 _R_ LakeSinai1_335_57597_gb_A_1-47_.prf _R_ LakeSinai1_335_57597_gb_A_1-47_.prf

HSCLNFQRRVAE-SWCLDQLLR---------YFHGVR--FISAST--GRW--AELG------H----RIHRC

SPVVMDGSFV-----------------------------------PEATH----------------------

-----------------------------------------------------HGVLCRR-QPSAC--RD--

RF------------EIPACVISHADYYMSPDQLA-STVT---GPTF------------IINH-DY--LAPD-

SLS------------------------------VAEAT--T-------------------------------

---------------------RV--A--------------------------------------------NG

H--VT----SSVREGPIYGPH
