# Supplementary material for: Sequence analysis reveals a conserved extension in the capping enzyme of the alphavirus supergroup, and a homologous domain in nodaviruses
Source: Biol Direct. 2015 Apr 11;10:16. doi: 10.1186/s13062-015-0050-0 (PMC4392871; doi:10.1186/s13062-015-0050-0)
Supplement: Additional file 1: — Compilation of all supplementary figures and tables, in .zip format. [file 13062_2015_50_MOESM1_ESM.zip › Additional File 1/Table S1_Accession numbers.doc]

**Table S1: Accession numbers of proteins of the alphavirus and nodavirus supergroups**

| **ALPHAVIRUS SUPERGROUP** | | | | | | | | | | | | | | | | | | | | | | | | | | | |  | | | |
| --- | --- | --- | --- | --- | --- | --- | --- | --- | --- | --- | --- | --- | --- | --- | --- | --- | --- | --- | --- | --- | --- | --- | --- | --- | --- | --- | --- | --- | --- | --- | --- |
| **Group** | **Family** | | | | | | | | | **Genus** | | | | | **Species** ^(1)^ | | | **Species abbreviation** | | | | **Protein name** | | | | **Accession number** | |  | | | |
| Alto | Bromoviridae | | | | | | | | | Alfamovirus | | | | | Alfalfa mosaic | | |  | | | | Replicase | | | | NP_041192.1 | |  | | | |
|  |  |  |  |  |  |  |  |  |  | Anulavirus | | | | | Pelargonium zonate spot | | |  | | | | Replicase | | | | NP_619770.1 | |  | | | |
|  |  |  |  |  |  |  |  |  |  | Bromovirus | | | | | Brome mosaic | | |  | | | | Replicase | | | | NP_041196.1 | |  | | | |
|  |  |  |  |  |  |  |  |  |  | Cucumovirus | | | | | Cucumber mosaic | | |  | | | | Replicase | | | | NP_049323.1 | |  | | | |
|  |  |  |  |  |  |  |  |  |  | Ilarvirus | | | | | Tobacco streak | | |  | | | | Replicase | | | | NP_620772.1 | |  | | | |
|  |  |  |  |  |  |  |  |  |  | Oleavirus | | | | | Olive latent 2 | | |  | | | | Replicase | | | | NP_620042.1 | |  | | | |
|  | Closteroviridae | | | | | | | | | Ampelovirus | | | | | Grapevine leafroll-associated 3 | | |  | | | | Replicase | | | | NP_813795.3 | |  | | | |
|  |  |  |  |  |  |  |  |  |  | Closterovirus | | | | | Citrus tristeza | | |  | | | | Replicase | | | | NP_733947.1 | |  | | | |
|  |  |  |  |  |  |  |  |  |  |  | | | | | Beet yellows | | |  | | | | Polyprotein | | | | Q08534.2 | |  | | | |
|  |  |  |  |  |  |  |  |  |  | Crinivirus | | | | | Lettuce infectious yellows | | |  | | | | Replicase | | | | NP_733943.1 | |  | | | |
|  | Endornaviridae | | | | | | | | | Endornavirus | | | | | Gremmeniella Abietina type B RNA XL | | |  | | | | Polyprotein | | | | YP_529670.1 | |  | | | |
|  | Hepeviridae | | | | | | | | | Hepevirus^(2)^ | | | | | Hepatitis E | | |  | | | | Polyprotein | | | | NP_056779.1 | |  | | | |
|  |  |  |  |  |  |  |  |  |  |  |  |  |  |  | Rat hepatitis E | | |  | | | | Polyprotein | | | | ADM35754.1 | |  | | | |
|  |  |  |  |  |  |  |  |  |  |  |  |  |  |  | Ferret hepatitis E | | |  | | | | Polyprotein | | | | AFO38376.1 | |  | | | |
|  |  |  |  |  |  |  |  |  |  |  |  |  |  |  | Avian hepatitis E | | |  | | | | Polyprotein | | | | AEX93357.1 | |  | | | |
|  |  |  |  |  |  |  |  |  |  |  |  |  |  |  | Bat hepevirus | | |  | | | | Polyprotein | | | | YP_006576507.1 | |  | | | |
|  |  |  |  |  |  |  |  |  |  | Piscihepevirus^(2)^ | | | | | Cutthroat trout | | |  | | | | Polyprotein | | | | YP_004464917.1 | |  | | | |
|  | Tetraviridae | | | | | | | | | Betatetravirus | | | | | Nudaurelia capensis beta | | |  | | | | RdRp | | | | NP_048059.1 | |  | | | |
|  |  |  |  |  |  |  |  |  |  | Omegatetravirus | | | | | Helicoverpa armigera stunt | | |  | | | | RdRp | | | | NP_049235.1 | |  | | | |
|  | Virgaviridae | | | | | | | | | Hordeivirus | | | | | Barley stripe mosaic | | |  | | | | Alpha protein | | | | NP_604474.1 | |  | | | |
|  |  |  |  |  |  |  |  |  |  | Furovirus | | | | | Soil-borne wheat mosaic | | |  | | | | Polyprotein | | | | NP_049336.1 | |  | | | |
|  |  |  |  |  |  |  |  |  |  | Pecluvirus | | | | | Indian peanut clump | | |  | | | | Replicase | | | | NP_835261.1 | |  | | | |
|  |  |  |  |  |  |  |  |  |  | Pomovirus | | | | | Potato mop-top | | |  | | | | Polymerase | | | | NP_620436.1 | |  | | | |
|  |  |  |  |  |  |  |  |  |  | Tobamovirus | | | | | Tobacco mosaic | | |  | | | | Replicase | | | | NP_056764.1 | |  | | | |
|  |  |  |  |  |  |  |  |  |  | Tobravirus | | | | | Tobacco rattle | | |  | | | |  | | | | NP_620670.1 | |  | | | |
|  | Togaviridae | | | | | | | | | Alphavirus | | | | | Sindbis | | |  | | | | nsP1 | | | | NP_740670.1 | |  | | | |
|  |  |  |  |  |  |  |  |  |  |  | | | | | Semliki forest | | | SFV | | | | nsP1 | | | | NP_740665.1 | |  | | | |
|  |  |  |  |  |  |  |  |  |  | Rubivirus | | | | | Rubella | | |  | | | | nsPolyprotein | | | | NP_062883.2 | |  | | | |
|  | Unclassified | | | | | | | | | Benyvirus | | | | | Beet necrotic yellow vein | | |  | | | | Polyprotein | | | | NP_612615.1 | |  | | | |
|  | Unclassified | | | | | | | | | Unclassified | | | | | Chara Australis virus | | |  | | | | Replicase | | | | AEJ33768.1 | |  | | | |
|  | Unclassified | | | | | | | | | Cilevirus | | | | | Citrus leprosis C | | |  | | | | Replicase | | | | YP_654538.1 | |  | | | |
|  | Unclassified | | | | | | | | | Higrevirus | | | | | Hibiscus green spot | | |  | | | | Polyprotein | | | | YP_004928118.1 | |  | | | |
|  | Unclassified | | | | | | | | | Mycohepevirus^(2)^ | | | | | Sclerotinia sclerotiorum RNA L | | |  | | | | Polyprotein | | | | ACE88957.1 | |  | | | |
|  | Unclassified | | | | | | | | | Negevirus | | | | | Negev | | |  | | | | Polyprotein | | | | AFI24684.1 | |  | | | |
|  | Unclassified | | | | | | | | | Idaeovirus | | | | | Raspberry bushy dwarf | | |  | | | | Polyprotein | | | | NP_620465.1 | |  | | | |
|  | Unclassified | | | | | | | | | Pteridovirus | | | | | Japanese holly fern mottle | | |  | | | | Polyprotein | | | | YP_003126903.1 | |  | | | |
| **Group** | | | **Family** | | **Genus** | | | | | | | **Species** ^(1)^ | | | | **Species abbreviation** | | | | **Protein name** | | | | **Accession number** | | |  | | | | |
| Tymo | Alphaflexiviridae | | | | | | | | | | Allexivirus | | | | Shallot X | | |  | | | | Replicase | | | | NP_620648.1 | |  | | | |
|  |  |  |  |  |  |  |  |  |  |  | Botrexvirus | | | | Botrytis X | | |  | | | | Replicase | | | | NP_932306.1 | |  | | | |
|  |  |  |  |  |  |  |  |  |  |  | Lolavirus | | | | Lolium latent | | |  | | | | Replicase | | | | YP_001718499.1 | |  | | | |
|  |  |  |  |  |  |  |  |  |  |  | Mandarivirus | | | | Indian citrus ringspot | | |  | | | | RdRp | | | | NP_203553.1 | |  | | | |
|  |  |  |  |  |  |  |  |  |  |  | Potexvirus | | | | Bamboo mosaic | | | BaMV | | | | Replicase | | | | NP_042582.1 | |  | | | |
|  |  |  |  |  |  |  |  |  |  |  | Sclerodernavirus | | | | Sclerotinia sclerotiorum debilitation-associated RNA | | |  | | | | Polyprotein | | | | YP_325662.1 | |  | | | |
|  | Betaflexiviridae | | | | | | | | | | Capillovirus | | | | Apple stem grooving | | |  | | | | Polyprotein | | | | NP_044335.1 | |  | | | |
|  |  |  |  |  |  |  |  |  |  |  | Carlavirus | | | | Potato M | | |  | | | | Polyprotein | | | | NP_056767.1 | |  | | | |
|  |  |  |  |  |  |  |  |  |  |  | Citrivirus | | | | Citrus leaf blotch | | |  | | | | Replicase | | | | NP_624333.1 | |  | | | |
|  |  |  |  |  |  |  |  |  |  |  | Foveavirus | | | | Apple stem pitting | | |  | | | |  | | | |  | |  | | | |
|  |  |  |  |  |  |  |  |  |  |  | Tepovirus | | | | Potato T | | |  | | | | Replicase | | | | YP_002019748.1 | |  | | | |
|  |  |  |  |  |  |  |  |  |  |  | Trichovirus | | | | Apple chlorotic leaf spot | | |  | | | | Replicase | | | | NP_040551.1 | |  | | | |
|  |  |  |  |  |  |  |  |  |  |  | Vitivirus | | | | Grapevine A | | |  | | | | Replicase | | | | AAO17778.1 | |  | | | |
|  | Gammaflexiviridae | | | | | | | | | | Mycoflexivirus | | | | Botrytis F | | |  | | | | Replicase | | | | NP_068549.1 | |  | | | |
|  | Tymoviridae | | | | | | | | | | Maculavirus | | | | Grapevine fleck | | |  | | | | Replicase | | | | NP_542612.1 | |  | | | |
|  |  | | | | | | | | | | Marafivirus | | | | Maize rayado fino | | |  | | | | Polyprotein | | | | NP_115454.1 | |  | | | |
|  |  | | | | | | | | | | Tymovirus | | | | Turnip yellow mosaic | | |  | | | | Polyprotein | | | | NP_663297.1 | |  | | | |
|  | Unclassified | | | | | | | | | | Unclassified | | | | Oyster mushroom spherical | | |  | | | | RdRp | | | | NP_783203.1 | |  | | | |
|  | Unclassified | | | | | | | | | | Unclassified | | | | Donkey orchid symptomless | | |  | | | | Replicase | | | | YP_008828152 | |  | | | |
| **NODAVIRUS SUPERGROUP** | | | | | | | | | | | | | | | | | | | | | | | | | | | |  | | | |
| **Group** | | | **Family** | | **Genus** | | | | | | | **Species** ^(1)^ | | | | **Species abbreviation** | | | | **Protein name** | | | | **Accession number** | | | |  | | | |
| Chropara | | | Unclassified | | Chroparavirus^(2)^ | | | | | | | Chronic bee paralysis virus | | | | CBPV | | | | ORF1 | | | | YP_001911136.1 | | | |  | | | |
|  |  |  |  |  | Unclassified | | | | | | | Anopheline-Associated virus C | | | | AAVC | | | | RdRp | | | | AGW51752.1 | | | |  | | | |
|  |  |  |  |  | Sinaivirus^(2)^ | | | | | | | Lake Sinai virus 1 | | | |  | | | | ORF1 | | | | AEH26192.1 | | | |  | | | |
|  |  |  |  |  | Sinaivirus^(2)^ | | | | | | | Lake Sinai virus 2 | | | |  | | | | ORF1 | | | | AEH26187.1 | | | |  | | | |
| Noda | | | Nodaviridae | | Alphanodavirus | | | | | | | Alphanodavirus HB-2007/CHN | | | |  | | | |  | | | | ADF97523.1 | | | |  | | | |
|  |  |  |  |  |  |  |  |  |  |  |  | Flock House | | | |  | | | | Protein A | | | | AEQ39075.1 | | | |  | | | |
|  |  |  |  |  |  |  |  |  |  |  |  | Nodamura | | | |  | | | | Protein A | | | | NP_077730.1 | | | |  | | | |
|  |  |  |  |  |  |  |  |  |  |  |  | Pariacoto | | | |  | | | | Protein A | | | | NP_620109.1 | | | |  | | | |
|  |  |  |  |  | Betanodavirus | | | | | | | Striped jack nervous necrosis | | | |  | | | | Protein A | | | | NP_599247.1 | | | |  | | | |
|  |  |  |  |  | Unclassified | | | | | | | Macrobrachium Rosenbergii nodavirus | | | |  | | | | Protein A | | | | NP_919036.1 | | | |  | | | |
|  |  |  |  |  | Unclassified | | | | | | | Wuhan Nodavirus  (also called “Pieris rapae virus”) | | | |  | | | | Protein A | | | | AAY27743.1 | | | |  | | | |
|  |  |  | Unclassified | | Unclassified | | | | | | | Plasmopara Halstedii A | | | |  | | | | RdRp | | | | ADK55577.1 | | | |  | | | |
|  |  |  | Unclassified | | Unclassified | | | | | | | Tetnovirus 1 | | | |  | | | | RdRp | | | | ADK97710.1 | | | |  | | | |
|  |  |  | Unclassified | | Unclassified | | | | | | | Tetnovirus 2 | | | |  | | | | RdRp | | | | ADK97710.1 | | | |  | | | |
|  | | |  | | |  | | | | | |  | | | |  | | | |  | | | |  | | | |  | | | |
| **NODAVIRUS SUPERGROUP (CONTINUED)** | | | | | | | | | | | | | | | | | | | | | | | | | | | |  | | | |
|  | | | |  | | | |  | | | | | |  | | |  | | | |  | | | |  | | | | | |  |
| **Group** | | | | **Family** | | | | **Genus** | | | | | | **Species** ^(1)^ | | | **Species abbreviation** | | | | **Protein name** | | | | **Accession number** | | | | | |  |
| Noda  (continued) | | | | Unclassified | | | Unclassified | | | | | | | Sclerophthora Macrospora A | | |  | | | | RdRp | | | | NP_995577.1 | | | | | |  |
|  |  |  |  | Unclassified | | | Unclassified | | | | | | | Bat Guano associated nodavirus GF-4n | | |  | | | | Protein A | | | | ADI48250.1 | | | | | |  |
|  |  |  |  | Unclassified | | | Unclassified | | | | | | | Betegovirus SF | | |  | | | | gp1 | | | | AHA86934.1 | | | | | |  |
| **UNCLASSIFIED, RELATED TO NODAVIRUS SUPERGROUP** | | | | | | | | | | | | | | | | | | | | | | | | | | | | |  | | |
| **Group** | | | | **Family** | | | | **Genus** | | | | | | **Species** ^(1)^ | | | **Species abbreviation** | | | | **Protein name** | | | | **Accession number** | | | | | |  |
| Santeuil | | Unclassified | | | | | | | Unclassified | | | | Le Blanc nodavirus ^(3)^ | | | | | |  | | RdRp | |  | | | | | | |  | |
|  |  | Unclassified | | | | | | | Unclassified | | | | Orsay virus ^(3)^ | | | | | |  | | RdRp | | ADW54428.2 | | | | | | |  | |
|  |  | Unclassified | | | | | | | Unclassified | | | | Santeuil nodavirus ^(3)^ | | | | | |  | | RdRp | | AFU90716.1 | | | | | | |  | |

Abbreviations: RdRp, RNA-dependent RNA Polymerase ;ns, non-structural

(1) the term “virus” is omitted from the species name

(2) proposed name

(3) Based on our results, we think that the term “nodavirus” should be removed from the species names (.e.g. “Santeuil virus” instead of “Santeuil nodavirus”).
