# Supplementary material for: Sequence analysis reveals a conserved extension in the capping enzyme of the alphavirus supergroup, and a homologous domain in nodaviruses
Source: Biol Direct. 2015 Apr 11;10:16. doi: 10.1186/s13062-015-0050-0 (PMC4392871; doi:10.1186/s13062-015-0050-0)
Supplement: Additional file 1: — Compilation of all supplementary figures and tables, in .zip format. [file 13062_2015_50_MOESM1_ESM.zip › Additional File 1/Tables S8 Membrane-binding regions.doc]

**Table S8A: Predicted amphipathic, membrane-binding α-helices in the MTase-GTase of the Alto group**

| **Family**^(1)^ | **Genus**^(2)^ | **Species** ^(2)^ | **Accession number** | **<** | **AMPHIPASEEK prediction** | **>** | **<** | **HELIQUEST prediction** | **>** |
| --- | --- | --- | --- | --- | --- | --- | --- | --- | --- |
|  |  |  |  | **Boundary**  **(aas)** | **Location within**  **MTase-GTase** | **Sequence** | **Boundary**  **(aas)** | **Location within**  **MTase-GTase** | **Sequence**^(3)^ |
| Bromo | Alfamo | Alfalfa mosaic | NP_041192.1 | - | - | - | - | - | - |
|  | Anula | Pelargonium zonate spot | NP_619770.1 | - | - | - | - | - | - |
|  | Bromo | Brome mosaic | NP_041196.1 | 421-425 | αI | HHFKT | 416-433 | αI | WKGWCHHFKTRFWWGGDS |
|  | Cucumo | Cucumber mosaic | NP_049323.1 | 453-457 | αI | FAALT | 446-468 | αI | ASSISKWFAALTRPLRVFFSSVV |
|  | Ilar | Tobacco streak | NP_620772.1 | 485-493 | Downstream  of αI | PHFTDVIKN | - | - | - |
|  | Olea | Olive latent 2 | NP_620042.1 | 404-416 | αI | NLWSWFKKKFELK | 403-423 | αI | RNLWSWFKKKFELKAHAFLFA |
| Clostero | Ampelo | Grapevine leafroll-associated 3 | NP_813795.3 | - |  |  | 828-853 | αI | SLAGVFKLFFQTVGDCFSNAVSVYAK |
|  | Clostero | Citrus tristeza | NP_733947.1 | - | - | - |  |  |  |
|  | Crini | Lettuce infectious yellows | NP_733943.1 | 434 |  | M | 419-445 | αI | SHYKPSIVNIIFRLIMHFLGGTKRFIY |
| Endorna | Endorna | fRNA XL | YP_529670.1 | 330-332  632-639 | αC  αI | ATQ  LWTAVKNW | 628-645 | αI | FLTGLWTAVKNWGRDFGS |
| Hepe | Orthohepe | Hepatitis E | NP_056779.1 | 410-416 | αI | LYSWLFE | - | - | - |
|  | Piscihepe | Cutthroat trout | YP_004464917.1 | 403-408 | αI | WQRICI | - | - | - |
| Tetra | Betatetra | Nudaurelia capensis beta | NP_048059.1 | 423-430 | αI | MRRIANWF | 417-436 | αI | ALLPVWMRRIANWFQDKFQI |
|  | Omegatetra | Helicoverpa armigera stunt | NP_049235.1 | - | - | - | 454-472 | αI | TSFWESLKHVLGSCCGLRN |
| Virga | Hordei | Barley stripe mosaic | NP_604474.1 | - | - | - | - | - | - |
|  | Furo | Soil-borne wheat mosaic | NP_049336.1 | - | - | - | 291-312 | αE’ | YKHNLNQLMKYLTKTFVVAKGG |
|  |  |  |  | - | - | - | 471-491 | αI | TLAHATFLKMFGKVSYYQRAL |
|  | Peclu | Indian peanut clump | NP_835261.1 | - | - | - | 478-495 | αI | SLKRIFKGVFEKFFGSVS |
|  | Pomo | Potato mop-top | NP_620436.1 | - | - | - | 297-314 | αE’ | HNLNSLMLYVTKTFVKAA |
|  | Tobamo | Tobacco mosaic | NP_056764.1 | - | - | - | 268-286 | αE’ | LNYCHSYSNILKYVCKTYF |
|  | Tobra | Tobacco rattle | NP_620670.1 | - | - | - | 464-482 | αI | TSLTDVLGLISRKMWKQ |
| Toga | Alpha | Sindbis | NP_740670.1 | 252-262 | αE’ | HRASLQSWHLP | - | - | - |
|  | Rubi | Rubella | NP_062883.2 | 23-28 | Upstream of MTase-GTase | VRDHVR | - | - | - |
|  |  |  |  | 423-434 | αI | DTIWDAIKRFLG | - | - | - |
| - | Beny | Beet necrotic yellow vein | NP_612615.1 | - | - | - | 515-532 | αE’ | GLGNGYNHVKSDWQTLLK |
| - | - | Chara Australis | AEJ33768.1 | - | - | - | - | - | - |
| - | Cile | Citrus leprosis C | YP_654538.1 | - | - | - | - | - | - |
| - | Higre | Hibiscus green spot | YP_004928118.1 | - | - | - | 549-668 | αI | RSASSGIVRCFSKLCDKLAS |
|  | Bluner | Blueberry ringspot necrotic virus | YP_004901700.1 | - | - | - | 466-485 | αH | SIYHCLWRRFFEQGKTVSSM |
| - | Mycohepe | Sclerotinia sclerotiorum RNA L | ACE88957.1 | - | - | - | - | - | - |
| - | Nege | Negev | AFI24684.1 | 3-4 | Upstream of MTase-GTase | SL | - | - | - |
|  |  |  |  | 521 | αI | R | 522-549 | αI and downstream | LHIRLPWNHRVQKFQQKMDRLIDEMKSF |
| - | Idaeo | Raspberry bushy dwarf | NP_620465.1 | - | - | - | 433-455 | αE’ | YRHDWEVYSKYLTSTVVSCGKHF |
|  |  |  |  | - | - | - | 627-646 | αI | LGSWVSSLKRVFYRILGFPA |
| - | Pterido | Japanese holly fern mottle | YP_003126903.1 | - | - | - | 298-315 | Long insertion between αC and βB’ | VSAYSSMSRRVKATLKEI |
|  |  |  |  | 413-419 | Long insertion between αC and βB’ | EGVNSVV | - | - | - |
|  |  |  |  | 764-783 | αI | SNFVSALKDIVTSFLKFVLP | 762-782 | αI | HFSNFVSALKDIVTSFLKFVL |

(1) The term “viridae” is omitted from the name of the family

(2) The term “virus” is omitted from the name of the genus and of the species

(3) In the last row (sequence of amphipathic helices predicted by Heliquest), any helix predicted by Amphipaseek (7^th^ row) is underlined.

**Table S8B: Predicted amphipathic, membrane-binding α-helices in the Tymo group**

| **Family**^(1)^ | **Genus**^(2)^ | **Species** ^(2)^ | **Accession number** | **<** | **AMPHIPASEEK prediction** | **>** | **<** | **HELIQUEST prediction** | **>** |
| --- | --- | --- | --- | --- | --- | --- | --- | --- | --- |
|  |  |  |  | **Boundary**  **(aas)** | **Location within**  **MTase-GTase** | **Sequence** | **Boundary**  **(aas)** | **Location within**  **MTase-GTase** | **Sequence**^(3)^ |
| Alphaflexi | Allexi | Shallot X | NP_620648.1 | 385-389 | αI | MRLRT | 374-395 |  | NLFGKMCASIRMRLRTFFQNLL |
|  | Botrex | Botrytis X | NP_932306.1 | - | - | - | - | - | - |
|  | Lola | Lolium latent | YP_001718499.1 | - | - | - | - | - | - |
|  | Mandari | Indian citrus ringspot | NP_203553.1 | - | - | - | - | - | - |
|  | Potex | Bamboo mosaic | NP_042582.1 | 366-368 | αI | PAI | 358-379 | αI | NILKRLFRPAIAKIQELRHWVS |
|  | Scleroderna | Sclerotinia sclerotiorum debilitation-associated RNA | YP_325662.1 | 602-612 | αI | VSPLKQWFSEI | 602-619 | αI | VSPLKQWFSEICEKFLGK |
| Betaflexi | Capillo | Apple stem grooving | NP_044335.1 | - | - | - | - | - | - |
|  | Carla | Potato M | NP_056767.1 | - |  |  |  |  |  |
|  | Citri | Citrus leaf blotch | NP_624333.1 | - | -- | - | - | - | - |
|  | Fovea | Apple stem pitting |  | - | - | - | - | - | - |
|  | Tepo | Potato T | YP_002019748.1 | - | - | - | - | - | - |
|  | Tricho | Apple chlorotic leaf spot | NP_040551.1 | - | - | - | - | - | - |
|  | Viti | Grapevine A | AAO17778.1 | - |  |  |  |  |  |
| Gammaflexi | Mycoflexi | Botrytis F | NP_068549.1 | - | - | - | - | - | - |
| Tymo | Macula | Grapevine fleck | NP_542612.1 | - | - | - | - | - | - |
|  | Marafi | Maize rayado fino | NP_115454.1 | - | - | - | - | - | - |
|  | Tymo | Turnip yellow mosaic | NP_663297.1 | - | - | - | 372-401 | same location as αI, but devoid of predicted secondary structure in *Tymoviridae* | LQSPIASLSLYLRQHWRRLTATAVPILSFL |
| - | Ostre^(4)^ | Oyster mushroom spherical | NP_783203.1 | 371-378 | αI | EWLPDWLF | 357-374 | - | GLSRLRFFIRRKAEEWLP |
| - | Dorsy^(4)^ | Donkey orchid symptomless | YP_008828152 | - | - | - | - | - | - |

(1) The term “viridae” is omitted from the name of the family

(2) The term “virus” is omitted from the name of the genus and of the species

(3) In the last row (sequence of amphipathic helices predicted by Heliquest), any helix predicted by Amphipaseek (7^th^ row) is underlined.

(4) Proposed name

**Table S8C: Predicted amphipathic, membrane-binding α-helices in the Noda, Chropara and Santeuil groups**

| **Group**^(1)^ | **Genus**^(2)^ | **Species** ^(2)^ | **Accession number** | **<** | **AMPHIPASEEK prediction** | **>** | **<** | **HELIQUEST prediction** | **>** |
| --- | --- | --- | --- | --- | --- | --- | --- | --- | --- |
|  |  |  |  | **Boundary**  **(aas)** | **Location within**  **MTase-GTase** | **Sequence** | **Boundary**  **(aas)** | **Location within**  **MTase-GTase** | **Sequence**^(3)^ |
| **Noda**  **Noda**  (continued) | Alphanoda | Alphanoda HB-2007/CHN | ADF97523.1 | - | - | - | -- | - | - |
|  |  | Flock House | AEQ39075.1 | - | - | - | -- | - | - |
|  |  | Nodamura | NP_077730.1 | - | - | - | 16-33 | Upstream of Core | NIVSRALGYRVPLAKSLA |
|  |  | Pariacoto | NP_620109.1 | 46-47 | Upstream of Core | HE |  |  |  |
|  | Betanoda | Striped jack nervous necrosis | NP_599247.1 | - | - | - | - | - | - |
|  |  | Epinephelus tauvina (=greasy grouper) nervous necrosis | NP_689433.1 | - | - | - | 210-227 | βH and variable region between βH and βI |  |
|  |  | Atlantic halibut nodavirus | AAY34458.1 | - | - | - | 210-227 | βH and variable region between βH and βI |  |
|  | Unclassified | Macrobrachium Rosenbergii noda | NP_919036.1 | - | - | - | -- | - | - |
|  |  | Wuhan Noda  (also called “Pieris rapae ”) | AAY27743.1 | - | - | - | 16-42 | Upstream of Core | TVKCVCVGVRQQYGLLRRHPKTIACAL |
|  |  |  |  | 95-101 | Upstream of Core | NCWTIFG | 86-103 | Upstream of Core | RFTAAKWRWNCWTIFGKF |
|  |  | Plasmopara Halstedii A | ADK55577.1 | 4 | Upstream of Core | G |  |  |  |
|  |  |  |  | 216-217 | Variable region between βH and βI | WT |  |  |  |
|  | Unclassified | Tetno 1 | ADK97710.1 | - | - | - | -- | - | - |
|  |  | Tetno 2 | ADK97710.1 | - | - | - | -- | - | - |
|  |  | Sclerophthora Macrospora A | NP_995577.1 | - | - | - | -- | - | - |
|  |  | Bat Guano associated noda GF-4n | ADI48250.1 | - | - | - | -- | - | - |
|  |  | Betego SF | AHA86934.1 | 4-7 | Upstream of Core | NYLF |  |  |  |
| **Chropara** | Chropara^(4)^ | Chronic bee paralysis | YP_001911136.1 | - | - | - | 462-485 | αI- αJ | INIFTRLRRHAMLFARAIRHTLAR |
|  |  |  |  | 501 | additional region conserved in Chropara (αJ) | T | 494-511 | αJ | RPILRQFTPWAFKSITMP |
|  |  | Anopheline-Associated C | AGW51752.1 | 3-7 | Upstream of MTase-GTase | IKKII | - | - | - |
|  |  |  |  | 55-59 | Upstream of MTase-GTase | AANSL | - | - | - |
|  |  |  |  | - | - | - | 493-510 | αJ | YVFRHPLSALTPWLFRRV |
|  | Sinai^(4)^ | Lake Sinai 1 | AEH26192.1 | 529-533 | Short region conserved in Chropara only, after αJ | KSAPW | 516-537 | αJ | SFVVSALHRFFGAKSAPWNWAS |
|  |  | Lake Sinai 2 | AEH26187.1 | 528-532 | Short region conserved in Chropara only, after αJ | KAAPW | 515-533 | αJ | SYVLNVLHRFFGAKAAPWN |
| **Santeuil** | Unclassified | Santeuil noda |  | - |  |  |  |  |  |
|  |  | Orsay |  | - |  |  |  |  |  |
|  |  | Leblanc noda |  | - |  |  |  |  |  |

(1) The term “viridae” is omitted from the name of the family

(2) The term “virus” is omitted from the name of the genus and of the species

(3) In the last row (sequence of amphipathic helices predicted by Heliquest), any helix predicted by Amphipaseek (7^th^ row) is underlined.

(4) Proposed name.

**Table S8D: Predicted transmembrane regions in the Noda, Chropara and Santeuil groups**

| **Group** | **Genus**^(1)^ | **Species** ^(1)^ | **Predicted transmembrane region** | |
| --- | --- | --- | --- | --- |
|  |  |  | **Boundary**  **(aas)** | **Location within**  **MTase-GTase** |
| **Noda** | Alphanoda | Alphanoda HB-2007/CHN | - |  |
|  |  | Flock House | 17-34 | Upstream of MTase-GTase |
|  |  | Nodamura | - |  |
|  |  | Pariacoto | - |  |
|  | Betanoda | Striped jack nervous necrosis | - |  |
|  |  | Epinephelus tauvina (=greasy grouper) nervous necrosis | - |  |
|  |  | Atlantic halibut noda | - |  |
|  |  | Macrobrachium Rosenbergii Noda | - |  |
|  |  | Wuhan Noda  (also called “Pieris rapae ”) | - |  |
|  |  | Plasmopara Halstedii A | - |  |
|  |  | Tetno 1 | - |  |
|  |  | Tetno 2 | - |  |
|  |  | Sclerophthora Macrospora A | 238-259 | βI- αE |
|  |  | Bat Guano associated noda GF-4n | 29-45 | Upstream of MTase-GTase |
|  |  | Betego SF | - |  |
| **Chropara** | Chropara^(2)^ | Chronic bee paralysis | 85-103 | Upstream of MTase-GTase |
|  |  | Anopheline-Associated C | 93-112 | Upstream of MTase-GTase |
|  | Sinai^(2)^ | Lake Sinai 1 | 128-153  496-520 | Upstream of MTase-GTase  αI |
|  |  | Lake Sinai 2 | 137-154  497-521 | Upstream of MTase-GTase  αI |
| **Santeuil** | - | Santeuil noda | 42-59 | Upstream of RdRP |
|  |  |  | 84-101 | Upstream of RdRP |
|  |  | Orsay | 47-70  82-100 | Upstream of RdRP |
|  |  | Le Blanc noda | 91-114 | Upstream of RdRP |

(1) the term “virus” is omitted from the genus and species name.

(2) Proposed name.
